# Identification of a three-long noncoding RNA prognostic model involved competitive endogenous RNA in kidney renal clear cell carcinoma

**DOI:** 10.1186/s12935-020-01423-4

**Published:** 2020-07-17

**Authors:** Di Zhang, Song Zeng, Xiaopeng Hu

**Affiliations:** 1 Department of Urology, Beijing Chao-Yang Hospital, Capital Medical University, No. 8 GongTi South Road, 100020 Beijing, China; 2 Institute of Urology, Capital Medical University, Beijing, China

**Keywords:** TCGA, KIRC, ceRNA, lncRNA prognostic model, Nomogram

## Abstract

**Background:**

Long noncoding RNA (lncRNA) is generally identified as competing endogenous RNA (ceRNA) that plays a vital role in the pathogenesis of kidney renal clear cell carcinoma (KIRC), the most common subtype of renal cell carcinoma with poor prognosis and unclear pathogenesis. This study established a novel ceRNA network and thus identified a three-lncRNA prognostic model in KIRC patients.

**Methods:**

Differentially expressed genes (DEGs) were screened out from The Cancer Genome Atlas (TCGA) database. The lncATLAS was applied to determine the differentially expressed lncRNAs (DElncRNAs) of the cytoplasm. The miRcode, miRDB, miRTarBase, and TargetScan databases were utilized to predict the interactions of DElncRNAs, DEmiRNAs, and DEmRNAs. Cytoscape was used to construct the ceRNA network. Then, a lncRNA prognostic model (LPM) was constructed based on ceRNA-related lncRNA that was significantly related to overall survival (OS), and its predictive ability was evaluated. Moreover, an LPM-based nomogram model was constructed. The significantly different expression of genes in the LPM was validated in an independent clinical cohort (N = 21) by quantitative RT-PCR.

**Results:**

A novel ceRNA regulatory network, including 73 lncRNAs, 8 miRNAs, and 21 mRNAs was constructed. Functional enrichment analysis indicated that integral components of membrane and PI3K–Akt signaling pathway represented the most significant GO terms and pathway, respectively. The LPM established based on three lncRNAs (MIAT, LINC00460, and LINC00443) of great prognostic value from the ceRNA network was proven to be independent of conventional clinical parameters to differentiate patients with low or high risk of poor survival, with the AUC of 1-, 5- and 10-year OS were 0.723, 0.714 and 0.826 respectively. Furthermore, the nomogram showed a better predictive value in KIRC patients than individual prognostic parameters. The expression of MIAT and LINC00460 was significantly upregulated in the KIRC samples, while the expression of LINC00443 was significantly downregulated compared with the adjacent normal samples in the clinical cohort, TCGA, and GTEx.

**Conclusion:**

This LPM based on three-lncRNA could serve as an independent prognostic factor with a tremendous predictive ability for KIRC patients, and the identified novel ceRNA network may provide insight into the prognostic biomarkers and therapeutic targets of KIRC.

## Background

Kidney renal clear cell carcinoma (KIRC) is the most common and aggressive malignant subtype of renal cell carcinoma that has a poor prognosis and high mortality in an advanced stage due to the lack of useful biomarkers and treatments [1]. Currently, there is a multitude of established treatments for KIRC, such as surgical resection, nonspecific immune approach, targeted therapy against vascular endothelial growth factor, and novel immunotherapy agents. Despite these treatments, about 50% of KIRC patients develop metastases, and the 5-year survival rate of these patients is still lower than 10% [2]. At present, the commonly used clinical prognostic markers of KIRC include the pathological grade system and tumor node metastasis (TNM) stage, microvascular invasion, tumor necrosis, and invasion of the collecting system [3]. These clinicopathological risk factors exhibit valuable but insufficient prediction of prognosis and estimation for subsets of KIRC patients. Previous researches have established some prognostic models and nomograms that incorporate necrosis, blood tests such as lactate dehydrogenase, hemoglobin, platelets, and calcium levels, prior nephrectomy, symptoms, and performance status [4, 5]. However, due to the intratumor heterogeneity of KIRC, these prognostic models cannot accurately predict the prognosis of KIRC patients in different subsets [6]. Therefore, the identification of useful prognostic biomarkers and therapeutic targets is urgingly needed to predict more accurately and improve the outcome of KIRC patients.

Long noncoding RNA (lncRNA), broadly defined as noncoding RNA molecules longer than 200 nucleotides, has gained emerging attention in cancer biology due to their direct and indirect regulatory roles [7]. It has been reported to be aberrantly expressed in a broad spectrum of tumors, leading to tumor initiation and progression [8]. Hence, they may serve as promising a new type of biomarkers for tumor diagnosis, prognosis, even in targeted gene therapy [9]. More recently, extensive research has explored the lncRNA expression profiling in the KIRC with the development of sequencing technology [10–12]. The determination of their interaction with other molecules and functional analysis is also widely investigated in recent years [13]. Notably, the subcellular localization of lncRNAs holds valuable clues to their molecular function [14]. In the cell nucleus, lncRNA could modulate nuclear functions, such as transcription, chromatin regulation, and variable splicing [15]. While in the cytoplasm, lncRNA could modulate mRNA mainly through the competitive endogenous RNA (ceRNA) regulation mechanism, according to the ceRNA hypothesis proposed by Salmena et al. in which lncRNA could compete with miRNA as a natural sponge and therefore indirectly regulate mRNA expression [16, 17]. Since proposed, numerous studies have investigated and validated that this lncRNA–miRNA–mRNA regulatory mechanism participates in tumor occurrence, progression, and metastasis and the potential prognosis of colorectal cancer, hepatocellular carcinoma, and lung cancer [18–20]. However, the overall regulatory functions of the lncRNA–miRNA–mRNA ceRNA network remain unclear, and the predictive accuracy of the prognostic model based on multiple lncRNAs’ expression is virtually worthy of exploring in KIRC patients.

In this study, we constructed a ceRNA network to elucidate the potential interaction of differentially expressed lncRNAs (DElncRNAs), DEmiRNAs, and DEmRNAs in KIRC. The subcellular localization of lncRNA was restricted to be cytoplasm in this network. We also explored the vital role of the network in KIRC. Subsequently, we identified the potential prognostic values of lncRNAs included in the ceRNA network and confirmed a lncRNA prognostic model (LPM) signature, which was established based on three lncRNAs of great prognostic value for overall survival (OS), as an independent prognostic biomarker. Then, we demonstrated a nomogram with better prediction value in KIRC patients than individual prognostic parameters. In the end, an independent clinical cohort was used to verify the significantly different expression of genes in the LPM by RT-PCR.

## Methods

### Data source and processing

The level 3 gene expression files and the corresponding clinical information for 530 KIRC patients, and miRNA expression files for 516 KIRC patients were retrieved from The Cancer Genome Atlas (TCGA) database (https://portal.gdc.cancer.gov/) (up to October 10, 2019). Sequencing was performed by Illumina HiSeq RNA-Seq and Illumina HiSeq miRNA-Seq platforms, respectively. Among these KIRC patients, a total of 539 KIRC samples and 72 adjacent normal samples in RNA sequence data, 545 KIRC samples and 71 adjacent normal samples in miRNA sequence data were subjected to subsequent analyses. The annotation data (antisense, lincRNA, and sense_intronic/sense overlapping, lncRNAs, 3′ overlapping ncRNAs, processed transcripts, antisense, and sense intronic) of probes of the TCGA RNA sequence data was recognized as lncRNA, and annotation data (protein-coding) as mRNA by using the GENCODE database (https://www.gencodegenes.org/). Then, the gene symbols were annotated based on the Homo_sapiens.GRCh38.84.chr.gtf file, which was downloaded from the Ensembl database (https://asia.ensembl.org/index.html).

### Identification of differentially expressed genes (DEGs)

We compared the KIRC samples and adjacent normal samples to identify DEGs by using DESeq 2 R package (Version 1.27.19; http://www.bioconductor.org/packages/devel/bioc/html/DESeq2.html) with a rigorous threshold as |log2-fold change (FC)| > 2.0 and FDR < 0.01 [21]. Then a heat map and volcano plot were drawn by using the heatmap R package (Version 1.0.1; https://www.rdocumentation.org/packages/pheatmap) and ggpubr R package (Version 0.2.4; https://www.rdocumentation.org/packages/ggpubr) in R software (Version 3.6.0; https://www.r-project.org/), to visualize the hierarchical clustering analysis of the identified DEGs.

### Construction of the ceRNA network

The lncATLAS database (http://lncatlas.crg.eu/) was used to identify the DElncRNAs located in the cytoplasm [22]. Then the DEmiRNAs which potentially interacted with DElncRNAs located in the cytoplasm were predicted using the miRcode (http://www.mircode.org/), a comprehensive searchable map of putative microRNA target sites in the long noncoding transcriptome [23]. Subsequently, the target DEmRNAs of DEmiRNA were predicted using miRDB (http://mirdb.org/), miRTarBase (http://mirtarbase.mbc.nctu.edu.tw/php/index.php) and TargetScan (http://www.targetscan.org/vert_72/) databases [24–26]. After that, Cytoscape software (Version 3.7.2; http://www.cytoscape.org/) was utilized to visualize and construct the ceRNA network [27].

### Functional enrichment analysis

The pathway and functional enrichment analysis were carried out by utilizing KO-Based Annotation System (KOBAS) (Version 3.0; http://kobas.cbi.pku.edu.cn/) and the Database for Annotation, Visualization and Integrated Discovery (DAVID) (Version: 6.8; https://david.ncifcrf.gov/), to investigate the potential biological implications of the ceRNA network [28, 29]. Significant GO terms and pathways were visualized by the GOplot (Version 1.0.2; https://cran.r-project.org/web/packages/GOplot/index.html) and ggalluvial (Version 0.9.1; https://www.rdocumentation.org/packages/ggalluvial) R packages, respectively.

### Construction and validation of a lncRNA-related prognostic model

Among 530 KIRC patients with RNA-sequencing data and clinical information, 514 KIRC patients were subjected to subsequent analyses after excluded according to the following criteria: (1) patients without survival information including survival time and status; (2) patients without complete lncRNA expression data; (3) patients who did not meet endpoint with following time less than 30 days. The expression files of DElncRNAs involved in the ceRNA network from 514 KIRC patients were analyzed through univariate Cox regression analysis in which genes were regarded as significant at *P* < 0.001, to identify the prognostic value of the DElncRNAs for OS. Then, the least absolute shrinkage and selection operator (LASSO) model with L1-penalty, performed by using the glmnet R package (Version 2.0-16; https://www.rdocumentation.org/packages/glmnet), was utilized to further select crucial lncRNAs from the prognostic DElncRNAs. In this method, a sub-selection of lncRNAs involved in KIRC patient prognosis was identified by shrinkage of the regression coefficient. Eventually, quite a few indicators with a weight of nonzero remained, and most of the potential indicators were shrunk to zero. In this process, we subsampled the data set 1000 times and selected the lncRNAs that were repeated > 900 times [30]. Finally, a risk-score based LPM was established using the regression coefficients from the multivariate Cox regression analysis in which genes were regarded as significant at *P* < 0.01. The formula of the risk score was constructed as follows:

$$ Risk\;score = \mathop \sum \limits_{i = 1}^{N} \left( {\beta *x} \right). $$

β stands for the regression coefficient of genes, X represents the expression level of genes, and N is the number of significant genes derived from the multivariate Cox regression analysis. The univariate and multivariate Cox regression analysis was performed utilizing the survival R package (Version 3.1-8; https://www.rdocumentation.org/packages/survival). Subsequently, the X-tile 3.6.1 software was applied to calculate the optimal cutoff to classify the KIRC patients into high- and low-risk groups [31]. Risk heatmap applying pheatmap R package (Version 1.0.12; https://www.rdocumentation.org/packages/pheatmap) was employed to cluster the expression files of core lncRNAs, which constructed the LPM, in the high- and low-risk groups. Then, the Kaplan–Meier (K–M) survival analysis and time‐dependent receiver operating characteristic (ROC) curves were used to evaluate the ability of the LPM to predict OS and disease-specific survival (DSS) by utilizing the survminer (Version 0.4.3; https://www.rdocumentation.org/packages/survminer) and survival ROC (Version 1.0.3; https://www.rdocumentation.org/packages/survivalROC) R package, respectively.

### Independence of the LPM from traditional clinical features

514 KIRC patients with complete lncRNA expression data, survival information, and complete clinical information, including age, gender, pathologic stage, and histologic grade, were subjected to subsequent univariate and multivariate Cox regression analyses, to assess the independent prognostic ability of the LPM for KIRC patients. To better visualize the prognostic value of risk score and clinical features, the forest plot was performed by using the ggplot2 R package (Version 3.2.1; https://www.rdocumentation.org/packages/ggplot2).

### Construction and validation of the nomogram

To further determine the predictive accuracy of model efficiency for 1-, 5-, 10-year, we constructed a novel nomogram, contained significant clinical features and calibration plots, based on the results of the multivariate Cox analysis utilizing the rms R package (Version 5.1-4; https://cran.r-project.org/web/packages/rms/index.html). The concordance index (C-index) was applied to evaluate the discrimination of the nomogram, and it was corrected by a bootstrap method with 1000 resamples [32]. Besides, the C-index and time-dependent ROC curves were performed to compare the predictive accuracies of the nomogram and individual prognostic factors. Besides, decision curve analysis (DCA) was performed to assess the clinical utility of the nomogram by comparing the benefits of different models.

### Gene set enrichment analysis (GSEA)

GSEA (Version 4.0; http://software.broadinstitute.org/gsea/index.jsp) was performed between high- and low-risk groups to identify the potential biological function of LPM [33]. An annotated gene set file (c5.bp.v7.0.entrez.gmt) was chosen as the reference gene set. The threshold was set at levels of |NES| > 2 and *P* < 0.01.

### Clinical samples and quantitative RT-PCR

In the clinical validation set, a total of 21 KIRC patients with KIRC samples and adjacent normal samples were selected according to the following criteria: (1) patients treated in the Beijing Chao-Yang Hospital; (2) patients who did not undergo treatment before surgery. Two individual experienced pathologists confirmed the final diagnosis of samples through identifying the morphology of the samples stained with H&E. This study was carried out in accordance with The Code of Ethics of the World Medical Association (Declaration of Helsinki). All patients signed the informed consent, and this study was approved by the ethics committees of Beijing Chao-Yang Hospital.

Total RNA of samples was extracted using the Trizol method, and then we synthesized cDNA via reverse transcription using the HiScript III RT SuperMix Kit (R323-01, Vazyme, China). The expression levels of lncRNAs were quantitated using the AceQ qPCR SYBR Green Master Mix (R323-01, Vazyme, China) by the ABI 7500 real-time PCR system (Applied Biosystems, Foster City, CA, USA). The primers used in this study are listed in Additional file 1: Table S1. Target lncRNA levels were normalized against GAPDH standards and calculated using the 2-ΔΔCt method.

### Validation of lncRNAs expression

The expression levels of the three lncRNAs were verified in KIRC patients with paired KIRC samples and adjacent normal samples from the Beijing Chao-Yang cohort (N = 21) and TCGA cohort (N = 72), respectively, by using Wilcoxon signed-rank test. Additionally, the different expression analysis was further performed in KIRC samples (N = 523) from TCGA and normal samples (N = 100) from the match TCGA normal and Genotype-Tissue Expression (GTEx) data by utilizing Gene Expression Profiling Interactive Analysis (GEPIA2) (http://gepia2.cancer-pku.cn/#index) [34]. *P *<0.05 was considered significant, and all statistical tests were two-sided.

## Results

### Identification of differentially expressed genes between KIRC samples and adjacent normal samples

**Fig. 1:**
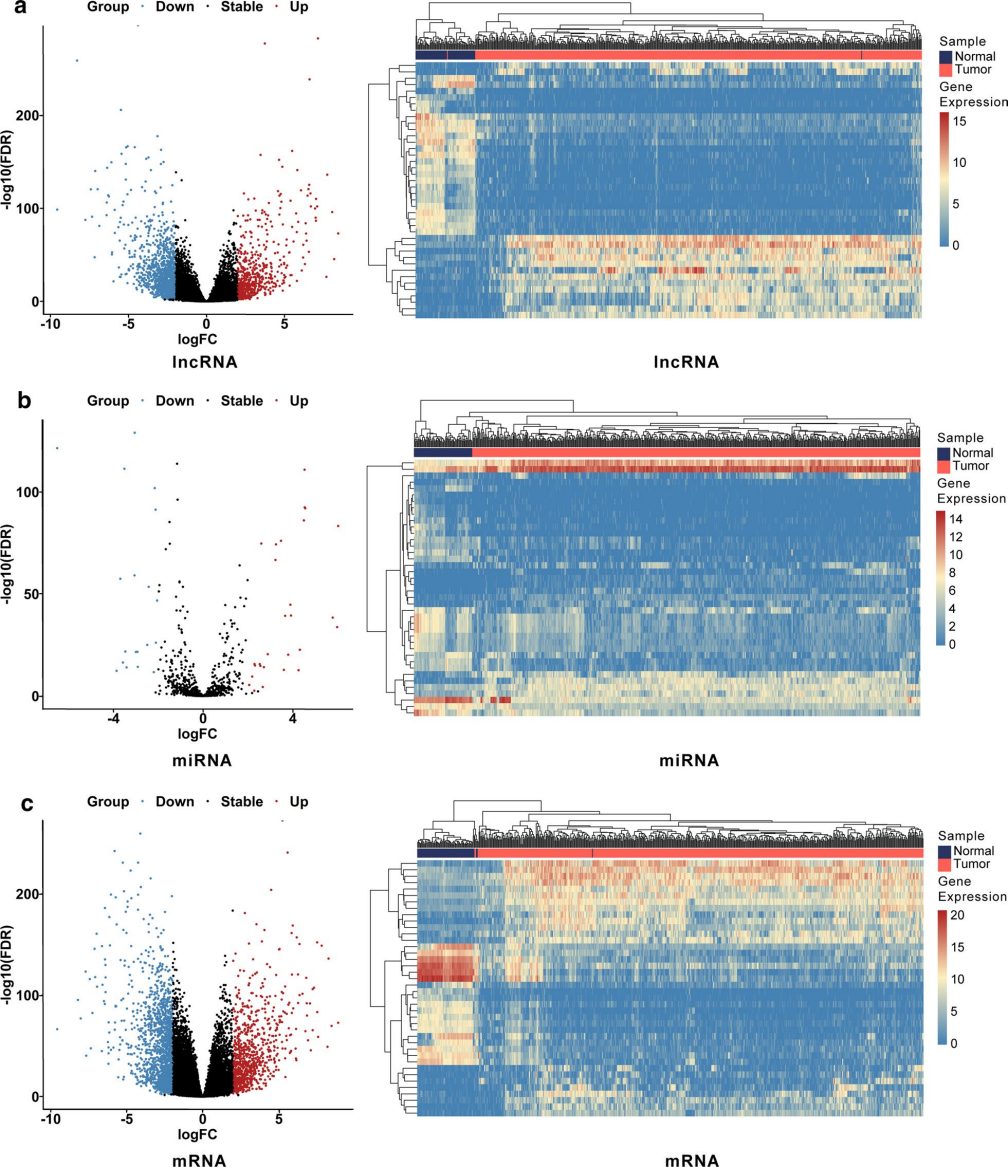
Identification of differentially expressed genes. Volcano plots and heatmap plots of differentially expressed lncRNAs (**a**), miRNAs (**b**), and mRNAs (**c**) between KIRC samples and adjacent normal samples. Red denotes upregulated genes, and blue denotes downregulated genes in both volcano plots and heatmaps. The horizontal axis of the heatmaps represents the samples, and the vertical axis of heatmaps presents the top forty DEGs

A total of 539 KIRC samples and 72 adjacent normal samples were utilized to screen DElncRNAs and DEmRNAs; 545 KIRC samples and 71 adjacent normal samples were utilized to screen DEmiRNAs. The DESeq 2 R package was performed to identify differentially expressed genes with a strict cutoff threshold of |log2 FC | > 2 and an adjusted *P *<0.01. Compared with normal samples, 2015 DElncRNAs, 47 DEmiRNAs, and 2314 DEmRNAs were differentially expressed, among which 1461 lncRNAs, 19 miRNAs, and 1511 mRNAs were upregulated as well as 554 lncRNAs, 28 miRNAs and 803 mRNAs were downregulated (Additional file 1: Table S2). The heat maps and volcano plots of DEGs between KIRC samples and adjacent normal samples were shown in Fig. 1.

### Construction of the ceRNA network

**Fig. 2:**
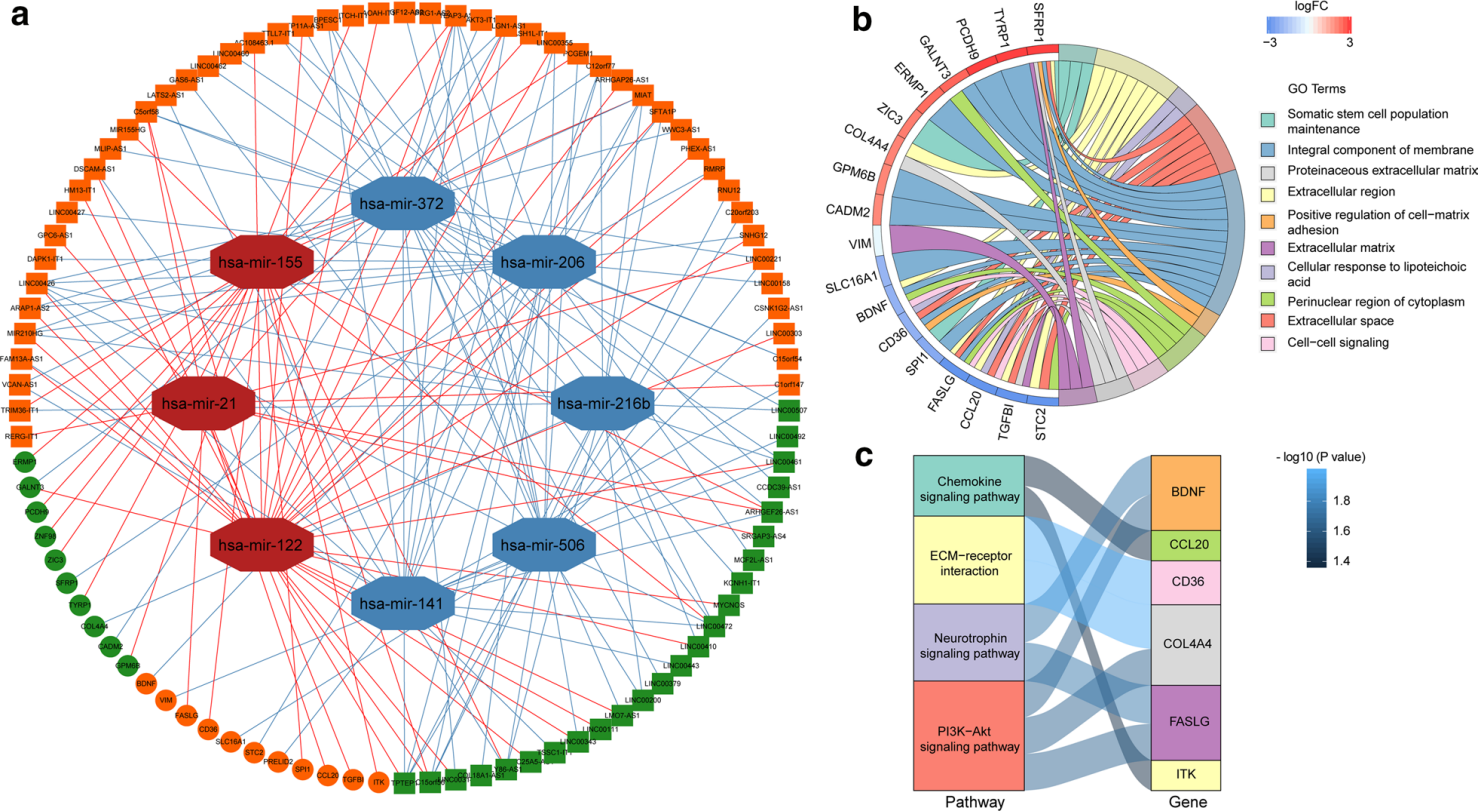
Construction and functional enrichment analysis of the ceRNA network. **a** The ceRNA regulatory network in KIRC. Red octagons represent upregulated miRNAs, and Blue octagons represent downregulated miRNAs. Orange circles and squares stand for upregulated mRNAs and lncRNAs, respectively. Green circles and squares present downregulated mRNAs and lncRNAs, respectively. **b** Chord plot of GO enrichment analysis for the ceRNA network. The GO terms are shown and annotated on the right of the chord diagram, while the DEmRNAs in the ceRNA network are shown on the left. Red bricks represent upregulated genes, and blue bricks represent downregulated genes. **c** Sankey diagram of KEGG pathway analysis for the ceRNA network. Rectangles on the left of the Sankey plot represent the significant pathways, while rectangles on the right represent DEmRNAs in the ceRNA network

Considering the nuclear-cytoplasmic localization of lncRNAs plays a vital role in its molecular function, we firstly confirmed the subcellular localization of the 2015 DElncRNAs by utilizing the lncATLAS database, and excluded 385 DElncRNAs which were located only in the nucleus because the endogenous competition role of lncRNAs is mainly exhibited in the cytoplasm. The detailed distribution information for the DElncRNAs was shown in Additional file 1: Table S3. Then the remained DElncRNAs were put into the miRcode database to identify the potential miRNAs targeting lncRNAs. However, only 12 out of predicted miRNAs were selected after taking the intersection with 47 DEmiRNAs. We then utilized the databases of miRDB, miRTarBase, and TargetScan to identify the downstream target mRNAs with reference to the 12 DEmiRNAs. In addition, we selected potential mRNAs that only shared by all three databases to enhance the veracity of the prediction. The results showed that 21 out of 2314 DEmRNAs were identified. Finally, a total of 73 DElncRNAs, 8 DEmiRNAs, and 21 DEmRNAs were eventually incorporated into the KIRC-associated ceRNA regulatory network by applying Cytoscape software (Fig. 2a, Additional file 1: Table S4).

To further reveal the biological functions and pathways associated with the ceRNA network, GO and KEGG enrichment analysis was performed via KOBAS and DAVID. The results of GO analysis indicated that the DEmRNAs involved in the ceRNA network were mainly enriched in the “Integral component of membrane”, “Extracellular region” and “Extracellular space” (Fig. 2b). Moreover, the results from KEGG analysis showed that the DEmRNAs were particularly enriched in “ECM-receptor interaction”, “Chemokine signaling pathway”, “PI3K–Akt signaling pathway” and Neurotrophin signaling pathway (Fig. 2c).

### Construction of an LPM and evaluation of its predictive ability

**Fig. 3:**
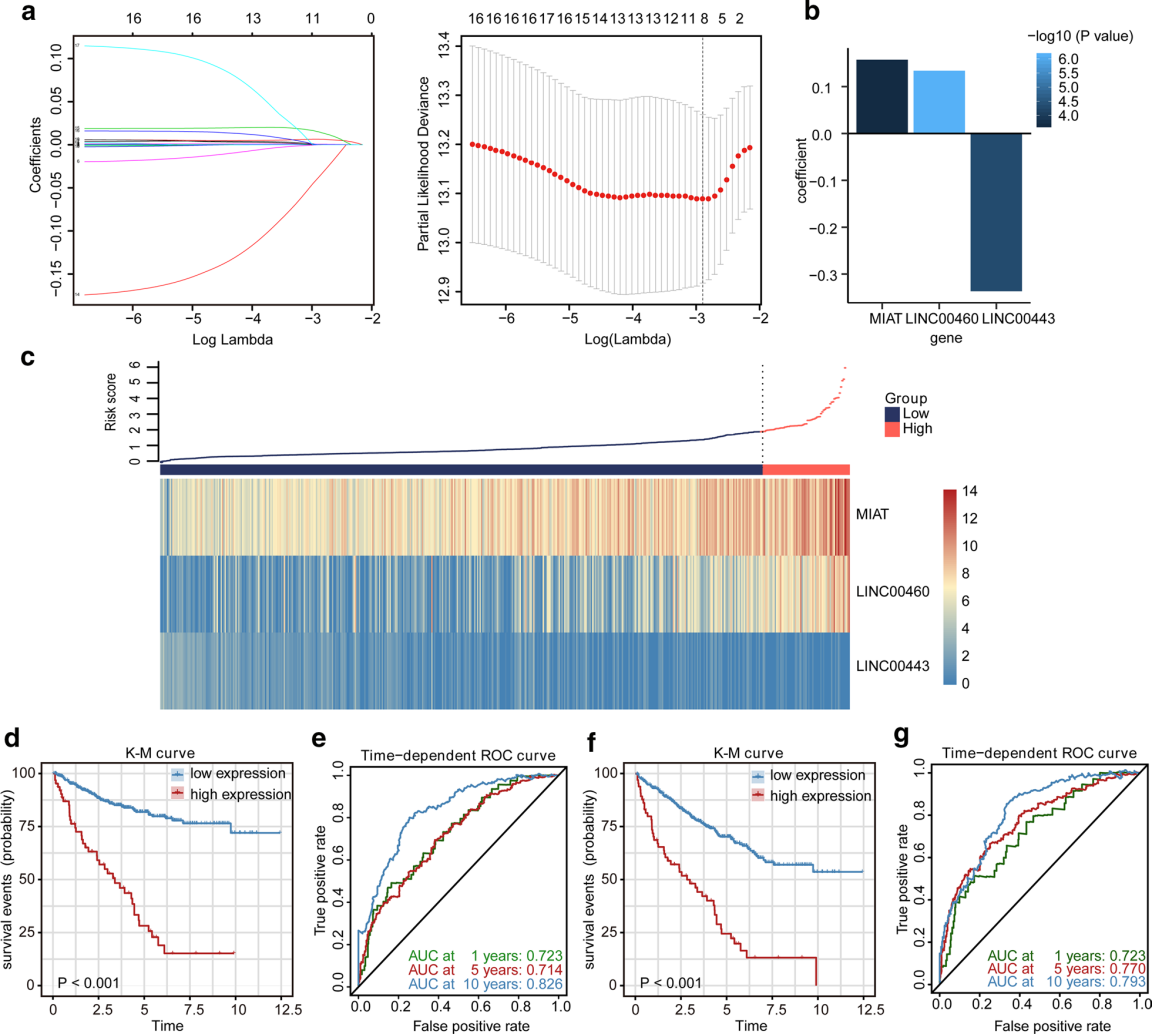
The lncRNA prognostic model. **a** Lasso-penalized Cox regression analysis of 17 DElncRNAs. Ten-fold cross-validation was applied to calculate the best lambda, which leads to a minimum mean cross-validated error. **b** The bar plot shows coefficients of three lncRNAs in the LPM. The color of the bars represented the P-value of the coefficients. **c** Risk score system of the LPM. The above scatter plot exhibits the risk scores of each KIRC patient with survival data, and the below heatmap displays the expression profiles of three lncRNAs in the LPM. K–M survival curves and time-dependent ROC curves of OS are shown in **d** and **e**, respectively. K–M survival curves and time-dependent ROC curves of DSS are demonstrated in **f** and **g**, respectively

To consider whether those DElncRNAs involved in the ceRNA network were closely related to the OS of KIRC patients in the TCGA cohort, we performed the univariate Cox regression to identify the prognostic value of the DElncRNAs for OS. The result indicated that 17 of the 73 DElncRNAs were significantly related to OS (Additional file 1: Table S5). To further find crucial lncRNAs from the prognostic DElncRNAs, we applied LASSO estimation and selected 8 lncRNAs which appeared > 900 times out of 1000 repetitions (Fig. 3a). Subsequently, multivariate Cox regression analysis was utilized to select lncRNAs with the best prognostic value and calculate their relative coefficients, to further establish a risk-score based LPM. Finally, we constructed the LPM to predict patient survival with the risk score of each patient calculated as follows: risk score = (0.13383 × expression level of LINC00460) + (− 0.33667 × expression level of LINC00443) + (0.15751 × expression level of MIAT) (Fig. 3b). Furthermore, we applied the X-tile software to find the optimal cutoff value of risk scores, and patients with risk scores greater than 2.08 (n = 65) were classified into the high‐risk group, while those with risk scores less than or equal to 2.08 (n = 449) were allocated to the low‐risk group. The risk score distribution and lncRNA expression data are shown in Fig. 3c. The K-M survival analysis indicated that the high-risk patients had a shorter OS than the low-risk patients (Fig. 3d). Additionally, the high-risk group showed a 3.768-fold higher risk [95% confidence interval (CI) 2.254–6.299, *P *<0.001)] than the low-risk group. The time-dependent ROC curve analysis validated the great prognostic value of the LPM (Fig. 3e). The area under the ROC curve (AUC) of the LPM for OS was 0.723 at 1 year, 0.714 at 5 years, and 0.826 at 10 years. Besides, the high-risk patients had a shorter DSS than the low-risk patients with a 5.561-fold higher risk (95% CI 2.929–10.560, *P *<0.001), and the AUC of the LPM for DSS was 0.723 at 1 year, 0.770 at 5 years and 0.793 at 10 years (Fig. 3f, g).

### High risk indicated an enhanced immune phenotype

**Fig. 4:**
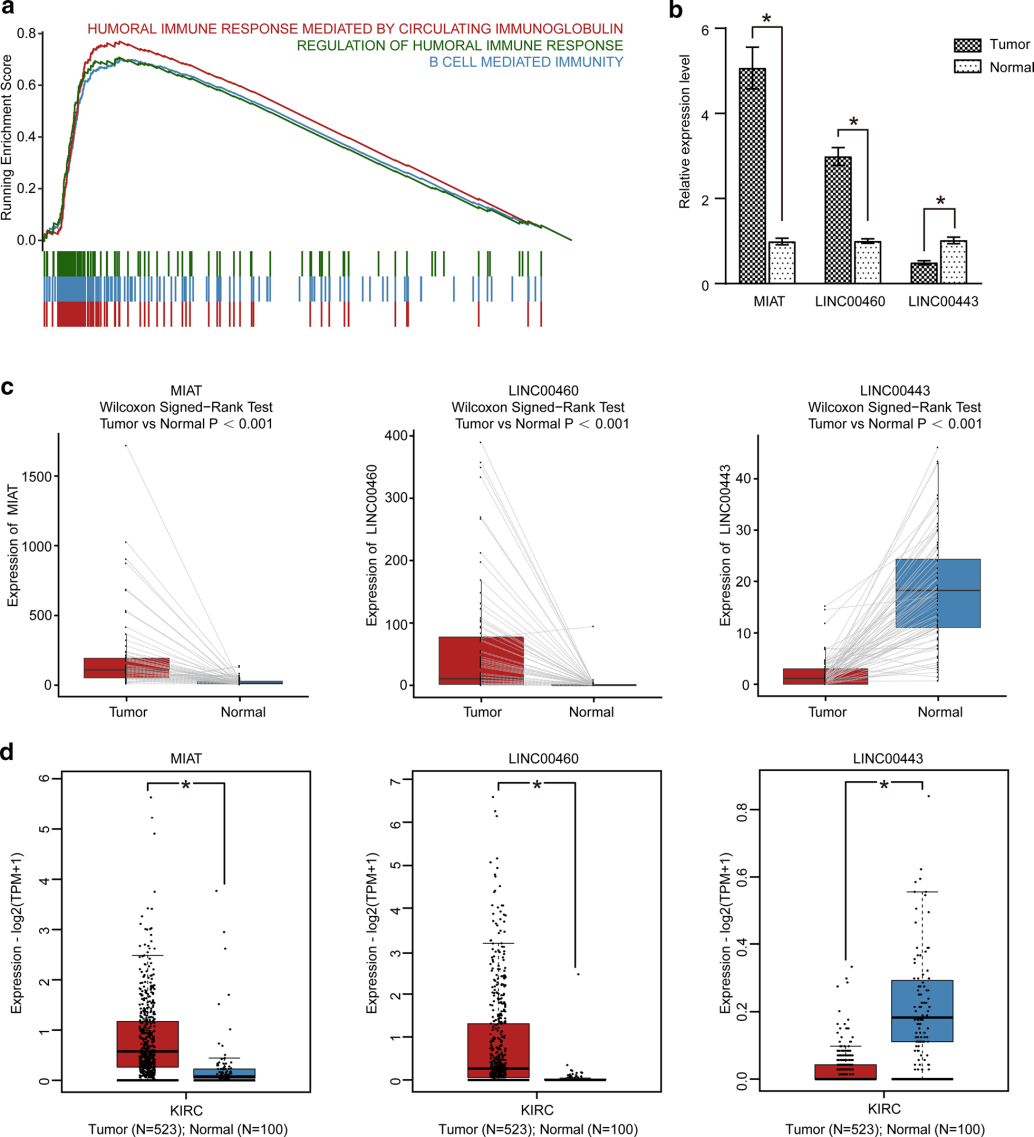
GSEA and validations of three lncRNAs. **a** GSEA plot shows the top three immune processes associated with high risk score. **b** The barplot exhibits the expressions of MIAT, LINC00443, and LINC00460 evaluated by RT-PCR in 21 KIRC samples and paired adjacent normal samples. ∗P < 0.05 versus control. **c** The differential expression patterns of three lncRNAs in 72 KIRC patients with paired KIRC and adjacent normal samples from TCGA. **d** The expression profiles of three lncRNAs were further compared between the TCGA KIRC cohort (523 KIRC samples) and the GTEx cohort (100 normal kidney samples) via GEPIA2

To explore the biological pathways associated with the LPM, GSEA was conducted between the 65 high-risk and 449 low-risk KIRC patients in the TCGA KIRC cohort. The result revealed that the high-risk patients were significantly related to 223 biological processes (Additional file 1: Table S6), in which the top 3 immune processes were HUMORAL_IMMUNE_RESPONSE_MEDIATED_BY_CIRCULATING_IMMUNOGLOBULIN (NES = 3.512, size = 136), B_CELL_MEDIATED_IMMUNITY (NES = 3.364, size = 199) and REGULATION_OF_HUMORAL_IMMUNE_RESPONSE (NES = 3.172, size = 124) (*P *<0.01) (Fig. 4a). Therefore, the high-risk score may confer an enhanced immune phenotype.

### Validation of lncRNAs in the clinical cohort, TCGA, and GTEx

According to the above analysis in the TCGA KIRC cohort, the three lncRNAs (LINC00460, LINC00443, MIAT) involved in the ceRNA network could serve as potential prognostic predictors for KIRC patients. To verify the validity and reliability of the results, the expression of the three lncRNAs were analyzed between 21 diagnosed KIRC samples and 21 adjacent normal samples through RT-PCR. As shown in Fig. 4b, the results demonstrated that the expression of MIAT and LINC00460 was significantly upregulated in the KIRC samples, while the expression of LINC00443 was significantly downregulated compared with the adjacent normal samples. In addition, we performed the difference analysis of three-lncRNA expression by using the TCGA paired samples and GEPIA2, to verify the expression of MIAT, LINC00460, LINC00443 further. A consistent finding with that of the clinical KIRC cohort was elucidated (Fig. 4c, d).

### Stratification analysis of OS for the LPM

**Fig. 5:**
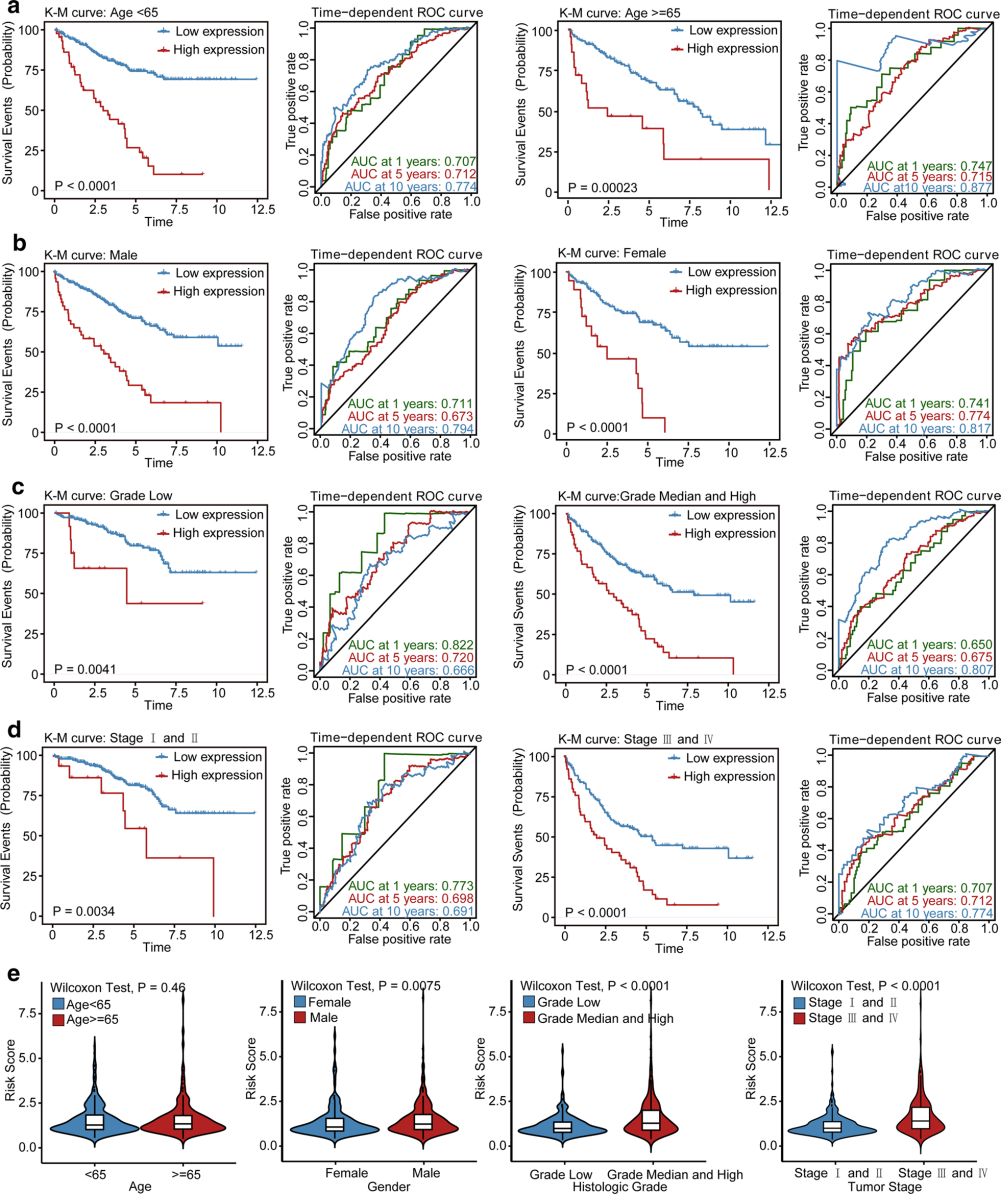
Stratification analysis of the LPM. Kaplan–Meier curves and time-dependent ROC curves illustrate the prognostic value of LPM based on the stratification of different clinical features. **a** age; **b** gender; **c** histological grade; **d** tumor stage; **e** violin plots exhibit the risk score distributions within different clinical parameters stratification

Stratification analysis was performed to determine whether the prognostic value of the LPM would remain stable in different subgroups. Therefore, patients in the TCGA KIRC cohort were classified into two groups according to age, sex, tumor grade, and tumor stage, respectively. As shown in Fig. 5, patients in the high-risk group showed worse survival compared to those in the low-risk group in patients with grade low or grade median and high tumors, stage I and II or stage III and IV tumors, younger or older, and male or female patients. Besides, the LPM still remained a stable and great predictive ability for KIRC patients in distinct subgroups.

### LPM is independent of traditional clinical features for KIRC Patients

**Fig. 6:**
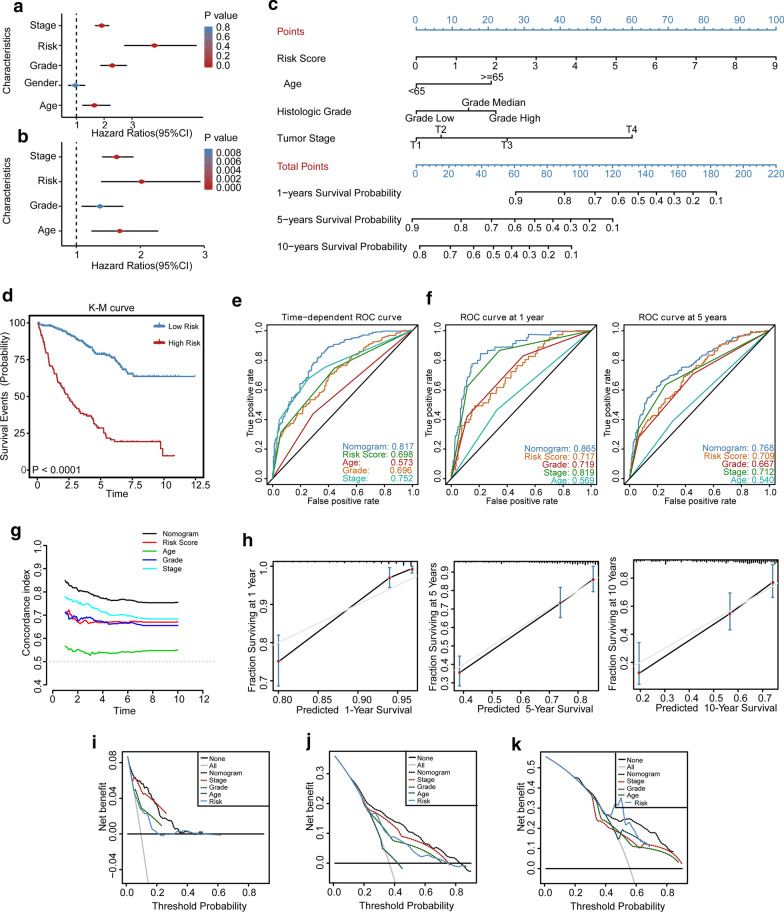
The LPM-based nomogram model. **a** Univariate and **b** multivariate regression analysis of the relation between the LPM and clinical features regarding prognostic value. **c** Nomogram for predicting the probability of 1-, 5-, and 10-year OS for KIRC patients. **d** Kaplan–Meier curves demonstrate the prognostic performance of the nomogram. **e**, **f** Time-dependent ROC curves of the nomogram, the LPM, age, histological grade, and tumor stage. **g** The prognostic performance was compared among the nomogram, the LPM, and conventional clinical characteristics by calculating the C-index. **h** Calibration plots of the nomogram for predicting the probability of OS at 1-, 5-, and 10-years. **i**–**k** DCA plots of the nomogram, the LPM, and clinical characteristics for predicting the probability oof OS at 1-, 5-, and 10-years

To identify whether the LPM is an independent clinical prognostic factor for KIRC patients, we firstly performed the univariate Cox regression analysis and demonstrated that the LPM was significantly associated with OS [Hazard ratio (HR): 3.809, 95% CI 2.720–5.330, *P *<0.001; Fig. 6a]. Then clinical characteristics, including gender, age, pathologic stage, and histological grade were adjusted by multivariate Cox regression analysis, and the result indicated that the LPM remained an independent prognostic factor with an HR of 2.020 in the TCGA KIRC cohort (95% CI 1.387–2.940, *P *<0.001; Fig. 6b).

### Construction and validation of an LPM-based nomogram model

Since other studies have confirmed that the nomogram model could predict the prognosis of cancer patients better [30, 35, 36], we established a nomogram model combining risk scores and independent clinical prognostic factors (age, histologic grade, and tumor stage) (Fig. 6c). According to this model, we can determine each point for factors by drawing a vertical line from the prognostic factor axis to the points axis to further calculate the total points. Similarly, we can determine the survival probability for total points by drawing a vertical line from the total points axis to the outcome axis. Based on the total nomogram points, we divided the patients into high-risk group and low-risk group with an optimal cutoff point 66, and the high-risk patients had a shorter OS than the low-risk patients with a 5.574-fold higher risk (95% CI 3.852–8.064, *P *<0.001; Fig. 6d). We then compared the predictive accuracy between the nomogram model and individual predictors with the time-dependent ROC curve analysis and C-index. The result indicated that the nomogram model had a better power for predicting the prognosis of KIRC patients with a larger AUC (Fig. 6e, f). Moreover, the nomogram model had a higher mean C-index (0.772) than other predictors (0.551 to 0.676) (Fig. 6g), which was consistent with the results of ROC analysis, further validating the robust prognostic value of the nomogram. The calibration plots demonstrated a good agreement between the observed and predicted probabilities with lines close to 45° (Fig. 6h). Finally, we compared the clinical benefit of the nomogram model and other individual predictors via DCA, a novel method for evaluating prediction models [37]. As a result, the nomogram model showed a better net benefit and broader threshold probability. Thus, it provided the best clinical utility compared with other independent prognostic factors (1-year OS: Fig. 6i; 5-year OS: Fig. 6j; 10-year OS: Fig. 6k).

## Discussion

As the most common and lethal subtype of renal carcinoma, KIRC is driven by distinct driver gene mutations and complex molecular alterations [38]. Though many effective treatments have been developed for KIRC, the unsatisfied survival rate and intolerant of chemotherapy make it an emerging need for new therapeutic targets and prognostic biomarkers to improve the clinical outcomes of KIRC patients in the future [39]. Previous research has demonstrated that lncRNAs located in the cytoplasm could regulate mRNA transcription indispensably, primarily through ceRNA regulatory networks, making these attractive molecules targets and prognostic biomarkers [40]. However, few studies have investigated the specific functions and prognostic value of lncRNAs involved in the ceRNA regulatory network in KIRC, especially lncRNAs located in the cytoplasm.

In this study, we established a lncRNA-miRNA-mRNA ceRNA regulatory network and constructed a novel three-lncRNA-based LPM that could identify KIRC patients who had a high risk of poor prognosis. Therefore, it is feasible to divide the KIRC patients into different subgroups with a particular risk score, and such patient stratification could clinically contribute to more individualized management for patients. The lncRNAs (MIAT, LINC00460, and LINC00443) that constitute the LPM could be regarded as individual targets in the future, and they may provide better performance in combination, depending on their prognostic significance. To facilitate the clinical use of the LPM, we further developed a nomogram to help clinicians conduct accurate risk assessment for individual KIRC patients. Since it consisted of the LPM and several independent clinical prognostic factors (age, histologic grade, and tumor stage), the nomogram can better account for intratumor heterogeneity of KIRC. Thus, it can accurately predict the prognosis of KIRC patients in different subsets.

An increasing number of researches have found that tumor initiation and progression can be primarily represented by DEGs [41, 42]. Thus, we firstly identified DEGs as candidate genes for the ceRNA network by using the DESeq 2 method, which has better statistical power in sensitivity and precision than edgeR and DESeq. To the best of our knowledge, our study is the first to use the DESeq 2 method in constructing the ceRNA network in KIRC, resulting in an inconsistent finding in the DEGs screen with other studies [35, 43]. Since the lncRNA–miRNA–mRNA interaction in the ceRNA network only presented in the cytoplasm [44], we excluded the lncRNAs that only located in the nucleus to enhance the accuracy of prediction. Based on these improved methods, we constructed a novel ceRNA network. In this network, the potential binding sites of the three lncRNA (LINC00443, LINC00460, and MIAT) on the targeted DEmiRNAs were showed in Additional file 1: Table S7. Moreover, in order to explore the potential biological function of the ceRNA network, we performed the KEGG pathway analysis. The result indicated that the function of the ceRNA network might be associated with the PI3K–Akt signaling pathway, a cancer-related pathway. Current studies have shown that the PI3K–Akt pathway is activated in many types of cancers. Recent large-scale integrated analyses have provided the genetic alteration rates of the PI3K–Akt pathway in KIRC patients, reiterating the critical role of the PI3K–Akt pathway [45]. Previous studies have reported that the PI3K–Akt pathway could closely connected with the VHL–HIF pathway, forming a large signaling network contributing to cell proliferation, migration, and invasion in KIRC [46].

In this study, we identified three lncRNAs of great prognostic value from the ceRNA network and further established a risk-score based LPM. Previous studies have reported several lncRNA-based prognosis models and nomograms of KIRC [35, 47, 48], but the LPM that we established had the following advantages. First, we selectively analyzed those DElncRNAs in the ceRNA network for their prognostic value. Because if lncRNA affected tumorigenesis with inconsiderable influences, their prognostic value would be diminished. Second, we performed the LASSO algorithm to further select crucial lncRNA. Third, the LPM contained only three lncRNAs whose differential expression pattern was further confirmed in our independent cohort. Fourth, the LPM scored above 0.70 for the AUC statistic, which was higher than that in other lncRNA-based prognosis models. These advantages ensured the accuracy of LPM and thus enhanced its feasibility of clinical transformation. Among the three lncRNAs in the LPM, LINC00460 is the most studied oncogenic lncRNA. On a large scale of cancer types, LINC00460 functions as a competing endogenous RNA, sponging multiple miRNAs, indicating that it plays a vital role in promoting tumor cell proliferation, migration, and invasion [49–51]. However, a rare study has explored the role of LINC00460 in KIRC, so this was the first study which found that high expression of LINC00460 is linked to poor prognosis in patients with KIRC. In the previous study, LINC00443 was reported as a tumor suppressor, which was associated with a better prognosis of KIRC, while MIAT was associated with worse prognosis [52–54]. In our study, expression and survival analysis of LINC00443 and MIAT revealed the high expression of MIAT and the low expression of LINC00443 in KIRC samples, which was related to poor prognosis and better prognosis, respectively. However, a further basic study needs to be undertaken to validate their molecular functions in the development of KIRC.

In addition, we revealed that the LPM was still an independent prognostic factor in KIRC patients after adjusting traditional clinical characteristics. This result indicated that LPM has the potential to improve the predictive power of traditional factors. Hence, we construct a nomogram model that combines the LPM and other independent clinical prognostic factors. The results of time-dependent ROC curve analysis, C-index, and DCA demonstrated the robust prognostic value of the nomogram for KIRC patients. The primary advantage of this model lies in developing a unique LPM based clinically associated risk scoring system for KIRC patients.

Our study provided new insight into developing a prognostic score system for KIRC patients. It can easily separate patients with poor prognosis from patients with good prognosis. Since this LPM consisted of only three lncRNAs which can be measured by PCR, it was convenient, cost-effective, and easy to use in clinical application. The nomogram, a unique LPM based clinically associated risk scoring system, had robust prognostic value for KIRC patients, and it could be a promising tool for clinicians in the future. Furthermore, clinicians can develop more individualized treatment regimens for patients with different prognosis assessed by nomogram, and this will make treatment more targeted. However, our study is limited because we only validated the expression of three lncRNAs in the individual clinical cohorts, and it would be better if clinical cohorts could validate the prognostic value of LPM. Besides, prospective studies are further needed to perform to confirm its predictive ability.

## Conclusions

In conclusion, we successfully constructed a novel ceRNA regulatory network, which narrowed the scope of predicting prognostic biomarkers and therapeutic targets for KIRC. Besides, we identified and validated an LPM which is based on three lncRNAs involved in the ceRNA network, and it has independent and great prognostic value for KIRC patients.

## Supplementary information

**Additional file 1: Table S1.** List of primers used for RT-PCR. **Table S2.** Differentially expressed lncRNAs, miRNAs, and mRNAs between KIRC samples and adjacent normal samples. **Table S3.** The subcellular distribution of lncRNAs in the ceRNA network. **Table S4.** The interactions of the ceRNA network in KIRC. **Table S5.** Seventeen lncRNAs associated with overall survival in KIRC. **Table S6.** The results of GSEA. **Table S7.** The potential binding sites of three lncRNAs (LINC00443, LINC00460 and MIAT) on the targeted DEmiRNAs in the ceRNA network.

## Data Availability

All data generated or analyzed during this study are included in this published article and its additional information files.

## Abbreviations

KIRC: Kidney renal clear cell carcinoma
lncRNA: Long noncoding RNA
ceRNA: Competitive endogenous RNA
DElncRNAs: Differentially expressed lncRNAs
LPM: lncRNA prognostic model
OS: Overall survival
TCGA: The Cancer Genome Atlas
LASSO: Least absolute shrinkage and selection operator
K-M: Kaplan–Meier
ROS: Receiver operating characteristic
DSS: Disease-specific survival
C-index: Concordance index
DCA: Decision curve analysis
GSEA: Gene set enrichment analysis
GTEx: Genotype-Tissue Expression
GEPIA2: Gene Expression Profiling Interactive Analysis
CI: Confidence interval
AUC: Area under the ROC curve
HR: Hazard ratio
