# Supplementary material for: Identification of a three-long noncoding RNA prognostic model involved competitive endogenous RNA in kidney renal clear cell carcinoma
Source: Cancer Cell Int. 2020 Jul 17;20:319. doi: 10.1186/s12935-020-01423-4 (PMC7367230; doi:10.1186/s12935-020-01423-4)
Supplement: Supplementary file 1 — Additional file 1: Table S1. List of primers used for RT-PCR. Table S2. Differentially expressed lncRNAs, miRNAs, and mRNAs between KIRC samples and adjacent normal samples. Table S3. The subcellular distribution of lncRNAs in the ceRNA network. Table S4. The interactions of the ceRNA network in KIRC. Table S5. Seventeen lncRNAs associated with overall survival in KIRC. Table S6. The results of GSEA. Table S7. The potential binding sites of three lncRNAs (LINC00443, LINC00460 and MIAT) on the targeted DEmiRNAs in the ceRNA network. [file 12935_2020_1423_MOESM1_ESM.docx]

**Additional Material**

**Table S1. List of primers used for RT-PCR**

| **Gene** | **Forward Sequence** | **Reverse Sequence** |
| --- | --- | --- |
| LINC00460 | ATGCACACTTCTCGGCTAAG | GGTCGTAACCTTCGTTCTCATC |
| LINC00443 | CAGAAGGTTGCTGTTCATTAC | CTCTTGGTCTTGAATAGGTGAT |
| MIAT | GGACGTTCACAACCACACTG | TCCCACTTTGGCATTCTAGG |
| GAPDH | ACAACTTTGGTATCGTGGAAGG | GCCATCACGCCACAGTTTC |

**Table S2. Differentially expressed lncRNAs, miRNAs, and mRNAs between KIRC samples and adjacent normal samples**

| **Gene** | **Type** | **Log2 fold change** | **Adjusted P value** |
| --- | --- | --- | --- |
| PVT1 | upregulated lncRNA | -4.384724333 | 0 |
| TTC21B-AS1 | upregulated lncRNA | -8.293695555 | 8.29E-256 |
| LINC00887 | upregulated lncRNA | -5.485440525 | 5.23E-203 |
| MIR210HG | upregulated lncRNA | -3.136861681 | 7.06E-175 |
| RP11-155G14.6 | upregulated lncRNA | -5.048895266 | 4.08E-164 |
| DGCR5 | upregulated lncRNA | -4.592749145 | 3.64E-163 |
| RP11-547D24.1 | upregulated lncRNA | -5.148169343 | 5.12E-163 |
| SLC16A1-AS1 | upregulated lncRNA | -2.91074103 | 1.99E-160 |
| RP11-513G11.2 | upregulated lncRNA | -5.450022965 | 3.03E-156 |
| DGCR9 | upregulated lncRNA | -3.714927355 | 2.25E-152 |
| GAS6-AS1 | upregulated lncRNA | -3.861037552 | 6.13E-151 |
| RP11-798M19.6 | upregulated lncRNA | -2.748043964 | 1.18E-147 |
| RP11-14C10.5 | upregulated lncRNA | -6.094822519 | 3.01E-147 |
| RP11-115N4.1 | upregulated lncRNA | -5.170503484 | 1.51E-145 |
| DARS-AS1 | upregulated lncRNA | -2.918641353 | 1.77E-145 |
| LINC00462 | upregulated lncRNA | -6.36771567 | 1.71E-141 |
| RP11-598F7.5 | upregulated lncRNA | -3.564999138 | 4.79E-139 |
| RP11-380J14.1 | upregulated lncRNA | -7.130594618 | 4.99E-138 |
| LINC01587 | upregulated lncRNA | -5.168144757 | 6.96E-129 |
| CTD-2015G9.2 | upregulated lncRNA | -3.90030207 | 6.91E-127 |
| RP11-356I2.4 | upregulated lncRNA | -2.189083118 | 7.23E-123 |
| CDKN2B-AS1 | upregulated lncRNA | -4.129847479 | 8.27E-121 |
| PHKA2-AS1 | upregulated lncRNA | -2.94036048 | 2.14E-120 |
| LINC01077 | upregulated lncRNA | -6.971452896 | 1.12E-118 |
| OSTM1-AS1 | upregulated lncRNA | -7.415374454 | 1.90E-118 |
| SFTA1P | upregulated lncRNA | -4.839170924 | 5.81E-118 |
| HIF1A-AS2 | upregulated lncRNA | -3.9441124 | 5.61E-117 |
| RP11-598F7.6 | upregulated lncRNA | -3.302391035 | 2.30E-116 |
| RP11-259N19.1 | upregulated lncRNA | -3.223844935 | 1.30E-114 |
| RP4-764O22.1 | upregulated lncRNA | -5.066404913 | 2.57E-113 |
| AP000439.3 | upregulated lncRNA | -6.298473282 | 2.87E-112 |
| RP6-191P20.4 | upregulated lncRNA | -6.252334108 | 4.54E-111 |
| RP11-529E15.1 | upregulated lncRNA | -5.323076233 | 1.43E-105 |
| RP11-848P1.3 | upregulated lncRNA | -2.268581111 | 1.43E-105 |
| AC114803.3 | upregulated lncRNA | -6.394041546 | 1.32E-104 |
| RP5-1120P11.1 | upregulated lncRNA | -2.78301989 | 2.40E-104 |
| SNHG12 | upregulated lncRNA | -2.801386651 | 6.77E-103 |
| LUCAT1 | upregulated lncRNA | -5.374155923 | 9.53E-102 |
| CTD-2026K11.6 | upregulated lncRNA | -3.31942969 | 1.09E-99 |
| AC079466.1 | upregulated lncRNA | -9.565858338 | 5.11E-97 |
| EGFR-AS1 | upregulated lncRNA | -5.879419922 | 9.42E-97 |
| RP11-434E6.4 | upregulated lncRNA | -2.829552779 | 4.69E-94 |
| MMP25-AS1 | upregulated lncRNA | -2.146837938 | 5.58E-94 |
| AC000095.11 | upregulated lncRNA | -3.376778233 | 1.44E-91 |
| AC078883.3 | upregulated lncRNA | -2.803640978 | 2.79E-91 |
| AC073115.6 | upregulated lncRNA | -6.404311511 | 3.37E-91 |
| AC133644.2 | upregulated lncRNA | -3.064331423 | 2.76E-90 |
| LINC00299 | upregulated lncRNA | -3.810174602 | 8.43E-90 |
| CTC-327F10.4 | upregulated lncRNA | -7.333903674 | 1.83E-89 |
| RP11-283G6.5 | upregulated lncRNA | -4.541064761 | 1.91E-88 |
| LINC01358 | upregulated lncRNA | -5.494743966 | 2.05E-88 |
| GATM-AS1 | upregulated lncRNA | -3.610742673 | 3.00E-86 |
| CTC-327F10.5 | upregulated lncRNA | -7.763039015 | 3.93E-86 |
| RP11-554I8.2 | upregulated lncRNA | -3.478553204 | 1.37E-85 |
| RP11-340F14.6 | upregulated lncRNA | -3.000450605 | 6.74E-85 |
| AC005264.2 | upregulated lncRNA | -3.71407173 | 8.15E-85 |
| RP11-536K7.3 | upregulated lncRNA | -2.099703156 | 1.39E-84 |
| MIAT | upregulated lncRNA | -4.183875438 | 1.39E-84 |
| CTD-2020K17.1 | upregulated lncRNA | -3.598447155 | 1.60E-84 |
| RP11-789C17.1 | upregulated lncRNA | -2.551561694 | 4.43E-84 |
| AC073115.7 | upregulated lncRNA | -5.542880629 | 7.48E-84 |
| CTD-3128G10.7 | upregulated lncRNA | -4.754805283 | 2.52E-83 |
| LINC00475 | upregulated lncRNA | -3.947309318 | 1.78E-82 |
| AC109826.1 | upregulated lncRNA | -2.727346069 | 1.90E-82 |
| L3MBTL4-AS1 | upregulated lncRNA | -2.705491443 | 1.10E-81 |
| RP11-123K3.9 | upregulated lncRNA | -5.296823862 | 3.47E-81 |
| GACAT2 | upregulated lncRNA | -5.006252452 | 9.09E-81 |
| RP1-232P20.1 | upregulated lncRNA | -3.567967271 | 2.94E-80 |
| RP11-177H13.2 | upregulated lncRNA | -2.033299252 | 3.06E-80 |
| RP11-118K6.3 | upregulated lncRNA | -2.091352083 | 4.13E-80 |
| RP11-211G23.2 | upregulated lncRNA | -6.87282554 | 8.05E-80 |
| LINC00528 | upregulated lncRNA | -2.141589626 | 1.68E-79 |
| LINC00487 | upregulated lncRNA | -3.770266711 | 2.25E-79 |
| RP11-121A14.2 | upregulated lncRNA | -2.888898003 | 8.35E-79 |
| LINC01428 | upregulated lncRNA | -3.865003495 | 1.19E-78 |
| RP11-19D2.2 | upregulated lncRNA | -5.641539211 | 1.86E-78 |
| RP11-341G23.4 | upregulated lncRNA | -3.65789831 | 3.61E-78 |
| TRG-AS1 | upregulated lncRNA | -2.444500209 | 4.74E-78 |
| PCED1B-AS1 | upregulated lncRNA | -2.485778824 | 3.34E-77 |
| RP11-309M7.1 | upregulated lncRNA | -4.415128616 | 7.55E-77 |
| LINC00944 | upregulated lncRNA | -3.645962157 | 8.16E-77 |
| RP11-181E10.3 | upregulated lncRNA | -3.346258944 | 1.02E-76 |
| FAM13A-AS1 | upregulated lncRNA | -2.2358131 | 1.94E-76 |
| RP5-940J5.3 | upregulated lncRNA | -2.514484591 | 4.51E-76 |
| LINC01094 | upregulated lncRNA | -2.654627961 | 9.02E-76 |
| RP11-543D5.1 | upregulated lncRNA | -3.679734691 | 8.94E-75 |
| MIR155HG | upregulated lncRNA | -3.080856132 | 9.06E-75 |
| CTC-523E23.5 | upregulated lncRNA | -2.244812938 | 1.07E-74 |
| DPP9-AS1 | upregulated lncRNA | -2.905752996 | 1.36E-74 |
| RP5-858L17.1 | upregulated lncRNA | -2.623627523 | 3.55E-74 |
| AC068858.1 | upregulated lncRNA | -5.879026164 | 2.27E-72 |
| LINC01235 | upregulated lncRNA | -3.718987912 | 3.91E-72 |
| RP11-680A11.5 | upregulated lncRNA | -2.045554678 | 4.30E-72 |
| RP11-274H2.5 | upregulated lncRNA | -2.707211174 | 5.69E-72 |
| RP11-284F21.7 | upregulated lncRNA | -2.724890157 | 7.83E-72 |
| AC073218.3 | upregulated lncRNA | -2.976527703 | 4.14E-71 |
| RP11-336A10.5 | upregulated lncRNA | -2.98472056 | 1.83E-70 |
| RP11-489O18.1 | upregulated lncRNA | -3.15368241 | 7.70E-70 |
| RP5-1120P11.3 | upregulated lncRNA | -4.580648422 | 1.13E-69 |
| YEATS2-AS1 | upregulated lncRNA | -2.237190562 | 1.27E-69 |
| RP11-196G11.2 | upregulated lncRNA | -2.904023805 | 2.65E-69 |
| LINC00943 | upregulated lncRNA | -3.727261291 | 4.20E-69 |
| RP1-60O19.2 | upregulated lncRNA | -4.856565063 | 7.19E-69 |
| RP11-496I9.1 | upregulated lncRNA | -3.383368327 | 7.80E-69 |
| RP13-297E16.4 | upregulated lncRNA | -4.225604251 | 8.87E-69 |
| RP11-212I21.2 | upregulated lncRNA | -4.483195232 | 3.93E-68 |
| LINC00342 | upregulated lncRNA | -2.115725379 | 9.21E-68 |
| RP11-124N14.3 | upregulated lncRNA | -2.519371971 | 1.15E-67 |
| AC156455.1 | upregulated lncRNA | -2.803728201 | 1.83E-67 |
| RP11-361L15.4 | upregulated lncRNA | -4.600857332 | 7.99E-67 |
| RP11-1334A24.6 | upregulated lncRNA | -2.353294485 | 1.25E-66 |
| AC011899.9 | upregulated lncRNA | -2.594886489 | 8.94E-66 |
| FTO-IT1 | upregulated lncRNA | -2.365212873 | 9.41E-66 |
| C20orf197 | upregulated lncRNA | -2.474811015 | 2.06E-65 |
| RP11-356J5.12 | upregulated lncRNA | -2.417535054 | 2.82E-65 |
| XXbac-B461K10.4 | upregulated lncRNA | -2.522585086 | 4.83E-65 |
| AC069363.1 | upregulated lncRNA | -4.580534139 | 5.55E-65 |
| PRR7-AS1 | upregulated lncRNA | -2.954564066 | 6.02E-65 |
| KB-1460A1.1 | upregulated lncRNA | -3.226819056 | 1.16E-64 |
| RP11-167N4.2 | upregulated lncRNA | -4.732023782 | 3.50E-64 |
| AC015977.6 | upregulated lncRNA | -5.560169996 | 4.01E-64 |
| RP11-659P15.1 | upregulated lncRNA | -3.818954152 | 4.85E-64 |
| LINC00861 | upregulated lncRNA | -2.615214319 | 1.31E-63 |
| AC073257.2 | upregulated lncRNA | -3.160747607 | 1.44E-63 |
| C5orf58 | upregulated lncRNA | -2.732040846 | 2.84E-63 |
| AC147651.4 | upregulated lncRNA | -2.302148188 | 8.77E-63 |
| LINC00173 | upregulated lncRNA | -2.949513465 | 2.32E-62 |
| PSORS1C3 | upregulated lncRNA | -2.920804278 | 7.50E-62 |
| RP11-365O16.6 | upregulated lncRNA | -2.359364976 | 8.02E-62 |
| RP11-567G11.1 | upregulated lncRNA | -3.413365178 | 8.66E-62 |
| RP5-1112D6.4 | upregulated lncRNA | -2.541739102 | 2.31E-61 |
| AC009501.4 | upregulated lncRNA | -2.135196998 | 2.60E-61 |
| RP11-299G20.2 | upregulated lncRNA | -2.749335305 | 6.65E-61 |
| LINC01268 | upregulated lncRNA | -2.153245028 | 1.08E-60 |
| RP3-477O4.14 | upregulated lncRNA | -2.243854556 | 1.36E-60 |
| RP11-1151B14.3 | upregulated lncRNA | -2.737093118 | 1.40E-60 |
| LINC01615 | upregulated lncRNA | -3.817466925 | 1.48E-60 |
| RP11-626H12.2 | upregulated lncRNA | -3.714057695 | 2.41E-60 |
| AC005785.2 | upregulated lncRNA | -2.105117225 | 4.11E-60 |
| RP11-474B16.1 | upregulated lncRNA | -2.988192189 | 6.17E-60 |
| RP11-64B16.4 | upregulated lncRNA | -3.303909105 | 7.17E-60 |
| AC019117.1 | upregulated lncRNA | -5.196016293 | 9.10E-60 |
| RP1-288H2.2 | upregulated lncRNA | -2.590116937 | 1.59E-59 |
| DLEU7-AS1 | upregulated lncRNA | -3.591254493 | 1.63E-59 |
| RP11-142A23.1 | upregulated lncRNA | -4.131956326 | 1.72E-59 |
| RP11-284F21.9 | upregulated lncRNA | -2.867396974 | 2.75E-59 |
| RP5-899E9.1 | upregulated lncRNA | -2.222615595 | 3.68E-59 |
| RP11-1094M14.5 | upregulated lncRNA | -2.743242342 | 5.24E-59 |
| RP5-1028K7.2 | upregulated lncRNA | -3.083336147 | 5.92E-59 |
| CTD-2540F13.2 | upregulated lncRNA | -2.128691342 | 2.48E-58 |
| XXbac-BPG299F13.14 | upregulated lncRNA | -2.763410869 | 4.56E-58 |
| RP11-404O13.1 | upregulated lncRNA | -3.140104058 | 5.03E-58 |
| RP5-1171I10.5 | upregulated lncRNA | -3.085501286 | 6.07E-58 |
| RP11-674N23.4 | upregulated lncRNA | -4.17564867 | 1.19E-57 |
| RP11-284F21.10 | upregulated lncRNA | -2.505304714 | 1.83E-57 |
| KCNMA1-AS1 | upregulated lncRNA | -4.009467851 | 1.98E-57 |
| RP11-45A17.4 | upregulated lncRNA | -2.642309624 | 5.23E-57 |
| RP11-1151B14.4 | upregulated lncRNA | -3.082004104 | 6.61E-57 |
| AC108463.1 | upregulated lncRNA | -2.052109725 | 1.14E-56 |
| AC137932.6 | upregulated lncRNA | -2.2500306 | 2.31E-56 |
| AC084117.3 | upregulated lncRNA | -2.96961805 | 4.31E-56 |
| LINC00158 | upregulated lncRNA | -4.258161176 | 7.03E-56 |
| RP11-44K6.4 | upregulated lncRNA | -5.26551891 | 8.22E-56 |
| RP1-142L7.5 | upregulated lncRNA | -3.02880262 | 1.51E-55 |
| CTD-2527I21.14 | upregulated lncRNA | -2.747458774 | 1.74E-55 |
| ITGB2-AS1 | upregulated lncRNA | -2.618119272 | 2.19E-55 |
| CTD-2035E11.5 | upregulated lncRNA | -2.298597404 | 2.76E-55 |
| RP4-647J21.1 | upregulated lncRNA | -2.747266358 | 3.43E-55 |
| LINC01480 | upregulated lncRNA | -2.460907158 | 3.88E-55 |
| RP11-525K10.3 | upregulated lncRNA | -2.303049995 | 7.33E-55 |
| LA16c-390H2.4 | upregulated lncRNA | -2.683927177 | 5.22E-54 |
| RP11-326C3.2 | upregulated lncRNA | -3.706193635 | 1.08E-53 |
| RP11-283G6.4 | upregulated lncRNA | -2.721017684 | 1.56E-53 |
| AC027601.1 | upregulated lncRNA | -2.148524428 | 1.58E-53 |
| RP11-302L19.3 | upregulated lncRNA | -2.557792142 | 1.79E-53 |
| CTB-26E19.1 | upregulated lncRNA | -4.566049375 | 2.35E-53 |
| RP13-714J12.1 | upregulated lncRNA | -4.104218805 | 2.46E-53 |
| RP3-393E18.2 | upregulated lncRNA | -3.115776967 | 2.94E-53 |
| ELDR | upregulated lncRNA | -4.940311875 | 3.81E-53 |
| RP1-167A14.2 | upregulated lncRNA | -2.55248947 | 7.58E-53 |
| RP11-455F5.5 | upregulated lncRNA | -2.921974432 | 1.90E-52 |
| RP11-121A8.1 | upregulated lncRNA | -2.12286803 | 2.49E-52 |
| RP11-480A16.1 | upregulated lncRNA | -2.29021512 | 2.99E-52 |
| RP11-64D24.4 | upregulated lncRNA | -3.705496989 | 3.00E-52 |
| RP11-258F1.1 | upregulated lncRNA | -2.221731911 | 3.86E-52 |
| CTA-228A9.3 | upregulated lncRNA | -2.419889704 | 4.26E-52 |
| LINC00460 | upregulated lncRNA | -6.049750469 | 9.66E-52 |
| LINC01272 | upregulated lncRNA | -2.49028157 | 1.15E-51 |
| C9orf139 | upregulated lncRNA | -2.061237767 | 1.48E-51 |
| RP4-671O14.7 | upregulated lncRNA | -3.128553225 | 1.56E-51 |
| AC159540.1 | upregulated lncRNA | -2.481922831 | 1.88E-51 |
| RP11-70D24.3 | upregulated lncRNA | -2.725031397 | 2.18E-51 |
| SEMA6A-AS1 | upregulated lncRNA | -2.033828375 | 3.72E-51 |
| CTC-251D13.1 | upregulated lncRNA | -2.118205739 | 4.30E-51 |
| RP11-807H17.1 | upregulated lncRNA | -3.111589352 | 4.36E-51 |
| RP11-861E21.2 | upregulated lncRNA | -2.40753793 | 4.54E-51 |
| RP11-557C18.4 | upregulated lncRNA | -4.934597825 | 1.05E-50 |
| RP11-143J12.3 | upregulated lncRNA | -2.155344249 | 2.39E-50 |
| RP11-488C13.6 | upregulated lncRNA | -2.336544121 | 5.02E-50 |
| RP11-38J22.3 | upregulated lncRNA | -2.952910034 | 5.10E-50 |
| HM13-IT1 | upregulated lncRNA | -2.107646393 | 6.14E-50 |
| LINC01234 | upregulated lncRNA | -6.060159611 | 1.26E-49 |
| RP11-12M5.3 | upregulated lncRNA | -3.305350788 | 1.31E-49 |
| RP11-255G12.3 | upregulated lncRNA | -4.611491926 | 1.98E-49 |
| LINC00426 | upregulated lncRNA | -2.389793157 | 5.26E-49 |
| RP11-1018N14.5 | upregulated lncRNA | -3.217478326 | 8.21E-49 |
| LINC00881 | upregulated lncRNA | -3.460007428 | 9.92E-49 |
| RP5-1091N2.9 | upregulated lncRNA | -2.115765674 | 1.68E-48 |
| RP11-352G9.1 | upregulated lncRNA | -3.048401076 | 1.86E-48 |
| LINC01152 | upregulated lncRNA | -2.787082363 | 2.35E-48 |
| LAMA5-AS1 | upregulated lncRNA | -2.741215092 | 2.83E-48 |
| RP11-112L6.3 | upregulated lncRNA | -2.548874134 | 3.82E-48 |
| CFLAR-AS1 | upregulated lncRNA | -2.347179006 | 6.47E-48 |
| RP11-1069G10.1 | upregulated lncRNA | -2.110143135 | 1.02E-47 |
| RP11-496D24.2 | upregulated lncRNA | -4.247315399 | 1.38E-47 |
| RP6-91H8.3 | upregulated lncRNA | -3.949344847 | 1.91E-47 |
| CTB-39G8.3 | upregulated lncRNA | -2.009474602 | 2.15E-47 |
| AC012613.2 | upregulated lncRNA | -4.242545529 | 3.12E-47 |
| CTB-127C13.1 | upregulated lncRNA | -4.603956916 | 3.64E-47 |
| SIRPG-AS1 | upregulated lncRNA | -4.763050996 | 5.49E-47 |
| RP11-452C13.1 | upregulated lncRNA | -2.837367581 | 7.60E-47 |
| RP11-598F7.3 | upregulated lncRNA | -3.713297764 | 8.68E-47 |
| RP11-264B17.3 | upregulated lncRNA | -2.060175051 | 9.83E-47 |
| RP11-865I6.2 | upregulated lncRNA | -4.916136176 | 1.03E-46 |
| ENO1-IT1 | upregulated lncRNA | -2.257583578 | 1.16E-46 |
| RP5-884M6.1 | upregulated lncRNA | -7.169198123 | 1.23E-46 |
| RP11-395I14.2 | upregulated lncRNA | -2.067243415 | 1.29E-46 |
| CTA-384D8.35 | upregulated lncRNA | -2.458915471 | 1.52E-46 |
| RP11-145H9.3 | upregulated lncRNA | -2.786989999 | 1.93E-46 |
| RP11-291B21.2 | upregulated lncRNA | -3.25714887 | 2.52E-46 |
| FLJ35934 | upregulated lncRNA | -2.750117323 | 2.73E-46 |
| RP4-753P9.3 | upregulated lncRNA | -2.407664923 | 2.85E-46 |
| CTD-2228K2.7 | upregulated lncRNA | -2.798661633 | 7.75E-46 |
| AC008746.12 | upregulated lncRNA | -2.243722737 | 1.05E-45 |
| RP11-359E10.1 | upregulated lncRNA | -2.325287809 | 1.05E-45 |
| TNK2-AS1 | upregulated lncRNA | -2.172195734 | 1.07E-45 |
| DUXAP8 | upregulated lncRNA | -2.645891244 | 1.91E-45 |
| OR7E47P | upregulated lncRNA | -2.790635955 | 2.21E-45 |
| PAQR9-AS1 | upregulated lncRNA | -4.204384284 | 2.67E-45 |
| RP11-465L10.10 | upregulated lncRNA | -3.333271876 | 2.67E-45 |
| RP11-327F22.2 | upregulated lncRNA | -2.207974956 | 2.82E-45 |
| RP13-516M14.10 | upregulated lncRNA | -2.21493798 | 2.92E-45 |
| CTC-542B22.2 | upregulated lncRNA | -2.214929239 | 3.17E-45 |
| RP11-785G17.1 | upregulated lncRNA | -3.271481684 | 4.92E-45 |
| LINC01271 | upregulated lncRNA | -2.883014426 | 5.49E-45 |
| CTD-2036P10.6 | upregulated lncRNA | -2.119947831 | 7.10E-45 |
| USP30-AS1 | upregulated lncRNA | -2.272736042 | 1.04E-44 |
| AC092580.4 | upregulated lncRNA | -2.285131847 | 1.08E-44 |
| RP3-522D1.1 | upregulated lncRNA | -3.214283819 | 1.61E-44 |
| CTD-2171N6.1 | upregulated lncRNA | -3.374058269 | 3.46E-44 |
| LINC01150 | upregulated lncRNA | -2.228766363 | 3.55E-44 |
| CTD-2616J11.3 | upregulated lncRNA | -3.830672189 | 3.98E-44 |
| RP4-665J23.1 | upregulated lncRNA | -2.151101403 | 6.64E-44 |
| CTD-2313F11.1 | upregulated lncRNA | -3.471508773 | 7.70E-44 |
| RP11-489G11.3 | upregulated lncRNA | -3.661297988 | 1.27E-43 |
| RP11-837J7.4 | upregulated lncRNA | -2.481631482 | 4.99E-43 |
| RP5-1172A22.1 | upregulated lncRNA | -4.011952779 | 5.03E-43 |
| LINC01546 | upregulated lncRNA | -3.015872799 | 5.44E-43 |
| AC145110.1 | upregulated lncRNA | -3.980815293 | 6.31E-43 |
| RP11-67C2.2 | upregulated lncRNA | -2.718377566 | 1.08E-42 |
| AC092667.2 | upregulated lncRNA | -2.549609072 | 1.78E-42 |
| RP11-222K16.2 | upregulated lncRNA | -2.305254625 | 1.94E-42 |
| CTD-3195I5.3 | upregulated lncRNA | -2.226021766 | 2.30E-42 |
| RP11-674P19.2 | upregulated lncRNA | -3.408500509 | 3.51E-42 |
| AC005387.2 | upregulated lncRNA | -2.424119041 | 3.75E-42 |
| RP11-572O17.1 | upregulated lncRNA | -2.365974777 | 5.47E-42 |
| RP11-21K12.3 | upregulated lncRNA | -2.026721213 | 1.19E-41 |
| LINC00707 | upregulated lncRNA | -2.737416064 | 1.20E-41 |
| RP11-395B7.2 | upregulated lncRNA | -4.008910215 | 1.43E-41 |
| AC007278.3 | upregulated lncRNA | -2.815705697 | 1.51E-41 |
| RP11-506B6.6 | upregulated lncRNA | -2.166185291 | 2.26E-41 |
| AC000067.1 | upregulated lncRNA | -3.224951186 | 2.32E-41 |
| TMEM92-AS1 | upregulated lncRNA | -3.45289548 | 2.35E-41 |
| HLA-DQB1-AS1 | upregulated lncRNA | -2.094460958 | 3.71E-41 |
| LINC01146 | upregulated lncRNA | -2.214830337 | 9.20E-41 |
| CTD-2201E18.5 | upregulated lncRNA | -2.473311939 | 9.48E-41 |
| RP11-54A4.2 | upregulated lncRNA | -2.327732359 | 1.51E-40 |
| RP11-44N11.2 | upregulated lncRNA | -2.3848675 | 1.57E-40 |
| AF064858.8 | upregulated lncRNA | -2.747237331 | 1.65E-40 |
| ADAM20P1 | upregulated lncRNA | -2.015341442 | 1.71E-40 |
| RP11-24F11.2 | upregulated lncRNA | -2.053854389 | 1.90E-40 |
| RP11-568J23.8 | upregulated lncRNA | -3.92511943 | 1.90E-40 |
| C15orf54 | upregulated lncRNA | -2.716102616 | 2.93E-40 |
| RP11-121A14.3 | upregulated lncRNA | -3.860710814 | 3.08E-40 |
| AC017002.1 | upregulated lncRNA | -2.187114185 | 3.43E-40 |
| GRPEL2-AS1 | upregulated lncRNA | -2.760773497 | 3.91E-40 |
| RP11-556E13.1 | upregulated lncRNA | -3.220911105 | 4.04E-40 |
| RP11-551L14.4 | upregulated lncRNA | -3.196221257 | 6.19E-40 |
| RP1-29C18.8 | upregulated lncRNA | -2.90041907 | 7.00E-40 |
| LINC01012 | upregulated lncRNA | -2.109211922 | 8.51E-40 |
| RP4-539M6.22 | upregulated lncRNA | -3.105510508 | 1.00E-39 |
| RP11-1151B14.2 | upregulated lncRNA | -4.291240091 | 1.09E-39 |
| AFAP1-AS1 | upregulated lncRNA | -3.508439208 | 1.17E-39 |
| AC079767.4 | upregulated lncRNA | -2.846343454 | 1.28E-39 |
| RP11-439C15.4 | upregulated lncRNA | -2.239506993 | 1.50E-39 |
| AP002954.4 | upregulated lncRNA | -2.844418763 | 1.60E-39 |
| AC068492.1 | upregulated lncRNA | -4.357476018 | 1.81E-39 |
| RP11-343J3.2 | upregulated lncRNA | -3.412215071 | 1.87E-39 |
| CTB-140J7.2 | upregulated lncRNA | -3.127291381 | 2.01E-39 |
| AC006369.2 | upregulated lncRNA | -2.378944498 | 2.13E-39 |
| CTD-3064M3.7 | upregulated lncRNA | -2.69782437 | 2.18E-39 |
| PP12613 | upregulated lncRNA | -4.714812098 | 3.20E-39 |
| CTD-2537I9.12 | upregulated lncRNA | -2.07990418 | 3.31E-39 |
| LINC00704 | upregulated lncRNA | -3.430750813 | 3.98E-39 |
| RP11-497H17.1 | upregulated lncRNA | -2.38433322 | 5.36E-39 |
| ARAP1-AS2 | upregulated lncRNA | -2.520093227 | 6.40E-39 |
| RP11-1151B14.5 | upregulated lncRNA | -3.191929135 | 6.47E-39 |
| RP11-102G14.1 | upregulated lncRNA | -2.627285133 | 7.72E-39 |
| LINC01163 | upregulated lncRNA | -4.362879981 | 9.08E-39 |
| LL22NC03-N14H11.1 | upregulated lncRNA | -3.22360155 | 1.16E-38 |
| AC004988.1 | upregulated lncRNA | -3.455994077 | 1.46E-38 |
| ASAP1-IT2 | upregulated lncRNA | -2.645978441 | 1.60E-38 |
| AC012593.1 | upregulated lncRNA | -3.261978283 | 1.81E-38 |
| AC124944.3 | upregulated lncRNA | -2.802308804 | 1.90E-38 |
| AC067945.4 | upregulated lncRNA | -2.505234996 | 1.91E-38 |
| AP000476.1 | upregulated lncRNA | -2.262007354 | 2.05E-38 |
| RP11-20I20.4 | upregulated lncRNA | -2.530731281 | 2.87E-38 |
| CTD-2278I10.1 | upregulated lncRNA | -3.316893314 | 3.40E-38 |
| LINC00607 | upregulated lncRNA | -2.088714872 | 4.83E-38 |
| RP11-44K6.2 | upregulated lncRNA | -4.35918411 | 4.85E-38 |
| RP11-322D14.2 | upregulated lncRNA | -3.701714528 | 5.01E-38 |
| ASMTL-AS1 | upregulated lncRNA | -2.39786371 | 5.18E-38 |
| LINC00264 | upregulated lncRNA | -3.688401497 | 5.26E-38 |
| RP11-523O18.5 | upregulated lncRNA | -2.369948923 | 6.77E-38 |
| RP11-1149M10.2 | upregulated lncRNA | -2.863645324 | 1.06E-37 |
| RP11-212I21.5 | upregulated lncRNA | -4.332355537 | 1.08E-37 |
| RP11-643C9.2 | upregulated lncRNA | -3.390951044 | 1.13E-37 |
| RP11-184A2.3 | upregulated lncRNA | -3.990212794 | 1.22E-37 |
| RP11-701H16.4 | upregulated lncRNA | -2.782606736 | 1.29E-37 |
| RP11-332J15.3 | upregulated lncRNA | -3.059477275 | 1.37E-37 |
| RP5-988G17.1 | upregulated lncRNA | -3.978174386 | 1.92E-37 |
| RP11-414H23.3 | upregulated lncRNA | -4.849874235 | 2.66E-37 |
| ABHD11-AS1 | upregulated lncRNA | -2.315235478 | 3.18E-37 |
| CTD-2357A8.3 | upregulated lncRNA | -3.72007989 | 4.93E-37 |
| RP11-10J5.1 | upregulated lncRNA | -4.435517219 | 5.64E-37 |
| LINC01033 | upregulated lncRNA | -3.22049527 | 6.12E-37 |
| CTD-2023M8.1 | upregulated lncRNA | -3.553674516 | 6.56E-37 |
| CTD-2553C6.1 | upregulated lncRNA | -3.690678336 | 6.71E-37 |
| PCAT29 | upregulated lncRNA | -2.448468484 | 8.48E-37 |
| CASC11 | upregulated lncRNA | -2.382551749 | 1.06E-36 |
| AC005306.3 | upregulated lncRNA | -2.27951737 | 1.55E-36 |
| GAPLINC | upregulated lncRNA | -2.11045947 | 1.71E-36 |
| RP11-663P9.1 | upregulated lncRNA | -3.340147597 | 1.99E-36 |
| NRIR | upregulated lncRNA | -2.206013683 | 2.16E-36 |
| EGLN3-AS1 | upregulated lncRNA | -4.247112928 | 2.29E-36 |
| AC108676.1 | upregulated lncRNA | -2.212200341 | 2.68E-36 |
| RP11-274H2.3 | upregulated lncRNA | -2.29540182 | 3.07E-36 |
| RP11-1259L22.1 | upregulated lncRNA | -4.335986466 | 3.16E-36 |
| RP5-902P8.10 | upregulated lncRNA | -2.089072281 | 3.34E-36 |
| AC006272.2 | upregulated lncRNA | -2.626385018 | 3.38E-36 |
| RP11-1151B14.1 | upregulated lncRNA | -4.315438336 | 3.97E-36 |
| RP11-70D24.4 | upregulated lncRNA | -3.444537276 | 5.55E-36 |
| RP11-510C10.3 | upregulated lncRNA | -5.614996588 | 5.92E-36 |
| RP11-405M12.3 | upregulated lncRNA | -2.191043292 | 6.04E-36 |
| CTD-2527I21.9 | upregulated lncRNA | -3.350250932 | 6.84E-36 |
| RP11-84D1.1 | upregulated lncRNA | -3.470132995 | 7.92E-36 |
| RP11-274H2.2 | upregulated lncRNA | -2.311857728 | 8.71E-36 |
| CTD-2537I9.13 | upregulated lncRNA | -2.302755793 | 1.01E-35 |
| AF064858.11 | upregulated lncRNA | -3.031927769 | 1.07E-35 |
| RP11-147L13.2 | upregulated lncRNA | -2.117008748 | 1.86E-35 |
| AC002059.10 | upregulated lncRNA | -2.284353501 | 2.04E-35 |
| RP11-497G19.7 | upregulated lncRNA | -3.555372974 | 2.12E-35 |
| AC010091.1 | upregulated lncRNA | -3.502043599 | 2.42E-35 |
| AP003774.1 | upregulated lncRNA | -2.411738643 | 2.68E-35 |
| RP11-331K21.1 | upregulated lncRNA | -4.098662539 | 2.78E-35 |
| RP13-39P12.3 | upregulated lncRNA | -2.1700783 | 3.12E-35 |
| RP11-244M2.1 | upregulated lncRNA | -2.239452298 | 3.69E-35 |
| RP11-359G22.2 | upregulated lncRNA | -2.129461884 | 3.74E-35 |
| AP000797.4 | upregulated lncRNA | -4.706692801 | 4.60E-35 |
| AC104820.2 | upregulated lncRNA | -2.54901348 | 5.12E-35 |
| RP11-81H14.1 | upregulated lncRNA | -2.565441212 | 5.25E-35 |
| CTB-91J4.1 | upregulated lncRNA | -4.061194911 | 5.62E-35 |
| RP3-434P1.6 | upregulated lncRNA | -3.143752287 | 5.73E-35 |
| RP11-348J24.2 | upregulated lncRNA | -2.9974545 | 6.11E-35 |
| RP11-472N13.2 | upregulated lncRNA | -2.546959317 | 6.11E-35 |
| WI2-87327B8.2 | upregulated lncRNA | -2.017330892 | 7.16E-35 |
| RP11-753H16.5 | upregulated lncRNA | -2.633570948 | 7.70E-35 |
| RP11-267N12.1 | upregulated lncRNA | -2.677467614 | 8.40E-35 |
| RP11-549L6.3 | upregulated lncRNA | -3.654414057 | 8.77E-35 |
| RP11-66A2.2 | upregulated lncRNA | -3.776891409 | 9.62E-35 |
| RP11-982M15.8 | upregulated lncRNA | -2.824990863 | 1.58E-34 |
| LINC01330 | upregulated lncRNA | -2.367236272 | 1.83E-34 |
| RP11-91P24.6 | upregulated lncRNA | -2.06354767 | 1.95E-34 |
| AC015849.16 | upregulated lncRNA | -2.308908244 | 2.02E-34 |
| RP11-261P9.4 | upregulated lncRNA | -2.278972665 | 2.41E-34 |
| AC093642.3 | upregulated lncRNA | -2.049306711 | 2.45E-34 |
| RP11-231E4.5 | upregulated lncRNA | -2.224420154 | 2.46E-34 |
| AP004372.1 | upregulated lncRNA | -4.986088238 | 2.68E-34 |
| AP001055.6 | upregulated lncRNA | -2.251376324 | 2.79E-34 |
| CTB-113P19.1 | upregulated lncRNA | -2.081036401 | 3.62E-34 |
| RP3-455J7.4 | upregulated lncRNA | -2.436072963 | 4.10E-34 |
| LA16c-361A3.3 | upregulated lncRNA | -2.006900133 | 4.33E-34 |
| RP11-84C10.4 | upregulated lncRNA | -2.159706892 | 5.25E-34 |
| RP11-264E20.1 | upregulated lncRNA | -2.675252993 | 5.28E-34 |
| CTD-2571L23.8 | upregulated lncRNA | -2.569610329 | 5.77E-34 |
| AC010729.1 | upregulated lncRNA | -4.406260097 | 6.61E-34 |
| RP11-455F5.6 | upregulated lncRNA | -2.192756286 | 7.19E-34 |
| RP11-395B7.4 | upregulated lncRNA | -2.560977698 | 7.92E-34 |
| AC131056.3 | upregulated lncRNA | -3.085181385 | 8.32E-34 |
| CTC-806A22.1 | upregulated lncRNA | -3.555008441 | 1.16E-33 |
| RP11-513G11.4 | upregulated lncRNA | -2.398462262 | 1.23E-33 |
| SERPINB9P1 | upregulated lncRNA | -2.020643121 | 1.25E-33 |
| RP11-264E20.2 | upregulated lncRNA | -3.025836043 | 1.28E-33 |
| RP11-426C22.4 | upregulated lncRNA | -2.352667951 | 1.41E-33 |
| INHBA-AS1 | upregulated lncRNA | -2.814620636 | 1.44E-33 |
| RP4-644L1.2 | upregulated lncRNA | -2.956326796 | 2.09E-33 |
| RP11-61I13.3 | upregulated lncRNA | -2.438527166 | 2.53E-33 |
| AC006262.4 | upregulated lncRNA | -4.993062411 | 3.17E-33 |
| C2-AS1 | upregulated lncRNA | -4.020671332 | 3.39E-33 |
| RP11-678G15.2 | upregulated lncRNA | -2.57510489 | 3.46E-33 |
| LINC01614 | upregulated lncRNA | -2.69290043 | 4.35E-33 |
| RP11-483L5.1 | upregulated lncRNA | -2.231229312 | 5.34E-33 |
| RP1-244F24.1 | upregulated lncRNA | -2.2769011 | 5.91E-33 |
| RP1-142L7.9 | upregulated lncRNA | -3.187384176 | 6.04E-33 |
| RP11-27G22.1 | upregulated lncRNA | -4.95168317 | 6.98E-33 |
| PLA2G4C-AS1 | upregulated lncRNA | -2.343971183 | 8.06E-33 |
| RP11-2L8.1 | upregulated lncRNA | -2.770032567 | 1.03E-32 |
| RP11-57A1.1 | upregulated lncRNA | -2.738314628 | 1.84E-32 |
| LINC01141 | upregulated lncRNA | -2.215998977 | 1.88E-32 |
| XXbac-B476C20.13 | upregulated lncRNA | -3.115342973 | 2.07E-32 |
| RP11-521I2.3 | upregulated lncRNA | -2.009990395 | 2.13E-32 |
| RP11-218E20.5 | upregulated lncRNA | -3.409485733 | 2.79E-32 |
| RP11-192P3.4 | upregulated lncRNA | -2.457293157 | 3.12E-32 |
| RP5-968J1.1 | upregulated lncRNA | -2.417793606 | 3.54E-32 |
| AC130469.2 | upregulated lncRNA | -2.907453474 | 4.97E-32 |
| AF127936.5 | upregulated lncRNA | -2.192331419 | 6.19E-32 |
| KCCAT211 | upregulated lncRNA | -2.509225572 | 9.84E-32 |
| LINC00664 | upregulated lncRNA | -2.390029399 | 1.08E-31 |
| LINC01265 | upregulated lncRNA | -2.581039636 | 1.35E-31 |
| RP11-1399P15.1 | upregulated lncRNA | -2.96640134 | 1.68E-31 |
| AC091177.1 | upregulated lncRNA | -2.490560759 | 2.01E-31 |
| RP11-14C10.3 | upregulated lncRNA | -3.845652333 | 2.13E-31 |
| RP11-903H12.3 | upregulated lncRNA | -2.141535638 | 2.59E-31 |
| C20orf203 | upregulated lncRNA | -2.645349173 | 2.70E-31 |
| RP4-545L17.12 | upregulated lncRNA | -2.918808449 | 3.01E-31 |
| TRPM2-AS | upregulated lncRNA | -2.02816811 | 3.58E-31 |
| RP11-184A2.2 | upregulated lncRNA | -3.673696114 | 4.14E-31 |
| RP11-290F5.1 | upregulated lncRNA | -2.712537432 | 5.25E-31 |
| LINC01260 | upregulated lncRNA | -2.313579063 | 5.35E-31 |
| DKFZp434J0226 | upregulated lncRNA | -3.30280244 | 5.77E-31 |
| RP11-319E12.2 | upregulated lncRNA | -3.052275123 | 7.48E-31 |
| RP11-1191J2.2 | upregulated lncRNA | -3.022638544 | 9.38E-31 |
| RP11-42O4.2 | upregulated lncRNA | -2.626983133 | 9.93E-31 |
| RP5-902P8.12 | upregulated lncRNA | -2.008309796 | 1.02E-30 |
| KB-1836B5.1 | upregulated lncRNA | -2.018629304 | 1.05E-30 |
| SH3PXD2A-AS1 | upregulated lncRNA | -2.121782439 | 1.05E-30 |
| RP11-528G1.2 | upregulated lncRNA | -3.306815332 | 1.19E-30 |
| AC015933.2 | upregulated lncRNA | -2.414126825 | 1.24E-30 |
| RP11-145M4.2 | upregulated lncRNA | -3.607386887 | 1.38E-30 |
| XX-C00717C00720L.1 | upregulated lncRNA | -2.355365503 | 1.59E-30 |
| RP11-118M9.3 | upregulated lncRNA | -2.300267965 | 1.66E-30 |
| U52111.14 | upregulated lncRNA | -2.123335967 | 2.21E-30 |
| AC007461.2 | upregulated lncRNA | -2.107942613 | 2.23E-30 |
| RP11-388C12.5 | upregulated lncRNA | -2.689431613 | 2.31E-30 |
| IL21-AS1 | upregulated lncRNA | -4.17690783 | 2.60E-30 |
| PIK3CD-AS1 | upregulated lncRNA | -2.435332476 | 2.93E-30 |
| LINC00160 | upregulated lncRNA | -4.370396093 | 3.17E-30 |
| CTD-2223O18.1 | upregulated lncRNA | -3.085278483 | 3.64E-30 |
| RP11-264K23.1 | upregulated lncRNA | -4.135236321 | 4.19E-30 |
| RP11-247A12.7 | upregulated lncRNA | -2.826958228 | 4.95E-30 |
| RP11-120K18.2 | upregulated lncRNA | -3.740629625 | 5.30E-30 |
| RP11-932O9.10 | upregulated lncRNA | -2.582000693 | 6.84E-30 |
| RP1-105O18.1 | upregulated lncRNA | -4.478173322 | 8.21E-30 |
| RP3-340N1.2 | upregulated lncRNA | -3.306569457 | 8.94E-30 |
| RP5-943J3.1 | upregulated lncRNA | -2.950735188 | 1.00E-29 |
| CTD-2083E4.7 | upregulated lncRNA | -3.603528423 | 1.49E-29 |
| AC068196.1 | upregulated lncRNA | -2.990533225 | 1.58E-29 |
| XXbac-BPG170G13.32 | upregulated lncRNA | -2.956833855 | 1.75E-29 |
| AC067959.1 | upregulated lncRNA | -3.355395848 | 1.93E-29 |
| RP11-50B3.2 | upregulated lncRNA | -3.195465688 | 2.15E-29 |
| AC005387.3 | upregulated lncRNA | -2.179903171 | 2.46E-29 |
| RP11-510C10.2 | upregulated lncRNA | -5.120373614 | 2.74E-29 |
| AC003003.5 | upregulated lncRNA | -2.784166147 | 3.07E-29 |
| AC007750.5 | upregulated lncRNA | -2.476588036 | 3.30E-29 |
| CTD-2195B23.3 | upregulated lncRNA | -2.84658273 | 3.67E-29 |
| RP1-140K8.5 | upregulated lncRNA | -2.602579559 | 3.75E-29 |
| CTD-2252P21.1 | upregulated lncRNA | -3.195205456 | 3.84E-29 |
| CTD-2521M24.5 | upregulated lncRNA | -2.929287234 | 4.02E-29 |
| LINC00298 | upregulated lncRNA | -3.134971514 | 4.65E-29 |
| RP11-167B3.2 | upregulated lncRNA | -3.380980455 | 6.43E-29 |
| AC073130.1 | upregulated lncRNA | -2.916333783 | 8.16E-29 |
| RP11-400N13.3 | upregulated lncRNA | -5.187642811 | 8.73E-29 |
| RP11-142M10.2 | upregulated lncRNA | -3.649045512 | 1.03E-28 |
| RP1-224A6.9 | upregulated lncRNA | -2.259607968 | 1.03E-28 |
| AC107218.3 | upregulated lncRNA | -2.502039584 | 1.11E-28 |
| RP11-206M11.7 | upregulated lncRNA | -5.452708948 | 1.28E-28 |
| RP11-552D4.1 | upregulated lncRNA | -2.574838476 | 1.65E-28 |
| SLC6A1-AS1 | upregulated lncRNA | -2.326716416 | 1.71E-28 |
| C1orf147 | upregulated lncRNA | -2.17917302 | 2.36E-28 |
| RP5-940J5.8 | upregulated lncRNA | -2.305140045 | 3.10E-28 |
| AC097713.3 | upregulated lncRNA | -3.932187113 | 3.87E-28 |
| WI2-8325B5.1 | upregulated lncRNA | -4.067209133 | 4.07E-28 |
| CTA-414D7.1 | upregulated lncRNA | -2.66792097 | 5.13E-28 |
| CTC-453G23.5 | upregulated lncRNA | -2.354618435 | 5.14E-28 |
| RP11-20J15.3 | upregulated lncRNA | -2.88925347 | 5.46E-28 |
| RP11-566K11.7 | upregulated lncRNA | -2.641716175 | 5.86E-28 |
| ARHGAP26-AS1 | upregulated lncRNA | -3.196590744 | 6.83E-28 |
| SNAP25-AS1 | upregulated lncRNA | -2.234266554 | 7.00E-28 |
| RP11-327F22.1 | upregulated lncRNA | -2.848148454 | 7.72E-28 |
| RP11-290F5.2 | upregulated lncRNA | -2.254359639 | 7.89E-28 |
| GS1-600G8.5 | upregulated lncRNA | -3.425475479 | 9.47E-28 |
| RP1-278C19.8 | upregulated lncRNA | -2.38188134 | 1.11E-27 |
| RP11-227G15.12 | upregulated lncRNA | -2.051118003 | 1.12E-27 |
| LINC00544 | upregulated lncRNA | -3.339578155 | 1.19E-27 |
| RP11-284H18.1 | upregulated lncRNA | -2.934523455 | 1.22E-27 |
| RP11-476D10.1 | upregulated lncRNA | -2.436205011 | 1.68E-27 |
| AP001604.3 | upregulated lncRNA | -3.182759411 | 1.71E-27 |
| LA16c-321D4.2 | upregulated lncRNA | -2.024628866 | 1.86E-27 |
| LA16c-358B7.4 | upregulated lncRNA | -2.031019211 | 1.90E-27 |
| RP11-617F23.2 | upregulated lncRNA | -3.20432448 | 1.98E-27 |
| RP11-661C8.2 | upregulated lncRNA | -3.584801454 | 2.17E-27 |
| RP11-6O2.4 | upregulated lncRNA | -2.28712679 | 2.37E-27 |
| RP5-1092A11.5 | upregulated lncRNA | -3.775888522 | 2.76E-27 |
| RP11-540K16.1 | upregulated lncRNA | -3.255503857 | 3.35E-27 |
| AC009133.17 | upregulated lncRNA | -2.07467451 | 3.46E-27 |
| RP11-638I2.9 | upregulated lncRNA | -2.149967497 | 3.61E-27 |
| LINC00906 | upregulated lncRNA | -3.218712593 | 6.80E-27 |
| RP11-227G15.9 | upregulated lncRNA | -2.504254503 | 8.36E-27 |
| FOXC2-AS1 | upregulated lncRNA | -3.150696761 | 1.03E-26 |
| AC009495.3 | upregulated lncRNA | -2.207304369 | 1.11E-26 |
| LINC00896 | upregulated lncRNA | -3.017365761 | 1.40E-26 |
| RP1-79C4.4 | upregulated lncRNA | -2.745862084 | 1.47E-26 |
| CTC-472C24.1 | upregulated lncRNA | -4.286105067 | 1.47E-26 |
| RP11-145A3.1 | upregulated lncRNA | -3.174118887 | 1.47E-26 |
| CTD-3187F8.14 | upregulated lncRNA | -2.225867153 | 1.74E-26 |
| RP11-1026M7.2 | upregulated lncRNA | -2.726655664 | 1.84E-26 |
| RP1-45C12.1 | upregulated lncRNA | -2.020179631 | 1.93E-26 |
| AP001257.1 | upregulated lncRNA | -3.034863499 | 2.82E-26 |
| RP11-282I1.1 | upregulated lncRNA | -2.878388452 | 2.88E-26 |
| AC091814.3 | upregulated lncRNA | -2.638267089 | 3.01E-26 |
| RP11-16E12.2 | upregulated lncRNA | -2.33385908 | 3.39E-26 |
| RP5-998N21.4 | upregulated lncRNA | -2.431307711 | 3.54E-26 |
| RP11-298O21.2 | upregulated lncRNA | -2.300767169 | 3.54E-26 |
| RP11-429B14.4 | upregulated lncRNA | -4.603732604 | 3.60E-26 |
| RP11-586K2.1 | upregulated lncRNA | -5.551416023 | 3.91E-26 |
| LINC01426 | upregulated lncRNA | -2.384182763 | 4.40E-26 |
| RP5-1031D4.2 | upregulated lncRNA | -2.574206193 | 4.78E-26 |
| AC002331.1 | upregulated lncRNA | -3.539229001 | 4.92E-26 |
| AC008060.7 | upregulated lncRNA | -5.507272374 | 7.21E-26 |
| RP11-6L6.2 | upregulated lncRNA | -2.130645442 | 9.99E-26 |
| RP11-861E21.1 | upregulated lncRNA | -3.006848557 | 1.09E-25 |
| RP11-380G5.2 | upregulated lncRNA | -2.068445922 | 1.17E-25 |
| AL592528.1 | upregulated lncRNA | -4.176354131 | 1.24E-25 |
| RP11-138I17.1 | upregulated lncRNA | -3.722122613 | 1.29E-25 |
| RP11-438D8.2 | upregulated lncRNA | -2.337004814 | 1.58E-25 |
| AF064858.7 | upregulated lncRNA | -3.329729204 | 1.58E-25 |
| RERG-IT1 | upregulated lncRNA | -2.032735271 | 1.60E-25 |
| CTB-102L5.8 | upregulated lncRNA | -2.109323439 | 1.68E-25 |
| KB-1043D8.8 | upregulated lncRNA | -3.349055404 | 2.27E-25 |
| RP11-804N13.1 | upregulated lncRNA | -4.209459028 | 2.43E-25 |
| NLGN1-AS1 | upregulated lncRNA | -3.753777396 | 2.48E-25 |
| XXbac-BPG300A18.13 | upregulated lncRNA | -2.091988881 | 3.05E-25 |
| LINC00626 | upregulated lncRNA | -2.830089858 | 3.57E-25 |
| LINC01611 | upregulated lncRNA | -4.300847102 | 4.92E-25 |
| RP11-479J7.2 | upregulated lncRNA | -3.977865685 | 4.93E-25 |
| CELF2-AS1 | upregulated lncRNA | -2.218000674 | 4.96E-25 |
| AP000593.7 | upregulated lncRNA | -2.01436476 | 6.40E-25 |
| RP11-166B2.7 | upregulated lncRNA | -2.809481723 | 6.40E-25 |
| RP11-178L8.9 | upregulated lncRNA | -2.241042969 | 6.47E-25 |
| RP11-367G18.1 | upregulated lncRNA | -2.539008128 | 8.51E-25 |
| SPRY4-IT1 | upregulated lncRNA | -2.05181225 | 1.04E-24 |
| RP13-1016M1.2 | upregulated lncRNA | -2.222282145 | 1.11E-24 |
| RP11-181G12.4 | upregulated lncRNA | -2.754668285 | 1.25E-24 |
| AC096558.1 | upregulated lncRNA | -3.053242096 | 1.26E-24 |
| RP3-404F18.5 | upregulated lncRNA | -2.689028014 | 1.37E-24 |
| RP5-1009E24.8 | upregulated lncRNA | -2.021174852 | 1.43E-24 |
| CTD-2616J11.2 | upregulated lncRNA | -2.908571702 | 1.46E-24 |
| AF011889.2 | upregulated lncRNA | -2.170399508 | 1.70E-24 |
| LINC01529 | upregulated lncRNA | -2.782570616 | 1.74E-24 |
| LIMS1-AS1 | upregulated lncRNA | -2.063040477 | 1.94E-24 |
| RP11-167B3.3 | upregulated lncRNA | -3.070394917 | 1.96E-24 |
| RP13-494C23.1 | upregulated lncRNA | -2.571856855 | 2.25E-24 |
| RP4-710M16.2 | upregulated lncRNA | -2.137339243 | 2.56E-24 |
| RP11-81H14.2 | upregulated lncRNA | -2.222624232 | 2.82E-24 |
| RP11-100G15.10 | upregulated lncRNA | -3.689327092 | 2.88E-24 |
| PCSK6-AS1 | upregulated lncRNA | -3.976172097 | 2.96E-24 |
| UNQ6494 | upregulated lncRNA | -2.075564247 | 3.11E-24 |
| RP11-3P17.4 | upregulated lncRNA | -2.878222958 | 3.57E-24 |
| CTD-2576D5.4 | upregulated lncRNA | -2.221471805 | 3.66E-24 |
| AC005757.6 | upregulated lncRNA | -3.238577732 | 3.96E-24 |
| RP11-421P23.2 | upregulated lncRNA | -2.970628876 | 4.52E-24 |
| AC006227.1 | upregulated lncRNA | -2.861870723 | 4.59E-24 |
| AC006262.5 | upregulated lncRNA | -4.107042028 | 7.34E-24 |
| RP11-763F8.1 | upregulated lncRNA | -2.546559892 | 7.69E-24 |
| RP11-302F12.10 | upregulated lncRNA | -3.228382714 | 7.80E-24 |
| RP11-483P21.6 | upregulated lncRNA | -2.052805264 | 8.06E-24 |
| RP3-460G2.2 | upregulated lncRNA | -2.255273055 | 8.14E-24 |
| RP11-20G13.2 | upregulated lncRNA | -2.216230154 | 9.20E-24 |
| CTA-384D8.34 | upregulated lncRNA | -2.526730787 | 1.10E-23 |
| TRBV11-2 | upregulated lncRNA | -2.300527482 | 1.17E-23 |
| RP11-713C5.1 | upregulated lncRNA | -2.570365909 | 1.26E-23 |
| CDKN2A-AS1 | upregulated lncRNA | -3.176942491 | 1.27E-23 |
| FIRRE | upregulated lncRNA | -2.206269485 | 1.42E-23 |
| CERS3-AS1 | upregulated lncRNA | -2.21993225 | 1.52E-23 |
| RP4-535B20.4 | upregulated lncRNA | -2.141809298 | 1.60E-23 |
| RP11-408O19.5 | upregulated lncRNA | -2.011835307 | 1.66E-23 |
| AC004906.3 | upregulated lncRNA | -3.409498407 | 1.71E-23 |
| RP11-91J3.3 | upregulated lncRNA | -4.077485703 | 1.88E-23 |
| VCAN-AS1 | upregulated lncRNA | -2.722992577 | 1.91E-23 |
| AC011524.1 | upregulated lncRNA | -3.421075532 | 1.93E-23 |
| IL21R-AS1 | upregulated lncRNA | -2.221506759 | 2.07E-23 |
| RP13-463N16.6 | upregulated lncRNA | -2.020963913 | 2.45E-23 |
| RP11-864I4.3 | upregulated lncRNA | -2.270636 | 2.70E-23 |
| AC007362.3 | upregulated lncRNA | -3.006329367 | 3.34E-23 |
| RP11-631F7.1 | upregulated lncRNA | -4.46604566 | 4.98E-23 |
| RP11-411B10.2 | upregulated lncRNA | -2.021449519 | 5.41E-23 |
| LINC01501 | upregulated lncRNA | -3.131823021 | 5.46E-23 |
| CTD-2263F21.1 | upregulated lncRNA | -2.204505685 | 5.50E-23 |
| RP11-20G13.3 | upregulated lncRNA | -2.125409788 | 6.86E-23 |
| RP11-404F10.2 | upregulated lncRNA | -2.300688636 | 8.84E-23 |
| RP11-429E11.3 | upregulated lncRNA | -2.982294276 | 9.39E-23 |
| RAPGEF4-AS1 | upregulated lncRNA | -2.956400671 | 9.90E-23 |
| CSNK1G2-AS1 | upregulated lncRNA | -2.34392443 | 1.01E-22 |
| RP11-13J8.1 | upregulated lncRNA | -2.066688954 | 1.05E-22 |
| AP000233.4 | upregulated lncRNA | -3.859455219 | 1.11E-22 |
| LINC01127 | upregulated lncRNA | -2.969144187 | 1.22E-22 |
| RP11-15I20.1 | upregulated lncRNA | -2.978502393 | 1.47E-22 |
| LINC01281 | upregulated lncRNA | -3.539162429 | 1.53E-22 |
| SLX1A-SULT1A3 | upregulated lncRNA | -2.299104994 | 1.54E-22 |
| RP11-124N19.3 | upregulated lncRNA | -2.33253646 | 2.11E-22 |
| RP11-146E13.4 | upregulated lncRNA | -3.457565697 | 2.18E-22 |
| RP11-757O6.1 | upregulated lncRNA | -2.20549656 | 2.24E-22 |
| CTC-459M5.1 | upregulated lncRNA | -2.45906139 | 2.29E-22 |
| RP11-867G23.1 | upregulated lncRNA | -2.31949866 | 2.41E-22 |
| LINC01583 | upregulated lncRNA | -4.156373019 | 2.54E-22 |
| RP11-53B2.1 | upregulated lncRNA | -2.648748318 | 2.57E-22 |
| CTC-340I23.2 | upregulated lncRNA | -3.514092432 | 3.23E-22 |
| RP11-75L1.1 | upregulated lncRNA | -2.720558574 | 4.05E-22 |
| LINC01021 | upregulated lncRNA | -2.797945267 | 4.54E-22 |
| AC064834.3 | upregulated lncRNA | -2.96464573 | 5.35E-22 |
| RP3-369A17.4 | upregulated lncRNA | -2.227164803 | 5.46E-22 |
| C3orf67-AS1 | upregulated lncRNA | -3.49256312 | 6.28E-22 |
| RP1-16A9.1 | upregulated lncRNA | -2.453059082 | 6.80E-22 |
| RP5-916L7.2 | upregulated lncRNA | -2.896958453 | 6.87E-22 |
| ELOVL2-AS1 | upregulated lncRNA | -3.287613742 | 7.53E-22 |
| AC003984.1 | upregulated lncRNA | -2.199003579 | 7.77E-22 |
| RP11-29P20.1 | upregulated lncRNA | -4.033582361 | 8.01E-22 |
| AC017060.1 | upregulated lncRNA | -2.430686223 | 8.39E-22 |
| RP11-347P5.1 | upregulated lncRNA | -2.012760195 | 9.43E-22 |
| RP11-493L12.5 | upregulated lncRNA | -3.227513385 | 1.03E-21 |
| RP5-921G16.2 | upregulated lncRNA | -3.668132818 | 1.08E-21 |
| CTB-35F21.1 | upregulated lncRNA | -2.135960147 | 1.10E-21 |
| CTD-2287O16.4 | upregulated lncRNA | -2.809328887 | 1.12E-21 |
| AC008991.1 | upregulated lncRNA | -3.495993368 | 1.34E-21 |
| CTA-243E7.4 | upregulated lncRNA | -2.696089546 | 1.37E-21 |
| RP11-133F8.2 | upregulated lncRNA | -2.233402499 | 1.61E-21 |
| ATP11A-AS1 | upregulated lncRNA | -3.128319721 | 1.76E-21 |
| RP11-60A8.1 | upregulated lncRNA | -2.482301964 | 1.85E-21 |
| RP5-912I13.1 | upregulated lncRNA | -5.955340275 | 2.07E-21 |
| RP1-56J10.8 | upregulated lncRNA | -2.25730561 | 2.15E-21 |
| CTB-186G2.1 | upregulated lncRNA | -3.147892038 | 2.21E-21 |
| RP11-815M8.1 | upregulated lncRNA | -4.343429524 | 2.23E-21 |
| RP11-148B3.2 | upregulated lncRNA | -3.063438208 | 2.33E-21 |
| RP11-357N13.1 | upregulated lncRNA | -2.231650525 | 2.56E-21 |
| AC000036.4 | upregulated lncRNA | -2.448232582 | 2.65E-21 |
| RP11-321P16.1 | upregulated lncRNA | -2.538715653 | 2.77E-21 |
| CTD-2587H19.3 | upregulated lncRNA | -2.642748702 | 3.10E-21 |
| AC019117.2 | upregulated lncRNA | -2.318205562 | 3.54E-21 |
| RP11-73M11.3 | upregulated lncRNA | -3.032834817 | 3.57E-21 |
| Z69666.2 | upregulated lncRNA | -2.01636515 | 3.77E-21 |
| RP11-809N8.4 | upregulated lncRNA | -2.356053398 | 4.29E-21 |
| LINC00632 | upregulated lncRNA | -2.384003475 | 5.11E-21 |
| RP11-16C1.2 | upregulated lncRNA | -3.018459938 | 5.22E-21 |
| RP11-290L1.3 | upregulated lncRNA | -2.3015368 | 5.99E-21 |
| RP11-332J15.4 | upregulated lncRNA | -2.940454986 | 6.34E-21 |
| RP5-1107A17.3 | upregulated lncRNA | -2.030199576 | 6.46E-21 |
| RP11-543P15.3 | upregulated lncRNA | -2.369704194 | 6.46E-21 |
| AC005954.3 | upregulated lncRNA | -2.180884198 | 7.94E-21 |
| STEAP3-AS1 | upregulated lncRNA | -2.005590326 | 8.14E-21 |
| RP11-486M23.1 | upregulated lncRNA | -2.289233089 | 8.21E-21 |
| FGF12-AS2 | upregulated lncRNA | -2.344895944 | 9.35E-21 |
| CTD-2562J17.2 | upregulated lncRNA | -4.001431978 | 9.68E-21 |
| AC096669.3 | upregulated lncRNA | -2.120505956 | 1.14E-20 |
| RP11-219E7.3 | upregulated lncRNA | -2.36886755 | 1.22E-20 |
| RP11-691H4.4 | upregulated lncRNA | -3.301591267 | 1.33E-20 |
| RP11-327F22.6 | upregulated lncRNA | -2.658405501 | 2.18E-20 |
| AC074366.3 | upregulated lncRNA | -3.256646468 | 2.19E-20 |
| RP11-856M7.2 | upregulated lncRNA | -3.024429755 | 2.40E-20 |
| AP000439.1 | upregulated lncRNA | -3.303135373 | 2.68E-20 |
| AC015849.2 | upregulated lncRNA | -2.268585765 | 2.88E-20 |
| RP11-642C5.1 | upregulated lncRNA | -2.369367598 | 3.25E-20 |
| RP13-216E22.5 | upregulated lncRNA | -2.505900949 | 4.18E-20 |
| RP11-93I21.3 | upregulated lncRNA | -2.021107985 | 4.31E-20 |
| RP1-90G24.11 | upregulated lncRNA | -2.319484417 | 5.79E-20 |
| CTD-2309O5.3 | upregulated lncRNA | -2.830506029 | 5.94E-20 |
| LINCMD1 | upregulated lncRNA | -2.049475294 | 6.77E-20 |
| RP11-108P20.2 | upregulated lncRNA | -2.728571785 | 7.62E-20 |
| RP11-240L7.4 | upregulated lncRNA | -2.019500895 | 7.96E-20 |
| RP11-737O24.1 | upregulated lncRNA | -2.810505584 | 9.02E-20 |
| LINC00705 | upregulated lncRNA | -3.275722488 | 9.76E-20 |
| LINC01513 | upregulated lncRNA | -2.072893022 | 1.22E-19 |
| RP11-407A16.3 | upregulated lncRNA | -3.758776261 | 1.32E-19 |
| RP1-158P9.1 | upregulated lncRNA | -2.394131185 | 1.35E-19 |
| AC096669.1 | upregulated lncRNA | -3.000992097 | 1.42E-19 |
| RP11-495K9.5 | upregulated lncRNA | -2.043640726 | 1.44E-19 |
| CTD-2532K18.2 | upregulated lncRNA | -5.059990514 | 1.49E-19 |
| RP11-867G23.12 | upregulated lncRNA | -2.505862503 | 1.56E-19 |
| RP11-575A19.2 | upregulated lncRNA | -2.089127726 | 1.57E-19 |
| LINC01433 | upregulated lncRNA | -2.480712565 | 1.65E-19 |
| IL20RB-AS1 | upregulated lncRNA | -4.250251755 | 1.66E-19 |
| AC011752.1 | upregulated lncRNA | -3.805558342 | 1.72E-19 |
| RP5-984P4.6 | upregulated lncRNA | -3.923335329 | 2.16E-19 |
| RP11-815J21.1 | upregulated lncRNA | -2.449753466 | 2.68E-19 |
| RP11-982M15.7 | upregulated lncRNA | -2.795264726 | 3.21E-19 |
| RP11-493P1.2 | upregulated lncRNA | -2.015071999 | 3.55E-19 |
| RP11-761I4.4 | upregulated lncRNA | -2.034013456 | 3.58E-19 |
| RP11-354K1.1 | upregulated lncRNA | -2.780616276 | 3.62E-19 |
| RP11-1105G2.4 | upregulated lncRNA | -2.072476256 | 3.87E-19 |
| LINC00313 | upregulated lncRNA | -3.058184546 | 4.15E-19 |
| LINC00942 | upregulated lncRNA | -2.696349233 | 4.87E-19 |
| RP11-554D20.1 | upregulated lncRNA | -2.513190496 | 5.69E-19 |
| RP11-617B3.2 | upregulated lncRNA | -2.557796354 | 6.53E-19 |
| AC097713.4 | upregulated lncRNA | -2.738954148 | 6.61E-19 |
| RP1-40E16.2 | upregulated lncRNA | -2.009572776 | 8.64E-19 |
| RP11-39M21.2 | upregulated lncRNA | -2.419826388 | 8.76E-19 |
| RP11-472G21.2 | upregulated lncRNA | -2.456872991 | 1.13E-18 |
| RP11-719N22.2 | upregulated lncRNA | -4.659891945 | 1.18E-18 |
| RP11-30L3.2 | upregulated lncRNA | -2.469419027 | 1.31E-18 |
| RP5-1096D14.3 | upregulated lncRNA | -2.555571682 | 1.36E-18 |
| RP1-118J21.25 | upregulated lncRNA | -2.0023348 | 1.53E-18 |
| SOX21-AS1 | upregulated lncRNA | -2.644745087 | 1.59E-18 |
| RNU12 | upregulated lncRNA | -2.16823878 | 1.90E-18 |
| RP11-229P13.15 | upregulated lncRNA | -3.008717341 | 1.92E-18 |
| RP11-357N13.3 | upregulated lncRNA | -2.806102592 | 1.96E-18 |
| CTB-73N10.1 | upregulated lncRNA | -2.872377505 | 2.50E-18 |
| RP11-554E23.2 | upregulated lncRNA | -2.706734846 | 2.52E-18 |
| AC008088.4 | upregulated lncRNA | -3.196403215 | 2.54E-18 |
| CTD-2542L18.1 | upregulated lncRNA | -2.588515123 | 2.60E-18 |
| AC116614.1 | upregulated lncRNA | -3.626890808 | 2.89E-18 |
| HP09025 | upregulated lncRNA | -2.456376135 | 3.25E-18 |
| RP11-809C18.3 | upregulated lncRNA | -2.335563141 | 3.36E-18 |
| CTD-2501M5.1 | upregulated lncRNA | -3.766048778 | 3.67E-18 |
| RP11-881L2.1 | upregulated lncRNA | -2.627362608 | 4.08E-18 |
| LINC00862 | upregulated lncRNA | -2.383187051 | 5.06E-18 |
| LINC00589 | upregulated lncRNA | -2.702226131 | 5.14E-18 |
| LINC00593 | upregulated lncRNA | -2.061483497 | 5.89E-18 |
| RP11-126O1.2 | upregulated lncRNA | -3.019034086 | 6.79E-18 |
| RP1-168P16.2 | upregulated lncRNA | -2.289787338 | 7.71E-18 |
| RP11-697N18.4 | upregulated lncRNA | -2.043733721 | 7.77E-18 |
| SALRNA2 | upregulated lncRNA | -2.088503035 | 9.62E-18 |
| RP11-989F5.3 | upregulated lncRNA | -2.195913383 | 1.02E-17 |
| RP11-229P13.19 | upregulated lncRNA | -2.332946671 | 1.19E-17 |
| RP11-276E17.2 | upregulated lncRNA | -2.728108471 | 1.38E-17 |
| RP11-301G7.1 | upregulated lncRNA | -4.019055685 | 1.45E-17 |
| AC069394.1 | upregulated lncRNA | -2.952730684 | 1.53E-17 |
| RP11-278A23.2 | upregulated lncRNA | -2.181030788 | 1.55E-17 |
| RP4-539M6.14 | upregulated lncRNA | -3.107476698 | 1.65E-17 |
| AC017104.2 | upregulated lncRNA | -2.76240401 | 1.77E-17 |
| RP11-471M2.3 | upregulated lncRNA | -4.314040876 | 1.80E-17 |
| LINC00162 | upregulated lncRNA | -2.341205583 | 1.81E-17 |
| CTD-2527I21.15 | upregulated lncRNA | -3.823098232 | 1.88E-17 |
| RP11-275I4.2 | upregulated lncRNA | -3.25496682 | 1.98E-17 |
| RP11-258F22.2 | upregulated lncRNA | -2.807112757 | 2.34E-17 |
| RP11-553L6.2 | upregulated lncRNA | -2.241317278 | 2.54E-17 |
| RP5-1073O3.2 | upregulated lncRNA | -2.43568229 | 3.00E-17 |
| LA16c-349E10.1 | upregulated lncRNA | -2.027444517 | 3.00E-17 |
| RP11-128B16.3 | upregulated lncRNA | -2.523811018 | 3.06E-17 |
| RP13-192B19.2 | upregulated lncRNA | -2.646885571 | 3.16E-17 |
| CTD-2529P6.3 | upregulated lncRNA | -2.052700695 | 3.23E-17 |
| RP11-429J17.5 | upregulated lncRNA | -2.658573381 | 3.67E-17 |
| RP11-445P17.3 | upregulated lncRNA | -3.041329419 | 3.72E-17 |
| CTC-344H19.4 | upregulated lncRNA | -2.431082639 | 3.86E-17 |
| RP11-578F21.6 | upregulated lncRNA | -2.029209844 | 3.89E-17 |
| RP11-909N17.2 | upregulated lncRNA | -3.312498338 | 4.06E-17 |
| RP11-831H9.3 | upregulated lncRNA | -2.394011382 | 4.32E-17 |
| RP11-322E11.2 | upregulated lncRNA | -2.022479564 | 4.63E-17 |
| CTC-435M10.10 | upregulated lncRNA | -3.181945154 | 5.01E-17 |
| RP11-25K21.6 | upregulated lncRNA | -2.649841687 | 5.07E-17 |
| AC007326.10 | upregulated lncRNA | -3.152920672 | 5.96E-17 |
| RP11-325E5.1 | upregulated lncRNA | -2.108564047 | 6.25E-17 |
| CTB-35F21.3 | upregulated lncRNA | -2.510463521 | 6.83E-17 |
| RP11-556O9.2 | upregulated lncRNA | -2.791475342 | 7.04E-17 |
| AC002044.4 | upregulated lncRNA | -2.614354836 | 7.33E-17 |
| RP11-19J5.2 | upregulated lncRNA | -2.916230524 | 7.42E-17 |
| CTB-33O18.2 | upregulated lncRNA | -2.27059886 | 7.52E-17 |
| RP11-227G15.8 | upregulated lncRNA | -2.093570536 | 8.17E-17 |
| RP5-1029K10.2 | upregulated lncRNA | -2.506792203 | 9.19E-17 |
| RP1-55C23.7 | upregulated lncRNA | -2.715842784 | 9.34E-17 |
| RP11-552M14.1 | upregulated lncRNA | -2.704943588 | 9.40E-17 |
| RP5-881P19.7 | upregulated lncRNA | -2.168762175 | 1.12E-16 |
| AP001056.1 | upregulated lncRNA | -2.332235376 | 1.32E-16 |
| CTA-833B7.2 | upregulated lncRNA | -2.492843841 | 1.36E-16 |
| RP11-861L17.4 | upregulated lncRNA | -2.155416392 | 1.38E-16 |
| KCNMB2-AS1 | upregulated lncRNA | -2.143154015 | 1.44E-16 |
| AC010894.5 | upregulated lncRNA | -2.58097653 | 1.55E-16 |
| RP11-283C24.1 | upregulated lncRNA | -2.479105768 | 1.68E-16 |
| RP3-495K2.2 | upregulated lncRNA | -4.174416089 | 1.81E-16 |
| RP5-1039K5.16 | upregulated lncRNA | -2.30980126 | 1.81E-16 |
| RP11-893F2.5 | upregulated lncRNA | -2.567801764 | 1.91E-16 |
| RP11-297C4.2 | upregulated lncRNA | -2.709903427 | 1.94E-16 |
| CTD-2589M5.5 | upregulated lncRNA | -2.805325366 | 2.08E-16 |
| RP11-486M23.2 | upregulated lncRNA | -3.220108488 | 2.13E-16 |
| RP11-353N4.5 | upregulated lncRNA | -2.362673633 | 2.14E-16 |
| LINC00384 | upregulated lncRNA | -2.359500379 | 2.21E-16 |
| RP11-19E11.1 | upregulated lncRNA | -3.916086302 | 2.30E-16 |
| RP11-61F12.1 | upregulated lncRNA | -2.071303653 | 2.50E-16 |
| LINC01411 | upregulated lncRNA | -4.18281521 | 2.75E-16 |
| RP1-144F13.3 | upregulated lncRNA | -2.586409715 | 3.25E-16 |
| CTD-3032J10.4 | upregulated lncRNA | -2.417462366 | 3.47E-16 |
| IFNG-AS1 | upregulated lncRNA | -2.030017094 | 3.49E-16 |
| RP11-58A18.1 | upregulated lncRNA | -2.602833932 | 3.94E-16 |
| RP11-856M7.1 | upregulated lncRNA | -2.435421224 | 3.95E-16 |
| AC116366.5 | upregulated lncRNA | -2.172358317 | 4.13E-16 |
| RP11-636O21.1 | upregulated lncRNA | -2.68626245 | 4.38E-16 |
| RP11-314N14.1 | upregulated lncRNA | -3.307751765 | 4.42E-16 |
| CTD-2376I20.1 | upregulated lncRNA | -2.686461897 | 4.50E-16 |
| RP11-750H9.7 | upregulated lncRNA | -2.959816785 | 4.68E-16 |
| AC004448.5 | upregulated lncRNA | -2.275571675 | 4.87E-16 |
| RP11-344P13.4 | upregulated lncRNA | -2.807913462 | 5.05E-16 |
| RP11-826N14.2 | upregulated lncRNA | -2.420054161 | 5.24E-16 |
| RP11-247A12.1 | upregulated lncRNA | -2.260632437 | 5.25E-16 |
| RP11-73M14.1 | upregulated lncRNA | -3.704711086 | 5.26E-16 |
| RP11-524N5.1 | upregulated lncRNA | -2.358499335 | 5.63E-16 |
| AP000797.3 | upregulated lncRNA | -3.050646245 | 5.98E-16 |
| RP11-84D1.2 | upregulated lncRNA | -3.053321536 | 6.18E-16 |
| AC093484.4 | upregulated lncRNA | -2.251140623 | 6.23E-16 |
| AC005550.3 | upregulated lncRNA | -3.446022096 | 6.24E-16 |
| RP3-413H6.2 | upregulated lncRNA | -2.442891336 | 6.47E-16 |
| RP11-318A15.8 | upregulated lncRNA | -2.485433745 | 6.86E-16 |
| AC097382.5 | upregulated lncRNA | -2.502549792 | 7.13E-16 |
| LL22NC03-63E9.3 | upregulated lncRNA | -3.45254361 | 7.24E-16 |
| RP11-543G18.1 | upregulated lncRNA | -2.80044148 | 7.94E-16 |
| RP1-69D17.3 | upregulated lncRNA | -2.068065837 | 8.20E-16 |
| LINC00838 | upregulated lncRNA | -2.871819936 | 8.38E-16 |
| RP1-167G20.1 | upregulated lncRNA | -2.83858151 | 9.02E-16 |
| RP11-356I2.1 | upregulated lncRNA | -3.134269491 | 9.11E-16 |
| RP11-403I13.5 | upregulated lncRNA | -2.007481646 | 9.28E-16 |
| RP11-103C16.2 | upregulated lncRNA | -2.020111761 | 9.77E-16 |
| RP11-799B12.2 | upregulated lncRNA | -2.145333036 | 1.07E-15 |
| RP11-65J3.2 | upregulated lncRNA | -2.063080991 | 1.08E-15 |
| LINC01559 | upregulated lncRNA | -2.991782312 | 1.10E-15 |
| RP11-563N6.6 | upregulated lncRNA | -2.622333749 | 1.11E-15 |
| SALRNA1 | upregulated lncRNA | -2.847454488 | 1.14E-15 |
| RP11-326C3.14 | upregulated lncRNA | -2.370519496 | 1.18E-15 |
| RP4-806M20.4 | upregulated lncRNA | -2.554803533 | 1.22E-15 |
| RP11-239L20.6 | upregulated lncRNA | -2.093019001 | 1.39E-15 |
| RP1-207H1.3 | upregulated lncRNA | -2.762159537 | 1.39E-15 |
| CTB-31O20.8 | upregulated lncRNA | -2.142728611 | 1.41E-15 |
| RP11-357N13.2 | upregulated lncRNA | -2.524744422 | 1.42E-15 |
| GS1-174L6.4 | upregulated lncRNA | -2.43698687 | 1.47E-15 |
| LINC01526 | upregulated lncRNA | -2.633987182 | 1.60E-15 |
| RP11-344N10.2 | upregulated lncRNA | -2.675094619 | 1.71E-15 |
| RP11-464D20.6 | upregulated lncRNA | -2.07222758 | 1.77E-15 |
| AC091153.4 | upregulated lncRNA | -2.578037086 | 1.79E-15 |
| RP11-422J15.1 | upregulated lncRNA | -3.556486034 | 2.13E-15 |
| AC005152.3 | upregulated lncRNA | -2.410765687 | 2.26E-15 |
| RP11-452L6.8 | upregulated lncRNA | -2.334755387 | 2.28E-15 |
| RP11-145M4.1 | upregulated lncRNA | -2.537261901 | 2.30E-15 |
| CTD-2024I7.13 | upregulated lncRNA | -2.283805031 | 2.34E-15 |
| LINC01280 | upregulated lncRNA | -3.029258395 | 2.39E-15 |
| RP11-1C1.4 | upregulated lncRNA | -2.698435527 | 2.60E-15 |
| LRP1-AS | upregulated lncRNA | -2.091010225 | 2.62E-15 |
| LINC00161 | upregulated lncRNA | -2.277939333 | 2.93E-15 |
| RP11-465K16.1 | upregulated lncRNA | -3.841988172 | 2.95E-15 |
| CTA-305I2.1 | upregulated lncRNA | -2.365953935 | 3.13E-15 |
| HOTTIP | upregulated lncRNA | -3.261018421 | 3.17E-15 |
| U95743.1 | upregulated lncRNA | -2.064076369 | 3.44E-15 |
| CTD-2528A14.5 | upregulated lncRNA | -2.528233826 | 3.64E-15 |
| AC009784.3 | upregulated lncRNA | -2.092827002 | 3.82E-15 |
| RP11-616M22.1 | upregulated lncRNA | -2.15884928 | 4.00E-15 |
| CTD-3032J10.3 | upregulated lncRNA | -2.429734196 | 4.08E-15 |
| CTD-2013N17.4 | upregulated lncRNA | -2.802296449 | 4.93E-15 |
| CTD-2354A18.1 | upregulated lncRNA | -2.943042194 | 5.14E-15 |
| RP4-569D19.8 | upregulated lncRNA | -2.633080247 | 5.44E-15 |
| AC007278.2 | upregulated lncRNA | -2.384189971 | 5.69E-15 |
| RP11-114M1.2 | upregulated lncRNA | -3.758861784 | 5.71E-15 |
| LINC00303 | upregulated lncRNA | -2.137671515 | 7.17E-15 |
| AC138430.4 | upregulated lncRNA | -2.26054752 | 7.94E-15 |
| RP11-114H24.7 | upregulated lncRNA | -2.59213072 | 9.27E-15 |
| RP11-70J12.1 | upregulated lncRNA | -2.345277736 | 1.02E-14 |
| CTC-232P5.3 | upregulated lncRNA | -2.233274702 | 1.05E-14 |
| Z69720.2 | upregulated lncRNA | -2.05918135 | 1.07E-14 |
| DSCAM-AS1 | upregulated lncRNA | -4.490740639 | 1.08E-14 |
| RP11-1137G4.3 | upregulated lncRNA | -2.651531495 | 1.14E-14 |
| RP1-71H24.1 | upregulated lncRNA | -2.693760075 | 1.30E-14 |
| LINC00928 | upregulated lncRNA | -3.288279063 | 1.45E-14 |
| RP11-569G13.3 | upregulated lncRNA | -3.038200714 | 1.49E-14 |
| RP11-478P10.1 | upregulated lncRNA | -2.761093994 | 1.53E-14 |
| RP1-102K2.6 | upregulated lncRNA | -2.65215467 | 1.73E-14 |
| RP11-805I24.1 | upregulated lncRNA | -2.994838831 | 1.82E-14 |
| RP11-455F5.4 | upregulated lncRNA | -2.032934524 | 1.86E-14 |
| AC003088.1 | upregulated lncRNA | -2.73179727 | 1.90E-14 |
| LINC00824 | upregulated lncRNA | -2.260996663 | 1.94E-14 |
| RP11-445N18.5 | upregulated lncRNA | -3.144133184 | 2.07E-14 |
| CTC-503J8.4 | upregulated lncRNA | -3.198206108 | 2.07E-14 |
| RP11-354P11.8 | upregulated lncRNA | -2.807015962 | 2.10E-14 |
| AOAH-IT1 | upregulated lncRNA | -2.498613177 | 2.41E-14 |
| RP11-662G23.1 | upregulated lncRNA | -3.05256666 | 2.72E-14 |
| AC114730.7 | upregulated lncRNA | -2.069928056 | 2.84E-14 |
| RP11-115D19.1 | upregulated lncRNA | -2.662268677 | 2.99E-14 |
| AC011893.3 | upregulated lncRNA | -2.516001271 | 3.38E-14 |
| AC092620.3 | upregulated lncRNA | -2.188759046 | 3.39E-14 |
| RP11-138I1.2 | upregulated lncRNA | -2.350239845 | 3.39E-14 |
| RP11-310H4.2 | upregulated lncRNA | -2.36532969 | 3.39E-14 |
| RP11-533O20.2 | upregulated lncRNA | -2.364347881 | 3.46E-14 |
| AC141930.2 | upregulated lncRNA | -2.516132054 | 3.70E-14 |
| RP11-360I2.1 | upregulated lncRNA | -2.497353597 | 3.79E-14 |
| LINC00427 | upregulated lncRNA | -2.000048226 | 3.87E-14 |
| RP11-377G16.2 | upregulated lncRNA | -3.470270089 | 3.93E-14 |
| RP11-2O17.2 | upregulated lncRNA | -2.536733091 | 3.97E-14 |
| RP11-2E17.2 | upregulated lncRNA | -2.435586149 | 4.04E-14 |
| RP11-310H4.3 | upregulated lncRNA | -2.459722323 | 4.31E-14 |
| CTC-241F20.4 | upregulated lncRNA | -3.374273353 | 4.77E-14 |
| MTUS2-AS1 | upregulated lncRNA | -2.959393294 | 4.93E-14 |
| RP11-295I5.4 | upregulated lncRNA | -2.015304628 | 5.00E-14 |
| CTD-2282P23.2 | upregulated lncRNA | -2.71497361 | 5.01E-14 |
| AC012668.2 | upregulated lncRNA | -2.811690047 | 5.02E-14 |
| CTD-2568A17.1 | upregulated lncRNA | -2.035579204 | 5.19E-14 |
| AC003092.1 | upregulated lncRNA | -2.821358349 | 5.27E-14 |
| LINC00272 | upregulated lncRNA | -2.49556232 | 5.72E-14 |
| RP11-616M22.2 | upregulated lncRNA | -2.073449004 | 5.75E-14 |
| RP11-75C10.9 | upregulated lncRNA | -2.11288825 | 6.69E-14 |
| CTB-181H17.1 | upregulated lncRNA | -2.040965777 | 6.86E-14 |
| CTB-50L17.5 | upregulated lncRNA | -2.540031113 | 6.89E-14 |
| RP11-867G23.13 | upregulated lncRNA | -2.290280205 | 7.29E-14 |
| DOCK4-AS1 | upregulated lncRNA | -2.172240992 | 7.47E-14 |
| LINC01479 | upregulated lncRNA | -2.157746433 | 7.53E-14 |
| AC012462.2 | upregulated lncRNA | -2.762112548 | 7.63E-14 |
| CTD-2583P5.3 | upregulated lncRNA | -2.34860687 | 7.92E-14 |
| RP11-678G15.1 | upregulated lncRNA | -2.819041356 | 8.53E-14 |
| AC093642.6 | upregulated lncRNA | -2.834433686 | 8.54E-14 |
| TBL1XR1-AS1 | upregulated lncRNA | -2.131092811 | 8.70E-14 |
| RP11-640N11.2 | upregulated lncRNA | -2.766594548 | 1.03E-13 |
| CTD-2530N21.5 | upregulated lncRNA | -2.584444435 | 1.04E-13 |
| AC016700.2 | upregulated lncRNA | -2.36549539 | 1.12E-13 |
| RP11-332J15.1 | upregulated lncRNA | -2.400649084 | 1.13E-13 |
| RP11-459O16.8 | upregulated lncRNA | -2.351676248 | 1.13E-13 |
| GPC6-AS2 | upregulated lncRNA | -2.51444407 | 1.13E-13 |
| RP11-440G9.1 | upregulated lncRNA | -3.38686726 | 1.14E-13 |
| CTD-2562J17.4 | upregulated lncRNA | -2.636460606 | 1.24E-13 |
| RP11-370B11.3 | upregulated lncRNA | -2.270173968 | 1.26E-13 |
| RP11-570L15.1 | upregulated lncRNA | -2.240664795 | 1.32E-13 |
| KCNK4-TEX40 | upregulated lncRNA | -2.732950963 | 1.40E-13 |
| RP1-149A16.12 | upregulated lncRNA | -2.61537011 | 1.41E-13 |
| CARS-AS1 | upregulated lncRNA | -2.000760175 | 1.41E-13 |
| RP11-202G18.1 | upregulated lncRNA | -2.569165832 | 1.41E-13 |
| RP3-395M20.3 | upregulated lncRNA | -2.500737779 | 1.44E-13 |
| CTD-2373N4.5 | upregulated lncRNA | -2.232620009 | 1.44E-13 |
| NUCB1-AS1 | upregulated lncRNA | -2.027288425 | 1.46E-13 |
| AC112715.2 | upregulated lncRNA | -2.663189127 | 1.50E-13 |
| RP11-526F3.1 | upregulated lncRNA | -2.018538781 | 1.59E-13 |
| LINC01468 | upregulated lncRNA | -2.584918781 | 1.61E-13 |
| AC092295.4 | upregulated lncRNA | -2.168165992 | 1.62E-13 |
| LZTS1-AS1 | upregulated lncRNA | -2.866023232 | 1.88E-13 |
| GS1-279B7.1 | upregulated lncRNA | -2.210440819 | 1.94E-13 |
| RP11-307O13.1 | upregulated lncRNA | -2.376588256 | 2.10E-13 |
| RP11-45M22.5 | upregulated lncRNA | -2.039888237 | 2.15E-13 |
| AKT3-IT1 | upregulated lncRNA | -2.010226731 | 2.20E-13 |
| RP11-219E7.2 | upregulated lncRNA | -2.173354939 | 2.41E-13 |
| CTD-2587H19.2 | upregulated lncRNA | -2.129067856 | 2.45E-13 |
| RP11-508N22.9 | upregulated lncRNA | -2.02763586 | 2.47E-13 |
| LINC01522 | upregulated lncRNA | -2.421225952 | 2.61E-13 |
| LINC01305 | upregulated lncRNA | -2.85042307 | 2.75E-13 |
| CTD-2116N20.1 | upregulated lncRNA | -2.035484697 | 2.87E-13 |
| RP11-616M22.5 | upregulated lncRNA | -2.409971393 | 2.89E-13 |
| CASC6 | upregulated lncRNA | -3.408429703 | 3.00E-13 |
| RP11-568J23.4 | upregulated lncRNA | -2.77355121 | 3.28E-13 |
| RP11-474I11.8 | upregulated lncRNA | -2.510176143 | 3.29E-13 |
| GRID1-AS1 | upregulated lncRNA | -2.599485989 | 3.35E-13 |
| CTD-2196E14.5 | upregulated lncRNA | -2.191093108 | 3.44E-13 |
| CTB-174D11.2 | upregulated lncRNA | -2.26176077 | 3.47E-13 |
| TTLL7-IT1 | upregulated lncRNA | -2.671427922 | 3.65E-13 |
| RP6-91H8.5 | upregulated lncRNA | -2.700655631 | 3.71E-13 |
| RP11-64D24.2 | upregulated lncRNA | -2.089537953 | 3.81E-13 |
| CTA-243E7.3 | upregulated lncRNA | -2.329349055 | 3.87E-13 |
| RP4-802A10.1 | upregulated lncRNA | -2.339961782 | 3.92E-13 |
| RP11-946L16.2 | upregulated lncRNA | -2.651801653 | 4.25E-13 |
| CTD-2168K21.1 | upregulated lncRNA | -2.314443767 | 4.25E-13 |
| RP11-883A18.3 | upregulated lncRNA | -2.102806749 | 4.37E-13 |
| CTB-138E5.1 | upregulated lncRNA | -2.745095654 | 4.54E-13 |
| LINC01206 | upregulated lncRNA | -2.783934405 | 4.60E-13 |
| RP11-412P11.1 | upregulated lncRNA | -2.347044222 | 4.71E-13 |
| RP11-416A14.1 | upregulated lncRNA | -2.302212102 | 5.11E-13 |
| RP11-42A4.1 | upregulated lncRNA | -2.276883415 | 5.50E-13 |
| RP11-138H8.2 | upregulated lncRNA | -2.417590621 | 5.53E-13 |
| RP11-10O22.1 | upregulated lncRNA | -4.461268026 | 5.60E-13 |
| GK-IT1 | upregulated lncRNA | -2.042550607 | 5.81E-13 |
| CTD-2621I17.6 | upregulated lncRNA | -2.114986344 | 6.06E-13 |
| RP5-837J1.4 | upregulated lncRNA | -2.032138902 | 6.32E-13 |
| AC007040.8 | upregulated lncRNA | -2.241590999 | 6.54E-13 |
| RP11-632K5.3 | upregulated lncRNA | -2.285331615 | 6.64E-13 |
| LINC00102 | upregulated lncRNA | -2.113004484 | 7.30E-13 |
| RP13-210D15.4 | upregulated lncRNA | -2.182972611 | 7.50E-13 |
| RP11-94H18.1 | upregulated lncRNA | -2.403767784 | 7.53E-13 |
| CTD-3195I5.4 | upregulated lncRNA | -2.596384899 | 8.70E-13 |
| RP5-1077H22.1 | upregulated lncRNA | -2.574050755 | 8.72E-13 |
| CTB-33O18.1 | upregulated lncRNA | -3.3816368 | 8.90E-13 |
| RP11-429E11.2 | upregulated lncRNA | -3.689827878 | 9.40E-13 |
| CTB-178M22.1 | upregulated lncRNA | -3.047138814 | 9.61E-13 |
| CTA-339C12.1 | upregulated lncRNA | -2.418510947 | 9.87E-13 |
| NALCN-AS1 | upregulated lncRNA | -2.714926696 | 1.01E-12 |
| RP11-28G8.1 | upregulated lncRNA | -2.231691392 | 1.11E-12 |
| RP11-3P22.2 | upregulated lncRNA | -2.489380777 | 1.20E-12 |
| RP11-1191J2.4 | upregulated lncRNA | -2.2624926 | 1.28E-12 |
| CTC-360G5.6 | upregulated lncRNA | -2.173912702 | 1.31E-12 |
| RP11-785D18.3 | upregulated lncRNA | -2.408371376 | 1.33E-12 |
| RP11-462B18.2 | upregulated lncRNA | -2.315684409 | 1.41E-12 |
| RP3-438O4.4 | upregulated lncRNA | -2.41099288 | 1.45E-12 |
| AC002306.1 | upregulated lncRNA | -2.373796044 | 1.48E-12 |
| RP11-16E23.3 | upregulated lncRNA | -2.345733507 | 1.54E-12 |
| RP11-165M1.3 | upregulated lncRNA | -2.023501499 | 1.61E-12 |
| SLC7A11-AS1 | upregulated lncRNA | -2.228439189 | 1.66E-12 |
| RP11-565A3.2 | upregulated lncRNA | -2.990179586 | 1.73E-12 |
| RP3-492J12.2 | upregulated lncRNA | -2.063290196 | 1.73E-12 |
| RP4-712E4.1 | upregulated lncRNA | -2.644425559 | 1.80E-12 |
| RP3-388N13.3 | upregulated lncRNA | -2.139088254 | 2.03E-12 |
| AC106873.4 | upregulated lncRNA | -2.873223639 | 2.14E-12 |
| RP11-319G9.5 | upregulated lncRNA | -2.051957308 | 2.17E-12 |
| RP11-1086F11.1 | upregulated lncRNA | -2.004727332 | 2.17E-12 |
| ARHGAP26-IT1 | upregulated lncRNA | -2.304022498 | 2.17E-12 |
| RP11-1C1.6 | upregulated lncRNA | -2.584151104 | 2.22E-12 |
| RP11-414H23.2 | upregulated lncRNA | -2.92480399 | 2.33E-12 |
| BX255923.3 | upregulated lncRNA | -2.375025946 | 2.39E-12 |
| RP11-338E21.2 | upregulated lncRNA | -2.652320003 | 2.41E-12 |
| RP11-886P16.6 | upregulated lncRNA | -2.015517459 | 2.43E-12 |
| CTC-529L17.2 | upregulated lncRNA | -2.067574284 | 2.51E-12 |
| RP11-831A10.2 | upregulated lncRNA | -2.210378773 | 2.53E-12 |
| RP11-39M21.1 | upregulated lncRNA | -2.470057471 | 2.55E-12 |
| RP11-41O4.2 | upregulated lncRNA | -2.525219108 | 2.56E-12 |
| RP13-297E16.5 | upregulated lncRNA | -2.41333446 | 2.56E-12 |
| AC104532.3 | upregulated lncRNA | -2.214075461 | 2.59E-12 |
| RP11-538I12.3 | upregulated lncRNA | -2.073686634 | 2.70E-12 |
| TBX5-AS1 | upregulated lncRNA | -2.901548779 | 2.71E-12 |
| RP1-142L7.8 | upregulated lncRNA | -2.602125865 | 2.72E-12 |
| GM140 | upregulated lncRNA | -2.238788614 | 2.86E-12 |
| RNF216-IT1 | upregulated lncRNA | -2.160258566 | 3.04E-12 |
| WASF3-AS1 | upregulated lncRNA | -3.161113039 | 3.16E-12 |
| CTD-3032J10.2 | upregulated lncRNA | -2.361856765 | 3.18E-12 |
| RP11-554D15.1 | upregulated lncRNA | -4.110169925 | 3.18E-12 |
| RP11-338O1.2 | upregulated lncRNA | -2.376312424 | 3.54E-12 |
| RP11-298O21.6 | upregulated lncRNA | -2.441562439 | 3.60E-12 |
| AC078842.3 | upregulated lncRNA | -2.652201161 | 3.61E-12 |
| KCNMA1-AS3 | upregulated lncRNA | -2.695896704 | 3.62E-12 |
| AC019064.1 | upregulated lncRNA | -2.689971882 | 3.63E-12 |
| CTD-2240J17.4 | upregulated lncRNA | -2.240021345 | 3.67E-12 |
| CTB-193M12.3 | upregulated lncRNA | -2.332223418 | 3.77E-12 |
| RP1-29C18.10 | upregulated lncRNA | -2.429936362 | 3.79E-12 |
| RP11-279O17.1 | upregulated lncRNA | -3.069451451 | 3.96E-12 |
| RP11-177B4.2 | upregulated lncRNA | -2.387658131 | 4.07E-12 |
| PHEX-AS1 | upregulated lncRNA | -2.138780605 | 4.70E-12 |
| RP11-776H12.1 | upregulated lncRNA | -2.485839906 | 4.74E-12 |
| AF064858.10 | upregulated lncRNA | -2.094593871 | 4.84E-12 |
| RP11-616M22.3 | upregulated lncRNA | -3.051627814 | 5.08E-12 |
| MUC2 | upregulated lncRNA | -2.284574346 | 5.19E-12 |
| AC092657.2 | upregulated lncRNA | -2.829869336 | 5.20E-12 |
| LINGO1-AS1 | upregulated lncRNA | -2.118420485 | 5.35E-12 |
| AC104699.1 | upregulated lncRNA | -2.124118846 | 5.42E-12 |
| AC114812.8 | upregulated lncRNA | -2.023400094 | 5.56E-12 |
| RP11-191N8.2 | upregulated lncRNA | -3.123508524 | 5.64E-12 |
| AC097495.2 | upregulated lncRNA | -2.002548932 | 5.67E-12 |
| KB-1460A1.2 | upregulated lncRNA | -2.180821688 | 8.20E-12 |
| RP11-1084E5.1 | upregulated lncRNA | -2.272578989 | 8.24E-12 |
| RP11-569G13.2 | upregulated lncRNA | -2.11305199 | 8.48E-12 |
| AC024084.1 | upregulated lncRNA | -2.418442066 | 8.61E-12 |
| CH17-360D5.2 | upregulated lncRNA | -3.058467252 | 8.70E-12 |
| AC005522.7 | upregulated lncRNA | -2.754304602 | 9.33E-12 |
| RP4-694A7.2 | upregulated lncRNA | -2.102313903 | 9.44E-12 |
| RP11-696F12.1 | upregulated lncRNA | -2.306378382 | 9.89E-12 |
| RP11-34F13.3 | upregulated lncRNA | -2.070069907 | 1.02E-11 |
| RP11-445N18.7 | upregulated lncRNA | -2.562955506 | 1.04E-11 |
| LINC01531 | upregulated lncRNA | -2.112307302 | 1.11E-11 |
| RP11-304L19.4 | upregulated lncRNA | -2.194861803 | 1.21E-11 |
| RP11-278H7.4 | upregulated lncRNA | -2.075850971 | 1.27E-11 |
| RP11-474I11.7 | upregulated lncRNA | -2.100909352 | 1.27E-11 |
| AC023669.1 | upregulated lncRNA | -2.598870552 | 1.32E-11 |
| CTA-722E9.1 | upregulated lncRNA | -2.148476113 | 1.32E-11 |
| RP11-91K8.5 | upregulated lncRNA | -2.034353956 | 1.36E-11 |
| RP5-896L10.1 | upregulated lncRNA | -2.6868915 | 1.38E-11 |
| RP11-383C6.2 | upregulated lncRNA | -2.000982722 | 1.51E-11 |
| AC018742.1 | upregulated lncRNA | -2.985956169 | 1.58E-11 |
| AC008592.5 | upregulated lncRNA | -2.063899766 | 1.58E-11 |
| TBX18-AS1 | upregulated lncRNA | -2.208386722 | 1.72E-11 |
| RP13-884E18.4 | upregulated lncRNA | -2.592401471 | 1.77E-11 |
| RP5-1057B20.3 | upregulated lncRNA | -2.058170021 | 1.81E-11 |
| LINC00165 | upregulated lncRNA | -2.204249402 | 1.86E-11 |
| RP11-5L12.1 | upregulated lncRNA | -3.099817577 | 1.91E-11 |
| RP13-60M5.2 | upregulated lncRNA | -2.764453791 | 1.98E-11 |
| JAKMIP2-AS1 | upregulated lncRNA | -2.214821035 | 2.08E-11 |
| RP11-10A14.6 | upregulated lncRNA | -2.201985961 | 2.20E-11 |
| RP5-867C24.4 | upregulated lncRNA | -2.29802463 | 2.22E-11 |
| CTC-268N12.3 | upregulated lncRNA | -2.281721539 | 2.24E-11 |
| RP11-149I2.4 | upregulated lncRNA | -2.17035489 | 2.28E-11 |
| RP11-126O1.4 | upregulated lncRNA | -2.142085239 | 2.35E-11 |
| ANKRD33B-AS1 | upregulated lncRNA | -2.227485985 | 2.38E-11 |
| RP11-1250I15.3 | upregulated lncRNA | -2.498307246 | 2.39E-11 |
| RP3-323N1.2 | upregulated lncRNA | -2.87044624 | 2.55E-11 |
| KB-173C10.1 | upregulated lncRNA | -2.466921725 | 2.71E-11 |
| RP11-495O11.1 | upregulated lncRNA | -2.082743294 | 2.84E-11 |
| MNX1-AS2 | upregulated lncRNA | -2.409752833 | 2.86E-11 |
| CTD-2363C16.1 | upregulated lncRNA | -2.706137521 | 3.01E-11 |
| RP11-172F10.1 | upregulated lncRNA | -2.664064284 | 3.57E-11 |
| RP11-269G24.6 | upregulated lncRNA | -2.171539382 | 3.67E-11 |
| CTD-2118P12.1 | upregulated lncRNA | -2.751404793 | 3.73E-11 |
| MNX1-AS1 | upregulated lncRNA | -2.955301181 | 3.93E-11 |
| XIAP-AS1 | upregulated lncRNA | -2.525932201 | 4.07E-11 |
| LINC01405 | upregulated lncRNA | -3.035943217 | 4.41E-11 |
| RP11-893F2.6 | upregulated lncRNA | -2.324557643 | 4.47E-11 |
| RP11-415D17.3 | upregulated lncRNA | -2.405224831 | 4.60E-11 |
| RP11-96A15.1 | upregulated lncRNA | -2.683115624 | 5.00E-11 |
| AC009264.1 | upregulated lncRNA | -2.879300979 | 5.11E-11 |
| LINC01397 | upregulated lncRNA | -2.173535153 | 5.16E-11 |
| RP11-521M14.2 | upregulated lncRNA | -3.027167191 | 6.48E-11 |
| RP11-650L12.1 | upregulated lncRNA | -2.332121241 | 7.23E-11 |
| ZBTB46-AS1 | upregulated lncRNA | -2.433021072 | 7.28E-11 |
| RP11-267C16.1 | upregulated lncRNA | -2.490784802 | 7.40E-11 |
| RP11-146N23.4 | upregulated lncRNA | -2.341509947 | 7.40E-11 |
| AF131215.4 | upregulated lncRNA | -2.369007309 | 7.70E-11 |
| RP11-127I20.7 | upregulated lncRNA | -2.054549548 | 7.89E-11 |
| RP11-88H10.2 | upregulated lncRNA | -2.655226733 | 8.00E-11 |
| CTA-797E19.1 | upregulated lncRNA | -2.328244813 | 8.58E-11 |
| RP11-196H14.4 | upregulated lncRNA | -2.091272035 | 9.13E-11 |
| RP11-629G13.1 | upregulated lncRNA | -2.22775621 | 9.25E-11 |
| ASH1L-IT1 | upregulated lncRNA | -2.464429918 | 9.38E-11 |
| RP11-14J7.6 | upregulated lncRNA | -2.393091622 | 1.05E-10 |
| MLIP-AS1 | upregulated lncRNA | -2.643289887 | 1.05E-10 |
| RP5-867C24.5 | upregulated lncRNA | -2.047553257 | 1.11E-10 |
| RP5-1195D24.1 | upregulated lncRNA | -2.601681689 | 1.11E-10 |
| RP11-10C8.2 | upregulated lncRNA | -2.473404339 | 1.15E-10 |
| RP11-43D2.2 | upregulated lncRNA | -2.053561515 | 1.26E-10 |
| CTD-2553L13.4 | upregulated lncRNA | -2.216658827 | 1.35E-10 |
| RP3-359N14.2 | upregulated lncRNA | -2.025126229 | 1.37E-10 |
| RP11-274H24.1 | upregulated lncRNA | -2.064126573 | 1.38E-10 |
| CTA-276F8.2 | upregulated lncRNA | -2.296737797 | 1.39E-10 |
| RP11-169E6.4 | upregulated lncRNA | -2.282674636 | 1.43E-10 |
| AC003009.1 | upregulated lncRNA | -2.441609201 | 1.60E-10 |
| AC009305.1 | upregulated lncRNA | -2.128299684 | 1.67E-10 |
| RP11-290O12.2 | upregulated lncRNA | -2.007594084 | 1.69E-10 |
| RP11-184I16.4 | upregulated lncRNA | -2.105208776 | 1.72E-10 |
| LINC01551 | upregulated lncRNA | -2.98204195 | 1.76E-10 |
| LINC01227 | upregulated lncRNA | -2.14761045 | 1.76E-10 |
| RP3-527G5.1 | upregulated lncRNA | -2.121648087 | 1.81E-10 |
| EML2-AS1 | upregulated lncRNA | -2.27913643 | 1.85E-10 |
| CTD-2034I4.2 | upregulated lncRNA | -2.311501456 | 1.94E-10 |
| RP11-1140I5.1 | upregulated lncRNA | -2.242620059 | 1.94E-10 |
| CTD-2193G5.1 | upregulated lncRNA | -2.072456471 | 1.96E-10 |
| AC006458.3 | upregulated lncRNA | -2.194845664 | 2.03E-10 |
| AC020956.3 | upregulated lncRNA | -2.667373601 | 2.18E-10 |
| PHACTR2-AS1 | upregulated lncRNA | -2.135999348 | 2.21E-10 |
| RP11-417L19.2 | upregulated lncRNA | -2.644425411 | 2.33E-10 |
| LINC00698 | upregulated lncRNA | -2.859781078 | 2.34E-10 |
| CTC-329D1.3 | upregulated lncRNA | -2.663801413 | 2.42E-10 |
| CTD-2085J24.3 | upregulated lncRNA | -2.153275206 | 2.55E-10 |
| AC007879.3 | upregulated lncRNA | -2.008868752 | 2.59E-10 |
| RP5-921G16.1 | upregulated lncRNA | -2.421853914 | 2.60E-10 |
| RP1-205F14P.1 | upregulated lncRNA | -2.548576267 | 2.70E-10 |
| AC069155.1 | upregulated lncRNA | -2.164545199 | 2.75E-10 |
| RP11-474B12.1 | upregulated lncRNA | -2.347877167 | 2.83E-10 |
| RP11-818F20.5 | upregulated lncRNA | -2.169436574 | 2.85E-10 |
| RP11-267L5.1 | upregulated lncRNA | -3.467496683 | 2.90E-10 |
| RP11-476K15.1 | upregulated lncRNA | -2.820377192 | 3.14E-10 |
| RP11-113I22.1 | upregulated lncRNA | -2.134138125 | 3.41E-10 |
| RP6-91H8.1 | upregulated lncRNA | -2.544104917 | 3.42E-10 |
| CTB-35F21.2 | upregulated lncRNA | -2.306926209 | 3.45E-10 |
| CTD-2130O13.1 | upregulated lncRNA | -2.713404723 | 3.47E-10 |
| RP11-161D15.1 | upregulated lncRNA | -4.120997089 | 3.50E-10 |
| WWC3-AS1 | upregulated lncRNA | -2.240630401 | 3.52E-10 |
| KB-1980E6.3 | upregulated lncRNA | -2.242923037 | 3.55E-10 |
| RP4-640H8.2 | upregulated lncRNA | -2.525930315 | 3.79E-10 |
| AC007050.17 | upregulated lncRNA | -2.358599646 | 3.90E-10 |
| MGC39584 | upregulated lncRNA | -3.23609476 | 4.09E-10 |
| RP1-111C20.3 | upregulated lncRNA | -2.403776312 | 4.33E-10 |
| RP11-526N18.1 | upregulated lncRNA | -2.025610233 | 4.41E-10 |
| RP11-162J8.3 | upregulated lncRNA | -2.088303137 | 4.47E-10 |
| AP001063.1 | upregulated lncRNA | -2.279764332 | 4.48E-10 |
| RP1-225E12.3 | upregulated lncRNA | -2.122958893 | 4.58E-10 |
| DAPK1-IT1 | upregulated lncRNA | -2.089996751 | 4.62E-10 |
| LINC01030 | upregulated lncRNA | -2.20007018 | 4.71E-10 |
| RP11-586D19.2 | upregulated lncRNA | -2.628419641 | 4.79E-10 |
| AC004840.8 | upregulated lncRNA | -2.141153822 | 4.90E-10 |
| RP11-9N12.2 | upregulated lncRNA | -2.552951468 | 5.03E-10 |
| AC002480.2 | upregulated lncRNA | -2.383343859 | 5.07E-10 |
| CTC-484P3.3 | upregulated lncRNA | -2.629838448 | 5.09E-10 |
| XXbac-B476C20.14 | upregulated lncRNA | -2.407110935 | 5.34E-10 |
| WI2-80269A6.1 | upregulated lncRNA | -2.039177195 | 5.57E-10 |
| XXbac-BPG248L24.13 | upregulated lncRNA | -2.351911787 | 5.82E-10 |
| AC073321.4 | upregulated lncRNA | -2.163543183 | 6.09E-10 |
| RP11-406H4.1 | upregulated lncRNA | -2.264565611 | 6.23E-10 |
| ITCH-IT1 | upregulated lncRNA | -2.166345834 | 6.33E-10 |
| RP11-739N10.1 | upregulated lncRNA | -2.551370572 | 6.46E-10 |
| AC007078.4 | upregulated lncRNA | -2.565765472 | 6.47E-10 |
| RP11-428C19.5 | upregulated lncRNA | -2.18037635 | 6.87E-10 |
| CTB-37A13.1 | upregulated lncRNA | -2.474531132 | 6.95E-10 |
| MAFA-AS1 | upregulated lncRNA | -2.432608538 | 7.16E-10 |
| RP11-473C19.1 | upregulated lncRNA | -2.156412493 | 8.43E-10 |
| RP11-203E8.1 | upregulated lncRNA | -2.012625581 | 8.52E-10 |
| RP11-252K23.1 | upregulated lncRNA | -2.144568761 | 8.85E-10 |
| AC018685.1 | upregulated lncRNA | -2.569732505 | 8.86E-10 |
| RP11-494H4.3 | upregulated lncRNA | -2.050352331 | 9.57E-10 |
| RP13-631K18.3 | upregulated lncRNA | -2.221335411 | 9.60E-10 |
| RP11-272D12.2 | upregulated lncRNA | -2.02041492 | 1.05E-09 |
| AC078852.2 | upregulated lncRNA | -2.214317683 | 1.30E-09 |
| RP11-265D19.6 | upregulated lncRNA | -2.083511643 | 1.32E-09 |
| RP11-686G23.2 | upregulated lncRNA | -2.57066502 | 1.34E-09 |
| RP5-1069C8.2 | upregulated lncRNA | -2.038635492 | 1.41E-09 |
| RP11-22H5.2 | upregulated lncRNA | -2.260253149 | 1.43E-09 |
| AC092675.3 | upregulated lncRNA | -2.376585517 | 1.59E-09 |
| RP11-680F20.6 | upregulated lncRNA | -2.062754012 | 1.59E-09 |
| RP11-630D6.5 | upregulated lncRNA | -2.025230433 | 1.62E-09 |
| AC007193.6 | upregulated lncRNA | -2.775942853 | 1.63E-09 |
| RP5-951N9.1 | upregulated lncRNA | -2.248727935 | 1.71E-09 |
| CTA-363E6.1 | upregulated lncRNA | -2.063268286 | 1.73E-09 |
| XXbac-BPG27H4.8 | upregulated lncRNA | -2.646032811 | 1.91E-09 |
| RP11-386M24.9 | upregulated lncRNA | -2.005120507 | 1.95E-09 |
| RP11-116D2.1 | upregulated lncRNA | -3.101785787 | 1.97E-09 |
| RP11-96B2.1 | upregulated lncRNA | -3.577056629 | 1.98E-09 |
| CITF22-62D4.1 | upregulated lncRNA | -2.511276182 | 2.07E-09 |
| RP11-384C4.6 | upregulated lncRNA | -2.624511016 | 2.16E-09 |
| RP11-204N11.1 | upregulated lncRNA | -2.625668016 | 2.46E-09 |
| RP11-115H15.2 | upregulated lncRNA | -2.126504513 | 2.88E-09 |
| AP000289.6 | upregulated lncRNA | -2.046100656 | 2.92E-09 |
| RP11-1029J19.4 | upregulated lncRNA | -2.097709887 | 3.04E-09 |
| RP11-83C7.1 | upregulated lncRNA | -2.41647651 | 3.41E-09 |
| RP11-15M15.2 | upregulated lncRNA | -2.384868839 | 3.75E-09 |
| RP11-285E9.5 | upregulated lncRNA | -2.200259831 | 3.90E-09 |
| RP1-63G5.7 | upregulated lncRNA | -2.412819679 | 3.90E-09 |
| RP5-983L19.2 | upregulated lncRNA | -2.033520553 | 4.21E-09 |
| AC055764.1 | upregulated lncRNA | -2.188964529 | 4.93E-09 |
| RP11-481C4.1 | upregulated lncRNA | -2.125724466 | 5.48E-09 |
| LINC01192 | upregulated lncRNA | -2.935412776 | 5.60E-09 |
| RP11-673E11.2 | upregulated lncRNA | -2.330630864 | 5.85E-09 |
| RP11-883G14.3 | upregulated lncRNA | -2.350896503 | 6.70E-09 |
| RP11-414J4.2 | upregulated lncRNA | -2.192086566 | 6.71E-09 |
| XXyac-YX65C7_A.3 | upregulated lncRNA | -2.015417664 | 6.84E-09 |
| RP11-654A16.1 | upregulated lncRNA | -2.406565763 | 6.86E-09 |
| RP11-703M24.5 | upregulated lncRNA | -2.379274468 | 7.06E-09 |
| AC006145.4 | upregulated lncRNA | -2.545512031 | 7.10E-09 |
| RP11-91I20.4 | upregulated lncRNA | -2.552328439 | 7.43E-09 |
| SPATA13-AS1 | upregulated lncRNA | -2.065868298 | 7.44E-09 |
| RP11-428C19.4 | upregulated lncRNA | -2.124592364 | 8.21E-09 |
| LINC00221 | upregulated lncRNA | -2.482731681 | 8.57E-09 |
| GS1-278J22.2 | upregulated lncRNA | -2.545914417 | 8.57E-09 |
| FOXN3-AS2 | upregulated lncRNA | -2.64549988 | 9.71E-09 |
| RP4-604K5.3 | upregulated lncRNA | -2.031301433 | 1.02E-08 |
| RP11-753D20.3 | upregulated lncRNA | -2.02734214 | 1.03E-08 |
| RP1-228P16.4 | upregulated lncRNA | -2.307861809 | 1.07E-08 |
| LINC00856 | upregulated lncRNA | -2.00230775 | 1.08E-08 |
| RP11-178L8.5 | upregulated lncRNA | -2.615540535 | 1.11E-08 |
| AC105402.4 | upregulated lncRNA | -2.057753311 | 1.12E-08 |
| RP13-379O24.2 | upregulated lncRNA | -2.84670947 | 1.19E-08 |
| LINC01492 | upregulated lncRNA | -2.260129923 | 1.21E-08 |
| RP11-767N15.1 | upregulated lncRNA | -3.177863677 | 1.26E-08 |
| TGFA-IT1 | upregulated lncRNA | -2.219889906 | 1.31E-08 |
| RP11-1007G5.2 | upregulated lncRNA | -2.094680548 | 1.31E-08 |
| RP11-161D15.2 | upregulated lncRNA | -3.422382406 | 1.35E-08 |
| RP11-255M2.2 | upregulated lncRNA | -2.280308639 | 1.42E-08 |
| RMRP | upregulated lncRNA | -3.483000018 | 1.43E-08 |
| RP11-18F14.4 | upregulated lncRNA | -2.088144499 | 1.58E-08 |
| RC3H1-IT1 | upregulated lncRNA | -2.616364619 | 1.70E-08 |
| LINC00868 | upregulated lncRNA | -2.302540392 | 1.75E-08 |
| RP11-540O11.8 | upregulated lncRNA | -2.473560436 | 1.89E-08 |
| CTC-575I10.1 | upregulated lncRNA | -2.002701994 | 2.01E-08 |
| RP11-367J7.3 | upregulated lncRNA | -2.256163769 | 2.08E-08 |
| RP11-107I14.4 | upregulated lncRNA | -2.631558941 | 2.09E-08 |
| LINC01429 | upregulated lncRNA | -2.426292548 | 2.26E-08 |
| RP11-595B24.2 | upregulated lncRNA | -2.618352982 | 2.46E-08 |
| RPS6KA2-AS1 | upregulated lncRNA | -2.131033875 | 2.57E-08 |
| PTPRJ-AS1 | upregulated lncRNA | -2.273392336 | 2.68E-08 |
| GPC6-AS1 | upregulated lncRNA | -2.311079604 | 2.68E-08 |
| RP11-1000B6.2 | upregulated lncRNA | -2.013282638 | 2.89E-08 |
| RP11-10N16.2 | upregulated lncRNA | -2.088923837 | 2.91E-08 |
| RP11-47J17.3 | upregulated lncRNA | -2.369659692 | 2.91E-08 |
| RP11-642D21.2 | upregulated lncRNA | -2.048621519 | 2.92E-08 |
| CASC20 | upregulated lncRNA | -2.516728077 | 2.99E-08 |
| AP000997.2 | upregulated lncRNA | -2.362857624 | 2.99E-08 |
| DEPDC1-AS1 | upregulated lncRNA | -2.003324665 | 3.10E-08 |
| RP11-662I13.2 | upregulated lncRNA | -2.512920072 | 3.25E-08 |
| LINC00202-2 | upregulated lncRNA | -2.201845019 | 3.61E-08 |
| LINC00971 | upregulated lncRNA | -2.335922608 | 3.65E-08 |
| RP11-94A24.1 | upregulated lncRNA | -3.110042812 | 3.69E-08 |
| RP11-201E8.1 | upregulated lncRNA | -2.337800818 | 3.87E-08 |
| RP11-680B3.2 | upregulated lncRNA | -2.455332552 | 4.02E-08 |
| AC114877.3 | upregulated lncRNA | -2.41356033 | 4.21E-08 |
| RP4-536B24.4 | upregulated lncRNA | -2.098793815 | 4.26E-08 |
| CTD-2583P5.1 | upregulated lncRNA | -2.387542834 | 4.58E-08 |
| RP11-299P2.1 | upregulated lncRNA | -2.145174319 | 4.66E-08 |
| RP11-73G16.1 | upregulated lncRNA | -2.191994478 | 4.68E-08 |
| CH17-360D5.1 | upregulated lncRNA | -2.298627442 | 4.72E-08 |
| LINC01523 | upregulated lncRNA | -2.54215945 | 4.89E-08 |
| RP11-883G14.4 | upregulated lncRNA | -2.015623033 | 5.34E-08 |
| LINC00521 | upregulated lncRNA | -2.591111894 | 5.39E-08 |
| RP11-59O6.3 | upregulated lncRNA | -2.350545007 | 5.74E-08 |
| HMBOX1-IT1 | upregulated lncRNA | -2.147586188 | 5.86E-08 |
| RP11-408N14.1 | upregulated lncRNA | -2.116691134 | 6.89E-08 |
| AP000997.1 | upregulated lncRNA | -2.253793701 | 7.25E-08 |
| RP1-101D8.1 | upregulated lncRNA | -2.077828106 | 7.53E-08 |
| RP11-107N7.1 | upregulated lncRNA | -2.122827312 | 7.67E-08 |
| PCGEM1 | upregulated lncRNA | -2.392980303 | 7.78E-08 |
| CTD-2534I21.9 | upregulated lncRNA | -2.381545851 | 8.39E-08 |
| RP4-536B24.3 | upregulated lncRNA | -2.218658765 | 8.62E-08 |
| AF121898.3 | upregulated lncRNA | -2.251196537 | 8.88E-08 |
| RP11-383J24.1 | upregulated lncRNA | -2.314648417 | 9.67E-08 |
| RP1-101G11.3 | upregulated lncRNA | -2.115750187 | 9.89E-08 |
| TPRG1-AS2 | upregulated lncRNA | -2.134779533 | 1.14E-07 |
| MYO16-AS1 | upregulated lncRNA | -2.557507202 | 1.15E-07 |
| RP11-2L8.2 | upregulated lncRNA | -2.49076555 | 1.16E-07 |
| AC093642.4 | upregulated lncRNA | -2.007561197 | 1.24E-07 |
| RP11-3J1.1 | upregulated lncRNA | -2.574802009 | 1.36E-07 |
| LINC00710 | upregulated lncRNA | -2.447230225 | 1.36E-07 |
| LINC00524 | upregulated lncRNA | -2.970251174 | 1.38E-07 |
| RP11-238I10.1 | upregulated lncRNA | -2.117863986 | 1.44E-07 |
| CTD-2015A6.2 | upregulated lncRNA | -2.153894667 | 1.49E-07 |
| RP3-495K2.3 | upregulated lncRNA | -3.267617964 | 1.53E-07 |
| RP11-456I15.2 | upregulated lncRNA | -2.088691372 | 1.62E-07 |
| RP11-774D14.1 | upregulated lncRNA | -2.522816286 | 1.74E-07 |
| AC004603.4 | upregulated lncRNA | -2.796135024 | 1.76E-07 |
| RP5-859D4.3 | upregulated lncRNA | -2.29240284 | 1.83E-07 |
| RP4-564F22.6 | upregulated lncRNA | -3.356779718 | 1.85E-07 |
| RP11-772C9.1 | upregulated lncRNA | -2.480501687 | 1.86E-07 |
| RP11-399F2.2 | upregulated lncRNA | -2.086529806 | 1.90E-07 |
| RP11-702F3.1 | upregulated lncRNA | -2.010647274 | 1.96E-07 |
| LINC00547 | upregulated lncRNA | -2.239151793 | 1.96E-07 |
| AC008271.1 | upregulated lncRNA | -2.325570628 | 1.97E-07 |
| TRIM36-IT1 | upregulated lncRNA | -2.402393845 | 1.99E-07 |
| RP11-1H15.2 | upregulated lncRNA | -2.307074875 | 2.16E-07 |
| CTD-2560E9.5 | upregulated lncRNA | -2.008365225 | 2.18E-07 |
| RP11-866E20.3 | upregulated lncRNA | -2.250223187 | 2.31E-07 |
| RP5-1185I7.1 | upregulated lncRNA | -2.109995552 | 2.42E-07 |
| RP11-70O5.2 | upregulated lncRNA | -2.105196377 | 2.48E-07 |
| AC133785.1 | upregulated lncRNA | -2.623858393 | 2.85E-07 |
| RP11-349K16.1 | upregulated lncRNA | -2.881249172 | 2.86E-07 |
| BPESC1 | upregulated lncRNA | -2.519778619 | 2.88E-07 |
| RP11-183I6.2 | upregulated lncRNA | -2.039802706 | 2.89E-07 |
| RP11-64P14.7 | upregulated lncRNA | -2.226267094 | 2.93E-07 |
| LINC01304 | upregulated lncRNA | -2.496567194 | 3.39E-07 |
| RP11-744D14.2 | upregulated lncRNA | -2.583149489 | 3.39E-07 |
| LINC00895 | upregulated lncRNA | -2.113668369 | 3.55E-07 |
| FLJ26245 | upregulated lncRNA | -2.241663565 | 3.60E-07 |
| C12orf77 | upregulated lncRNA | -2.295152337 | 3.62E-07 |
| AC128709.3 | upregulated lncRNA | -2.074379729 | 3.62E-07 |
| LINC01151 | upregulated lncRNA | -2.112749433 | 3.63E-07 |
| LATS2-AS1 | upregulated lncRNA | -2.156128822 | 3.70E-07 |
| RP11-550H2.1 | upregulated lncRNA | -2.405636808 | 3.74E-07 |
| RP11-263E1.1 | upregulated lncRNA | -2.301550509 | 4.25E-07 |
| RP11-115C10.1 | upregulated lncRNA | -2.146463168 | 4.37E-07 |
| AP003025.2 | upregulated lncRNA | -2.234730622 | 4.76E-07 |
| BRWD1-IT1 | upregulated lncRNA | -2.233914393 | 5.38E-07 |
| CTD-2291D10.3 | upregulated lncRNA | -2.745132187 | 5.73E-07 |
| CTD-2587H24.5 | upregulated lncRNA | -2.177073981 | 6.19E-07 |
| LA16c-381G6.1 | upregulated lncRNA | -2.268908548 | 6.43E-07 |
| CTD-2105E13.16 | upregulated lncRNA | -2.062572718 | 6.61E-07 |
| RP11-8L2.1 | upregulated lncRNA | -2.479493745 | 6.84E-07 |
| AC011286.1 | upregulated lncRNA | -2.5824342 | 7.43E-07 |
| RP11-161D15.3 | upregulated lncRNA | -3.034655129 | 8.08E-07 |
| RP11-609N14.1 | upregulated lncRNA | -2.067677181 | 8.39E-07 |
| CTB-31N19.5 | upregulated lncRNA | -2.061804972 | 8.43E-07 |
| RP11-338L22.2 | upregulated lncRNA | -2.092102328 | 8.57E-07 |
| CTD-3023L14.2 | upregulated lncRNA | -2.100938687 | 8.64E-07 |
| RP11-392B6.1 | upregulated lncRNA | -2.086972733 | 8.85E-07 |
| DKFZP434L187 | upregulated lncRNA | -2.287934266 | 9.04E-07 |
| PCAT2 | upregulated lncRNA | -2.241248647 | 9.66E-07 |
| RP11-344E13.4 | upregulated lncRNA | -2.143146284 | 1.05E-06 |
| LINC00922 | upregulated lncRNA | -2.299802461 | 1.08E-06 |
| AC003092.2 | upregulated lncRNA | -2.395327237 | 1.10E-06 |
| RP11-63G10.2 | upregulated lncRNA | -2.103730189 | 1.10E-06 |
| CTD-2236F14.1 | upregulated lncRNA | -2.032099968 | 1.11E-06 |
| RP11-751H17.1 | upregulated lncRNA | -2.841599458 | 1.11E-06 |
| RP11-311F12.2 | upregulated lncRNA | -2.138371841 | 1.15E-06 |
| AC073257.1 | upregulated lncRNA | -2.002590205 | 1.33E-06 |
| RP11-497D6.5 | upregulated lncRNA | -2.236165329 | 1.42E-06 |
| RP11-327I22.5 | upregulated lncRNA | -2.424124066 | 1.44E-06 |
| RP11-442J21.2 | upregulated lncRNA | -2.373963043 | 1.50E-06 |
| RP11-691H4.3 | upregulated lncRNA | -2.402446505 | 1.54E-06 |
| RP11-66H6.3 | upregulated lncRNA | -2.021650668 | 1.55E-06 |
| RP11-543N12.1 | upregulated lncRNA | -2.010938241 | 1.63E-06 |
| RP11-20D14.3 | upregulated lncRNA | -2.009803772 | 1.64E-06 |
| RP11-401F2.4 | upregulated lncRNA | -2.283046461 | 1.73E-06 |
| RP11-702L15.4 | upregulated lncRNA | -2.057282616 | 1.76E-06 |
| RP11-172E10.1 | upregulated lncRNA | -2.134150512 | 1.77E-06 |
| RP11-143A12.3 | upregulated lncRNA | -2.075164199 | 1.78E-06 |
| LINC01440 | upregulated lncRNA | -2.147876458 | 1.84E-06 |
| AC124997.1 | upregulated lncRNA | -2.001626566 | 1.85E-06 |
| AC092484.1 | upregulated lncRNA | -3.114853529 | 1.94E-06 |
| RP11-242P2.2 | upregulated lncRNA | -2.216291332 | 2.07E-06 |
| LINC00477 | upregulated lncRNA | -2.109450305 | 2.09E-06 |
| C17orf77 | upregulated lncRNA | -2.374523025 | 2.14E-06 |
| RP11-255M2.1 | upregulated lncRNA | -2.075911815 | 2.26E-06 |
| RP11-350G24.1 | upregulated lncRNA | -2.541723042 | 2.36E-06 |
| RP11-2N5.2 | upregulated lncRNA | -2.15110464 | 2.79E-06 |
| RP4-719C8.1 | upregulated lncRNA | -2.64416723 | 2.94E-06 |
| RP11-58G13.1 | upregulated lncRNA | -2.598028062 | 2.94E-06 |
| RP11-789C2.1 | upregulated lncRNA | -2.310205531 | 3.12E-06 |
| AC009518.4 | upregulated lncRNA | -2.176747051 | 3.27E-06 |
| RP11-85O21.5 | upregulated lncRNA | -2.042820723 | 3.40E-06 |
| AP000146.2 | upregulated lncRNA | -2.037146258 | 3.67E-06 |
| RP4-566L20.1 | upregulated lncRNA | -2.432013246 | 3.81E-06 |
| RP11-133L19.3 | upregulated lncRNA | -2.819719544 | 4.12E-06 |
| LINC00355 | upregulated lncRNA | -2.675933507 | 4.16E-06 |
| CTD-2330K9.2 | upregulated lncRNA | -2.200778352 | 4.30E-06 |
| RP11-267A15.3 | upregulated lncRNA | -2.312123496 | 4.62E-06 |
| RP11-122C21.1 | upregulated lncRNA | -2.264564291 | 4.83E-06 |
| AC008268.1 | upregulated lncRNA | -2.765196423 | 4.97E-06 |
| RP11-454P21.1 | upregulated lncRNA | -2.819217471 | 5.09E-06 |
| RP11-67M9.1 | upregulated lncRNA | -2.45065701 | 6.36E-06 |
| CTB-78F1.1 | upregulated lncRNA | -2.149097242 | 7.43E-06 |
| CTB-118P15.2 | upregulated lncRNA | -2.05426065 | 7.62E-06 |
| CTA-125H2.2 | upregulated lncRNA | -2.222911542 | 7.96E-06 |
| RP11-737F9.1 | upregulated lncRNA | -2.033933422 | 8.74E-06 |
| RP11-100E13.1 | upregulated lncRNA | -2.119156239 | 8.95E-06 |
| CH17-360D5.3 | upregulated lncRNA | -2.675773353 | 9.38E-06 |
| AC104777.2 | upregulated lncRNA | -2.438775941 | 9.60E-06 |
| RP1-209A6.1 | upregulated lncRNA | -2.274823688 | 9.88E-06 |
| RP11-313P18.1 | upregulated lncRNA | -2.260843545 | 1.13E-05 |
| CTA-221G9.11 | upregulated lncRNA | -2.022499479 | 1.15E-05 |
| RP4-668E10.4 | upregulated lncRNA | -2.235959631 | 1.20E-05 |
| RP3-462C17.1 | upregulated lncRNA | -2.091553534 | 1.31E-05 |
| GS1-278J22.1 | upregulated lncRNA | -2.054279117 | 1.35E-05 |
| RP11-553K8.5 | upregulated lncRNA | -2.132028508 | 1.36E-05 |
| CTD-2010I22.2 | upregulated lncRNA | -2.315933676 | 1.40E-05 |
| RP11-514D23.1 | upregulated lncRNA | -2.045791407 | 1.55E-05 |
| KCNIP4-IT1 | upregulated lncRNA | -2.223163717 | 1.55E-05 |
| RP11-543H12.1 | upregulated lncRNA | -2.161753386 | 1.56E-05 |
| AC008067.2 | upregulated lncRNA | -2.19675836 | 1.89E-05 |
| LINC00678 | upregulated lncRNA | -2.38942621 | 1.95E-05 |
| LINC01067 | upregulated lncRNA | -2.230264116 | 1.98E-05 |
| RP11-964E11.2 | upregulated lncRNA | -2.237012001 | 2.15E-05 |
| RP11-567E21.3 | upregulated lncRNA | -3.231009401 | 2.33E-05 |
| TCF4-AS2 | upregulated lncRNA | -2.083786041 | 2.33E-05 |
| LINC01048 | upregulated lncRNA | -2.069409749 | 2.58E-05 |
| CDRT7 | upregulated lncRNA | -2.24227133 | 2.96E-05 |
| RP11-307L3.4 | upregulated lncRNA | -2.070635925 | 2.97E-05 |
| CTC-379B2.4 | upregulated lncRNA | -2.016154 | 3.03E-05 |
| CTB-61M7.1 | upregulated lncRNA | -2.097597973 | 3.34E-05 |
| RP11-608O21.1 | upregulated lncRNA | -2.855116161 | 3.57E-05 |
| RP11-115D19.2 | upregulated lncRNA | -2.247338507 | 3.60E-05 |
| RP11-605F22.1 | upregulated lncRNA | -3.250978588 | 5.66E-05 |
| TMEM108-AS1 | upregulated lncRNA | -2.26167506 | 6.52E-05 |
| RP11-711K1.7 | upregulated lncRNA | -2.304982387 | 6.53E-05 |
| RP11-1029J19.2 | upregulated lncRNA | -2.603365882 | 6.83E-05 |
| LINC00879 | upregulated lncRNA | -3.374948408 | 7.12E-05 |
| RP11-313P18.2 | upregulated lncRNA | -2.802808569 | 7.45E-05 |
| RP11-115D19.3 | upregulated lncRNA | -2.257773984 | 7.47E-05 |
| LINC00397 | upregulated lncRNA | -2.161817475 | 7.78E-05 |
| RP11-260O18.1 | upregulated lncRNA | -2.0000001 | 8.59E-05 |
| AC093063.3 | upregulated lncRNA | -2.085604067 | 9.60E-05 |
| LINC00836 | upregulated lncRNA | -2.14095441 | 9.95E-05 |
| AC011524.2 | upregulated lncRNA | -2.073594337 | 0.000111718 |
| RP11-138H10.2 | upregulated lncRNA | -2.483206495 | 0.000122507 |
| RP11-713N11.5 | upregulated lncRNA | -2.46690749 | 0.000131839 |
| RP1-170O19.17 | upregulated lncRNA | -2.000620627 | 0.000177989 |
| AP000459.7 | upregulated lncRNA | -2.102674555 | 0.000187497 |
| RP11-1263C18.1 | upregulated lncRNA | -2.14442854 | 0.000207599 |
| AC011524.3 | upregulated lncRNA | -2.126181704 | 0.000207636 |
| RP11-554D15.3 | upregulated lncRNA | -2.061112367 | 0.000304138 |
| RP11-35J23.1 | upregulated lncRNA | -2.615038561 | 0.000390302 |
| AC079154.1 | upregulated lncRNA | -2.169006555 | 0.000416209 |
| NRG3-AS1 | upregulated lncRNA | -2.514069334 | 0.00049968 |
| RP11-352D3.2 | upregulated lncRNA | -2.00194702 | 0.00050007 |
| RP11-89M20.2 | upregulated lncRNA | -2.771692608 | 0.000623411 |
| CTD-2265O21.3 | upregulated lncRNA | -2.129805841 | 0.000631083 |
| RP11-202D18.2 | upregulated lncRNA | -2.708106293 | 0.000934377 |
| BARX1-AS1 | upregulated lncRNA | -2.007059727 | 0.001492001 |
| RP11-138J23.1 | upregulated lncRNA | -2.083875535 | 0.001775127 |
| AC092635.1 | upregulated lncRNA | -2.341419669 | 0.002360301 |
| RP11-217E22.5 | upregulated lncRNA | -2.069201971 | 0.002748986 |
| CH507-513H4.3 | upregulated lncRNA | -2.230280758 | 0.003047296 |
| KCNQ5-IT1 | upregulated lncRNA | -2.311109358 | 0.003207201 |
| AC131056.5 | upregulated lncRNA | -2.585578539 | 0.004247847 |
| LINC00588 | upregulated lncRNA | -2.598690302 | 0.004823032 |
| RP5-1070A16.1 | upregulated lncRNA | -2.440653124 | 0.006016338 |
| RP11-955H22.1 | upregulated lncRNA | -2.004110588 | 0.006894694 |
| RP11-554D15.4 | upregulated lncRNA | -2.423570209 | 0.0084647 |
| LINC00675 | downregulated lncRNA | 7.142884027 | 2.30E-279 |
| AC074286.1 | downregulated lncRNA | 3.735864985 | 4.77E-274 |
| RP11-61L19.2 | downregulated lncRNA | 6.607276801 | 9.30E-236 |
| RP5-1018K9.1 | downregulated lncRNA | 5.49374071 | 2.93E-159 |
| RP11-834C11.5 | downregulated lncRNA | 3.468324053 | 4.39E-155 |
| RP4-655J12.4 | downregulated lncRNA | 4.649174075 | 6.52E-150 |
| RP11-536G4.1 | downregulated lncRNA | 4.864403038 | 1.54E-142 |
| AP000696.2 | downregulated lncRNA | 5.825717946 | 5.21E-139 |
| RP11-390N6.1 | downregulated lncRNA | 7.740840229 | 4.16E-134 |
| RP11-116O18.1 | downregulated lncRNA | 6.585075889 | 2.52E-123 |
| LINC01378 | downregulated lncRNA | 6.534844022 | 1.04E-118 |
| RP11-527L4.6 | downregulated lncRNA | 6.060003637 | 1.65E-117 |
| RP1-56K13.5 | downregulated lncRNA | 4.586224902 | 1.27E-116 |
| RP11-195B3.1 | downregulated lncRNA | 6.665770525 | 3.22E-114 |
| RP3-466P17.1 | downregulated lncRNA | 2.400293438 | 3.72E-114 |
| RP11-89B16.1 | downregulated lncRNA | 4.760093961 | 1.30E-113 |
| RP11-573D15.8 | downregulated lncRNA | 4.677730147 | 7.35E-112 |
| LINC01020 | downregulated lncRNA | 6.480969456 | 7.56E-112 |
| RP11-180C16.1 | downregulated lncRNA | 3.062610086 | 7.96E-109 |
| RP11-531H8.2 | downregulated lncRNA | 7.027320715 | 1.91E-108 |
| RP11-363J20.1 | downregulated lncRNA | 2.683413822 | 6.78E-108 |
| LINC00371 | downregulated lncRNA | 5.388830821 | 1.84E-106 |
| RP11-18H21.3 | downregulated lncRNA | 4.803939991 | 2.42E-105 |
| AC003090.1 | downregulated lncRNA | 4.001437647 | 4.06E-104 |
| RP11-208K4.1 | downregulated lncRNA | 7.096003088 | 1.97E-100 |
| RP11-142A12.1 | downregulated lncRNA | 6.695727195 | 2.24E-100 |
| WSPAR | downregulated lncRNA | 4.735991721 | 1.35E-98 |
| LINC01571 | downregulated lncRNA | 6.957966004 | 3.87E-98 |
| FAM215A | downregulated lncRNA | 3.578462787 | 3.02E-96 |
| RP11-321G12.1 | downregulated lncRNA | 3.157712678 | 4.96E-96 |
| RP4-735C1.4 | downregulated lncRNA | 3.715290669 | 1.38E-94 |
| RP11-386B13.4 | downregulated lncRNA | 8.057413166 | 2.07E-94 |
| RP11-999E24.3 | downregulated lncRNA | 3.564003531 | 2.38E-93 |
| RP11-752D24.2 | downregulated lncRNA | 5.750627505 | 5.26E-93 |
| RP11-128L5.1 | downregulated lncRNA | 3.80345931 | 6.54E-92 |
| RP11-714L20.1 | downregulated lncRNA | 4.670714271 | 9.44E-92 |
| RP11-482D24.3 | downregulated lncRNA | 2.667719953 | 5.96E-91 |
| RP11-295M3.4 | downregulated lncRNA | 3.949191602 | 6.34E-91 |
| LYPLAL1-AS1 | downregulated lncRNA | 2.356479578 | 9.73E-91 |
| SLC25A5-AS1 | downregulated lncRNA | 2.01217329 | 3.46E-90 |
| RP11-317M11.1 | downregulated lncRNA | 6.996496395 | 1.93E-88 |
| FOXCUT | downregulated lncRNA | 3.892544597 | 3.86E-87 |
| AC096574.5 | downregulated lncRNA | 2.81178504 | 1.20E-86 |
| RP11-94C24.13 | downregulated lncRNA | 3.287852948 | 3.47E-86 |
| RP11-536C5.2 | downregulated lncRNA | 3.406742111 | 4.11E-86 |
| RP1-168L15.5 | downregulated lncRNA | 2.630163749 | 1.27E-85 |
| VWA8-AS1 | downregulated lncRNA | 2.512376265 | 2.02E-85 |
| RP11-516J2.1 | downregulated lncRNA | 7.019932466 | 3.30E-85 |
| RP13-650J16.1 | downregulated lncRNA | 4.118364148 | 2.32E-84 |
| CTD-2007H18.1 | downregulated lncRNA | 5.875976449 | 4.43E-84 |
| LINC01055 | downregulated lncRNA | 6.3550867 | 1.42E-82 |
| RP11-90P13.1 | downregulated lncRNA | 3.793528047 | 5.67E-82 |
| LINC01555 | downregulated lncRNA | 4.893793657 | 2.45E-81 |
| LINC00443 | downregulated lncRNA | 3.326126328 | 1.86E-79 |
| CTB-79E8.2 | downregulated lncRNA | 4.200986788 | 3.81E-79 |
| LINC00652 | downregulated lncRNA | 2.181446581 | 4.39E-79 |
| RP11-131N11.4 | downregulated lncRNA | 3.552950909 | 6.03E-79 |
| RP1-137K24.1 | downregulated lncRNA | 5.494493171 | 1.01E-78 |
| LINC00551 | downregulated lncRNA | 3.850919082 | 2.10E-75 |
| PLS3-AS1 | downregulated lncRNA | 2.807356072 | 1.17E-73 |
| RP11-480D4.2 | downregulated lncRNA | 2.52460636 | 3.13E-73 |
| RP11-245J24.1 | downregulated lncRNA | 5.791631484 | 3.22E-73 |
| TARID | downregulated lncRNA | 2.878690987 | 1.61E-72 |
| RP11-850F7.7 | downregulated lncRNA | 8.4420794 | 8.82E-72 |
| LINC01543 | downregulated lncRNA | 6.386157287 | 1.05E-71 |
| AC012123.1 | downregulated lncRNA | 3.905094493 | 8.84E-71 |
| LINC01159 | downregulated lncRNA | 2.356463232 | 1.02E-69 |
| RP11-643M14.1 | downregulated lncRNA | 2.235460805 | 1.36E-68 |
| RP11-211N11.5 | downregulated lncRNA | 2.881924475 | 3.91E-68 |
| PP7080 | downregulated lncRNA | 2.438738786 | 1.88E-67 |
| LINC00982 | downregulated lncRNA | 5.141986163 | 2.66E-65 |
| RP11-451B8.1 | downregulated lncRNA | 3.310946292 | 3.59E-65 |
| AC005616.2 | downregulated lncRNA | 6.416156424 | 3.57E-64 |
| RP11-816J8.1 | downregulated lncRNA | 3.867149369 | 2.19E-63 |
| RP11-527L4.2 | downregulated lncRNA | 5.58141308 | 2.76E-63 |
| F11-AS1 | downregulated lncRNA | 4.307318809 | 7.88E-63 |
| AC010884.1 | downregulated lncRNA | 2.827949253 | 2.66E-61 |
| HNF4A-AS1 | downregulated lncRNA | 4.61904575 | 2.89E-60 |
| RP11-834C11.4 | downregulated lncRNA | 2.218571817 | 4.69E-60 |
| RP11-575B7.3 | downregulated lncRNA | 6.202080923 | 8.68E-60 |
| RP11-61O1.1 | downregulated lncRNA | 3.685938638 | 1.43E-59 |
| RP11-528A4.2 | downregulated lncRNA | 3.37432349 | 2.93E-59 |
| AC006960.7 | downregulated lncRNA | 5.117215855 | 3.77E-59 |
| RP11-25G10.2 | downregulated lncRNA | 2.277756697 | 8.68E-59 |
| RP11-494M8.4 | downregulated lncRNA | 3.831416486 | 2.67E-57 |
| RP11-77I22.2 | downregulated lncRNA | 2.969975844 | 6.52E-57 |
| RP11-480D4.6 | downregulated lncRNA | 2.182968162 | 4.31E-56 |
| RP11-470M17.2 | downregulated lncRNA | 3.767249739 | 5.74E-55 |
| RP11-2I17.4 | downregulated lncRNA | 2.105404362 | 2.28E-53 |
| TINCR | downregulated lncRNA | 2.489777008 | 1.44E-52 |
| AC068138.1 | downregulated lncRNA | 6.561767459 | 2.60E-52 |
| RP4-655J12.5 | downregulated lncRNA | 3.201187895 | 2.63E-52 |
| RP11-511B23.2 | downregulated lncRNA | 2.215805594 | 4.76E-52 |
| RP11-550H2.2 | downregulated lncRNA | 5.323348547 | 7.59E-52 |
| RRS1-AS1 | downregulated lncRNA | 2.893463386 | 8.95E-52 |
| GATA3-AS1 | downregulated lncRNA | 5.291426586 | 1.45E-51 |
| RP11-545A16.1 | downregulated lncRNA | 4.562324596 | 1.48E-51 |
| AC007255.8 | downregulated lncRNA | 2.469250362 | 3.39E-51 |
| LINC00472 | downregulated lncRNA | 2.047534106 | 5.08E-51 |
| LINC00379 | downregulated lncRNA | 4.396308129 | 7.46E-51 |
| RP11-554A11.9 | downregulated lncRNA | 2.622845019 | 1.25E-50 |
| RP11-456H18.2 | downregulated lncRNA | 2.249040087 | 2.24E-50 |
| RP11-254F7.3 | downregulated lncRNA | 2.235589679 | 2.32E-50 |
| SPATA13 | downregulated lncRNA | 2.424093183 | 3.79E-50 |
| RP11-25E2.1 | downregulated lncRNA | 3.469421828 | 9.05E-50 |
| RP11-475O23.2 | downregulated lncRNA | 3.11803057 | 1.50E-49 |
| RP1-78O14.1 | downregulated lncRNA | 2.948038511 | 5.93E-49 |
| LINC00845 | downregulated lncRNA | 3.296818193 | 7.48E-49 |
| RP11-74M11.2 | downregulated lncRNA | 3.452593416 | 5.61E-48 |
| KB-1562D12.1 | downregulated lncRNA | 4.961807576 | 2.87E-47 |
| GAS1RR | downregulated lncRNA | 2.134546167 | 8.10E-47 |
| RP11-579D7.4 | downregulated lncRNA | 2.157915215 | 1.09E-46 |
| RP11-66B24.5 | downregulated lncRNA | 2.938312756 | 1.90E-46 |
| AC124944.5 | downregulated lncRNA | 6.196193367 | 2.26E-46 |
| AC144831.1 | downregulated lncRNA | 2.513804915 | 2.41E-46 |
| AC144831.3 | downregulated lncRNA | 2.528914244 | 6.52E-46 |
| RP11-362F19.1 | downregulated lncRNA | 2.870554938 | 1.29E-45 |
| CTC-340D7.1 | downregulated lncRNA | 3.337653717 | 2.61E-45 |
| RP4-547N15.3 | downregulated lncRNA | 3.82633862 | 6.01E-45 |
| CTD-2247C11.5 | downregulated lncRNA | 8.189487351 | 1.02E-44 |
| CTD-2515H24.2 | downregulated lncRNA | 2.44183349 | 2.99E-44 |
| RP1-38C16.2 | downregulated lncRNA | 4.722372083 | 1.02E-43 |
| RP11-536G4.2 | downregulated lncRNA | 2.56255457 | 2.35E-43 |
| RP11-370F5.4 | downregulated lncRNA | 2.745999357 | 2.91E-43 |
| AC016735.2 | downregulated lncRNA | 3.17487263 | 3.71E-43 |
| CLDN10-AS1 | downregulated lncRNA | 2.758690744 | 5.09E-43 |
| RP11-482D24.2 | downregulated lncRNA | 2.362186095 | 6.39E-43 |
| RP11-311F12.1 | downregulated lncRNA | 2.533314993 | 3.24E-42 |
| RP1-206D15.6 | downregulated lncRNA | 2.43146605 | 3.88E-42 |
| RP11-10L7.1 | downregulated lncRNA | 2.450718969 | 8.98E-42 |
| RP4-646N3.1 | downregulated lncRNA | 2.005677289 | 1.37E-41 |
| CTD-2227I18.1 | downregulated lncRNA | 3.004531396 | 1.80E-41 |
| AE000662.93 | downregulated lncRNA | 2.999821785 | 2.33E-41 |
| RP11-392O17.1 | downregulated lncRNA | 3.026094104 | 4.27E-41 |
| AP000697.6 | downregulated lncRNA | 4.977217293 | 1.08E-40 |
| RP11-359N11.1 | downregulated lncRNA | 4.108695787 | 1.10E-40 |
| RP11-168K11.3 | downregulated lncRNA | 2.125606841 | 1.50E-40 |
| LINC00864 | downregulated lncRNA | 3.628953272 | 2.64E-40 |
| AC022431.3 | downregulated lncRNA | 2.84697608 | 3.22E-40 |
| RP11-554A11.4 | downregulated lncRNA | 2.825779198 | 6.23E-40 |
| RP3-380B4.1 | downregulated lncRNA | 3.653613837 | 8.69E-40 |
| RP11-21A7A.2 | downregulated lncRNA | 4.153282377 | 1.09E-39 |
| AC107057.1 | downregulated lncRNA | 6.305805619 | 1.82E-39 |
| BMPR1B-AS1 | downregulated lncRNA | 4.980911046 | 2.06E-39 |
| BVES-AS1 | downregulated lncRNA | 2.167338169 | 2.50E-39 |
| RP11-689K5.3 | downregulated lncRNA | 3.190163825 | 2.70E-39 |
| RP11-401P9.4 | downregulated lncRNA | 2.724987705 | 3.91E-39 |
| RP3-429O6.1 | downregulated lncRNA | 4.793922679 | 7.02E-39 |
| FAM167A-AS1 | downregulated lncRNA | 3.869358712 | 1.65E-38 |
| LINC00380 | downregulated lncRNA | 3.378599861 | 2.40E-38 |
| PROX1-AS1 | downregulated lncRNA | 3.751309881 | 4.23E-38 |
| AC105398.3 | downregulated lncRNA | 5.173348231 | 4.97E-38 |
| RP11-879F14.1 | downregulated lncRNA | 3.088541731 | 7.33E-38 |
| SRGAP3-AS4 | downregulated lncRNA | 3.673582246 | 1.56E-37 |
| RP11-433J22.3 | downregulated lncRNA | 3.436130197 | 2.20E-37 |
| AC013460.1 | downregulated lncRNA | 2.230162779 | 4.58E-37 |
| RP11-63A11.1 | downregulated lncRNA | 4.659462741 | 6.51E-37 |
| RP11-22C11.2 | downregulated lncRNA | 4.145162107 | 1.01E-36 |
| DLG1-AS1 | downregulated lncRNA | 2.2645003 | 1.53E-36 |
| RP11-21A7A.3 | downregulated lncRNA | 3.843752837 | 3.41E-36 |
| RP11-725G5.2 | downregulated lncRNA | 2.352958165 | 3.87E-36 |
| RP11-728F11.4 | downregulated lncRNA | 2.814706719 | 4.75E-36 |
| AL161668.5 | downregulated lncRNA | 3.70487669 | 1.29E-35 |
| RP11-310P5.2 | downregulated lncRNA | 5.100863673 | 1.29E-35 |
| AC124861.1 | downregulated lncRNA | 3.005185356 | 1.33E-35 |
| RP11-460I13.2 | downregulated lncRNA | 2.266681926 | 1.81E-35 |
| RP11-393N21.2 | downregulated lncRNA | 3.010238036 | 2.04E-35 |
| RP11-457K10.1 | downregulated lncRNA | 4.174803501 | 2.07E-35 |
| RP11-95P13.1 | downregulated lncRNA | 2.346079686 | 2.60E-35 |
| AP000345.1 | downregulated lncRNA | 3.173657994 | 2.63E-35 |
| RP11-366L20.2 | downregulated lncRNA | 3.217798249 | 4.44E-35 |
| RP11-467P9.1 | downregulated lncRNA | 2.01448442 | 4.46E-35 |
| LINC01317 | downregulated lncRNA | 2.316072951 | 8.86E-35 |
| LINC01031 | downregulated lncRNA | 2.456503534 | 1.02E-34 |
| RP11-671P2.1 | downregulated lncRNA | 2.627022763 | 1.74E-34 |
| PTCSC3 | downregulated lncRNA | 3.597543993 | 5.44E-34 |
| RP1-10C16.1 | downregulated lncRNA | 2.043134318 | 5.80E-34 |
| RP11-297L17.2 | downregulated lncRNA | 2.451905893 | 6.84E-34 |
| COL18A1-AS1 | downregulated lncRNA | 2.477567245 | 8.86E-34 |
| RP11-360O19.4 | downregulated lncRNA | 3.088047762 | 1.34E-33 |
| CTC-498J12.1 | downregulated lncRNA | 2.410920283 | 4.44E-33 |
| RP11-108E14.1 | downregulated lncRNA | 3.608228353 | 4.81E-33 |
| AC005082.12 | downregulated lncRNA | 2.941271851 | 5.97E-33 |
| RP11-586L23.1 | downregulated lncRNA | 6.108200831 | 9.39E-33 |
| RP11-113O24.3 | downregulated lncRNA | 3.450663538 | 1.01E-32 |
| RP11-141O11.2 | downregulated lncRNA | 2.04528606 | 1.33E-32 |
| RP11-476M19.2 | downregulated lncRNA | 3.615118417 | 4.93E-32 |
| RP11-449J21.5 | downregulated lncRNA | 2.213438499 | 5.21E-32 |
| SCHLAP1 | downregulated lncRNA | 4.180968307 | 6.98E-32 |
| LMO7-AS1 | downregulated lncRNA | 2.324691889 | 1.48E-31 |
| RP11-35J10.7 | downregulated lncRNA | 4.565750866 | 1.91E-31 |
| IFT74-AS1 | downregulated lncRNA | 2.087743632 | 1.94E-31 |
| RP11-285G1.9 | downregulated lncRNA | 2.201703905 | 3.11E-31 |
| AC103563.8 | downregulated lncRNA | 2.982014642 | 4.51E-31 |
| RP4-794I6.4 | downregulated lncRNA | 2.002663074 | 7.53E-31 |
| TSSC1-IT1 | downregulated lncRNA | 2.364603574 | 1.09E-30 |
| AC013463.2 | downregulated lncRNA | 2.64677029 | 1.29E-30 |
| RP11-31F19.1 | downregulated lncRNA | 2.729940023 | 2.56E-30 |
| FAM3D-AS1 | downregulated lncRNA | 2.655719091 | 2.67E-30 |
| RP4-753M9.1 | downregulated lncRNA | 3.265289177 | 2.70E-30 |
| RP11-563M4.1 | downregulated lncRNA | 2.972854343 | 5.00E-30 |
| AC005301.8 | downregulated lncRNA | 4.819824457 | 6.46E-30 |
| MYCNOS | downregulated lncRNA | 2.569898279 | 6.70E-30 |
| RP11-567M16.2 | downregulated lncRNA | 3.219583897 | 7.76E-30 |
| RP11-622A1.2 | downregulated lncRNA | 3.854059606 | 8.37E-30 |
| SSTR5-AS1 | downregulated lncRNA | 3.790465414 | 1.99E-29 |
| RP1-80N2.2 | downregulated lncRNA | 3.296696568 | 2.68E-29 |
| HOXB-AS3 | downregulated lncRNA | 2.459999233 | 3.06E-29 |
| RP11-238K6.1 | downregulated lncRNA | 2.964671259 | 3.69E-29 |
| RP11-12L8.1 | downregulated lncRNA | 2.481756783 | 4.60E-29 |
| RP11-415C15.1 | downregulated lncRNA | 4.067065971 | 7.25E-29 |
| RP11-794G24.1 | downregulated lncRNA | 3.102724637 | 1.03E-28 |
| RP11-486L19.2 | downregulated lncRNA | 2.790416952 | 2.38E-28 |
| RP11-2E11.5 | downregulated lncRNA | 2.526227928 | 2.38E-28 |
| CTD-2004A9.1 | downregulated lncRNA | 3.387722022 | 2.47E-28 |
| SEMA3B-AS1 | downregulated lncRNA | 2.67425059 | 3.61E-28 |
| RP4-737E23.2 | downregulated lncRNA | 2.775608066 | 5.39E-28 |
| CTA-392C11.1 | downregulated lncRNA | 5.45470593 | 5.47E-28 |
| RP11-14K3.7 | downregulated lncRNA | 4.979755205 | 6.55E-28 |
| RP13-259N13.2 | downregulated lncRNA | 5.386288912 | 7.63E-28 |
| LINC00645 | downregulated lncRNA | 4.145834267 | 8.05E-28 |
| CTD-3162L10.3 | downregulated lncRNA | 2.036630615 | 8.17E-28 |
| AC026471.6 | downregulated lncRNA | 2.47468585 | 1.01E-27 |
| RP13-616I3.1 | downregulated lncRNA | 2.29345351 | 1.35E-27 |
| AC019181.2 | downregulated lncRNA | 3.265717793 | 1.44E-27 |
| LINC01589 | downregulated lncRNA | 2.526574031 | 3.45E-27 |
| RP11-881M11.1 | downregulated lncRNA | 2.547346871 | 8.43E-27 |
| RP11-358M14.2 | downregulated lncRNA | 2.001665859 | 8.94E-27 |
| LINC00473 | downregulated lncRNA | 3.578163657 | 1.59E-26 |
| RP11-35J10.6 | downregulated lncRNA | 4.390388669 | 1.86E-26 |
| RP3-368B9.2 | downregulated lncRNA | 3.300173995 | 2.83E-26 |
| CALML3-AS1 | downregulated lncRNA | 2.412375962 | 3.07E-26 |
| FLJ22763 | downregulated lncRNA | 3.357804949 | 3.52E-26 |
| RP11-476H24.1 | downregulated lncRNA | 7.685595973 | 3.91E-26 |
| CTC-391G2.1 | downregulated lncRNA | 3.371066243 | 4.60E-26 |
| RP11-120I21.2 | downregulated lncRNA | 5.061038707 | 4.69E-26 |
| CTC-458G6.2 | downregulated lncRNA | 3.131338825 | 4.91E-26 |
| RP11-641D5.2 | downregulated lncRNA | 2.586149242 | 6.44E-26 |
| AC002401.1 | downregulated lncRNA | 2.642707257 | 7.12E-26 |
| LINC00461 | downregulated lncRNA | 3.377087641 | 7.83E-26 |
| AC099552.4 | downregulated lncRNA | 4.130758239 | 7.96E-26 |
| RP5-881L22.6 | downregulated lncRNA | 3.519110532 | 8.74E-26 |
| C15orf56 | downregulated lncRNA | 3.039820284 | 1.00E-25 |
| AC093802.1 | downregulated lncRNA | 2.880869325 | 1.37E-25 |
| RP11-318G21.4 | downregulated lncRNA | 2.813222591 | 2.71E-25 |
| RP11-536I6.2 | downregulated lncRNA | 3.417443944 | 3.08E-25 |
| RP11-132N15.3 | downregulated lncRNA | 5.003480668 | 3.46E-25 |
| RP11-115H13.1 | downregulated lncRNA | 3.612494117 | 5.02E-25 |
| RP11-95P13.2 | downregulated lncRNA | 3.998857572 | 5.37E-25 |
| PWRN3 | downregulated lncRNA | 3.816902142 | 6.03E-25 |
| AC011239.1 | downregulated lncRNA | 3.159780297 | 6.31E-25 |
| LINC01224 | downregulated lncRNA | 2.551286242 | 1.47E-24 |
| TPTEP1 | downregulated lncRNA | 2.265039948 | 1.76E-24 |
| RP11-754N21.1 | downregulated lncRNA | 3.409246945 | 1.99E-24 |
| MESTIT1 | downregulated lncRNA | 2.416828187 | 2.22E-24 |
| RP11-445F12.1 | downregulated lncRNA | 4.212802665 | 2.62E-24 |
| RP11-129I19.2 | downregulated lncRNA | 4.117981277 | 3.20E-24 |
| RP1-81D8.3 | downregulated lncRNA | 4.15745003 | 3.68E-24 |
| LY86-AS1 | downregulated lncRNA | 2.285258835 | 4.39E-24 |
| RP11-843A23.1 | downregulated lncRNA | 2.082235251 | 4.48E-24 |
| LINC00410 | downregulated lncRNA | 3.62029395 | 8.62E-24 |
| RP11-659E9.2 | downregulated lncRNA | 2.796146814 | 2.07E-23 |
| RP1-167O22.1 | downregulated lncRNA | 2.229579013 | 2.38E-23 |
| RP11-685F15.1 | downregulated lncRNA | 3.567209823 | 3.05E-23 |
| AC005281.2 | downregulated lncRNA | 2.705261282 | 3.78E-23 |
| RP1-170O19.24 | downregulated lncRNA | 2.047291535 | 4.00E-23 |
| LINC00955 | downregulated lncRNA | 3.164820467 | 4.20E-23 |
| RP11-509A17.3 | downregulated lncRNA | 2.55414653 | 7.77E-23 |
| MCF2L-AS1 | downregulated lncRNA | 2.228578051 | 1.55E-22 |
| RP11-554A11.5 | downregulated lncRNA | 2.721859934 | 1.96E-22 |
| RP11-78C3.1 | downregulated lncRNA | 2.798307026 | 2.04E-22 |
| RP11-379F12.4 | downregulated lncRNA | 4.943060788 | 2.07E-22 |
| RP1-67A8.3 | downregulated lncRNA | 2.436985784 | 2.98E-22 |
| RP11-132E11.2 | downregulated lncRNA | 4.92317797 | 3.05E-22 |
| CTC-273B12.10 | downregulated lncRNA | 2.473479506 | 4.30E-22 |
| RP11-587P21.2 | downregulated lncRNA | 3.402798872 | 4.98E-22 |
| ARHGEF26-AS1 | downregulated lncRNA | 2.177335591 | 5.42E-22 |
| LINC01606 | downregulated lncRNA | 4.553873128 | 6.97E-22 |
| RP11-3G21.1 | downregulated lncRNA | 5.795745314 | 7.55E-22 |
| RP11-117L5.4 | downregulated lncRNA | 3.174102674 | 1.63E-21 |
| RP11-1030E3.1 | downregulated lncRNA | 3.424510641 | 3.68E-21 |
| CTD-2626G11.2 | downregulated lncRNA | 3.970900481 | 4.37E-21 |
| RP11-92A5.2 | downregulated lncRNA | 4.198868291 | 4.67E-21 |
| RP11-18H21.2 | downregulated lncRNA | 2.964494908 | 7.19E-21 |
| MYCNUT | downregulated lncRNA | 3.541932682 | 9.18E-21 |
| RP1-154K9.2 | downregulated lncRNA | 3.061172767 | 9.49E-21 |
| LINC01621 | downregulated lncRNA | 2.376395515 | 1.08E-20 |
| LINC01314 | downregulated lncRNA | 2.222059575 | 1.10E-20 |
| WT1-AS | downregulated lncRNA | 2.713473998 | 1.24E-20 |
| TCL6 | downregulated lncRNA | 2.718039795 | 1.42E-20 |
| RP11-354P11.2 | downregulated lncRNA | 3.04591284 | 1.64E-20 |
| AP000344.3 | downregulated lncRNA | 2.25552862 | 1.73E-20 |
| LINC01510 | downregulated lncRNA | 2.178313859 | 2.14E-20 |
| RP11-379F12.3 | downregulated lncRNA | 4.176988335 | 2.39E-20 |
| LINC01561 | downregulated lncRNA | 2.965779422 | 3.56E-20 |
| RP11-128P17.2 | downregulated lncRNA | 3.862277439 | 4.07E-20 |
| RP5-1024C24.1 | downregulated lncRNA | 3.409987878 | 5.24E-20 |
| CTD-2008P7.9 | downregulated lncRNA | 4.976092982 | 6.06E-20 |
| RP11-675F6.3 | downregulated lncRNA | 2.698597402 | 6.74E-20 |
| RP11-480D4.1 | downregulated lncRNA | 2.657022648 | 6.80E-20 |
| RP4-684O24.5 | downregulated lncRNA | 2.262195755 | 7.30E-20 |
| CTD-2251F13.1 | downregulated lncRNA | 3.480460895 | 8.03E-20 |
| RP5-1065P14.2 | downregulated lncRNA | 2.653352573 | 1.11E-19 |
| APCDD1L-AS1 | downregulated lncRNA | 2.344253002 | 1.36E-19 |
| RP1-58B11.1 | downregulated lncRNA | 4.325273241 | 1.47E-19 |
| RP5-875O13.1 | downregulated lncRNA | 2.218840995 | 1.62E-19 |
| RP11-46C24.3 | downregulated lncRNA | 4.028256552 | 1.71E-19 |
| RP11-141O11.1 | downregulated lncRNA | 2.724242089 | 1.79E-19 |
| AC099684.1 | downregulated lncRNA | 2.973427317 | 2.10E-19 |
| RP11-120K24.3 | downregulated lncRNA | 2.505429525 | 2.38E-19 |
| RP5-1056H1.2 | downregulated lncRNA | 2.098879214 | 2.57E-19 |
| CTD-2231H16.1 | downregulated lncRNA | 2.929525661 | 2.91E-19 |
| PCAT14 | downregulated lncRNA | 3.186639508 | 3.39E-19 |
| RP11-473L15.3 | downregulated lncRNA | 2.900634692 | 3.82E-19 |
| LINC01517 | downregulated lncRNA | 2.517681338 | 5.15E-19 |
| RP1-163G9.2 | downregulated lncRNA | 3.329645164 | 5.56E-19 |
| RP11-128P17.1 | downregulated lncRNA | 3.738448715 | 5.94E-19 |
| ADAMTS19-AS1 | downregulated lncRNA | 4.421610934 | 6.13E-19 |
| AC068535.3 | downregulated lncRNA | 4.860728375 | 6.67E-19 |
| RP11-373E16.3 | downregulated lncRNA | 2.659481164 | 6.83E-19 |
| RP4-541C22.5 | downregulated lncRNA | 2.303539462 | 6.92E-19 |
| AP001059.5 | downregulated lncRNA | 2.427414083 | 9.14E-19 |
| AC004901.1 | downregulated lncRNA | 3.041738979 | 9.98E-19 |
| LINC00343 | downregulated lncRNA | 2.582807882 | 1.56E-18 |
| RP11-66N11.7 | downregulated lncRNA | 3.056390387 | 1.70E-18 |
| NAV2-AS3 | downregulated lncRNA | 2.976965412 | 1.75E-18 |
| RP11-706C16.7 | downregulated lncRNA | 3.060819548 | 2.15E-18 |
| CTB-107G13.1 | downregulated lncRNA | 2.165103655 | 3.88E-18 |
| TRPC7-AS2 | downregulated lncRNA | 2.785834196 | 6.70E-18 |
| PWRN1 | downregulated lncRNA | 2.631503813 | 8.57E-18 |
| RP11-663N22.1 | downregulated lncRNA | 2.908415388 | 8.79E-18 |
| CTD-2545H1.2 | downregulated lncRNA | 2.240580202 | 9.65E-18 |
| LINC00602 | downregulated lncRNA | 3.077705035 | 1.06E-17 |
| RP11-552E20.1 | downregulated lncRNA | 2.558982913 | 1.55E-17 |
| RP3-333A15.2 | downregulated lncRNA | 2.005500462 | 1.55E-17 |
| RP11-21A7A.4 | downregulated lncRNA | 3.378848514 | 1.78E-17 |
| LINC01018 | downregulated lncRNA | 2.380985129 | 2.03E-17 |
| RP11-404P21.3 | downregulated lncRNA | 2.60097257 | 2.11E-17 |
| RP11-44F14.2 | downregulated lncRNA | 2.902918783 | 2.53E-17 |
| SLC14A2-AS1 | downregulated lncRNA | 2.031583929 | 3.08E-17 |
| LINC01616 | downregulated lncRNA | 2.851538666 | 3.13E-17 |
| AC123023.1 | downregulated lncRNA | 2.9611769 | 3.28E-17 |
| LINC00051 | downregulated lncRNA | 3.014259105 | 3.43E-17 |
| LINC00284 | downregulated lncRNA | 2.667785422 | 3.53E-17 |
| AC103563.9 | downregulated lncRNA | 2.476500623 | 3.79E-17 |
| TRPC7-AS1 | downregulated lncRNA | 2.021368735 | 4.95E-17 |
| RP11-16D22.2 | downregulated lncRNA | 4.468016519 | 5.60E-17 |
| RP1-7G5.6 | downregulated lncRNA | 2.251543662 | 7.09E-17 |
| TMEM246-AS1 | downregulated lncRNA | 2.059623896 | 7.10E-17 |
| RP5-1100I6.1 | downregulated lncRNA | 5.332377596 | 7.24E-17 |
| RP4-543J13.1 | downregulated lncRNA | 2.191261563 | 8.44E-17 |
| LINC01447 | downregulated lncRNA | 2.107872775 | 8.74E-17 |
| RP11-432J24.2 | downregulated lncRNA | 2.091413858 | 8.96E-17 |
| RP11-451G4.2 | downregulated lncRNA | 2.440667974 | 9.34E-17 |
| RP11-44F14.8 | downregulated lncRNA | 2.576741383 | 1.20E-16 |
| CTB-70G10.1 | downregulated lncRNA | 2.986850976 | 1.40E-16 |
| RP11-55L3.1 | downregulated lncRNA | 4.687430217 | 1.43E-16 |
| RP11-51G5.1 | downregulated lncRNA | 3.469681958 | 1.73E-16 |
| RP3-523K23.2 | downregulated lncRNA | 3.171692332 | 1.82E-16 |
| RP5-881L22.5 | downregulated lncRNA | 3.116907642 | 2.18E-16 |
| RP11-700H6.2 | downregulated lncRNA | 2.167841964 | 2.55E-16 |
| LINC01207 | downregulated lncRNA | 3.51370622 | 3.93E-16 |
| CTC-537E7.2 | downregulated lncRNA | 2.422157835 | 3.93E-16 |
| LINC01485 | downregulated lncRNA | 2.364203651 | 3.93E-16 |
| KIRREL3-AS1 | downregulated lncRNA | 2.792142282 | 3.94E-16 |
| RP6-24A23.3 | downregulated lncRNA | 2.676027503 | 5.06E-16 |
| RP1-170O19.23 | downregulated lncRNA | 2.260340444 | 5.52E-16 |
| CTD-2297D10.2 | downregulated lncRNA | 2.210652587 | 6.59E-16 |
| RP13-30A9.1 | downregulated lncRNA | 4.257980577 | 8.11E-16 |
| RP11-436F23.1 | downregulated lncRNA | 3.647213153 | 8.53E-16 |
| RP3-417L20.4 | downregulated lncRNA | 4.41075957 | 8.86E-16 |
| RP11-89K21.1 | downregulated lncRNA | 2.685254334 | 9.63E-16 |
| RP11-478J18.2 | downregulated lncRNA | 3.370041401 | 1.08E-15 |
| LINC00871 | downregulated lncRNA | 3.839715524 | 1.59E-15 |
| CTD-2130F23.2 | downregulated lncRNA | 3.552526297 | 2.73E-15 |
| RP4-568C11.4 | downregulated lncRNA | 3.112814884 | 4.19E-15 |
| AC034110.1 | downregulated lncRNA | 2.395614614 | 5.00E-15 |
| CCDC39-AS1 | downregulated lncRNA | 2.054436064 | 5.44E-15 |
| RP11-44M6.1 | downregulated lncRNA | 2.197572576 | 8.17E-15 |
| RP13-895J2.3 | downregulated lncRNA | 2.071995694 | 1.15E-14 |
| ANKRD62P1-PARP4P3 | downregulated lncRNA | 2.567951563 | 1.16E-14 |
| RP11-716O23.1 | downregulated lncRNA | 2.675313107 | 1.62E-14 |
| RP13-30A9.2 | downregulated lncRNA | 4.092498869 | 1.73E-14 |
| CTA-398F10.2 | downregulated lncRNA | 2.776520317 | 1.91E-14 |
| AC004066.3 | downregulated lncRNA | 2.209563587 | 2.04E-14 |
| RP6-24A23.7 | downregulated lncRNA | 2.565156829 | 2.22E-14 |
| RP11-13N12.1 | downregulated lncRNA | 2.014880651 | 2.46E-14 |
| LA16c-329F2.1 | downregulated lncRNA | 2.267886448 | 3.02E-14 |
| AC097499.1 | downregulated lncRNA | 3.714420628 | 4.15E-14 |
| RP11-103H7.3 | downregulated lncRNA | 2.147521771 | 5.68E-14 |
| RP5-952N6.1 | downregulated lncRNA | 2.071815888 | 6.95E-14 |
| CTD-2315E11.1 | downregulated lncRNA | 2.909147427 | 7.13E-14 |
| AC098828.2 | downregulated lncRNA | 2.154096056 | 8.25E-14 |
| NPSR1-AS1 | downregulated lncRNA | 3.101298632 | 1.03E-13 |
| AC024592.9 | downregulated lncRNA | 2.325407704 | 1.07E-13 |
| RP5-1173A5.1 | downregulated lncRNA | 2.802579055 | 1.24E-13 |
| LINC00111 | downregulated lncRNA | 2.43929497 | 1.77E-13 |
| AC090505.6 | downregulated lncRNA | 2.137863703 | 1.87E-13 |
| AC142119.1 | downregulated lncRNA | 2.224406757 | 2.27E-13 |
| AF067845.1 | downregulated lncRNA | 2.253348098 | 2.33E-13 |
| RP11-713P17.5 | downregulated lncRNA | 2.604081948 | 2.40E-13 |
| CTD-2008P7.8 | downregulated lncRNA | 3.483063933 | 2.66E-13 |
| LINC01351 | downregulated lncRNA | 3.771047285 | 3.38E-13 |
| RP4-594I10.3 | downregulated lncRNA | 2.057855792 | 3.51E-13 |
| LINC00237 | downregulated lncRNA | 2.186072874 | 3.69E-13 |
| AC011625.1 | downregulated lncRNA | 3.309522016 | 4.56E-13 |
| RP11-498B4.5 | downregulated lncRNA | 2.827246423 | 6.21E-13 |
| RP11-531H8.1 | downregulated lncRNA | 2.440302216 | 6.46E-13 |
| LINC01544 | downregulated lncRNA | 2.936149681 | 7.02E-13 |
| RP11-390E23.3 | downregulated lncRNA | 2.508794604 | 8.11E-13 |
| GPC5-IT1 | downregulated lncRNA | 3.014239591 | 1.01E-12 |
| RP4-813D12.3 | downregulated lncRNA | 2.714566196 | 1.72E-12 |
| RP11-469H8.6 | downregulated lncRNA | 3.97669176 | 1.94E-12 |
| RP11-339D23.1 | downregulated lncRNA | 3.431660619 | 2.34E-12 |
| RP11-351M16.3 | downregulated lncRNA | 2.031743963 | 2.53E-12 |
| RP3-399L15.1 | downregulated lncRNA | 3.439522625 | 2.65E-12 |
| RP11-396O20.1 | downregulated lncRNA | 2.338740058 | 4.43E-12 |
| CHL1-AS2 | downregulated lncRNA | 3.332153438 | 6.64E-12 |
| RP5-1028L10.2 | downregulated lncRNA | 2.295734508 | 7.05E-12 |
| RP11-168L22.2 | downregulated lncRNA | 3.076171271 | 7.62E-12 |
| RP11-320H14.1 | downregulated lncRNA | 4.271558927 | 8.25E-12 |
| AC093326.1 | downregulated lncRNA | 2.8592189 | 8.48E-12 |
| RP11-5P4.3 | downregulated lncRNA | 4.890368815 | 1.01E-11 |
| RP11-438B23.2 | downregulated lncRNA | 2.184772802 | 1.20E-11 |
| RP5-1031J8.1 | downregulated lncRNA | 2.021264116 | 1.40E-11 |
| RP11-538D16.3 | downregulated lncRNA | 2.286782138 | 1.53E-11 |
| RP11-1260E13.1 | downregulated lncRNA | 2.143494492 | 2.17E-11 |
| RP11-107I14.2 | downregulated lncRNA | 2.222651003 | 2.29E-11 |
| RP13-895J2.6 | downregulated lncRNA | 2.648668559 | 3.73E-11 |
| RP11-266E6.3 | downregulated lncRNA | 3.282026011 | 3.86E-11 |
| RP11-675F6.4 | downregulated lncRNA | 2.101992968 | 5.68E-11 |
| RP11-626P14.1 | downregulated lncRNA | 2.232249192 | 6.10E-11 |
| LINC00885 | downregulated lncRNA | 2.351124426 | 7.32E-11 |
| AC079612.1 | downregulated lncRNA | 2.531244346 | 8.84E-11 |
| LA16c-444G7.1 | downregulated lncRNA | 2.10621128 | 1.14E-10 |
| RP3-462D8.2 | downregulated lncRNA | 2.860541727 | 1.21E-10 |
| LINC01255 | downregulated lncRNA | 2.497247191 | 1.50E-10 |
| RP11-26E5.1 | downregulated lncRNA | 2.926948013 | 1.86E-10 |
| XXbac-BPG254F23.7 | downregulated lncRNA | 4.090845877 | 2.12E-10 |
| AC025811.3 | downregulated lncRNA | 2.297449956 | 2.19E-10 |
| LINC00603 | downregulated lncRNA | 2.096679575 | 2.24E-10 |
| LINC00454 | downregulated lncRNA | 2.186287508 | 2.52E-10 |
| LINC00437 | downregulated lncRNA | 2.339702542 | 3.61E-10 |
| AC083867.4 | downregulated lncRNA | 2.017982831 | 3.66E-10 |
| RP11-734K21.5 | downregulated lncRNA | 3.076452489 | 5.18E-10 |
| RP11-560I19.1 | downregulated lncRNA | 2.081989391 | 6.87E-10 |
| RP11-4O3.1 | downregulated lncRNA | 3.010770139 | 8.26E-10 |
| AC093627.11 | downregulated lncRNA | 2.67072866 | 1.34E-09 |
| RP11-531A24.3 | downregulated lncRNA | 2.41785984 | 1.48E-09 |
| RP11-128P17.4 | downregulated lncRNA | 3.736789472 | 1.94E-09 |
| RP11-766F14.1 | downregulated lncRNA | 2.151337857 | 2.19E-09 |
| FAM230C | downregulated lncRNA | 3.25595329 | 2.43E-09 |
| RP11-506E9.3 | downregulated lncRNA | 2.133373856 | 2.83E-09 |
| RP11-1070N10.6 | downregulated lncRNA | 2.214918118 | 3.23E-09 |
| RP1-251I12.1 | downregulated lncRNA | 3.051760061 | 3.46E-09 |
| RP3-470L22.1 | downregulated lncRNA | 2.465810002 | 3.78E-09 |
| CTB-92J24.3 | downregulated lncRNA | 2.180501819 | 4.06E-09 |
| LA16c-325D7.1 | downregulated lncRNA | 2.265565904 | 4.54E-09 |
| RP11-758N13.1 | downregulated lncRNA | 2.406003211 | 4.61E-09 |
| RP11-662M24.2 | downregulated lncRNA | 3.164603731 | 4.98E-09 |
| RP1-283K11.2 | downregulated lncRNA | 2.226748303 | 5.04E-09 |
| RP11-132N15.1 | downregulated lncRNA | 2.889249824 | 5.05E-09 |
| U47924.27 | downregulated lncRNA | 2.919875246 | 5.28E-09 |
| RP11-445K13.2 | downregulated lncRNA | 2.122520929 | 7.10E-09 |
| AC026167.1 | downregulated lncRNA | 2.789617331 | 7.52E-09 |
| LINC01226 | downregulated lncRNA | 2.040515596 | 1.48E-08 |
| RP11-357D18.1 | downregulated lncRNA | 2.397897218 | 1.87E-08 |
| LINC01312 | downregulated lncRNA | 2.410906357 | 2.49E-08 |
| RP11-53B5.1 | downregulated lncRNA | 3.785410901 | 2.71E-08 |
| RP11-941H19.3 | downregulated lncRNA | 2.497775138 | 3.25E-08 |
| AC099552.3 | downregulated lncRNA | 4.087117623 | 3.26E-08 |
| RP11-396O20.2 | downregulated lncRNA | 2.185655302 | 3.26E-08 |
| RP4-555D20.4 | downregulated lncRNA | 2.424310458 | 3.44E-08 |
| AC012307.2 | downregulated lncRNA | 2.165077651 | 3.57E-08 |
| CTB-43E15.1 | downregulated lncRNA | 2.273567037 | 7.24E-08 |
| LINC01612 | downregulated lncRNA | 3.140443031 | 7.36E-08 |
| UCA1 | downregulated lncRNA | 2.094635955 | 9.90E-08 |
| RP11-361I14.2 | downregulated lncRNA | 2.337463939 | 1.16E-07 |
| UG0898H09 | downregulated lncRNA | 2.150085462 | 1.88E-07 |
| CTD-2016O11.1 | downregulated lncRNA | 2.185544671 | 1.93E-07 |
| RP11-76C10.2 | downregulated lncRNA | 2.346881299 | 1.98E-07 |
| RP11-62F24.1 | downregulated lncRNA | 2.083643514 | 2.56E-07 |
| AC004862.6 | downregulated lncRNA | 2.41579838 | 2.94E-07 |
| CTC-535M15.2 | downregulated lncRNA | 2.323194665 | 3.14E-07 |
| XXbac-B33L19.12 | downregulated lncRNA | 2.004670006 | 3.19E-07 |
| RP11-264A11.1 | downregulated lncRNA | 3.758294187 | 3.24E-07 |
| RP11-445F12.2 | downregulated lncRNA | 2.670305972 | 3.25E-07 |
| RP11-654G14.1 | downregulated lncRNA | 2.72450287 | 4.22E-07 |
| RP11-351A20.1 | downregulated lncRNA | 2.259848496 | 4.36E-07 |
| AC011516.2 | downregulated lncRNA | 3.039612835 | 5.05E-07 |
| RP11-482E14.2 | downregulated lncRNA | 2.475466971 | 5.78E-07 |
| LINC00314 | downregulated lncRNA | 2.77024147 | 5.83E-07 |
| RP11-565P22.2 | downregulated lncRNA | 2.238101186 | 6.20E-07 |
| ENOX1-AS2 | downregulated lncRNA | 2.922766016 | 9.53E-07 |
| RP11-132N15.2 | downregulated lncRNA | 2.271920381 | 1.29E-06 |
| AC027119.1 | downregulated lncRNA | 2.410118568 | 2.25E-06 |
| LINC00676 | downregulated lncRNA | 2.226414111 | 2.50E-06 |
| AC090505.1 | downregulated lncRNA | 2.229672864 | 2.93E-06 |
| RP11-554L12.1 | downregulated lncRNA | 2.147608992 | 3.01E-06 |
| RP11-354K4.2 | downregulated lncRNA | 2.730712047 | 3.43E-06 |
| RP5-1121A15.3 | downregulated lncRNA | 2.567119711 | 3.63E-06 |
| FGF10-AS1 | downregulated lncRNA | 2.106281791 | 3.85E-06 |
| LINC01541 | downregulated lncRNA | 2.276904607 | 4.90E-06 |
| LINC00200 | downregulated lncRNA | 2.17971875 | 5.44E-06 |
| RP11-305P14.1 | downregulated lncRNA | 2.057216269 | 5.50E-06 |
| KCNH1-IT1 | downregulated lncRNA | 2.019445228 | 6.28E-06 |
| RP11-167N24.6 | downregulated lncRNA | 2.337509623 | 6.42E-06 |
| CTB-49A3.4 | downregulated lncRNA | 2.570151686 | 6.48E-06 |
| RORB-AS1 | downregulated lncRNA | 2.091270861 | 6.83E-06 |
| RP11-25O3.1 | downregulated lncRNA | 2.038353849 | 6.87E-06 |
| RP11-123K19.1 | downregulated lncRNA | 2.06426353 | 1.25E-05 |
| RP11-146N18.1 | downregulated lncRNA | 2.764330515 | 1.44E-05 |
| RP11-385G16.1 | downregulated lncRNA | 2.281613522 | 1.75E-05 |
| DLGAP2-AS1 | downregulated lncRNA | 2.27023797 | 2.35E-05 |
| RP11-168O22.1 | downregulated lncRNA | 2.864315753 | 2.39E-05 |
| RP11-152O14.1 | downregulated lncRNA | 2.001828941 | 2.51E-05 |
| CTD-2377O17.1 | downregulated lncRNA | 2.148319221 | 2.85E-05 |
| RP13-895J2.7 | downregulated lncRNA | 2.289048171 | 3.32E-05 |
| RP11-687D19.1 | downregulated lncRNA | 2.516431142 | 3.34E-05 |
| RP11-734K21.2 | downregulated lncRNA | 2.483192749 | 4.30E-05 |
| AP000472.3 | downregulated lncRNA | 2.513737917 | 4.77E-05 |
| LINC00507 | downregulated lncRNA | 2.393072362 | 5.24E-05 |
| RP11-670N15.1 | downregulated lncRNA | 2.286014443 | 5.92E-05 |
| RP11-329N22.1 | downregulated lncRNA | 2.592369609 | 6.99E-05 |
| RP11-293M10.1 | downregulated lncRNA | 2.038842777 | 7.67E-05 |
| LINC01538 | downregulated lncRNA | 2.071091607 | 7.89E-05 |
| RP11-278H7.3 | downregulated lncRNA | 2.104727325 | 8.01E-05 |
| AC019055.1 | downregulated lncRNA | 2.853403663 | 8.23E-05 |
| RP11-701I24.3 | downregulated lncRNA | 2.643579766 | 8.28E-05 |
| RP11-350D17.2 | downregulated lncRNA | 2.079717973 | 9.34E-05 |
| RP11-817J15.3 | downregulated lncRNA | 2.04396262 | 9.68E-05 |
| AC009478.1 | downregulated lncRNA | 2.610044027 | 0.000100189 |
| RP11-524H19.2 | downregulated lncRNA | 2.434421822 | 0.000102228 |
| CTD-3118D11.3 | downregulated lncRNA | 2.350347288 | 0.000119967 |
| RP11-109P6.2 | downregulated lncRNA | 2.329328561 | 0.000221311 |
| RP11-384F7.2 | downregulated lncRNA | 2.424376887 | 0.000223019 |
| CTD-2207A17.1 | downregulated lncRNA | 2.227466591 | 0.000225821 |
| RP11-369C8.1 | downregulated lncRNA | 2.238429489 | 0.000227476 |
| CTB-1I21.1 | downregulated lncRNA | 2.484746317 | 0.000287521 |
| LINC00307 | downregulated lncRNA | 2.584806089 | 0.000447274 |
| RP11-5P4.1 | downregulated lncRNA | 2.691283956 | 0.000449621 |
| RP11-315F22.1 | downregulated lncRNA | 2.732778375 | 0.000571051 |
| CTD-2297D10.1 | downregulated lncRNA | 2.000531463 | 0.0006392 |
| EPHA5-AS1 | downregulated lncRNA | 2.185887443 | 0.000675042 |
| RP11-173L6.1 | downregulated lncRNA | 3.273820405 | 0.000698454 |
| RP11-849I19.1 | downregulated lncRNA | 2.160973258 | 0.000886663 |
| RP11-5P22.3 | downregulated lncRNA | 2.263767413 | 0.001268635 |
| LINC00492 | downregulated lncRNA | 2.513080563 | 0.001683328 |
| RP11-142G1.3 | downregulated lncRNA | 2.079841302 | 0.00371794 |
| RP13-436F16.1 | downregulated lncRNA | 2.124227522 | 0.005196931 |
| LINC00919 | downregulated lncRNA | 2.150683308 | 0.005617182 |
| RP11-469N6.3 | downregulated lncRNA | 2.777379846 | 0.006373869 |
| RP11-566H8.3 | downregulated lncRNA | 2.231478809 | 0.006908058 |
| RP11-333B11.1 | downregulated lncRNA | 2.347817952 | 0.007290329 |
| hsa-mir-210 | upregulated miRNA | -3.052092131 | 7.01E-127 |
| hsa-mir-122 | upregulated miRNA | -6.505457564 | 1.27E-119 |
| hsa-mir-155 | upregulated miRNA | -3.512656869 | 8.78E-110 |
| hsa-mir-21 | upregulated miRNA | -2.16105761 | 1.84E-100 |
| hsa-mir-584 | upregulated miRNA | -2.119033142 | 3.87E-90 |
| hsa-mir-592 | upregulated miRNA | -3.061657866 | 3.59E-58 |
| hsa-mir-885 | upregulated miRNA | -3.693079043 | 1.54E-56 |
| hsa-mir-224 | upregulated miRNA | -2.42813291 | 1.14E-52 |
| hsa-mir-4772 | upregulated miRNA | -2.053849557 | 4.59E-46 |
| hsa-mir-6509 | upregulated miRNA | -2.109460405 | 8.00E-26 |
| hsa-mir-3941 | upregulated miRNA | -2.493014078 | 9.15E-25 |
| hsa-mir-4652 | upregulated miRNA | -3.472174447 | 1.56E-21 |
| hsa-mir-4773-1 | upregulated miRNA | -2.930563284 | 2.25E-21 |
| hsa-mir-4773-2 | upregulated miRNA | -3.000429537 | 2.74E-21 |
| hsa-mir-1293 | upregulated miRNA | -3.576893778 | 2.68E-16 |
| hsa-mir-374c | upregulated miRNA | -2.917653451 | 3.13E-14 |
| hsa-mir-599 | upregulated miRNA | -3.415704423 | 6.01E-14 |
| hsa-mir-875 | upregulated miRNA | -3.859494089 | 2.51E-12 |
| hsa-mir-3591 | upregulated miRNA | -2.200897328 | 1.08E-11 |
| hsa-mir-508 | downregulated miRNA | 4.516614491 | 1.86E-109 |
| hsa-mir-514a-1 | downregulated miRNA | 4.524342556 | 3.64E-91 |
| hsa-mir-514a-3 | downregulated miRNA | 4.547643729 | 9.67E-91 |
| hsa-mir-514a-2 | downregulated miRNA | 4.487538928 | 6.90E-85 |
| hsa-mir-506 | downregulated miRNA | 6.015703194 | 3.45E-82 |
| hsa-mir-509-3 | downregulated miRNA | 3.468366315 | 5.68E-75 |
| hsa-mir-362 | downregulated miRNA | 2.588721811 | 1.13E-73 |
| hsa-mir-509-2 | downregulated miRNA | 3.242278892 | 2.65E-73 |
| hsa-mir-509-1 | downregulated miRNA | 3.220848733 | 1.01E-65 |
| hsa-mir-129-1 | downregulated miRNA | 3.885311714 | 3.81E-44 |
| hsa-mir-206 | downregulated miRNA | 3.912384156 | 6.69E-39 |
| hsa-mir-129-2 | downregulated miRNA | 3.652007938 | 8.50E-39 |
| hsa-mir-934 | downregulated miRNA | 5.773248237 | 6.47E-38 |
| hsa-mir-514b | downregulated miRNA | 5.969703205 | 2.55E-33 |
| hsa-mir-184 | downregulated miRNA | 4.307893472 | 2.32E-22 |
| hsa-mir-200c | downregulated miRNA | 2.877015673 | 2.78E-20 |
| hsa-mir-513c | downregulated miRNA | 3.788436692 | 5.08E-20 |
| hsa-mir-1251 | downregulated miRNA | 2.283565146 | 1.41E-15 |
| hsa-mir-203b | downregulated miRNA | 2.519055088 | 1.55E-15 |
| hsa-mir-138-2 | downregulated miRNA | 2.305823261 | 7.33E-15 |
| hsa-mir-138-1 | downregulated miRNA | 2.548922645 | 1.12E-14 |
| hsa-mir-372 | downregulated miRNA | 2.702475063 | 4.43E-14 |
| hsa-mir-216b | downregulated miRNA | 3.594022064 | 9.21E-13 |
| hsa-mir-507 | downregulated miRNA | 4.249605616 | 1.27E-12 |
| hsa-mir-141 | downregulated miRNA | 2.180686028 | 1.37E-09 |
| hsa-mir-6507 | downregulated miRNA | 2.050637013 | 1.27E-05 |
| hsa-mir-513a-1 | downregulated miRNA | 2.654361308 | 0.000112588 |
| hsa-mir-513b | downregulated miRNA | 2.2771479 | 0.005614827 |
| SPAG4 | upregulated mRNA | -4.115853077 | 4.27E-256 |
| NDUFA4L2 | upregulated mRNA | -5.806304211 | 4.42E-239 |
| GABRD | upregulated mRNA | -5.267840373 | 6.39E-228 |
| EGLN3 | upregulated mRNA | -4.258996419 | 6.39E-228 |
| HILPDA | upregulated mRNA | -4.749744969 | 3.80E-220 |
| NOL3 | upregulated mRNA | -3.435946597 | 1.81E-212 |
| SCARB1 | upregulated mRNA | -4.083552851 | 5.47E-204 |
| COL23A1 | upregulated mRNA | -5.191089808 | 6.44E-200 |
| CA9 | upregulated mRNA | -6.051660566 | 1.03E-196 |
| STC2 | upregulated mRNA | -4.257460575 | 1.37E-196 |
| DDB2 | upregulated mRNA | -2.038013588 | 3.51E-195 |
| HSF4 | upregulated mRNA | -5.732774598 | 4.87E-194 |
| CDCA2 | upregulated mRNA | -4.702918236 | 3.18E-193 |
| CDKN2A | upregulated mRNA | -4.95138799 | 5.88E-190 |
| ANGPTL4 | upregulated mRNA | -4.952498597 | 9.57E-185 |
| FABP6 | upregulated mRNA | -6.439071826 | 1.21E-183 |
| TREM2 | upregulated mRNA | -4.267374688 | 5.79E-182 |
| SIGLEC8 | upregulated mRNA | -5.076860507 | 5.70E-180 |
| SAP30 | upregulated mRNA | -2.488749578 | 7.05E-180 |
| VEGFA | upregulated mRNA | -3.4801378 | 3.66E-177 |
| DOC2A | upregulated mRNA | -6.147507113 | 7.27E-175 |
| NETO2 | upregulated mRNA | -3.310057284 | 8.60E-174 |
| SEMA5B | upregulated mRNA | -3.896564286 | 6.66E-173 |
| ST8SIA4 | upregulated mRNA | -3.565256361 | 6.09E-168 |
| TNFAIP6 | upregulated mRNA | -5.668214046 | 1.04E-167 |
| SLC16A3 | upregulated mRNA | -3.052963182 | 5.41E-164 |
| APOC1 | upregulated mRNA | -5.224194251 | 2.46E-162 |
| VIM | upregulated mRNA | -2.581587231 | 3.30E-162 |
| COL5A3 | upregulated mRNA | -3.556055705 | 3.35E-160 |
| GRIK3 | upregulated mRNA | -5.021810215 | 2.43E-159 |
| ARHGEF39 | upregulated mRNA | -2.736974751 | 4.22E-159 |
| NPTX2 | upregulated mRNA | -6.984716607 | 1.20E-158 |
| LILRB1 | upregulated mRNA | -2.936386361 | 1.76E-155 |
| CD70 | upregulated mRNA | -6.110233671 | 3.61E-147 |
| PNCK | upregulated mRNA | -6.63345033 | 1.56E-146 |
| ENPP3 | upregulated mRNA | -4.718916134 | 1.57E-146 |
| CXCR4 | upregulated mRNA | -2.790639194 | 2.62E-146 |
| HK2 | upregulated mRNA | -3.44506074 | 1.25E-145 |
| ARHGAP22 | upregulated mRNA | -2.774669263 | 9.19E-145 |
| MYEOV | upregulated mRNA | -7.12571502 | 6.85E-144 |
| ZNF395 | upregulated mRNA | -2.637755822 | 1.94E-143 |
| P2RX7 | upregulated mRNA | -2.683299781 | 1.15E-142 |
| AGAP2 | upregulated mRNA | -2.716915869 | 1.30E-142 |
| TMEM74B | upregulated mRNA | -3.749609863 | 2.79E-142 |
| MTCP1 | upregulated mRNA | -3.273380262 | 1.43E-141 |
| ODF3B | upregulated mRNA | -3.563494645 | 9.87E-141 |
| CFAP74 | upregulated mRNA | -4.038762243 | 1.16E-138 |
| ESM1 | upregulated mRNA | -3.58018959 | 6.79E-137 |
| EDA2R | upregulated mRNA | -2.299968102 | 9.10E-137 |
| STAMBPL1 | upregulated mRNA | -2.482223496 | 1.19E-136 |
| SDS | upregulated mRNA | -4.266093129 | 3.80E-134 |
| ENO2 | upregulated mRNA | -3.216154305 | 3.92E-134 |
| SLC6A3 | upregulated mRNA | -6.439405683 | 1.45E-133 |
| SOX11 | upregulated mRNA | -5.412284174 | 5.99E-133 |
| CYP2J2 | upregulated mRNA | -5.133317995 | 2.30E-132 |
| NXPH4 | upregulated mRNA | -4.545144938 | 3.38E-131 |
| AHNAK2 | upregulated mRNA | -4.100542518 | 5.62E-131 |
| INHBB | upregulated mRNA | -3.830991911 | 2.10E-130 |
| TMEM91 | upregulated mRNA | -3.759589848 | 4.80E-130 |
| KRBA1 | upregulated mRNA | -2.806843308 | 1.00E-129 |
| DOK3 | upregulated mRNA | -2.648434432 | 9.12E-129 |
| KISS1R | upregulated mRNA | -6.404477984 | 3.65E-128 |
| IGFBP3 | upregulated mRNA | -3.506035715 | 7.81E-128 |
| PIK3R5 | upregulated mRNA | -2.814207404 | 1.16E-127 |
| TMEM155 | upregulated mRNA | -5.152961006 | 1.41E-127 |
| ITGAX | upregulated mRNA | -3.305202485 | 4.33E-127 |
| RASAL3 | upregulated mRNA | -2.689398446 | 9.35E-127 |
| FAM78A | upregulated mRNA | -2.382836525 | 2.58E-126 |
| PARVG | upregulated mRNA | -2.890895897 | 2.87E-126 |
| RAB42 | upregulated mRNA | -4.880045456 | 3.11E-126 |
| IDO1 | upregulated mRNA | -3.965541648 | 3.49E-125 |
| CD300A | upregulated mRNA | -2.993681619 | 5.62E-125 |
| TNFRSF4 | upregulated mRNA | -3.312290789 | 1.27E-124 |
| LAMA4 | upregulated mRNA | -2.667727479 | 2.77E-124 |
| HLA-F | upregulated mRNA | -2.465081916 | 2.14E-123 |
| PHKA2 | upregulated mRNA | -2.193957978 | 3.32E-123 |
| TYMP | upregulated mRNA | -2.966339308 | 4.13E-123 |
| BHLHE41 | upregulated mRNA | -2.762270576 | 4.72E-123 |
| CD1D | upregulated mRNA | -2.285841888 | 4.23E-122 |
| PRDM1 | upregulated mRNA | -2.283909455 | 4.43E-122 |
| GAL3ST4 | upregulated mRNA | -2.899187835 | 9.21E-122 |
| NNMT | upregulated mRNA | -3.939578144 | 9.98E-122 |
| C5orf46 | upregulated mRNA | -7.711813469 | 1.04E-121 |
| CD300LF | upregulated mRNA | -3.029363228 | 3.83E-121 |
| FCGR3A | upregulated mRNA | -3.344201357 | 7.97E-121 |
| GAS2L3 | upregulated mRNA | -3.115413187 | 5.16E-119 |
| ITGAD | upregulated mRNA | -4.803760851 | 1.75E-118 |
| GJC1 | upregulated mRNA | -2.755221609 | 1.90E-117 |
| PLXDC1 | upregulated mRNA | -2.889655524 | 1.44E-116 |
| BIRC7 | upregulated mRNA | -7.079326028 | 2.39E-116 |
| PTHLH | upregulated mRNA | -6.40242663 | 5.48E-116 |
| DGKD | upregulated mRNA | -2.122582527 | 1.19E-115 |
| FATE1 | upregulated mRNA | -3.543341892 | 4.08E-115 |
| TYMS | upregulated mRNA | -2.064787398 | 1.00E-114 |
| FABP7 | upregulated mRNA | -7.440615538 | 2.19E-114 |
| LCP2 | upregulated mRNA | -2.226897573 | 7.14E-114 |
| CAV2 | upregulated mRNA | -2.011042698 | 4.20E-113 |
| TMIGD3 | upregulated mRNA | -2.930997305 | 7.00E-113 |
| PGF | upregulated mRNA | -4.689366616 | 8.12E-113 |
| IL2RB | upregulated mRNA | -3.017951517 | 1.99E-112 |
| ADM | upregulated mRNA | -2.719963769 | 1.23E-111 |
| TRPM2 | upregulated mRNA | -2.777141135 | 1.20E-110 |
| MYO1F | upregulated mRNA | -2.520955235 | 1.24E-110 |
| PLK2 | upregulated mRNA | -2.231730885 | 1.80E-109 |
| LPCAT1 | upregulated mRNA | -2.557965215 | 2.77E-109 |
| HTRA4 | upregulated mRNA | -4.864874952 | 3.74E-109 |
| VASH1 | upregulated mRNA | -2.182930411 | 6.05E-109 |
| SLFN13 | upregulated mRNA | -2.179524468 | 6.79E-109 |
| IL20RB | upregulated mRNA | -6.599381643 | 7.24E-109 |
| ANGPT2 | upregulated mRNA | -3.12065756 | 7.54E-109 |
| RELT | upregulated mRNA | -2.10102703 | 1.76E-108 |
| IGSF6 | upregulated mRNA | -2.700418839 | 1.04E-107 |
| LAPTM5 | upregulated mRNA | -2.550751545 | 1.64E-107 |
| SCD | upregulated mRNA | -3.015623626 | 1.73E-107 |
| OR2A4 | upregulated mRNA | -6.961122493 | 5.42E-107 |
| DNAH11 | upregulated mRNA | -4.643828709 | 7.19E-107 |
| PIK3R6 | upregulated mRNA | -3.393994073 | 6.08E-106 |
| DLX5 | upregulated mRNA | -3.442171172 | 8.05E-106 |
| PSORS1C1 | upregulated mRNA | -2.985104511 | 2.22E-105 |
| STAC3 | upregulated mRNA | -2.29830226 | 3.96E-105 |
| LAIR1 | upregulated mRNA | -2.655524042 | 1.17E-104 |
| TYROBP | upregulated mRNA | -2.674407386 | 3.43E-104 |
| NLRC5 | upregulated mRNA | -2.264016671 | 6.03E-104 |
| CSPG4 | upregulated mRNA | -2.740383812 | 7.29E-104 |
| PFKFB4 | upregulated mRNA | -2.434056614 | 1.64E-103 |
| SLC37A2 | upregulated mRNA | -2.748053054 | 6.96E-103 |
| POU5F1 | upregulated mRNA | -3.429013143 | 1.01E-102 |
| SIGLEC10 | upregulated mRNA | -2.986589137 | 1.32E-102 |
| DPEP2 | upregulated mRNA | -2.293490369 | 2.48E-102 |
| MYO3A | upregulated mRNA | -3.320426181 | 3.71E-102 |
| CES4A | upregulated mRNA | -4.163934099 | 6.20E-102 |
| NUSAP1 | upregulated mRNA | -2.151479702 | 6.22E-102 |
| PLPPR5 | upregulated mRNA | -6.400784991 | 1.02E-101 |
| LGALS9 | upregulated mRNA | -2.199387636 | 1.25E-101 |
| EHD2 | upregulated mRNA | -2.255717318 | 1.27E-101 |
| VSIG1 | upregulated mRNA | -3.98011219 | 1.60E-101 |
| LILRB4 | upregulated mRNA | -3.132019018 | 2.01E-101 |
| ADAMTS7 | upregulated mRNA | -2.452052836 | 6.30E-101 |
| TNFRSF14 | upregulated mRNA | -2.153183668 | 8.24E-101 |
| PSMB9 | upregulated mRNA | -2.238876382 | 2.09E-100 |
| HS3ST2 | upregulated mRNA | -4.91035833 | 2.65E-100 |
| VWF | upregulated mRNA | -3.015978424 | 2.65E-100 |
| FAM111B | upregulated mRNA | -2.530314973 | 7.76E-100 |
| RNASET2 | upregulated mRNA | -3.373512992 | 1.58E-99 |
| CD68 | upregulated mRNA | -3.187021729 | 2.03E-99 |
| TNFSF9 | upregulated mRNA | -3.488095595 | 5.07E-99 |
| RASD2 | upregulated mRNA | -2.905095917 | 2.50E-98 |
| FCER1G | upregulated mRNA | -2.549809751 | 1.52E-97 |
| NKG7 | upregulated mRNA | -3.506403243 | 6.24E-97 |
| LAT2 | upregulated mRNA | -2.170586047 | 8.07E-97 |
| PLA2G7 | upregulated mRNA | -3.679724848 | 1.30E-96 |
| FCGR1A | upregulated mRNA | -3.112193625 | 1.37E-96 |
| LILRB2 | upregulated mRNA | -2.299565225 | 3.09E-96 |
| C1QB | upregulated mRNA | -2.941106844 | 9.01E-96 |
| CLEC2D | upregulated mRNA | -2.581612645 | 2.01E-95 |
| FAM153C | upregulated mRNA | -5.24398898 | 2.07E-95 |
| GNLY | upregulated mRNA | -3.141905019 | 2.35E-95 |
| PKMYT1 | upregulated mRNA | -2.96164449 | 2.67E-95 |
| CAV1 | upregulated mRNA | -2.241682162 | 3.63E-95 |
| KLHL6 | upregulated mRNA | -2.625173655 | 3.71E-95 |
| OSCAR | upregulated mRNA | -2.596636935 | 6.53E-95 |
| SASH3 | upregulated mRNA | -2.491954906 | 9.66E-95 |
| CCND1 | upregulated mRNA | -2.300644466 | 1.41E-94 |
| OLFML2A | upregulated mRNA | -2.687464586 | 1.44E-94 |
| P2RY1 | upregulated mRNA | -2.439910979 | 1.52E-94 |
| FMNL1 | upregulated mRNA | -2.25557113 | 1.64E-94 |
| MS4A14 | upregulated mRNA | -3.228607759 | 2.22E-94 |
| IL12RB1 | upregulated mRNA | -2.605010901 | 2.67E-94 |
| LTB4R | upregulated mRNA | -2.552448946 | 2.94E-94 |
| ADAM18 | upregulated mRNA | -8.219634963 | 4.12E-94 |
| PTPRN | upregulated mRNA | -5.360019903 | 7.56E-94 |
| PYGL | upregulated mRNA | -2.018776733 | 9.56E-94 |
| CDK18 | upregulated mRNA | -2.371121159 | 1.24E-93 |
| CSF3R | upregulated mRNA | -2.764327023 | 2.09E-93 |
| CDC45 | upregulated mRNA | -2.740372076 | 2.24E-93 |
| SPI1 | upregulated mRNA | -2.372277037 | 2.86E-93 |
| TUBA3D | upregulated mRNA | -6.508118202 | 5.75E-93 |
| KLRD1 | upregulated mRNA | -2.347772629 | 6.98E-93 |
| APOBEC3G | upregulated mRNA | -2.346710204 | 9.56E-93 |
| C1QTNF6 | upregulated mRNA | -2.298675192 | 2.20E-92 |
| PRELID2 | upregulated mRNA | -2.087290094 | 2.78E-92 |
| RASSF2 | upregulated mRNA | -2.20138468 | 3.67E-92 |
| LY86 | upregulated mRNA | -2.495761939 | 5.86E-92 |
| HLX | upregulated mRNA | -2.557573993 | 5.98E-92 |
| RUNX3 | upregulated mRNA | -2.697334795 | 8.44E-92 |
| C3 | upregulated mRNA | -3.752866865 | 8.69E-92 |
| GOLGA7B | upregulated mRNA | -3.879412914 | 1.10E-91 |
| RGS1 | upregulated mRNA | -3.121270769 | 2.60E-91 |
| C1orf162 | upregulated mRNA | -2.359938231 | 8.15E-91 |
| ETV7 | upregulated mRNA | -2.710014896 | 8.55E-91 |
| KCNK9 | upregulated mRNA | -4.569283836 | 9.48E-91 |
| TRIM9 | upregulated mRNA | -2.852435045 | 1.09E-90 |
| ATP8B3 | upregulated mRNA | -4.243422075 | 1.83E-90 |
| TRPA1 | upregulated mRNA | -3.767572576 | 2.17E-90 |
| CCDC88B | upregulated mRNA | -2.604590058 | 2.45E-90 |
| HTR6 | upregulated mRNA | -6.453460637 | 2.87E-90 |
| C1QC | upregulated mRNA | -2.76134221 | 3.18E-90 |
| ISG20 | upregulated mRNA | -2.379275102 | 5.27E-90 |
| FAM163A | upregulated mRNA | -3.231755912 | 7.37E-90 |
| BTN3A2 | upregulated mRNA | -2.004161028 | 7.41E-90 |
| PRF1 | upregulated mRNA | -2.649551111 | 9.96E-90 |
| MS4A7 | upregulated mRNA | -2.434226667 | 1.29E-89 |
| FCGR1B | upregulated mRNA | -2.875240843 | 1.45E-89 |
| APOBEC3H | upregulated mRNA | -2.927833798 | 1.52E-89 |
| C1orf127 | upregulated mRNA | -3.16681063 | 4.43E-89 |
| LZTS1 | upregulated mRNA | -2.398850178 | 5.75E-89 |
| C4orf47 | upregulated mRNA | -2.536677677 | 6.93E-89 |
| NFAM1 | upregulated mRNA | -2.126476712 | 9.82E-89 |
| ITGB2 | upregulated mRNA | -2.400195755 | 1.17E-88 |
| ARHGAP9 | upregulated mRNA | -2.573831779 | 1.28E-88 |
| CD247 | upregulated mRNA | -2.598601725 | 1.46E-88 |
| PADI1 | upregulated mRNA | -5.742803551 | 2.21E-88 |
| LOX | upregulated mRNA | -4.048149033 | 2.66E-88 |
| CCL18 | upregulated mRNA | -5.702145395 | 4.91E-88 |
| SIGLEC9 | upregulated mRNA | -2.321920824 | 7.06E-88 |
| BTBD16 | upregulated mRNA | -3.587346235 | 7.18E-88 |
| MCHR1 | upregulated mRNA | -5.736427172 | 9.60E-88 |
| FGD2 | upregulated mRNA | -2.523364837 | 1.29E-87 |
| UHRF1 | upregulated mRNA | -2.786505215 | 1.87E-87 |
| FASLG | upregulated mRNA | -3.507511248 | 2.17E-87 |
| BIN2 | upregulated mRNA | -2.025245543 | 2.38E-87 |
| YPEL4 | upregulated mRNA | -2.677357947 | 3.21E-87 |
| DNAJC5B | upregulated mRNA | -3.766719146 | 5.23E-87 |
| TMC8 | upregulated mRNA | -2.540387951 | 1.05E-86 |
| GZMH | upregulated mRNA | -3.004071574 | 1.24E-86 |
| SLC16A1 | upregulated mRNA | -2.047536537 | 1.34E-86 |
| C6orf223 | upregulated mRNA | -3.509439312 | 2.94E-86 |
| LILRB3 | upregulated mRNA | -2.478488353 | 3.42E-86 |
| OLFML2B | upregulated mRNA | -2.694752548 | 3.91E-86 |
| RUFY4 | upregulated mRNA | -4.990981909 | 9.04E-86 |
| PPP1R3G | upregulated mRNA | -2.144129563 | 1.29E-85 |
| TROAP | upregulated mRNA | -3.272951722 | 1.76E-85 |
| TMSB10 | upregulated mRNA | -2.153865232 | 2.39E-85 |
| TBX21 | upregulated mRNA | -2.48500386 | 6.35E-85 |
| SLA2 | upregulated mRNA | -2.930488507 | 9.17E-85 |
| PRAM1 | upregulated mRNA | -2.477070199 | 1.00E-84 |
| CD72 | upregulated mRNA | -2.734373478 | 1.09E-84 |
| CCL5 | upregulated mRNA | -3.247918804 | 2.19E-84 |
| PLIN2 | upregulated mRNA | -2.918022269 | 2.46E-84 |
| E2F1 | upregulated mRNA | -2.204592099 | 2.56E-84 |
| GZMA | upregulated mRNA | -3.148782221 | 2.62E-84 |
| ALOX15B | upregulated mRNA | -4.303662867 | 2.87E-84 |
| VAV1 | upregulated mRNA | -2.323067436 | 3.80E-84 |
| SCN1B | upregulated mRNA | -2.011880265 | 9.18E-84 |
| GBP5 | upregulated mRNA | -3.524205822 | 1.44E-83 |
| TNFRSF9 | upregulated mRNA | -4.08466658 | 1.47E-83 |
| MYO1G | upregulated mRNA | -2.66024424 | 3.09E-83 |
| LGI4 | upregulated mRNA | -4.59724144 | 3.29E-83 |
| BTK | upregulated mRNA | -2.197161988 | 3.96E-83 |
| RFX8 | upregulated mRNA | -3.451577366 | 5.60E-83 |
| OTOA | upregulated mRNA | -2.944577649 | 6.24E-83 |
| DLL4 | upregulated mRNA | -2.434377828 | 7.41E-83 |
| CP | upregulated mRNA | -4.601166536 | 9.32E-83 |
| LST1 | upregulated mRNA | -2.273859251 | 1.06E-82 |
| PATL2 | upregulated mRNA | -2.866328332 | 1.41E-82 |
| JAKMIP1 | upregulated mRNA | -3.591720344 | 1.91E-82 |
| FERMT3 | upregulated mRNA | -2.119305249 | 2.15E-82 |
| EOMES | upregulated mRNA | -3.5273298 | 2.82E-82 |
| ZAP70 | upregulated mRNA | -2.914765437 | 3.16E-82 |
| HK3 | upregulated mRNA | -2.605613947 | 5.84E-82 |
| KIF18B | upregulated mRNA | -3.25339262 | 8.35E-82 |
| BARX2 | upregulated mRNA | -2.645697732 | 1.02E-81 |
| TNFAIP8L2 | upregulated mRNA | -2.276522969 | 1.58E-81 |
| C10orf99 | upregulated mRNA | -7.264693378 | 1.85E-81 |
| KCNN1 | upregulated mRNA | -4.308011317 | 1.97E-81 |
| CORO1A | upregulated mRNA | -2.445610692 | 2.76E-81 |
| GBP2 | upregulated mRNA | -2.00210718 | 4.12E-81 |
| C10orf10 | upregulated mRNA | -2.268362102 | 4.57E-81 |
| CAPN12 | upregulated mRNA | -3.165227876 | 4.95E-81 |
| PRSS53 | upregulated mRNA | -3.546393092 | 9.04E-81 |
| CTSW | upregulated mRNA | -3.041706216 | 9.18E-81 |
| MCAM | upregulated mRNA | -2.077123634 | 1.24E-80 |
| GAL3ST1 | upregulated mRNA | -2.942914951 | 1.31E-80 |
| FKBP10 | upregulated mRNA | -2.811801956 | 1.85E-80 |
| CST7 | upregulated mRNA | -3.138152819 | 2.73E-80 |
| KCNE3 | upregulated mRNA | -2.133919611 | 3.87E-80 |
| ITGAL | upregulated mRNA | -2.532740616 | 4.09E-80 |
| PNMA2 | upregulated mRNA | -2.835557239 | 4.42E-80 |
| THEMIS2 | upregulated mRNA | -2.076816602 | 6.41E-80 |
| C1QA | upregulated mRNA | -2.628061513 | 1.21E-79 |
| HCK | upregulated mRNA | -2.056017342 | 1.42E-79 |
| MCM10 | upregulated mRNA | -2.534759476 | 1.77E-79 |
| HLA-G | upregulated mRNA | -3.864342223 | 2.52E-79 |
| MUC3A | upregulated mRNA | -3.443584532 | 5.97E-79 |
| EFNA3 | upregulated mRNA | -2.424076207 | 6.91E-79 |
| TRIB3 | upregulated mRNA | -3.14659464 | 7.47E-79 |
| CD8A | upregulated mRNA | -3.60542057 | 1.72E-78 |
| MYBL2 | upregulated mRNA | -3.434404679 | 2.25E-78 |
| AICDA | upregulated mRNA | -4.545716276 | 2.47E-78 |
| FAM26F | upregulated mRNA | -2.982856069 | 4.60E-78 |
| DEF6 | upregulated mRNA | -2.291067043 | 5.55E-78 |
| PSTPIP1 | upregulated mRNA | -2.807004718 | 5.86E-78 |
| SELPLG | upregulated mRNA | -2.063593311 | 6.60E-78 |
| PYHIN1 | upregulated mRNA | -2.988837392 | 9.36E-78 |
| SLC1A3 | upregulated mRNA | -2.737952705 | 9.57E-78 |
| CD84 | upregulated mRNA | -2.795213469 | 1.39E-77 |
| APOBEC3C | upregulated mRNA | -2.11505191 | 1.48E-77 |
| CDH8 | upregulated mRNA | -3.484669473 | 1.57E-77 |
| CD37 | upregulated mRNA | -2.156085455 | 1.60E-77 |
| JAK3 | upregulated mRNA | -2.456760861 | 3.21E-77 |
| CHSY3 | upregulated mRNA | -2.220447841 | 3.73E-77 |
| CRACR2A | upregulated mRNA | -2.177649831 | 5.24E-77 |
| APOBEC3D | upregulated mRNA | -2.03650147 | 1.15E-76 |
| AURKB | upregulated mRNA | -3.058938594 | 1.62E-76 |
| CD2 | upregulated mRNA | -3.033749985 | 2.03E-76 |
| LILRA6 | upregulated mRNA | -2.527777802 | 2.21E-76 |
| MBOAT4 | upregulated mRNA | -2.391823335 | 2.87E-76 |
| BATF | upregulated mRNA | -3.046508715 | 3.21E-76 |
| C8orf22 | upregulated mRNA | -8.035010566 | 3.31E-76 |
| KIF21B | upregulated mRNA | -2.145552264 | 3.69E-76 |
| TNFSF14 | upregulated mRNA | -3.820167296 | 4.74E-76 |
| CXCL10 | upregulated mRNA | -3.40943399 | 4.74E-76 |
| CLEC2B | upregulated mRNA | -2.321282768 | 4.89E-76 |
| DCLK3 | upregulated mRNA | -3.315772007 | 5.02E-76 |
| DIRAS2 | upregulated mRNA | -3.000837556 | 5.67E-76 |
| CCR5 | upregulated mRNA | -2.904637558 | 5.68E-76 |
| MS4A6A | upregulated mRNA | -2.257299933 | 6.40E-76 |
| APLN | upregulated mRNA | -2.466149548 | 7.22E-76 |
| CCNA2 | upregulated mRNA | -2.254304778 | 7.24E-76 |
| NME8 | upregulated mRNA | -2.828578085 | 7.58E-76 |
| CAPN11 | upregulated mRNA | -3.355675141 | 8.27E-76 |
| S1PR5 | upregulated mRNA | -2.415931698 | 1.01E-75 |
| CGREF1 | upregulated mRNA | -3.054562112 | 1.19E-75 |
| CXCL9 | upregulated mRNA | -3.597869512 | 1.25E-75 |
| PRKCDBP | upregulated mRNA | -2.321112074 | 1.46E-75 |
| APBB1IP | upregulated mRNA | -2.434304567 | 1.64E-75 |
| ZNF683 | upregulated mRNA | -3.404352214 | 1.65E-75 |
| TMEM45A | upregulated mRNA | -3.502926638 | 1.67E-75 |
| MXD3 | upregulated mRNA | -2.582146261 | 4.01E-75 |
| MUC12 | upregulated mRNA | -4.367570301 | 4.47E-75 |
| AVPR1B | upregulated mRNA | -3.73109685 | 5.40E-75 |
| WAS | upregulated mRNA | -2.192445328 | 5.43E-75 |
| NKAIN1 | upregulated mRNA | -4.036739699 | 6.87E-75 |
| CLEC7A | upregulated mRNA | -2.36141072 | 8.39E-75 |
| TBC1D10C | upregulated mRNA | -2.560982944 | 1.19E-74 |
| HSPB8 | upregulated mRNA | -2.171558079 | 1.69E-74 |
| NCKAP1L | upregulated mRNA | -2.322375669 | 1.74E-74 |
| TREML1 | upregulated mRNA | -2.969825555 | 2.25E-74 |
| KIF14 | upregulated mRNA | -2.736106798 | 3.26E-74 |
| C19orf67 | upregulated mRNA | -5.173317478 | 5.01E-74 |
| CHRNA1 | upregulated mRNA | -5.341990443 | 6.02E-74 |
| MTCL1 | upregulated mRNA | -2.261348096 | 8.17E-74 |
| STRA8 | upregulated mRNA | -3.354308055 | 8.23E-74 |
| DDIT4 | upregulated mRNA | -2.177800391 | 8.91E-74 |
| ARHGAP30 | upregulated mRNA | -2.036230375 | 1.02E-73 |
| HCG27 | upregulated mRNA | -2.908743865 | 2.18E-73 |
| SLCO1C1 | upregulated mRNA | -2.515634345 | 2.25E-73 |
| SIRPB2 | upregulated mRNA | -2.140920667 | 3.24E-73 |
| CLEC12A | upregulated mRNA | -2.822692794 | 3.58E-73 |
| ACKR3 | upregulated mRNA | -2.293094917 | 3.88E-73 |
| UNC13D | upregulated mRNA | -2.193452697 | 3.93E-73 |
| IL10RA | upregulated mRNA | -2.245082141 | 7.75E-73 |
| CD244 | upregulated mRNA | -2.326944661 | 7.77E-73 |
| E2F2 | upregulated mRNA | -2.374127471 | 8.94E-73 |
| CDT1 | upregulated mRNA | -2.29482321 | 1.11E-72 |
| SLC17A9 | upregulated mRNA | -3.378779073 | 1.30E-72 |
| C11orf21 | upregulated mRNA | -2.661770894 | 1.37E-72 |
| IGLON5 | upregulated mRNA | -6.194548739 | 2.21E-72 |
| RAB7B | upregulated mRNA | -2.072008901 | 2.21E-72 |
| FCRL6 | upregulated mRNA | -2.514923699 | 2.73E-72 |
| DLX1 | upregulated mRNA | -3.208659925 | 3.20E-72 |
| TEX15 | upregulated mRNA | -5.778735118 | 3.24E-72 |
| SAMD3 | upregulated mRNA | -2.350505822 | 3.58E-72 |
| ASF1B | upregulated mRNA | -2.176623082 | 5.15E-72 |
| CRTAM | upregulated mRNA | -3.055877095 | 8.19E-72 |
| HJURP | upregulated mRNA | -2.915717959 | 8.75E-72 |
| DLGAP5 | upregulated mRNA | -2.736310906 | 8.92E-72 |
| LTB4R2 | upregulated mRNA | -2.21162724 | 1.19E-71 |
| ARRDC5 | upregulated mRNA | -3.807528831 | 1.24E-71 |
| CXCL11 | upregulated mRNA | -3.521009764 | 2.00E-71 |
| IL21R | upregulated mRNA | -2.622309106 | 2.11E-71 |
| HLA-DPB1 | upregulated mRNA | -2.078170596 | 2.49E-71 |
| SCGN | upregulated mRNA | -4.678635893 | 2.49E-71 |
| EBF2 | upregulated mRNA | -2.786027412 | 3.27E-71 |
| C9orf172 | upregulated mRNA | -2.093396819 | 3.28E-71 |
| NDC80 | upregulated mRNA | -2.063893447 | 5.26E-71 |
| LSP1 | upregulated mRNA | -2.265940726 | 7.41E-71 |
| NEIL3 | upregulated mRNA | -3.101466654 | 7.42E-71 |
| DTL | upregulated mRNA | -2.150607915 | 8.33E-71 |
| HAPLN3 | upregulated mRNA | -2.40982939 | 8.34E-71 |
| GDF6 | upregulated mRNA | -3.521212704 | 1.10E-70 |
| GNRH1 | upregulated mRNA | -2.843922952 | 1.28E-70 |
| PPFIA4 | upregulated mRNA | -3.175059156 | 1.92E-70 |
| SNX20 | upregulated mRNA | -2.467480242 | 2.23E-70 |
| CCL4 | upregulated mRNA | -2.662204858 | 3.07E-70 |
| ICOS | upregulated mRNA | -3.381177635 | 3.63E-70 |
| ATHL1 | upregulated mRNA | -3.60184196 | 4.23E-70 |
| KIAA0101 | upregulated mRNA | -2.241179532 | 4.32E-70 |
| MMP25 | upregulated mRNA | -2.108491379 | 4.56E-70 |
| HRH2 | upregulated mRNA | -3.441373468 | 4.64E-70 |
| CXCR6 | upregulated mRNA | -2.721328872 | 6.12E-70 |
| CXCR3 | upregulated mRNA | -3.186157034 | 1.23E-69 |
| LPAR5 | upregulated mRNA | -2.170189756 | 1.27E-69 |
| ADAMDEC1 | upregulated mRNA | -4.297671118 | 1.97E-69 |
| ANO4 | upregulated mRNA | -3.752219918 | 2.39E-69 |
| PRR33 | upregulated mRNA | -3.75723186 | 3.04E-69 |
| AMZ1 | upregulated mRNA | -3.807266513 | 3.80E-69 |
| MSC | upregulated mRNA | -3.107749329 | 6.16E-69 |
| CD300C | upregulated mRNA | -2.30042195 | 8.44E-69 |
| ATG16L2 | upregulated mRNA | -2.338172899 | 1.11E-68 |
| FCHO1 | upregulated mRNA | -2.651769145 | 1.29E-68 |
| MATK | upregulated mRNA | -2.272090308 | 1.37E-68 |
| MS4A4E | upregulated mRNA | -3.168061323 | 1.76E-68 |
| LHFPL2 | upregulated mRNA | -2.022801596 | 1.98E-68 |
| LAT | upregulated mRNA | -3.39200061 | 2.43E-68 |
| NCF4 | upregulated mRNA | -2.03354513 | 2.60E-68 |
| EBI3 | upregulated mRNA | -2.317459042 | 3.28E-68 |
| HAPLN1 | upregulated mRNA | -3.557275242 | 4.21E-68 |
| CD6 | upregulated mRNA | -2.381125563 | 4.44E-68 |
| RGS5 | upregulated mRNA | -2.599954251 | 5.19E-68 |
| HMOX1 | upregulated mRNA | -2.335526146 | 5.27E-68 |
| RP11-644F5.10 | upregulated mRNA | -2.034686741 | 6.80E-68 |
| NCAPG | upregulated mRNA | -2.394534582 | 7.61E-68 |
| PLEKHN1 | upregulated mRNA | -2.492053847 | 9.34E-68 |
| PARP15 | upregulated mRNA | -2.698496276 | 1.45E-67 |
| SIRPG | upregulated mRNA | -3.39250477 | 1.82E-67 |
| PLCB2 | upregulated mRNA | -2.143653943 | 1.93E-67 |
| MAP4K1 | upregulated mRNA | -2.41290303 | 2.16E-67 |
| ABCC3 | upregulated mRNA | -2.333018064 | 3.17E-67 |
| GZMB | upregulated mRNA | -2.585232558 | 3.38E-67 |
| IFNG | upregulated mRNA | -4.494591466 | 3.69E-67 |
| LOXL2 | upregulated mRNA | -2.698642376 | 5.02E-67 |
| TNFSF13B | upregulated mRNA | -2.530230896 | 6.31E-67 |
| OR51E1 | upregulated mRNA | -2.504505425 | 8.20E-67 |
| SIGLEC1 | upregulated mRNA | -2.550252083 | 8.29E-67 |
| NOD2 | upregulated mRNA | -2.470523063 | 8.88E-67 |
| TPX2 | upregulated mRNA | -2.519688615 | 1.03E-66 |
| SLAMF8 | upregulated mRNA | -2.71802766 | 1.20E-66 |
| CTAGE9 | upregulated mRNA | -3.975985891 | 1.28E-66 |
| LGALS1 | upregulated mRNA | -2.167509521 | 1.43E-66 |
| HPCA | upregulated mRNA | -2.814326794 | 1.44E-66 |
| UBASH3A | upregulated mRNA | -2.810364945 | 2.05E-66 |
| CD8B | upregulated mRNA | -3.423045066 | 2.20E-66 |
| QRFPR | upregulated mRNA | -2.93605743 | 2.63E-66 |
| CDH4 | upregulated mRNA | -4.084519052 | 2.67E-66 |
| CD86 | upregulated mRNA | -2.151997204 | 3.27E-66 |
| NCF1 | upregulated mRNA | -2.304011608 | 4.06E-66 |
| CSTA | upregulated mRNA | -2.353957146 | 5.03E-66 |
| GSG1L2 | upregulated mRNA | -9.579968277 | 9.30E-66 |
| CDON | upregulated mRNA | -2.244930259 | 9.30E-66 |
| SPINK13 | upregulated mRNA | -4.663353117 | 1.01E-65 |
| TEX11 | upregulated mRNA | -4.237387324 | 1.32E-65 |
| FFAR4 | upregulated mRNA | -2.782537725 | 1.40E-65 |
| TP73 | upregulated mRNA | -3.077167943 | 1.69E-65 |
| GUCA2B | upregulated mRNA | -5.79294882 | 2.85E-65 |
| TNFRSF18 | upregulated mRNA | -2.958397225 | 2.93E-65 |
| MILR1 | upregulated mRNA | -2.000904116 | 3.34E-65 |
| AOAH | upregulated mRNA | -2.189511334 | 3.82E-65 |
| SH2D2A | upregulated mRNA | -2.756032955 | 4.12E-65 |
| CHIT1 | upregulated mRNA | -5.068647178 | 4.12E-65 |
| LAG3 | upregulated mRNA | -3.569655701 | 4.28E-65 |
| RP11-872D17.8 | upregulated mRNA | -3.112818459 | 9.03E-65 |
| PLEK | upregulated mRNA | -2.260527895 | 9.42E-65 |
| RIMKLA | upregulated mRNA | -2.341684175 | 9.54E-65 |
| CD96 | upregulated mRNA | -2.488110728 | 1.36E-64 |
| ADGRE1 | upregulated mRNA | -2.74914987 | 1.42E-64 |
| ZNF469 | upregulated mRNA | -2.07818245 | 1.45E-64 |
| IQGAP3 | upregulated mRNA | -2.503970868 | 1.49E-64 |
| ELOVL2 | upregulated mRNA | -3.230556779 | 1.50E-64 |
| PCSK6 | upregulated mRNA | -2.980737043 | 1.78E-64 |
| CD27 | upregulated mRNA | -3.320975799 | 1.87E-64 |
| ANXA2R | upregulated mRNA | -2.054044088 | 2.15E-64 |
| ASPHD1 | upregulated mRNA | -2.676647771 | 2.73E-64 |
| HLA-DQB1 | upregulated mRNA | -2.307393491 | 3.05E-64 |
| TFR2 | upregulated mRNA | -4.059847721 | 4.18E-64 |
| ATP2B2 | upregulated mRNA | -3.447375152 | 4.63E-64 |
| TGFBI | upregulated mRNA | -4.142275408 | 5.13E-64 |
| CD3D | upregulated mRNA | -2.902400428 | 5.64E-64 |
| ASPM | upregulated mRNA | -2.646114447 | 5.85E-64 |
| CD7 | upregulated mRNA | -2.76366422 | 6.62E-64 |
| PSORS1C2 | upregulated mRNA | -3.767543459 | 1.13E-63 |
| DOCK2 | upregulated mRNA | -2.263189925 | 1.14E-63 |
| GPRIN1 | upregulated mRNA | -2.192056465 | 2.19E-63 |
| SLFN12L | upregulated mRNA | -2.712216982 | 3.13E-63 |
| UBD | upregulated mRNA | -3.184616407 | 4.30E-63 |
| UCN | upregulated mRNA | -2.620633299 | 5.34E-63 |
| GTSE1 | upregulated mRNA | -2.354298918 | 5.43E-63 |
| TNIP3 | upregulated mRNA | -3.773313688 | 6.23E-63 |
| CASP5 | upregulated mRNA | -2.968768576 | 6.85E-63 |
| KCNE4 | upregulated mRNA | -2.056200937 | 8.44E-63 |
| NT5DC3 | upregulated mRNA | -2.280492648 | 1.00E-62 |
| MICALL2 | upregulated mRNA | -2.08032466 | 1.26E-62 |
| SKA3 | upregulated mRNA | -2.104047126 | 1.30E-62 |
| SCGB3A2 | upregulated mRNA | -6.157847348 | 1.46E-62 |
| NCR1 | upregulated mRNA | -2.642768658 | 1.46E-62 |
| ZBP1 | upregulated mRNA | -2.888462982 | 1.55E-62 |
| SLC10A6 | upregulated mRNA | -2.614169741 | 1.97E-62 |
| SLC11A1 | upregulated mRNA | -2.405894064 | 2.69E-62 |
| BATF3 | upregulated mRNA | -2.147315679 | 3.18E-62 |
| CENPM | upregulated mRNA | -2.229159288 | 3.57E-62 |
| CDC25C | upregulated mRNA | -2.747585183 | 5.44E-62 |
| SCG2 | upregulated mRNA | -3.914259614 | 6.17E-62 |
| CYGB | upregulated mRNA | -2.032637661 | 6.89E-62 |
| KIF4A | upregulated mRNA | -2.232938991 | 1.03E-61 |
| KLRK1 | upregulated mRNA | -3.418407175 | 1.07E-61 |
| GZMK | upregulated mRNA | -3.353358022 | 1.86E-61 |
| GFI1 | upregulated mRNA | -2.494757473 | 2.00E-61 |
| BUB1 | upregulated mRNA | -2.42814532 | 2.70E-61 |
| PTGDR | upregulated mRNA | -2.230401622 | 3.25E-61 |
| RTEL1-TNFRSF6B | upregulated mRNA | -2.497574192 | 3.32E-61 |
| ADA | upregulated mRNA | -2.245781911 | 4.33E-61 |
| MARCH4 | upregulated mRNA | -2.788215554 | 4.88E-61 |
| HAMP | upregulated mRNA | -3.800387431 | 1.40E-60 |
| COX4I2 | upregulated mRNA | -2.886653498 | 1.62E-60 |
| KIAA0895L | upregulated mRNA | -2.071577178 | 1.68E-60 |
| MELK | upregulated mRNA | -2.400558081 | 2.24E-60 |
| ADAMTS14 | upregulated mRNA | -3.420609929 | 3.19E-60 |
| DNAJB13 | upregulated mRNA | -3.758653405 | 3.28E-60 |
| PTTG1 | upregulated mRNA | -2.392085184 | 3.28E-60 |
| PLK1 | upregulated mRNA | -2.400059208 | 3.69E-60 |
| SLAMF7 | upregulated mRNA | -2.831956261 | 4.29E-60 |
| PTCRA | upregulated mRNA | -2.957809262 | 5.40E-60 |
| TBX15 | upregulated mRNA | -3.136638081 | 5.61E-60 |
| BIRC3 | upregulated mRNA | -2.336835568 | 8.34E-60 |
| SPN | upregulated mRNA | -2.142323549 | 8.77E-60 |
| TICRR | upregulated mRNA | -2.315928777 | 1.61E-59 |
| SLC12A5 | upregulated mRNA | -2.443700705 | 1.68E-59 |
| IKZF1 | upregulated mRNA | -2.084388905 | 1.74E-59 |
| TIGIT | upregulated mRNA | -2.90474234 | 1.75E-59 |
| SLC2A1 | upregulated mRNA | -2.006335398 | 1.82E-59 |
| GPR19 | upregulated mRNA | -2.233301194 | 3.63E-59 |
| B4GALNT1 | upregulated mRNA | -4.078210955 | 3.74E-59 |
| HES4 | upregulated mRNA | -2.326979968 | 3.77E-59 |
| PIEZO2 | upregulated mRNA | -2.158993913 | 4.50E-59 |
| FOXM1 | upregulated mRNA | -2.445155789 | 5.00E-59 |
| CD3E | upregulated mRNA | -2.663636488 | 5.64E-59 |
| FPR3 | upregulated mRNA | -2.210810867 | 6.52E-59 |
| HLA-DQA1 | upregulated mRNA | -2.214703743 | 7.79E-59 |
| TESPA1 | upregulated mRNA | -2.471520138 | 1.13E-58 |
| MLC1 | upregulated mRNA | -2.216952704 | 1.17E-58 |
| RNF175 | upregulated mRNA | -2.438552116 | 1.66E-58 |
| E2F8 | upregulated mRNA | -2.672718457 | 1.75E-58 |
| GABRE | upregulated mRNA | -2.958401722 | 1.79E-58 |
| SPC24 | upregulated mRNA | -2.342743498 | 1.83E-58 |
| SLAMF6 | upregulated mRNA | -2.538569493 | 2.89E-58 |
| APOL5 | upregulated mRNA | -4.003889101 | 3.54E-58 |
| TOP2A | upregulated mRNA | -2.326379768 | 4.33E-58 |
| HSD3B7 | upregulated mRNA | -2.247472793 | 5.31E-58 |
| CDHR1 | upregulated mRNA | -4.222508131 | 5.83E-58 |
| OPN4 | upregulated mRNA | -5.450083349 | 6.84E-58 |
| TSPAN32 | upregulated mRNA | -2.033719366 | 7.41E-58 |
| RAD54L | upregulated mRNA | -2.521956941 | 9.47E-58 |
| MEFV | upregulated mRNA | -2.269039465 | 1.24E-57 |
| LIMD2 | upregulated mRNA | -2.024171586 | 1.47E-57 |
| TMEM233 | upregulated mRNA | -2.099622664 | 1.74E-57 |
| CDC6 | upregulated mRNA | -2.013261701 | 1.91E-57 |
| DTHD1 | upregulated mRNA | -3.253593443 | 2.30E-57 |
| EME1 | upregulated mRNA | -2.109799602 | 2.58E-57 |
| LRRC25 | upregulated mRNA | -2.310540907 | 3.23E-57 |
| ITGAM | upregulated mRNA | -2.013104563 | 3.66E-57 |
| SIRPB1 | upregulated mRNA | -2.676284479 | 4.47E-57 |
| CTC-479C5.12 | upregulated mRNA | -2.136875498 | 5.47E-57 |
| FAM193B | upregulated mRNA | -2.125761768 | 5.81E-57 |
| EIF4EBP1 | upregulated mRNA | -2.142428486 | 8.65E-57 |
| IKZF3 | upregulated mRNA | -2.585471061 | 1.00E-56 |
| SLC6A1 | upregulated mRNA | -2.301066196 | 1.28E-56 |
| CEP55 | upregulated mRNA | -2.383200555 | 1.79E-56 |
| CD3G | upregulated mRNA | -2.563975568 | 2.29E-56 |
| MMP9 | upregulated mRNA | -3.78392925 | 3.46E-56 |
| PDCD1 | upregulated mRNA | -3.342200049 | 4.51E-56 |
| LGALS12 | upregulated mRNA | -4.168771577 | 7.16E-56 |
| CKAP2L | upregulated mRNA | -2.261993437 | 7.16E-56 |
| TMEM145 | upregulated mRNA | -3.893656522 | 9.42E-56 |
| CD80 | upregulated mRNA | -2.483788177 | 1.24E-55 |
| BIRC5 | upregulated mRNA | -2.499284294 | 1.41E-55 |
| SIT1 | upregulated mRNA | -2.722195406 | 2.06E-55 |
| SLC29A4 | upregulated mRNA | -2.205114197 | 2.37E-55 |
| HLA-DQB2 | upregulated mRNA | -2.821035286 | 3.07E-55 |
| RAC2 | upregulated mRNA | -2.098921554 | 3.62E-55 |
| SH2D5 | upregulated mRNA | -2.492566033 | 3.98E-55 |
| IBSP | upregulated mRNA | -5.29012 | 4.45E-55 |
| CPA6 | upregulated mRNA | -2.53454398 | 5.37E-55 |
| PTPN22 | upregulated mRNA | -2.085909866 | 6.96E-55 |
| TNNI2 | upregulated mRNA | -2.468558752 | 9.71E-55 |
| LBX2 | upregulated mRNA | -2.068532621 | 9.79E-55 |
| PRR7 | upregulated mRNA | -2.406312013 | 1.01E-54 |
| MMP11 | upregulated mRNA | -2.045489568 | 1.25E-54 |
| RTP2 | upregulated mRNA | -5.295029485 | 1.25E-54 |
| UNC5A | upregulated mRNA | -3.445432477 | 1.27E-54 |
| MAPK8IP3 | upregulated mRNA | -2.11485498 | 1.34E-54 |
| RHOH | upregulated mRNA | -2.236837306 | 1.76E-54 |
| BCL2A1 | upregulated mRNA | -2.585672319 | 2.24E-54 |
| UBE2C | upregulated mRNA | -2.969790215 | 2.41E-54 |
| AIM2 | upregulated mRNA | -3.071937006 | 2.51E-54 |
| CTLA4 | upregulated mRNA | -2.976780113 | 3.03E-54 |
| PYCARD | upregulated mRNA | -2.088577084 | 3.24E-54 |
| HES5 | upregulated mRNA | -3.854537627 | 3.92E-54 |
| TENM1 | upregulated mRNA | -2.476773447 | 4.09E-54 |
| ALDOC | upregulated mRNA | -2.295083264 | 4.89E-54 |
| FAM64A | upregulated mRNA | -2.323891979 | 8.61E-54 |
| RIN1 | upregulated mRNA | -2.067984927 | 1.30E-53 |
| S100Z | upregulated mRNA | -2.288323955 | 1.39E-53 |
| LTA | upregulated mRNA | -2.658635087 | 1.48E-53 |
| MAP3K7CL | upregulated mRNA | -2.129139611 | 1.62E-53 |
| MMP16 | upregulated mRNA | -2.740730057 | 1.78E-53 |
| ITPKA | upregulated mRNA | -3.751357054 | 1.88E-53 |
| TUBA3E | upregulated mRNA | -6.246685351 | 3.00E-53 |
| SIGLEC7 | upregulated mRNA | -2.12586961 | 3.59E-53 |
| PCDH17 | upregulated mRNA | -2.004577062 | 4.31E-53 |
| ADAMTS10 | upregulated mRNA | -2.189841092 | 5.47E-53 |
| ADAMTSL4 | upregulated mRNA | -2.1247384 | 7.99E-53 |
| COL5A2 | upregulated mRNA | -2.076801603 | 1.24E-52 |
| DLX6 | upregulated mRNA | -2.606736507 | 1.27E-52 |
| PLD4 | upregulated mRNA | -2.275183814 | 1.47E-52 |
| NMB | upregulated mRNA | -2.591543715 | 1.49E-52 |
| CD52 | upregulated mRNA | -2.122267138 | 1.55E-52 |
| MSR1 | upregulated mRNA | -2.085597754 | 2.11E-52 |
| NPIPB5 | upregulated mRNA | -2.967764843 | 2.79E-52 |
| GZMM | upregulated mRNA | -2.100540582 | 3.34E-52 |
| EIF4A1 | upregulated mRNA | -2.147962176 | 5.13E-52 |
| P4HA3 | upregulated mRNA | -2.859385473 | 5.33E-52 |
| DEPDC1 | upregulated mRNA | -2.393904271 | 6.84E-52 |
| TTC24 | upregulated mRNA | -3.784745877 | 7.75E-52 |
| SEZ6L2 | upregulated mRNA | -2.299845277 | 1.02E-51 |
| PRRT2 | upregulated mRNA | -2.445263758 | 1.07E-51 |
| CXCL5 | upregulated mRNA | -5.012049475 | 1.10E-51 |
| CXorf65 | upregulated mRNA | -3.479379429 | 1.23E-51 |
| GPR141 | upregulated mRNA | -2.3234598 | 1.36E-51 |
| CD180 | upregulated mRNA | -2.140998668 | 1.72E-51 |
| CTHRC1 | upregulated mRNA | -3.169788762 | 2.31E-51 |
| SLC17A4 | upregulated mRNA | -3.614215706 | 4.26E-51 |
| SH2D1A | upregulated mRNA | -2.488354349 | 4.31E-51 |
| HIST1H2AI | upregulated mRNA | -3.949326026 | 5.28E-51 |
| F2 | upregulated mRNA | -5.437202229 | 5.85E-51 |
| XCL2 | upregulated mRNA | -2.588409403 | 6.17E-51 |
| SIX1 | upregulated mRNA | -2.477537172 | 6.33E-51 |
| UBAP1L | upregulated mRNA | -2.195244181 | 7.21E-51 |
| AANAT | upregulated mRNA | -2.653708704 | 7.92E-51 |
| KIF20A | upregulated mRNA | -2.377486075 | 8.42E-51 |
| RBM46 | upregulated mRNA | -3.669117486 | 9.58E-51 |
| CD5L | upregulated mRNA | -4.907768425 | 9.71E-51 |
| POLQ | upregulated mRNA | -2.173684028 | 9.72E-51 |
| AHSA2 | upregulated mRNA | -2.123707031 | 9.73E-51 |
| PLAC8L1 | upregulated mRNA | -2.673299532 | 2.24E-50 |
| RLTPR | upregulated mRNA | -2.532829009 | 2.25E-50 |
| RP11-793H13.10 | upregulated mRNA | -2.339919819 | 3.74E-50 |
| CLEC12B | upregulated mRNA | -3.772551162 | 4.51E-50 |
| CXCL13 | upregulated mRNA | -4.218232584 | 4.51E-50 |
| STMN3 | upregulated mRNA | -2.166845009 | 4.69E-50 |
| DCSTAMP | upregulated mRNA | -3.025097656 | 6.45E-50 |
| MKI67 | upregulated mRNA | -2.236527612 | 8.32E-50 |
| ZP1 | upregulated mRNA | -4.700627236 | 1.24E-49 |
| NLGN1 | upregulated mRNA | -2.031846115 | 1.31E-49 |
| MYH15 | upregulated mRNA | -2.407112114 | 1.32E-49 |
| CLECL1 | upregulated mRNA | -2.323336114 | 2.04E-49 |
| LRRC71 | upregulated mRNA | -3.034171267 | 2.46E-49 |
| NFKBID | upregulated mRNA | -2.195227533 | 2.54E-49 |
| ATG9B | upregulated mRNA | -2.951939852 | 2.83E-49 |
| ADSSL1 | upregulated mRNA | -2.469300359 | 3.04E-49 |
| PKD2L1 | upregulated mRNA | -3.236650961 | 6.07E-49 |
| PAQR6 | upregulated mRNA | -2.625982866 | 6.14E-49 |
| LAIR2 | upregulated mRNA | -3.51281914 | 6.17E-49 |
| LINGO3 | upregulated mRNA | -2.518763944 | 7.76E-49 |
| CD5 | upregulated mRNA | -2.122594672 | 8.32E-49 |
| FIBCD1 | upregulated mRNA | -4.411956402 | 8.69E-49 |
| KIAA0319 | upregulated mRNA | -2.867969262 | 1.06E-48 |
| E2F7 | upregulated mRNA | -2.444155389 | 1.22E-48 |
| HSD17B3 | upregulated mRNA | -3.077792383 | 1.25E-48 |
| AGER | upregulated mRNA | -2.107766419 | 1.35E-48 |
| IFI30 | upregulated mRNA | -2.337488342 | 1.79E-48 |
| IL4I1 | upregulated mRNA | -2.133589414 | 2.61E-48 |
| PLEKHG4 | upregulated mRNA | -2.146009375 | 2.75E-48 |
| CENPA | upregulated mRNA | -2.47575292 | 4.25E-48 |
| FGG | upregulated mRNA | -6.208183371 | 4.29E-48 |
| C16orf74 | upregulated mRNA | -3.098889293 | 4.38E-48 |
| TNNT1 | upregulated mRNA | -4.744712778 | 5.57E-48 |
| HP | upregulated mRNA | -5.884131661 | 6.78E-48 |
| LYZ | upregulated mRNA | -2.45084576 | 8.77E-48 |
| LY6G5B | upregulated mRNA | -2.384480035 | 9.10E-48 |
| PBK | upregulated mRNA | -2.111357487 | 9.59E-48 |
| ITK | upregulated mRNA | -2.116432073 | 1.06E-47 |
| PMCH | upregulated mRNA | -4.889930917 | 1.17E-47 |
| CRYGS | upregulated mRNA | -2.136230727 | 1.19E-47 |
| NGF | upregulated mRNA | -2.141277869 | 1.24E-47 |
| SKA1 | upregulated mRNA | -2.15710332 | 1.27E-47 |
| SLC2A3 | upregulated mRNA | -2.035451746 | 1.36E-47 |
| KIR2DL4 | upregulated mRNA | -2.41689316 | 1.40E-47 |
| DLK2 | upregulated mRNA | -2.358289851 | 1.63E-47 |
| HHLA2 | upregulated mRNA | -3.131328042 | 1.66E-47 |
| TMPRSS9 | upregulated mRNA | -2.753120917 | 2.19E-47 |
| NPIPB4 | upregulated mRNA | -2.012698929 | 3.32E-47 |
| DLX2 | upregulated mRNA | -3.157569632 | 3.44E-47 |
| PAEP | upregulated mRNA | -7.373221025 | 3.62E-47 |
| ARHGAP33 | upregulated mRNA | -2.124596729 | 4.23E-47 |
| UGT1A3 | upregulated mRNA | -4.297596745 | 6.45E-47 |
| ZNF831 | upregulated mRNA | -2.268852466 | 1.01E-46 |
| CACNA2D4 | upregulated mRNA | -2.091681808 | 1.12E-46 |
| TEN1-CDK3 | upregulated mRNA | -2.034292676 | 1.28E-46 |
| MSH4 | upregulated mRNA | -2.969395817 | 1.60E-46 |
| ACAN | upregulated mRNA | -2.621817297 | 1.78E-46 |
| PIF1 | upregulated mRNA | -2.28555196 | 1.90E-46 |
| APOB | upregulated mRNA | -4.199479104 | 2.02E-46 |
| OTOF | upregulated mRNA | -2.621221735 | 3.82E-46 |
| DHRS9 | upregulated mRNA | -2.414530805 | 3.96E-46 |
| TSHR | upregulated mRNA | -2.401117186 | 4.40E-46 |
| RAB33A | upregulated mRNA | -2.16359389 | 4.51E-46 |
| SLFNL1 | upregulated mRNA | -2.230499659 | 4.79E-46 |
| CTC-435M10.3 | upregulated mRNA | -2.792489548 | 4.94E-46 |
| TMEM179 | upregulated mRNA | -4.016565529 | 5.26E-46 |
| FAM57B | upregulated mRNA | -3.709092387 | 8.14E-46 |
| CLEC1B | upregulated mRNA | -2.839467135 | 8.32E-46 |
| KCNMA1 | upregulated mRNA | -2.299076124 | 8.37E-46 |
| MDS2 | upregulated mRNA | -2.223103393 | 8.84E-46 |
| MSH5 | upregulated mRNA | -2.203604311 | 8.99E-46 |
| LY6H | upregulated mRNA | -3.506303848 | 9.64E-46 |
| TERT | upregulated mRNA | -6.041799121 | 1.09E-45 |
| CCL20 | upregulated mRNA | -3.65230062 | 1.10E-45 |
| CATSPER1 | upregulated mRNA | -2.02760477 | 1.11E-45 |
| ADAMTS20 | upregulated mRNA | -5.493433536 | 1.43E-45 |
| SLC17A2 | upregulated mRNA | -3.296483982 | 1.66E-45 |
| FCRL3 | upregulated mRNA | -2.835035961 | 1.84E-45 |
| PERM1 | upregulated mRNA | -3.10451366 | 2.04E-45 |
| RRM2 | upregulated mRNA | -2.181640131 | 2.11E-45 |
| GAPT | upregulated mRNA | -2.103140987 | 2.34E-45 |
| KRT81 | upregulated mRNA | -3.36995659 | 3.93E-45 |
| ADAM8 | upregulated mRNA | -2.151281661 | 4.00E-45 |
| PPEF1 | upregulated mRNA | -2.547122903 | 4.47E-45 |
| NKPD1 | upregulated mRNA | -2.698413037 | 5.24E-45 |
| LY9 | upregulated mRNA | -2.114534072 | 6.52E-45 |
| HCST | upregulated mRNA | -2.037746681 | 6.72E-45 |
| CCDC141 | upregulated mRNA | -2.144949062 | 1.07E-44 |
| HOXD13 | upregulated mRNA | -3.196024577 | 1.98E-44 |
| NPIPB3 | upregulated mRNA | -2.178106143 | 2.00E-44 |
| MUC17 | upregulated mRNA | -6.645508616 | 2.10E-44 |
| SMTNL1 | upregulated mRNA | -2.969347558 | 2.61E-44 |
| SIRPD | upregulated mRNA | -3.162171681 | 3.48E-44 |
| TIMP1 | upregulated mRNA | -2.087822651 | 3.92E-44 |
| CDK3 | upregulated mRNA | -2.431009361 | 4.81E-44 |
| MALL | upregulated mRNA | -2.068349242 | 6.41E-44 |
| CD36 | upregulated mRNA | -2.182542714 | 6.57E-44 |
| KRT36 | upregulated mRNA | -3.748968748 | 7.09E-44 |
| ALOX5 | upregulated mRNA | -2.106280081 | 1.07E-43 |
| LMNTD2 | upregulated mRNA | -2.015802166 | 1.27E-43 |
| RP11-514O12.4 | upregulated mRNA | -2.25994057 | 1.40E-43 |
| SAPCD1 | upregulated mRNA | -3.008397124 | 1.42E-43 |
| PLA2G5 | upregulated mRNA | -2.810144683 | 1.45E-43 |
| GOLGA8A | upregulated mRNA | -2.868265263 | 1.83E-43 |
| SCNN1D | upregulated mRNA | -2.415763878 | 1.93E-43 |
| ATP2A1 | upregulated mRNA | -2.398527511 | 2.44E-43 |
| KIR2DL3 | upregulated mRNA | -2.586573098 | 2.71E-43 |
| RNASE2 | upregulated mRNA | -2.453481139 | 3.23E-43 |
| MEF2B | upregulated mRNA | -2.043841806 | 3.67E-43 |
| DLX4 | upregulated mRNA | -2.691979374 | 4.09E-43 |
| TMIGD2 | upregulated mRNA | -2.202673272 | 5.27E-43 |
| TLR7 | upregulated mRNA | -2.095640254 | 7.84E-43 |
| PAQR9 | upregulated mRNA | -3.667505695 | 7.94E-43 |
| SLITRK5 | upregulated mRNA | -3.420862297 | 8.64E-43 |
| TPRG1 | upregulated mRNA | -2.29018092 | 8.79E-43 |
| EN1 | upregulated mRNA | -3.612420476 | 1.02E-42 |
| ADAM11 | upregulated mRNA | -2.112477237 | 1.09E-42 |
| HPX | upregulated mRNA | -2.451946485 | 1.31E-42 |
| RASL10A | upregulated mRNA | -2.068498675 | 1.32E-42 |
| CLVS2 | upregulated mRNA | -4.100680996 | 1.48E-42 |
| HEATR9 | upregulated mRNA | -3.474824746 | 1.75E-42 |
| C1QL1 | upregulated mRNA | -3.714370096 | 2.40E-42 |
| PLA2G4D | upregulated mRNA | -4.509018285 | 2.77E-42 |
| PMFBP1 | upregulated mRNA | -2.120324368 | 2.85E-42 |
| TBX5 | upregulated mRNA | -6.208415764 | 2.89E-42 |
| VWCE | upregulated mRNA | -2.785730204 | 2.99E-42 |
| CHRNA6 | upregulated mRNA | -3.010724998 | 3.06E-42 |
| TRAT1 | upregulated mRNA | -2.373235465 | 3.29E-42 |
| XCL1 | upregulated mRNA | -2.395178764 | 3.82E-42 |
| DCLK1 | upregulated mRNA | -2.156506094 | 4.81E-42 |
| FBXO39 | upregulated mRNA | -2.328660559 | 8.76E-42 |
| BTNL9 | upregulated mRNA | -2.180465699 | 8.98E-42 |
| COL1A1 | upregulated mRNA | -2.814927708 | 9.99E-42 |
| F2RL3 | upregulated mRNA | -2.352068497 | 1.03E-41 |
| EPO | upregulated mRNA | -4.929335526 | 1.05E-41 |
| CD200R1 | upregulated mRNA | -2.272376318 | 1.28E-41 |
| OR10Q1 | upregulated mRNA | -4.760359906 | 1.71E-41 |
| ITIH1 | upregulated mRNA | -4.670700465 | 2.64E-41 |
| AQP9 | upregulated mRNA | -3.394283495 | 3.95E-41 |
| CCL4L2 | upregulated mRNA | -2.568383292 | 7.31E-41 |
| NEK2 | upregulated mRNA | -2.078759604 | 9.33E-41 |
| INHBE | upregulated mRNA | -3.355587466 | 9.96E-41 |
| CDH23 | upregulated mRNA | -2.371964279 | 1.33E-40 |
| ANKK1 | upregulated mRNA | -2.216729197 | 1.52E-40 |
| TLR8 | upregulated mRNA | -2.136638337 | 1.89E-40 |
| FBLN7 | upregulated mRNA | -2.207268594 | 3.41E-40 |
| SLC18A3 | upregulated mRNA | -7.673257907 | 5.08E-40 |
| ANKLE1 | upregulated mRNA | -2.077668669 | 5.65E-40 |
| GPR18 | upregulated mRNA | -2.206939969 | 6.00E-40 |
| OR51E2 | upregulated mRNA | -2.221024385 | 6.44E-40 |
| CACNG8 | upregulated mRNA | -2.217277237 | 6.72E-40 |
| KIR2DL1 | upregulated mRNA | -2.468388515 | 8.11E-40 |
| SPTA1 | upregulated mRNA | -2.694954303 | 8.41E-40 |
| OR52N4 | upregulated mRNA | -2.561214621 | 9.71E-40 |
| THEMIS | upregulated mRNA | -2.232405503 | 1.53E-39 |
| WDR97 | upregulated mRNA | -2.454000071 | 1.68E-39 |
| BGLAP | upregulated mRNA | -2.335834443 | 1.70E-39 |
| GPR82 | upregulated mRNA | -2.071591491 | 1.75E-39 |
| KCTD19 | upregulated mRNA | -2.474190559 | 2.68E-39 |
| MLIP | upregulated mRNA | -2.167740303 | 3.03E-39 |
| MYB | upregulated mRNA | -2.421649135 | 3.48E-39 |
| PSD2 | upregulated mRNA | -2.436628764 | 3.85E-39 |
| IGFL2 | upregulated mRNA | -4.304254398 | 4.25E-39 |
| NHLH1 | upregulated mRNA | -2.093642496 | 4.30E-39 |
| SLC16A8 | upregulated mRNA | -2.089485472 | 5.04E-39 |
| HIST1H3H | upregulated mRNA | -2.593506706 | 5.75E-39 |
| PCDHGC5 | upregulated mRNA | -2.180634937 | 7.51E-39 |
| CLEC5A | upregulated mRNA | -2.077390534 | 8.40E-39 |
| TAS2R20 | upregulated mRNA | -2.0132354 | 8.73E-39 |
| ABCA12 | upregulated mRNA | -2.464680227 | 9.31E-39 |
| MYO1A | upregulated mRNA | -2.737717408 | 9.44E-39 |
| IL27 | upregulated mRNA | -2.461229461 | 1.02E-38 |
| AL589743.1 | upregulated mRNA | -3.022877241 | 1.39E-38 |
| C3orf36 | upregulated mRNA | -2.114104399 | 1.45E-38 |
| CDCA7 | upregulated mRNA | -2.031870491 | 1.74E-38 |
| ADD2 | upregulated mRNA | -2.73144036 | 1.93E-38 |
| DRD4 | upregulated mRNA | -2.008375438 | 2.09E-38 |
| LILRA4 | upregulated mRNA | -2.538752548 | 3.11E-38 |
| FCGR2B | upregulated mRNA | -2.004637461 | 3.44E-38 |
| DNASE2B | upregulated mRNA | -2.950665099 | 4.53E-38 |
| COL21A1 | upregulated mRNA | -2.227360669 | 4.89E-38 |
| CARD11 | upregulated mRNA | -2.127625127 | 5.37E-38 |
| GPR174 | upregulated mRNA | -2.473670734 | 7.53E-38 |
| KPNA7 | upregulated mRNA | -3.419400114 | 7.63E-38 |
| EXOC3L4 | upregulated mRNA | -2.064222302 | 1.14E-37 |
| MUC16 | upregulated mRNA | -3.161033472 | 1.42E-37 |
| MEP1A | upregulated mRNA | -4.082157642 | 1.52E-37 |
| FAM153A | upregulated mRNA | -3.275190731 | 1.97E-37 |
| KIR3DX1 | upregulated mRNA | -2.52079817 | 2.71E-37 |
| C19orf33 | upregulated mRNA | -2.561619041 | 2.99E-37 |
| LRAT | upregulated mRNA | -2.300437455 | 3.17E-37 |
| KRTAP5-10 | upregulated mRNA | -2.769998545 | 3.94E-37 |
| CARD14 | upregulated mRNA | -2.663423374 | 6.00E-37 |
| KREMEN2 | upregulated mRNA | -2.826380356 | 6.32E-37 |
| NPIPB11 | upregulated mRNA | -2.374258016 | 6.66E-37 |
| PABPC1L | upregulated mRNA | -2.246011141 | 6.82E-37 |
| IGF2BP3 | upregulated mRNA | -3.741462942 | 9.28E-37 |
| RP11-434D12.1 | upregulated mRNA | -2.014613741 | 9.68E-37 |
| RP11-347C12.1 | upregulated mRNA | -3.159344475 | 1.03E-36 |
| ANLN | upregulated mRNA | -2.065838726 | 1.11E-36 |
| DMP1 | upregulated mRNA | -3.745896358 | 1.52E-36 |
| KCNK17 | upregulated mRNA | -3.530921471 | 2.03E-36 |
| TF | upregulated mRNA | -4.010566581 | 2.05E-36 |
| TREX2 | upregulated mRNA | -2.662419485 | 2.17E-36 |
| LHX8 | upregulated mRNA | -5.54134756 | 2.38E-36 |
| TIFAB | upregulated mRNA | -2.448949758 | 2.46E-36 |
| SLC26A10 | upregulated mRNA | -2.794762514 | 3.10E-36 |
| BHLHA15 | upregulated mRNA | -2.433492581 | 3.29E-36 |
| ABCB4 | upregulated mRNA | -2.123721488 | 3.82E-36 |
| PREX2 | upregulated mRNA | -2.081632567 | 3.82E-36 |
| RPA4 | upregulated mRNA | -2.884692428 | 4.43E-36 |
| CACNA1F | upregulated mRNA | -2.294649452 | 6.36E-36 |
| CYP21A2 | upregulated mRNA | -2.531033431 | 7.19E-36 |
| RGS20 | upregulated mRNA | -2.881419608 | 7.39E-36 |
| KCNT1 | upregulated mRNA | -2.399761898 | 8.01E-36 |
| HLA-DOB | upregulated mRNA | -2.193728508 | 1.04E-35 |
| P2RX3 | upregulated mRNA | -2.921628279 | 1.31E-35 |
| CCDC78 | upregulated mRNA | -2.88121719 | 1.31E-35 |
| DAND5 | upregulated mRNA | -2.760838483 | 1.88E-35 |
| RNASE10 | upregulated mRNA | -2.344545608 | 2.73E-35 |
| HORMAD1 | upregulated mRNA | -3.276071293 | 2.97E-35 |
| DCST2 | upregulated mRNA | -2.026806945 | 3.16E-35 |
| ARID3C | upregulated mRNA | -3.024177287 | 3.22E-35 |
| BEST4 | upregulated mRNA | -2.704597228 | 3.86E-35 |
| TM4SF19 | upregulated mRNA | -2.900174086 | 4.44E-35 |
| GPR84 | upregulated mRNA | -2.129433232 | 5.00E-35 |
| KIR3DL1 | upregulated mRNA | -2.215836576 | 7.27E-35 |
| GPC2 | upregulated mRNA | -2.004545192 | 8.34E-35 |
| PILRB | upregulated mRNA | -2.448504802 | 9.19E-35 |
| PRND | upregulated mRNA | -3.285654891 | 1.31E-34 |
| GP6 | upregulated mRNA | -2.274886726 | 1.37E-34 |
| IL9R | upregulated mRNA | -2.143847532 | 1.38E-34 |
| OMG | upregulated mRNA | -2.360326198 | 2.03E-34 |
| PLA2G2D | upregulated mRNA | -3.307779065 | 3.19E-34 |
| SPIC | upregulated mRNA | -3.850155343 | 3.25E-34 |
| CCR8 | upregulated mRNA | -2.811208566 | 3.42E-34 |
| RTP5 | upregulated mRNA | -3.124256381 | 5.72E-34 |
| CCER2 | upregulated mRNA | -2.662632823 | 6.62E-34 |
| FBXO41 | upregulated mRNA | -2.002054631 | 6.81E-34 |
| DCST1 | upregulated mRNA | -2.51561581 | 7.98E-34 |
| ADAMTS4 | upregulated mRNA | -2.007703338 | 8.82E-34 |
| LAX1 | upregulated mRNA | -2.04411932 | 1.00E-33 |
| SNAP25 | upregulated mRNA | -2.022699047 | 1.10E-33 |
| CSAG1 | upregulated mRNA | -3.582267187 | 1.20E-33 |
| SMC1B | upregulated mRNA | -2.330731975 | 1.30E-33 |
| GRIA4 | upregulated mRNA | -3.656639455 | 1.73E-33 |
| WNT10B | upregulated mRNA | -2.66012773 | 1.98E-33 |
| RDM1 | upregulated mRNA | -2.035444905 | 3.11E-33 |
| ZNF80 | upregulated mRNA | -2.623516434 | 3.52E-33 |
| CCR6 | upregulated mRNA | -2.591074498 | 3.79E-33 |
| ASIC3 | upregulated mRNA | -2.029645737 | 5.86E-33 |
| GPR35 | upregulated mRNA | -2.442805182 | 8.67E-33 |
| BAAT | upregulated mRNA | -3.220388929 | 1.14E-32 |
| C1orf61 | upregulated mRNA | -2.712763713 | 1.22E-32 |
| LCN1 | upregulated mRNA | -4.194511912 | 2.11E-32 |
| CYP3A5 | upregulated mRNA | -2.110294285 | 2.26E-32 |
| GOLGA6L2 | upregulated mRNA | -6.209576718 | 3.22E-32 |
| C17orf74 | upregulated mRNA | -3.665449286 | 3.24E-32 |
| ZAN | upregulated mRNA | -4.872077993 | 3.98E-32 |
| KIR3DL2 | upregulated mRNA | -2.388425548 | 4.02E-32 |
| BDNF | upregulated mRNA | -2.141272646 | 4.58E-32 |
| CHRND | upregulated mRNA | -3.46842792 | 6.00E-32 |
| AL008723.1 | upregulated mRNA | -3.760141491 | 9.65E-32 |
| KCNV2 | upregulated mRNA | -2.436104328 | 1.20E-31 |
| MGARP | upregulated mRNA | -2.98656812 | 1.51E-31 |
| CRP | upregulated mRNA | -4.416169525 | 1.62E-31 |
| KRT32 | upregulated mRNA | -3.790714422 | 2.11E-31 |
| WISP2 | upregulated mRNA | -3.361284356 | 2.23E-31 |
| DERL3 | upregulated mRNA | -2.188961919 | 2.55E-31 |
| NR2E1 | upregulated mRNA | -3.910200126 | 2.84E-31 |
| PRG2 | upregulated mRNA | -3.11662759 | 2.89E-31 |
| RP11-231C14.4 | upregulated mRNA | -2.149276056 | 3.10E-31 |
| C19orf84 | upregulated mRNA | -2.010045152 | 3.31E-31 |
| EFCAB3 | upregulated mRNA | -3.683623075 | 3.49E-31 |
| HIST1H2AM | upregulated mRNA | -2.73931644 | 4.09E-31 |
| IL2RA | upregulated mRNA | -2.224448604 | 4.33E-31 |
| ALK | upregulated mRNA | -2.279114544 | 4.49E-31 |
| LYZL1 | upregulated mRNA | -4.305392328 | 4.74E-31 |
| APOL1 | upregulated mRNA | -2.075123084 | 4.78E-31 |
| MUC5B | upregulated mRNA | -2.909285707 | 5.08E-31 |
| ARMC12 | upregulated mRNA | -2.051710029 | 8.24E-31 |
| ERMN | upregulated mRNA | -2.078982852 | 8.59E-31 |
| IL17REL | upregulated mRNA | -2.703436612 | 8.81E-31 |
| OR9Q1 | upregulated mRNA | -3.312296457 | 1.04E-30 |
| KRT72 | upregulated mRNA | -3.908073763 | 1.20E-30 |
| ANK1 | upregulated mRNA | -2.230674415 | 1.23E-30 |
| KLRC4-KLRK1 | upregulated mRNA | -2.315611912 | 1.23E-30 |
| MYH13 | upregulated mRNA | -4.7279609 | 1.26E-30 |
| FAM72C | upregulated mRNA | -2.381447632 | 1.55E-30 |
| GBX2 | upregulated mRNA | -3.349443744 | 2.34E-30 |
| CCDC154 | upregulated mRNA | -2.327668939 | 2.47E-30 |
| LA16c-431H6.6 | upregulated mRNA | -2.26078449 | 3.32E-30 |
| C4orf50 | upregulated mRNA | -2.51002113 | 4.33E-30 |
| VSX1 | upregulated mRNA | -3.231550046 | 4.89E-30 |
| YJEFN3 | upregulated mRNA | -2.121077289 | 5.44E-30 |
| AMH | upregulated mRNA | -2.595318117 | 6.13E-30 |
| SAA1 | upregulated mRNA | -4.326485619 | 6.21E-30 |
| SLC2A14 | upregulated mRNA | -2.409265889 | 6.79E-30 |
| CHST13 | upregulated mRNA | -2.011961319 | 7.22E-30 |
| C3orf22 | upregulated mRNA | -3.108878079 | 9.13E-30 |
| CPB1 | upregulated mRNA | -3.178456845 | 1.18E-29 |
| MT3 | upregulated mRNA | -3.359355317 | 1.44E-29 |
| DPEP3 | upregulated mRNA | -2.294466252 | 1.74E-29 |
| STX16-NPEPL1 | upregulated mRNA | -2.022259991 | 3.38E-29 |
| ANGPTL8 | upregulated mRNA | -3.270231552 | 3.49E-29 |
| TMEM92 | upregulated mRNA | -2.794625866 | 4.45E-29 |
| COL5A1 | upregulated mRNA | -2.045880018 | 5.45E-29 |
| RETN | upregulated mRNA | -2.688712216 | 6.75E-29 |
| HOXA13 | upregulated mRNA | -2.775218146 | 6.97E-29 |
| UTS2 | upregulated mRNA | -2.784551673 | 7.71E-29 |
| VNN3 | upregulated mRNA | -2.480865849 | 8.27E-29 |
| SIGLEC12 | upregulated mRNA | -2.617200174 | 9.08E-29 |
| NETO1 | upregulated mRNA | -2.548884726 | 9.23E-29 |
| TMPRSS6 | upregulated mRNA | -2.530948607 | 9.33E-29 |
| PRIMA1 | upregulated mRNA | -3.073613352 | 1.06E-28 |
| CARD17 | upregulated mRNA | -3.076033747 | 1.08E-28 |
| GOLGA8B | upregulated mRNA | -2.036394802 | 1.56E-28 |
| GPR150 | upregulated mRNA | -2.244706345 | 2.27E-28 |
| B4GALNT4 | upregulated mRNA | -3.214466701 | 2.36E-28 |
| TPBGL | upregulated mRNA | -2.362927611 | 2.73E-28 |
| HLA-DQA2 | upregulated mRNA | -2.467381773 | 2.85E-28 |
| C1QL4 | upregulated mRNA | -3.049533445 | 3.00E-28 |
| C2 | upregulated mRNA | -2.195821146 | 3.93E-28 |
| LHX2 | upregulated mRNA | -3.374715155 | 4.35E-28 |
| OTOG | upregulated mRNA | -2.229994008 | 4.72E-28 |
| RNASE3 | upregulated mRNA | -2.531269221 | 4.80E-28 |
| RPE65 | upregulated mRNA | -2.759468081 | 6.65E-28 |
| OBP2B | upregulated mRNA | -3.395631657 | 8.53E-28 |
| SEC14L3 | upregulated mRNA | -3.868712474 | 8.55E-28 |
| LRIT2 | upregulated mRNA | -4.151141303 | 1.06E-27 |
| PGLYRP2 | upregulated mRNA | -3.447131794 | 1.13E-27 |
| CADM3 | upregulated mRNA | -3.030583038 | 1.37E-27 |
| NPFFR1 | upregulated mRNA | -2.528474201 | 1.52E-27 |
| TRIM73 | upregulated mRNA | -2.062738959 | 1.93E-27 |
| PODNL1 | upregulated mRNA | -2.589098061 | 2.03E-27 |
| SPDEF | upregulated mRNA | -3.009737885 | 2.06E-27 |
| VIP | upregulated mRNA | -2.118536569 | 2.16E-27 |
| ZMAT4 | upregulated mRNA | -3.484025139 | 2.35E-27 |
| IGFN1 | upregulated mRNA | -4.153734096 | 2.89E-27 |
| OBP2A | upregulated mRNA | -3.824203292 | 2.93E-27 |
| GRM8 | upregulated mRNA | -2.035658415 | 3.00E-27 |
| CDR1 | upregulated mRNA | -3.374309926 | 3.80E-27 |
| CNPY1 | upregulated mRNA | -4.309588518 | 4.63E-27 |
| ANKRD33 | upregulated mRNA | -3.231365308 | 4.81E-27 |
| FGF20 | upregulated mRNA | -2.126628916 | 5.71E-27 |
| HIST1H2BL | upregulated mRNA | -2.678272011 | 6.18E-27 |
| EPS8L3 | upregulated mRNA | -3.589771459 | 6.96E-27 |
| CA1 | upregulated mRNA | -3.213343283 | 7.05E-27 |
| SLC2A7 | upregulated mRNA | -2.311271275 | 7.88E-27 |
| CALB2 | upregulated mRNA | -2.328866017 | 8.08E-27 |
| ADGRG2 | upregulated mRNA | -2.556149695 | 9.84E-27 |
| GSDMC | upregulated mRNA | -3.356724469 | 1.41E-26 |
| SERPINE1 | upregulated mRNA | -2.258873178 | 1.74E-26 |
| KCNK3 | upregulated mRNA | -2.167528884 | 1.97E-26 |
| LIM2 | upregulated mRNA | -3.214982263 | 1.98E-26 |
| KIAA0408 | upregulated mRNA | -2.194116769 | 2.17E-26 |
| CHI3L2 | upregulated mRNA | -2.328993598 | 2.18E-26 |
| FAM9C | upregulated mRNA | -2.11086054 | 2.36E-26 |
| ADCY2 | upregulated mRNA | -2.592704201 | 2.53E-26 |
| CTRC | upregulated mRNA | -2.964982433 | 2.71E-26 |
| LRRC14B | upregulated mRNA | -3.041112168 | 3.34E-26 |
| SHOX2 | upregulated mRNA | -2.379845441 | 4.76E-26 |
| ITIH4 | upregulated mRNA | -2.406857808 | 5.18E-26 |
| C15orf53 | upregulated mRNA | -3.196539687 | 5.27E-26 |
| PRSS37 | upregulated mRNA | -2.369967344 | 5.31E-26 |
| SAA2 | upregulated mRNA | -3.99806551 | 5.64E-26 |
| IGFBP1 | upregulated mRNA | -3.643230175 | 5.80E-26 |
| SLFN14 | upregulated mRNA | -2.585376194 | 6.98E-26 |
| KLRC2 | upregulated mRNA | -2.543132275 | 7.76E-26 |
| CREB3L3 | upregulated mRNA | -2.700709438 | 8.02E-26 |
| SPATA21 | upregulated mRNA | -2.970257233 | 8.34E-26 |
| MGAM2 | upregulated mRNA | -2.090874765 | 9.03E-26 |
| CGB7 | upregulated mRNA | -2.400106966 | 1.56E-25 |
| CRLF2 | upregulated mRNA | -2.364045386 | 1.71E-25 |
| CDSN | upregulated mRNA | -2.982413473 | 2.63E-25 |
| EPYC | upregulated mRNA | -3.675495943 | 2.72E-25 |
| KRT25 | upregulated mRNA | -4.677376435 | 3.40E-25 |
| RP5-1042K10.14 | upregulated mRNA | -2.24060089 | 3.97E-25 |
| GP9 | upregulated mRNA | -2.338487214 | 5.06E-25 |
| LGALS4 | upregulated mRNA | -3.46217467 | 5.15E-25 |
| GTSF1L | upregulated mRNA | -3.798263874 | 5.35E-25 |
| STMN2 | upregulated mRNA | -3.80141663 | 5.46E-25 |
| SLC5A5 | upregulated mRNA | -2.461523669 | 5.53E-25 |
| FRZB | upregulated mRNA | -2.042665488 | 5.64E-25 |
| HAVCR1 | upregulated mRNA | -2.195172029 | 6.28E-25 |
| CREG2 | upregulated mRNA | -2.032955555 | 7.07E-25 |
| POU5F2 | upregulated mRNA | -2.674209817 | 7.54E-25 |
| PLXNB3 | upregulated mRNA | -2.038921358 | 8.21E-25 |
| KLRC4 | upregulated mRNA | -2.349923956 | 8.24E-25 |
| SAP25 | upregulated mRNA | -2.395191227 | 9.23E-25 |
| REG1B | upregulated mRNA | -4.109010087 | 1.05E-24 |
| STRC | upregulated mRNA | -2.81336222 | 1.07E-24 |
| CIB4 | upregulated mRNA | -2.543928138 | 1.17E-24 |
| TREM1 | upregulated mRNA | -2.130190252 | 1.35E-24 |
| WFDC10B | upregulated mRNA | -3.724430197 | 1.57E-24 |
| CTSE | upregulated mRNA | -2.83848957 | 1.72E-24 |
| JSRP1 | upregulated mRNA | -2.33229044 | 2.08E-24 |
| TBC1D3L | upregulated mRNA | -2.265236363 | 3.17E-24 |
| CORO6 | upregulated mRNA | -2.176663412 | 4.19E-24 |
| RGSL1 | upregulated mRNA | -3.2699752 | 5.06E-24 |
| MCEMP1 | upregulated mRNA | -2.057461075 | 5.44E-24 |
| NMU | upregulated mRNA | -2.886977482 | 5.81E-24 |
| RP11-561B11.2 | upregulated mRNA | -2.048400673 | 5.93E-24 |
| CTC-360G5.8 | upregulated mRNA | -2.864105394 | 7.57E-24 |
| WNT1 | upregulated mRNA | -2.550678334 | 8.83E-24 |
| NPPA | upregulated mRNA | -2.050538555 | 9.14E-24 |
| SORCS3 | upregulated mRNA | -3.205064687 | 9.45E-24 |
| AZU1 | upregulated mRNA | -2.410252246 | 1.07E-23 |
| ORM2 | upregulated mRNA | -3.461322301 | 2.19E-23 |
| ATP1A3 | upregulated mRNA | -2.057809839 | 2.19E-23 |
| APOC2 | upregulated mRNA | -2.048227153 | 2.44E-23 |
| UGT1A1 | upregulated mRNA | -2.261944042 | 2.62E-23 |
| EFCAB8 | upregulated mRNA | -2.582665395 | 2.69E-23 |
| MARCO | upregulated mRNA | -2.385821381 | 2.73E-23 |
| TBC1D26 | upregulated mRNA | -3.019531284 | 2.75E-23 |
| SCN10A | upregulated mRNA | -3.659875283 | 3.27E-23 |
| PITX1 | upregulated mRNA | -3.679894853 | 3.42E-23 |
| NPIPA3 | upregulated mRNA | -2.492521173 | 3.43E-23 |
| TUBA3C | upregulated mRNA | -4.719349855 | 3.55E-23 |
| TBC1D3B | upregulated mRNA | -2.314141613 | 5.43E-23 |
| SMIM9 | upregulated mRNA | -3.300923463 | 5.75E-23 |
| CBLN4 | upregulated mRNA | -2.08205158 | 5.82E-23 |
| SPINK5 | upregulated mRNA | -2.146994624 | 6.45E-23 |
| TSPAN16 | upregulated mRNA | -2.456951318 | 6.70E-23 |
| IL12RB2 | upregulated mRNA | -2.625236071 | 9.77E-23 |
| HHATL | upregulated mRNA | -4.619129852 | 1.05E-22 |
| KIF4B | upregulated mRNA | -2.410676547 | 1.11E-22 |
| CTAGE6 | upregulated mRNA | -3.009378981 | 1.12E-22 |
| A3GALT2 | upregulated mRNA | -2.309050027 | 1.13E-22 |
| FAM209A | upregulated mRNA | -2.019667298 | 1.31E-22 |
| ASMT | upregulated mRNA | -2.076676091 | 2.00E-22 |
| DQX1 | upregulated mRNA | -2.965286468 | 2.06E-22 |
| PPP1R14D | upregulated mRNA | -2.674341297 | 2.29E-22 |
| TRIM74 | upregulated mRNA | -2.220127543 | 2.67E-22 |
| MMP13 | upregulated mRNA | -4.451276425 | 4.47E-22 |
| TSGA10IP | upregulated mRNA | -2.158310634 | 4.70E-22 |
| SST | upregulated mRNA | -3.640100608 | 6.46E-22 |
| COL11A1 | upregulated mRNA | -2.724274907 | 7.37E-22 |
| LHFPL5 | upregulated mRNA | -3.373314117 | 9.77E-22 |
| PRSS2 | upregulated mRNA | -3.069309888 | 1.07E-21 |
| SLC22A16 | upregulated mRNA | -2.120497854 | 1.17E-21 |
| MAP7D2 | upregulated mRNA | -2.406649563 | 1.17E-21 |
| TRIML2 | upregulated mRNA | -3.68265358 | 2.03E-21 |
| PRSS57 | upregulated mRNA | -2.24863723 | 2.45E-21 |
| CA6 | upregulated mRNA | -2.903710898 | 2.45E-21 |
| AQP8 | upregulated mRNA | -2.439841668 | 2.47E-21 |
| ZNF114 | upregulated mRNA | -2.495458998 | 2.90E-21 |
| HOXC12 | upregulated mRNA | -2.279806926 | 3.09E-21 |
| CPNE7 | upregulated mRNA | -2.263147523 | 3.51E-21 |
| HIST1H4J | upregulated mRNA | -2.248175236 | 3.79E-21 |
| POU4F1 | upregulated mRNA | -3.614202781 | 4.40E-21 |
| RIMBP3C | upregulated mRNA | -2.630695067 | 5.60E-21 |
| NXF5 | upregulated mRNA | -2.052656899 | 5.70E-21 |
| ADCY8 | upregulated mRNA | -3.242127368 | 6.25E-21 |
| PRAME | upregulated mRNA | -2.80348773 | 7.15E-21 |
| CLEC18A | upregulated mRNA | -2.067211383 | 7.48E-21 |
| CD1A | upregulated mRNA | -2.482070019 | 7.72E-21 |
| UGT1A10 | upregulated mRNA | -3.931904486 | 7.97E-21 |
| PADI3 | upregulated mRNA | -3.654419836 | 8.58E-21 |
| BARX1 | upregulated mRNA | -3.624668758 | 1.20E-20 |
| IL2 | upregulated mRNA | -2.59786614 | 1.23E-20 |
| MOGAT3 | upregulated mRNA | -2.261811903 | 1.27E-20 |
| PTX4 | upregulated mRNA | -2.190430391 | 1.39E-20 |
| PGA5 | upregulated mRNA | -2.369466325 | 1.56E-20 |
| FOXH1 | upregulated mRNA | -2.032802704 | 1.64E-20 |
| KLK14 | upregulated mRNA | -2.011655105 | 1.82E-20 |
| CCL25 | upregulated mRNA | -3.152101516 | 2.10E-20 |
| SAGE1 | upregulated mRNA | -3.645242213 | 2.51E-20 |
| NOG | upregulated mRNA | -2.534113281 | 2.65E-20 |
| RP4-576H24.4 | upregulated mRNA | -2.935370645 | 3.28E-20 |
| HIST1H2BH | upregulated mRNA | -2.165103978 | 3.38E-20 |
| AC005779.2 | upregulated mRNA | -2.674567811 | 4.39E-20 |
| GPR31 | upregulated mRNA | -2.276951262 | 4.43E-20 |
| SERPINA12 | upregulated mRNA | -4.093140236 | 5.16E-20 |
| MMP17 | upregulated mRNA | -2.053399263 | 5.19E-20 |
| MNX1 | upregulated mRNA | -2.522932943 | 6.44E-20 |
| KLRF2 | upregulated mRNA | -3.4190215 | 7.56E-20 |
| SULT1A3 | upregulated mRNA | -2.434611122 | 8.59E-20 |
| MS4A15 | upregulated mRNA | -3.38204332 | 9.30E-20 |
| KRT6A | upregulated mRNA | -4.362912403 | 1.11E-19 |
| PF4V1 | upregulated mRNA | -2.144233408 | 1.55E-19 |
| TMPRSS11A | upregulated mRNA | -3.928954908 | 1.55E-19 |
| IFITM5 | upregulated mRNA | -3.843810127 | 1.56E-19 |
| SYNGR4 | upregulated mRNA | -2.025121943 | 1.60E-19 |
| NKX2-5 | upregulated mRNA | -4.409478316 | 1.78E-19 |
| C10orf142 | upregulated mRNA | -2.296692166 | 1.88E-19 |
| FDCSP | upregulated mRNA | -5.198891801 | 1.88E-19 |
| LBP | upregulated mRNA | -3.420289914 | 2.56E-19 |
| ASIC4 | upregulated mRNA | -2.204413344 | 2.87E-19 |
| PRSS21 | upregulated mRNA | -2.285509171 | 3.17E-19 |
| HHIPL2 | upregulated mRNA | -2.486743904 | 3.43E-19 |
| HIST1H2BF | upregulated mRNA | -2.807745803 | 3.86E-19 |
| SYT12 | upregulated mRNA | -2.004673684 | 3.93E-19 |
| S100G | upregulated mRNA | -5.566346926 | 4.09E-19 |
| GPR25 | upregulated mRNA | -2.173493821 | 4.10E-19 |
| KRT14 | upregulated mRNA | -2.904056628 | 4.39E-19 |
| HOXB13 | upregulated mRNA | -3.734633181 | 4.63E-19 |
| IL26 | upregulated mRNA | -2.718885944 | 4.67E-19 |
| MROH2A | upregulated mRNA | -2.565876068 | 5.20E-19 |
| LGALS9B | upregulated mRNA | -2.10263406 | 8.75E-19 |
| HMHB1 | upregulated mRNA | -2.796545939 | 8.84E-19 |
| FGA | upregulated mRNA | -3.326056831 | 1.03E-18 |
| DMRT3 | upregulated mRNA | -4.329469837 | 1.06E-18 |
| AC007326.1 | upregulated mRNA | -3.192407885 | 1.11E-18 |
| SCN1A | upregulated mRNA | -2.132747642 | 1.13E-18 |
| KLRC3 | upregulated mRNA | -2.34037843 | 1.26E-18 |
| GNGT1 | upregulated mRNA | -2.257866815 | 1.41E-18 |
| STH | upregulated mRNA | -3.188177515 | 1.78E-18 |
| MAGEA10 | upregulated mRNA | -3.670915582 | 1.92E-18 |
| TNNT3 | upregulated mRNA | -2.362369456 | 2.17E-18 |
| ARX | upregulated mRNA | -3.564600678 | 3.59E-18 |
| NLRP7 | upregulated mRNA | -2.172600706 | 5.36E-18 |
| SLC1A7 | upregulated mRNA | -2.169537862 | 7.25E-18 |
| GAD2 | upregulated mRNA | -2.858340865 | 7.64E-18 |
| PRTN3 | upregulated mRNA | -2.705498911 | 7.66E-18 |
| RSPO4 | upregulated mRNA | -2.220088437 | 7.90E-18 |
| RP11-385D13.1 | upregulated mRNA | -2.359376555 | 8.81E-18 |
| AC110602.1 | upregulated mRNA | -2.141186654 | 9.16E-18 |
| TNFSF11 | upregulated mRNA | -2.15218111 | 1.37E-17 |
| PTPRH | upregulated mRNA | -2.565331757 | 1.41E-17 |
| TPSG1 | upregulated mRNA | -2.490569794 | 1.47E-17 |
| ORM1 | upregulated mRNA | -3.432525858 | 1.73E-17 |
| MUC22 | upregulated mRNA | -2.668001617 | 1.73E-17 |
| CPA4 | upregulated mRNA | -2.662973266 | 2.41E-17 |
| SMIM23 | upregulated mRNA | -3.157442031 | 2.53E-17 |
| HIST1H4E | upregulated mRNA | -2.045862936 | 2.54E-17 |
| UROC1 | upregulated mRNA | -2.060761726 | 2.64E-17 |
| CEACAM20 | upregulated mRNA | -3.116563576 | 3.03E-17 |
| CALML6 | upregulated mRNA | -2.151148527 | 3.91E-17 |
| SNCB | upregulated mRNA | -3.273452289 | 4.22E-17 |
| PI3 | upregulated mRNA | -2.791823565 | 4.23E-17 |
| CHAT | upregulated mRNA | -5.236137321 | 4.59E-17 |
| SEZ6L | upregulated mRNA | -2.040604013 | 4.95E-17 |
| UGT1A4 | upregulated mRNA | -3.240552341 | 5.37E-17 |
| MROH9 | upregulated mRNA | -2.905681829 | 5.42E-17 |
| FAM9B | upregulated mRNA | -2.48637998 | 5.83E-17 |
| ISL2 | upregulated mRNA | -2.578098589 | 6.65E-17 |
| SAA4 | upregulated mRNA | -3.652355781 | 6.72E-17 |
| SERPINA9 | upregulated mRNA | -3.532601626 | 6.87E-17 |
| SLC35G3 | upregulated mRNA | -3.727151952 | 7.06E-17 |
| CRX | upregulated mRNA | -2.942180383 | 8.03E-17 |
| LRRTM4 | upregulated mRNA | -2.468946764 | 9.27E-17 |
| HIST1H2BO | upregulated mRNA | -2.164745356 | 9.93E-17 |
| C6orf141 | upregulated mRNA | -2.152467252 | 1.28E-16 |
| CBLN1 | upregulated mRNA | -2.101703497 | 1.33E-16 |
| DPYSL4 | upregulated mRNA | -2.220297327 | 1.37E-16 |
| PPEF2 | upregulated mRNA | -2.079293303 | 1.42E-16 |
| CPLX2 | upregulated mRNA | -3.639650277 | 1.43E-16 |
| MS4A6E | upregulated mRNA | -2.566580106 | 1.75E-16 |
| SAG | upregulated mRNA | -2.367905273 | 2.34E-16 |
| SAA2-SAA4 | upregulated mRNA | -4.280295816 | 2.34E-16 |
| ADAM29 | upregulated mRNA | -2.19723627 | 2.42E-16 |
| AGBL1 | upregulated mRNA | -3.445013291 | 2.52E-16 |
| CATSPERD | upregulated mRNA | -3.394573317 | 2.53E-16 |
| GRM4 | upregulated mRNA | -2.023390568 | 2.74E-16 |
| ONECUT3 | upregulated mRNA | -2.457275991 | 2.93E-16 |
| GS1-393G12.13 | upregulated mRNA | -2.47825521 | 3.06E-16 |
| C11orf86 | upregulated mRNA | -2.501273039 | 3.57E-16 |
| DMBX1 | upregulated mRNA | -3.544736327 | 3.64E-16 |
| MYOD1 | upregulated mRNA | -3.626827596 | 4.08E-16 |
| MMP1 | upregulated mRNA | -2.101660839 | 4.30E-16 |
| REG1A | upregulated mRNA | -2.674453013 | 5.22E-16 |
| SLC5A1 | upregulated mRNA | -2.114306426 | 5.36E-16 |
| TMEM249 | upregulated mRNA | -2.413087387 | 6.51E-16 |
| KLF1 | upregulated mRNA | -2.219350717 | 8.11E-16 |
| SLC6A7 | upregulated mRNA | -2.005625943 | 1.31E-15 |
| FAM9A | upregulated mRNA | -3.423200085 | 1.37E-15 |
| HBG2 | upregulated mRNA | -2.031164606 | 1.56E-15 |
| TGM5 | upregulated mRNA | -2.489243192 | 1.65E-15 |
| KRT73 | upregulated mRNA | -2.385449026 | 2.02E-15 |
| ENTHD1 | upregulated mRNA | -2.215592405 | 2.06E-15 |
| KLK4 | upregulated mRNA | -3.989569381 | 2.13E-15 |
| NPY4R | upregulated mRNA | -2.825815327 | 2.37E-15 |
| SLX1B | upregulated mRNA | -2.4685515 | 2.58E-15 |
| ETV3L | upregulated mRNA | -2.809823571 | 3.10E-15 |
| IFNL1 | upregulated mRNA | -2.62995571 | 3.10E-15 |
| TREML4 | upregulated mRNA | -2.603530267 | 3.29E-15 |
| KRT84 | upregulated mRNA | -2.880763937 | 3.52E-15 |
| KERA | upregulated mRNA | -3.442374218 | 3.52E-15 |
| PKP1 | upregulated mRNA | -2.118216271 | 3.57E-15 |
| CST2 | upregulated mRNA | -2.463847172 | 3.75E-15 |
| ZIC5 | upregulated mRNA | -3.689995917 | 4.20E-15 |
| AC010287.1 | upregulated mRNA | -2.536570961 | 4.72E-15 |
| AC016549.1 | upregulated mRNA | -2.527977164 | 4.89E-15 |
| SULT4A1 | upregulated mRNA | -2.552911614 | 5.15E-15 |
| C4orf26 | upregulated mRNA | -2.106998129 | 5.46E-15 |
| NIPAL4 | upregulated mRNA | -2.213522915 | 5.50E-15 |
| CALY | upregulated mRNA | -2.190783919 | 6.32E-15 |
| SLN | upregulated mRNA | -2.94683394 | 6.79E-15 |
| CELA1 | upregulated mRNA | -2.263995052 | 6.98E-15 |
| PADI6 | upregulated mRNA | -2.85408606 | 7.16E-15 |
| RPTN | upregulated mRNA | -4.079872969 | 7.16E-15 |
| IGLL5 | upregulated mRNA | -2.207982765 | 8.19E-15 |
| SBSN | upregulated mRNA | -3.40464946 | 1.17E-14 |
| OR56B1 | upregulated mRNA | -2.778650052 | 1.22E-14 |
| ODF3 | upregulated mRNA | -2.759606618 | 1.28E-14 |
| CHRNG | upregulated mRNA | -2.301894809 | 1.58E-14 |
| KRT78 | upregulated mRNA | -3.232028609 | 1.63E-14 |
| FOXL2NB | upregulated mRNA | -3.085752554 | 1.93E-14 |
| PPP2R2C | upregulated mRNA | -2.24567378 | 1.94E-14 |
| SOX1 | upregulated mRNA | -4.189450349 | 2.14E-14 |
| PRKCG | upregulated mRNA | -2.464703594 | 2.14E-14 |
| ZIC2 | upregulated mRNA | -3.063647705 | 2.65E-14 |
| C14orf180 | upregulated mRNA | -2.460996114 | 3.13E-14 |
| CDHR4 | upregulated mRNA | -2.302672137 | 3.58E-14 |
| UGT1A8 | upregulated mRNA | -2.801437866 | 3.71E-14 |
| GATA4 | upregulated mRNA | -3.370491875 | 4.16E-14 |
| PRR25 | upregulated mRNA | -2.296942896 | 4.20E-14 |
| TCN1 | upregulated mRNA | -2.586147963 | 4.65E-14 |
| BECN2 | upregulated mRNA | -3.704857574 | 5.21E-14 |
| FGB | upregulated mRNA | -3.075406479 | 5.59E-14 |
| WFDC13 | upregulated mRNA | -2.658376666 | 6.09E-14 |
| LYPD4 | upregulated mRNA | -3.818941734 | 6.48E-14 |
| IL37 | upregulated mRNA | -2.599878582 | 7.24E-14 |
| FCRL5 | upregulated mRNA | -2.049634499 | 1.07E-13 |
| DMRT1 | upregulated mRNA | -3.1702639 | 1.11E-13 |
| GPR15 | upregulated mRNA | -2.138315519 | 1.19E-13 |
| SLC30A10 | upregulated mRNA | -3.033053336 | 1.23E-13 |
| SOX21 | upregulated mRNA | -2.369608404 | 1.31E-13 |
| MAGEA12 | upregulated mRNA | -2.326020582 | 1.41E-13 |
| CDK5R2 | upregulated mRNA | -2.488409081 | 1.50E-13 |
| OR2B6 | upregulated mRNA | -2.109067971 | 1.64E-13 |
| IFNK | upregulated mRNA | -2.872128825 | 2.01E-13 |
| REG3A | upregulated mRNA | -3.900663688 | 2.07E-13 |
| C12orf40 | upregulated mRNA | -2.96258891 | 2.08E-13 |
| ACTL6B | upregulated mRNA | -3.181722052 | 2.10E-13 |
| PRAC2 | upregulated mRNA | -3.933056409 | 2.13E-13 |
| SYT8 | upregulated mRNA | -2.623768948 | 2.21E-13 |
| AKR1D1 | upregulated mRNA | -2.409890664 | 2.32E-13 |
| RNF113B | upregulated mRNA | -2.394083243 | 3.27E-13 |
| FFAR3 | upregulated mRNA | -2.139738692 | 3.35E-13 |
| NKX2-3 | upregulated mRNA | -3.155224638 | 3.64E-13 |
| HIST1H1T | upregulated mRNA | -2.015329634 | 3.83E-13 |
| UGT1A5 | upregulated mRNA | -2.811522572 | 3.92E-13 |
| MOG | upregulated mRNA | -3.404015537 | 4.40E-13 |
| HIST1H2BM | upregulated mRNA | -3.379767436 | 4.72E-13 |
| MBL2 | upregulated mRNA | -3.772718325 | 5.05E-13 |
| HIST1H3J | upregulated mRNA | -2.482437297 | 5.05E-13 |
| KRT20 | upregulated mRNA | -2.987300321 | 5.33E-13 |
| OLIG2 | upregulated mRNA | -2.834671987 | 5.59E-13 |
| TM4SF19-TCTEX1D2 | upregulated mRNA | -2.398084883 | 5.84E-13 |
| ACTL8 | upregulated mRNA | -4.304605882 | 5.87E-13 |
| LINC01620 | upregulated mRNA | -2.411266692 | 6.09E-13 |
| BPIFB1 | upregulated mRNA | -2.727330964 | 6.83E-13 |
| THEG | upregulated mRNA | -3.006591561 | 7.82E-13 |
| PRLHR | upregulated mRNA | -2.797734244 | 7.91E-13 |
| CSRP3 | upregulated mRNA | -2.73411419 | 8.19E-13 |
| AC024361.1 | upregulated mRNA | -2.14120067 | 8.26E-13 |
| PAGE2B | upregulated mRNA | -2.41291274 | 1.16E-12 |
| RTL1 | upregulated mRNA | -4.350001328 | 1.16E-12 |
| MAGEC3 | upregulated mRNA | -2.675141651 | 1.22E-12 |
| BRDT | upregulated mRNA | -2.328622033 | 1.23E-12 |
| OVOL3 | upregulated mRNA | -2.047667602 | 1.26E-12 |
| C22orf42 | upregulated mRNA | -2.245780193 | 1.95E-12 |
| HIST1H3B | upregulated mRNA | -2.946666578 | 2.00E-12 |
| ZNF705A | upregulated mRNA | -2.104066714 | 2.21E-12 |
| ALPI | upregulated mRNA | -2.446031037 | 3.03E-12 |
| KCNA7 | upregulated mRNA | -2.013367789 | 3.32E-12 |
| PSG1 | upregulated mRNA | -2.853765908 | 4.80E-12 |
| CCDC177 | upregulated mRNA | -2.501557195 | 5.74E-12 |
| XKR3 | upregulated mRNA | -2.105946142 | 6.47E-12 |
| GLYATL1P3 | upregulated mRNA | -2.323838433 | 6.59E-12 |
| IQCA1L | upregulated mRNA | -2.608008426 | 7.47E-12 |
| GALNTL5 | upregulated mRNA | -3.04456656 | 7.62E-12 |
| C8orf74 | upregulated mRNA | -2.943381424 | 9.30E-12 |
| RLBP1 | upregulated mRNA | -2.711038976 | 1.06E-11 |
| GPR42 | upregulated mRNA | -2.760328629 | 1.12E-11 |
| RP4-777O23.3 | upregulated mRNA | -2.027490362 | 1.28E-11 |
| PTF1A | upregulated mRNA | -2.184830056 | 2.07E-11 |
| KRT33A | upregulated mRNA | -2.917420031 | 2.32E-11 |
| KLK13 | upregulated mRNA | -2.316028584 | 2.63E-11 |
| CELA2A | upregulated mRNA | -2.011054949 | 2.77E-11 |
| KRT33B | upregulated mRNA | -2.406025555 | 2.97E-11 |
| ATP4A | upregulated mRNA | -2.703986182 | 3.48E-11 |
| KCNU1 | upregulated mRNA | -3.721298573 | 3.53E-11 |
| CST1 | upregulated mRNA | -2.587569888 | 3.97E-11 |
| NKX6-3 | upregulated mRNA | -2.824481733 | 4.07E-11 |
| ABCB5 | upregulated mRNA | -2.065216597 | 4.26E-11 |
| DDI1 | upregulated mRNA | -2.362316392 | 4.27E-11 |
| SLX1A | upregulated mRNA | -2.101121138 | 5.29E-11 |
| ASCL1 | upregulated mRNA | -2.709118224 | 6.65E-11 |
| TEX13B | upregulated mRNA | -2.646686613 | 6.75E-11 |
| MLN | upregulated mRNA | -2.318938665 | 7.18E-11 |
| SLC1A6 | upregulated mRNA | -2.466323267 | 8.26E-11 |
| F13B | upregulated mRNA | -2.64978381 | 8.77E-11 |
| OTP | upregulated mRNA | -2.329479396 | 9.54E-11 |
| SULT2A1 | upregulated mRNA | -3.060269226 | 9.69E-11 |
| HIST3H3 | upregulated mRNA | -2.040214596 | 1.05E-10 |
| FOXB1 | upregulated mRNA | -2.222825589 | 1.10E-10 |
| AIPL1 | upregulated mRNA | -2.534915127 | 1.10E-10 |
| KRTAP29-1 | upregulated mRNA | -2.235968508 | 1.32E-10 |
| TBPL2 | upregulated mRNA | -2.323230332 | 1.32E-10 |
| SP7 | upregulated mRNA | -2.064031147 | 1.53E-10 |
| AHSP | upregulated mRNA | -2.103090238 | 1.58E-10 |
| OR1G1 | upregulated mRNA | -2.322604835 | 1.59E-10 |
| MAGEA3 | upregulated mRNA | -4.062043716 | 1.82E-10 |
| CACNG2 | upregulated mRNA | -3.083146968 | 2.11E-10 |
| PHGR1 | upregulated mRNA | -2.364990474 | 2.23E-10 |
| HIST1H1B | upregulated mRNA | -2.788540156 | 2.39E-10 |
| ANKRD62 | upregulated mRNA | -2.264530321 | 2.39E-10 |
| OR2H1 | upregulated mRNA | -3.077105744 | 2.54E-10 |
| PIP | upregulated mRNA | -2.945431633 | 2.76E-10 |
| MMP12 | upregulated mRNA | -2.139581952 | 2.96E-10 |
| TMPRSS11F | upregulated mRNA | -2.393707616 | 3.20E-10 |
| KRT6B | upregulated mRNA | -3.262739803 | 3.22E-10 |
| BEST2 | upregulated mRNA | -2.219671157 | 3.74E-10 |
| AC092384.1 | upregulated mRNA | -2.056393652 | 3.91E-10 |
| PAGE2 | upregulated mRNA | -3.116252586 | 4.20E-10 |
| RHAG | upregulated mRNA | -2.584112827 | 4.69E-10 |
| PROP1 | upregulated mRNA | -2.884801387 | 5.31E-10 |
| MSLNL | upregulated mRNA | -2.842552754 | 5.61E-10 |
| LA16c-380H5.3 | upregulated mRNA | -2.137818327 | 5.92E-10 |
| C7orf33 | upregulated mRNA | -2.815964879 | 6.00E-10 |
| LHX9 | upregulated mRNA | -2.720124018 | 6.45E-10 |
| C20orf173 | upregulated mRNA | -2.440841037 | 7.45E-10 |
| OR2AT4 | upregulated mRNA | -3.18563468 | 8.80E-10 |
| FCRL4 | upregulated mRNA | -2.721933767 | 9.66E-10 |
| INSM2 | upregulated mRNA | -2.253958529 | 1.12E-09 |
| SLAMF9 | upregulated mRNA | -2.002366523 | 1.40E-09 |
| NANOS2 | upregulated mRNA | -3.1239742 | 1.41E-09 |
| DSPP | upregulated mRNA | -2.39109139 | 1.52E-09 |
| LRRC74B | upregulated mRNA | -2.15988628 | 1.64E-09 |
| SERPINA11 | upregulated mRNA | -2.652420121 | 1.95E-09 |
| HIST1H3F | upregulated mRNA | -2.563128424 | 1.98E-09 |
| HIST1H2AJ | upregulated mRNA | -2.706747789 | 1.99E-09 |
| KHDC1L | upregulated mRNA | -2.185334181 | 2.11E-09 |
| GLRA1 | upregulated mRNA | -2.072275645 | 2.22E-09 |
| RP11-166N6.3 | upregulated mRNA | -2.263549094 | 2.24E-09 |
| SPDYE16 | upregulated mRNA | -2.059528462 | 2.42E-09 |
| SLC36A3 | upregulated mRNA | -2.025133243 | 2.61E-09 |
| KRTAP5-11 | upregulated mRNA | -2.142431712 | 3.30E-09 |
| KRTAP16-1 | upregulated mRNA | -2.216455061 | 3.54E-09 |
| ADGRF4 | upregulated mRNA | -2.036811578 | 3.76E-09 |
| C3orf84 | upregulated mRNA | -2.338328968 | 3.93E-09 |
| FIGLA | upregulated mRNA | -2.046463526 | 4.07E-09 |
| ADGRG7 | upregulated mRNA | -2.063020497 | 4.13E-09 |
| C2orf83 | upregulated mRNA | -2.859612696 | 4.37E-09 |
| NR5A1 | upregulated mRNA | -2.36174398 | 4.54E-09 |
| FOXB2 | upregulated mRNA | -2.708659427 | 5.00E-09 |
| CYP2F1 | upregulated mRNA | -2.143974075 | 5.20E-09 |
| TRIML1 | upregulated mRNA | -2.071899966 | 5.23E-09 |
| TLX1 | upregulated mRNA | -2.585542251 | 5.66E-09 |
| DMRTC2 | upregulated mRNA | -3.210886518 | 5.74E-09 |
| FGF21 | upregulated mRNA | -2.885402126 | 5.80E-09 |
| FAM71C | upregulated mRNA | -2.027247212 | 6.14E-09 |
| SPPL2C | upregulated mRNA | -2.352266223 | 7.30E-09 |
| PSG6 | upregulated mRNA | -3.136198788 | 7.45E-09 |
| ZSCAN10 | upregulated mRNA | -2.144312511 | 7.50E-09 |
| AC243756.1 | upregulated mRNA | -2.084011352 | 7.58E-09 |
| TNNI3 | upregulated mRNA | -2.427931751 | 7.69E-09 |
| TBX20 | upregulated mRNA | -2.479580974 | 7.79E-09 |
| ZIC4 | upregulated mRNA | -2.223715992 | 8.01E-09 |
| ADH7 | upregulated mRNA | -2.985480072 | 8.03E-09 |
| GOLGA6B | upregulated mRNA | -2.11862981 | 8.12E-09 |
| KRT4 | upregulated mRNA | -2.397929107 | 8.19E-09 |
| SIGLECL1 | upregulated mRNA | -2.438972139 | 8.58E-09 |
| DSG3 | upregulated mRNA | -2.373707845 | 8.92E-09 |
| PSG3 | upregulated mRNA | -2.994920501 | 9.27E-09 |
| TMEM14EP | upregulated mRNA | -2.28086695 | 1.04E-08 |
| PANX3 | upregulated mRNA | -2.457075826 | 1.43E-08 |
| PABPN1L | upregulated mRNA | -2.052837238 | 1.48E-08 |
| SEPT12 | upregulated mRNA | -2.147635594 | 1.70E-08 |
| WNT8A | upregulated mRNA | -2.16774934 | 1.71E-08 |
| CRNN | upregulated mRNA | -2.189827866 | 1.96E-08 |
| SERPINB13 | upregulated mRNA | -2.636555991 | 2.10E-08 |
| FOXR1 | upregulated mRNA | -2.128596133 | 2.41E-08 |
| HIST1H2AH | upregulated mRNA | -2.147246547 | 2.52E-08 |
| ZNF716 | upregulated mRNA | -2.388813342 | 2.94E-08 |
| PNPLA5 | upregulated mRNA | -2.860034829 | 2.98E-08 |
| GIP | upregulated mRNA | -2.908634432 | 3.31E-08 |
| SPRR3 | upregulated mRNA | -3.14510114 | 3.67E-08 |
| AL591806.1 | upregulated mRNA | -2.214503555 | 3.70E-08 |
| GAPDHS | upregulated mRNA | -2.182597806 | 3.76E-08 |
| NLRP13 | upregulated mRNA | -2.745238585 | 3.81E-08 |
| OR2H2 | upregulated mRNA | -2.065976737 | 3.90E-08 |
| PDCL2 | upregulated mRNA | -2.822302706 | 4.39E-08 |
| C9orf57 | upregulated mRNA | -2.301497932 | 4.78E-08 |
| TEX19 | upregulated mRNA | -2.171625631 | 4.93E-08 |
| DEFB118 | upregulated mRNA | -2.757894 | 5.69E-08 |
| NBPF4 | upregulated mRNA | -2.372958628 | 5.73E-08 |
| CIB3 | upregulated mRNA | -2.142367772 | 6.58E-08 |
| CLRN1 | upregulated mRNA | -2.160590996 | 6.63E-08 |
| PSG2 | upregulated mRNA | -2.661883532 | 7.19E-08 |
| PGA4 | upregulated mRNA | -2.392564567 | 8.32E-08 |
| CATSPER4 | upregulated mRNA | -2.686100984 | 9.71E-08 |
| TBC1D3E | upregulated mRNA | -2.317199888 | 1.22E-07 |
| SEPT14 | upregulated mRNA | -2.109358001 | 1.28E-07 |
| OR11A1 | upregulated mRNA | -2.519077785 | 1.32E-07 |
| CABP5 | upregulated mRNA | -2.330533075 | 1.35E-07 |
| OR5C1 | upregulated mRNA | -2.45180087 | 1.46E-07 |
| ST8SIA3 | upregulated mRNA | -2.447084949 | 1.57E-07 |
| KLK2 | upregulated mRNA | -2.291319401 | 1.65E-07 |
| OR9A4 | upregulated mRNA | -2.135095455 | 1.75E-07 |
| AADAC | upregulated mRNA | -2.049567586 | 1.88E-07 |
| KRT77 | upregulated mRNA | -2.745361409 | 1.92E-07 |
| CTC-479C5.6 | upregulated mRNA | -2.223787412 | 1.96E-07 |
| IRGC | upregulated mRNA | -2.212683196 | 2.31E-07 |
| NLRP5 | upregulated mRNA | -2.493722771 | 2.62E-07 |
| TEX101 | upregulated mRNA | -2.025308744 | 2.79E-07 |
| HBM | upregulated mRNA | -2.223438303 | 2.89E-07 |
| C20orf141 | upregulated mRNA | -2.602684096 | 3.20E-07 |
| RFX6 | upregulated mRNA | -2.065526339 | 3.29E-07 |
| MSMB | upregulated mRNA | -2.438368511 | 3.54E-07 |
| IGFL3 | upregulated mRNA | -2.223568152 | 3.76E-07 |
| SPATA31D1 | upregulated mRNA | -2.353457695 | 4.63E-07 |
| AMELX | upregulated mRNA | -2.308965615 | 4.91E-07 |
| HIST1H2BI | upregulated mRNA | -2.316455453 | 5.21E-07 |
| SOHLH1 | upregulated mRNA | -2.313728986 | 5.25E-07 |
| KRT75 | upregulated mRNA | -2.848607769 | 5.48E-07 |
| OR1K1 | upregulated mRNA | -2.003797146 | 6.93E-07 |
| TMEM225 | upregulated mRNA | -2.517782195 | 8.40E-07 |
| LMAN1L | upregulated mRNA | -2.423817447 | 9.26E-07 |
| ANKRD30BL | upregulated mRNA | -2.28594619 | 1.29E-06 |
| ZNF280A | upregulated mRNA | -2.57954333 | 1.43E-06 |
| TBC1D3D | upregulated mRNA | -3.015113983 | 1.49E-06 |
| DEFA3 | upregulated mRNA | -2.024293763 | 1.72E-06 |
| SP8 | upregulated mRNA | -2.817027191 | 1.80E-06 |
| APOA4 | upregulated mRNA | -3.485223092 | 2.20E-06 |
| KRT82 | upregulated mRNA | -2.195296564 | 2.39E-06 |
| KLK12 | upregulated mRNA | -2.658374776 | 2.42E-06 |
| TFAP2D | upregulated mRNA | -2.549498786 | 2.65E-06 |
| CYLC2 | upregulated mRNA | -2.26648221 | 3.56E-06 |
| ZNF705G | upregulated mRNA | -2.75411395 | 3.58E-06 |
| OR10A2 | upregulated mRNA | -2.105900408 | 3.62E-06 |
| HIST1H2AL | upregulated mRNA | -2.148012603 | 4.21E-06 |
| TMEM239 | upregulated mRNA | -2.051746605 | 4.30E-06 |
| SI | upregulated mRNA | -2.280350451 | 5.50E-06 |
| OR52H1 | upregulated mRNA | -2.112650291 | 6.05E-06 |
| NLRP8 | upregulated mRNA | -2.184274404 | 6.23E-06 |
| PRSS38 | upregulated mRNA | -2.397086513 | 8.25E-06 |
| CYP2A13 | upregulated mRNA | -2.23038668 | 9.30E-06 |
| DNTT | upregulated mRNA | -2.073344402 | 1.01E-05 |
| SPATA3 | upregulated mRNA | -2.164646345 | 1.27E-05 |
| KCNK16 | upregulated mRNA | -2.005790207 | 1.30E-05 |
| PTH | upregulated mRNA | -2.483440982 | 1.49E-05 |
| PSG8 | upregulated mRNA | -2.550437042 | 1.75E-05 |
| HIST1H4F | upregulated mRNA | -2.700820764 | 1.76E-05 |
| BARHL2 | upregulated mRNA | -2.082268115 | 1.84E-05 |
| NTSR2 | upregulated mRNA | -2.219461304 | 1.86E-05 |
| DPRX | upregulated mRNA | -2.040199599 | 2.00E-05 |
| C16orf92 | upregulated mRNA | -2.037396663 | 2.05E-05 |
| PSG11 | upregulated mRNA | -2.233273841 | 2.33E-05 |
| LGALS14 | upregulated mRNA | -2.111847131 | 2.38E-05 |
| PRG3 | upregulated mRNA | -2.306239661 | 2.55E-05 |
| GALP | upregulated mRNA | -2.461478047 | 2.58E-05 |
| DPPA3 | upregulated mRNA | -2.162027742 | 2.64E-05 |
| RP11-546B8.6 | upregulated mRNA | -2.401487505 | 2.93E-05 |
| OPALIN | upregulated mRNA | -2.491230185 | 3.70E-05 |
| GLT6D1 | upregulated mRNA | -2.121184703 | 3.99E-05 |
| PRR27 | upregulated mRNA | -2.081259474 | 4.38E-05 |
| NEUROD4 | upregulated mRNA | -2.6032508 | 4.48E-05 |
| PRR30 | upregulated mRNA | -2.371704252 | 5.37E-05 |
| ADAD1 | upregulated mRNA | -2.16987558 | 6.43E-05 |
| MAGEB2 | upregulated mRNA | -3.113200563 | 8.12E-05 |
| CCDC105 | upregulated mRNA | -2.139277552 | 9.38E-05 |
| GPR32 | upregulated mRNA | -2.157090698 | 9.52E-05 |
| GABRG2 | upregulated mRNA | -2.304278795 | 9.82E-05 |
| NR0B1 | upregulated mRNA | -2.290885032 | 9.88E-05 |
| NKX2-8 | upregulated mRNA | -2.305360031 | 0.000107352 |
| OR10W1 | upregulated mRNA | -2.057893479 | 0.000128339 |
| HIST1H3I | upregulated mRNA | -2.352858298 | 0.00015032 |
| BPIFB2 | upregulated mRNA | -2.444065316 | 0.000174035 |
| SSX1 | upregulated mRNA | -2.280726546 | 0.00021214 |
| TGM6 | upregulated mRNA | -2.055607117 | 0.000213156 |
| OR6S1 | upregulated mRNA | -2.10543209 | 0.000267714 |
| OR4K2 | upregulated mRNA | -2.113803589 | 0.000272693 |
| CALML5 | upregulated mRNA | -2.515797414 | 0.000276225 |
| TAAR5 | upregulated mRNA | -2.021508055 | 0.000281684 |
| OR5A1 | upregulated mRNA | -2.046439657 | 0.000432702 |
| PRAMEF17 | upregulated mRNA | -2.035571478 | 0.000673509 |
| HBG1 | upregulated mRNA | -2.766218021 | 0.000825777 |
| MAGEA6 | upregulated mRNA | -2.128748951 | 0.001335665 |
| MAGEC2 | upregulated mRNA | -2.806492569 | 0.001453551 |
| PASD1 | upregulated mRNA | -2.525985712 | 0.002197392 |
| LIN28B | upregulated mRNA | -2.264630672 | 0.002540884 |
| KRTAP10-12 | upregulated mRNA | -2.200033096 | 0.003469256 |
| RHOXF2 | upregulated mRNA | -2.03265105 | 0.003908681 |
| AJ239318.1 | upregulated mRNA | -2.717666145 | 0.007414157 |
| MFSD4 | downregulated mRNA | 5.23797978 | 0 |
| ACPP | downregulated mRNA | 5.576031647 | 1.14E-237 |
| SIM2 | downregulated mRNA | 4.490085384 | 3.26E-201 |
| ATP1A1 | downregulated mRNA | 2.762508013 | 9.53E-179 |
| ADGRF3 | downregulated mRNA | 3.530232382 | 6.96E-168 |
| ESRRB | downregulated mRNA | 5.915375329 | 1.16E-166 |
| MYLK3 | downregulated mRNA | 4.04973752 | 2.97E-162 |
| GPC5 | downregulated mRNA | 5.834782072 | 2.63E-159 |
| KCNJ10 | downregulated mRNA | 6.178701455 | 4.55E-155 |
| HSPA2 | downregulated mRNA | 3.882982847 | 4.69E-151 |
| CALB1 | downregulated mRNA | 7.517594901 | 3.79E-150 |
| MTURN | downregulated mRNA | 3.039532491 | 5.65E-149 |
| DDN | downregulated mRNA | 6.361253625 | 1.78E-148 |
| ELF5 | downregulated mRNA | 7.814254972 | 5.80E-147 |
| ENPP6 | downregulated mRNA | 5.04709315 | 1.25E-143 |
| IRX2 | downregulated mRNA | 5.012819979 | 3.04E-143 |
| HS6ST1 | downregulated mRNA | 2.151686629 | 3.28E-139 |
| SLC12A1 | downregulated mRNA | 8.26986012 | 2.16E-134 |
| GSTM3 | downregulated mRNA | 3.30320939 | 1.05E-133 |
| HYKK | downregulated mRNA | 2.002653986 | 3.69E-132 |
| FGF1 | downregulated mRNA | 4.221320338 | 5.66E-129 |
| SLC4A11 | downregulated mRNA | 4.413686952 | 4.03E-126 |
| ERMP1 | downregulated mRNA | 2.44982959 | 5.71E-123 |
| SLC4A8 | downregulated mRNA | 2.696350041 | 1.87E-122 |
| C14orf37 | downregulated mRNA | 3.550006442 | 8.08E-121 |
| CLUL1 | downregulated mRNA | 3.442350538 | 1.47E-119 |
| HS6ST2 | downregulated mRNA | 6.260252419 | 6.19E-119 |
| CLDN16 | downregulated mRNA | 5.857931697 | 7.22E-119 |
| TRPV6 | downregulated mRNA | 4.82164685 | 1.91E-118 |
| ACSF2 | downregulated mRNA | 3.278295581 | 5.47E-118 |
| AKAP3 | downregulated mRNA | 2.331434685 | 1.62E-117 |
| DUSP9 | downregulated mRNA | 6.801622028 | 1.30E-115 |
| PLA2R1 | downregulated mRNA | 3.101185427 | 1.45E-115 |
| WNK4 | downregulated mRNA | 4.074783174 | 1.80E-114 |
| WNT8B | downregulated mRNA | 3.919908105 | 1.69E-110 |
| ALDH6A1 | downregulated mRNA | 3.047475684 | 3.16E-110 |
| USP44 | downregulated mRNA | 3.00918266 | 1.89E-108 |
| RBP2 | downregulated mRNA | 3.963410696 | 2.84E-107 |
| EGF | downregulated mRNA | 5.410876349 | 3.71E-107 |
| FAM171A1 | downregulated mRNA | 2.169671269 | 3.40E-106 |
| UNCX | downregulated mRNA | 7.311177285 | 4.66E-106 |
| KLHL14 | downregulated mRNA | 3.821206749 | 8.72E-106 |
| ATP12A | downregulated mRNA | 7.227037719 | 1.28E-104 |
| COBLL1 | downregulated mRNA | 2.376246069 | 1.02E-103 |
| RANBP3L | downregulated mRNA | 5.190029963 | 7.23E-102 |
| NHLRC4 | downregulated mRNA | 3.192206678 | 7.44E-101 |
| SFXN2 | downregulated mRNA | 2.368369883 | 9.14E-101 |
| LGI2 | downregulated mRNA | 3.269219488 | 9.68E-101 |
| SFRP1 | downregulated mRNA | 5.277016513 | 1.12E-100 |
| CPNE6 | downregulated mRNA | 6.555977186 | 4.28E-100 |
| SLC14A2 | downregulated mRNA | 6.358108124 | 8.33E-99 |
| CYFIP2 | downregulated mRNA | 2.258518925 | 1.03E-98 |
| TCF21 | downregulated mRNA | 3.522675186 | 4.44E-98 |
| CNTD2 | downregulated mRNA | 4.769275359 | 5.08E-98 |
| CLIC5 | downregulated mRNA | 3.816860902 | 5.37E-98 |
| PRKAR2B | downregulated mRNA | 2.227056338 | 6.74E-98 |
| NOS1 | downregulated mRNA | 4.408820748 | 1.35E-97 |
| TMEM45B | downregulated mRNA | 3.896886794 | 4.91E-97 |
| NT5C1A | downregulated mRNA | 4.215378645 | 6.28E-97 |
| CACNA2D2 | downregulated mRNA | 2.281533417 | 1.22E-96 |
| SRGAP3 | downregulated mRNA | 2.440273021 | 2.32E-95 |
| SIAH3 | downregulated mRNA | 5.1817995 | 1.55E-94 |
| WNT9B | downregulated mRNA | 4.094429578 | 1.06E-93 |
| ABAT | downregulated mRNA | 3.051236832 | 1.44E-93 |
| PROX1 | downregulated mRNA | 4.312873774 | 1.49E-91 |
| CLDN19 | downregulated mRNA | 5.687934177 | 2.29E-91 |
| KCNJ1 | downregulated mRNA | 6.264079103 | 3.81E-91 |
| KCNK13 | downregulated mRNA | 2.628880497 | 5.19E-91 |
| SOST | downregulated mRNA | 6.835683605 | 6.80E-91 |
| HRG | downregulated mRNA | 7.044120213 | 8.91E-91 |
| UPP2 | downregulated mRNA | 4.313172326 | 1.50E-90 |
| EFHD1 | downregulated mRNA | 2.618205175 | 2.34E-90 |
| RNF212B | downregulated mRNA | 4.111996176 | 4.89E-90 |
| FOXC1 | downregulated mRNA | 2.170202055 | 6.51E-90 |
| SH3GL3 | downregulated mRNA | 5.621814542 | 1.55E-89 |
| SEMA6D | downregulated mRNA | 2.932703568 | 1.17E-88 |
| PLCL1 | downregulated mRNA | 2.648687217 | 7.06E-88 |
| CPAMD8 | downregulated mRNA | 2.77391907 | 7.48E-88 |
| PTPRO | downregulated mRNA | 3.194105929 | 8.76E-88 |
| CCNI2 | downregulated mRNA | 2.917789428 | 1.83E-87 |
| ERVMER34-1 | downregulated mRNA | 4.669831555 | 6.45E-87 |
| SLC16A5 | downregulated mRNA | 3.003196281 | 8.61E-87 |
| ACADSB | downregulated mRNA | 2.014615576 | 4.15E-86 |
| NPHS1 | downregulated mRNA | 5.947852561 | 5.30E-86 |
| PRR15 | downregulated mRNA | 3.911707783 | 1.15E-85 |
| CRHBP | downregulated mRNA | 4.075198535 | 1.49E-85 |
| ABHD17C | downregulated mRNA | 2.029344687 | 2.88E-85 |
| FAM81A | downregulated mRNA | 2.542334208 | 1.02E-84 |
| EMX1 | downregulated mRNA | 2.608219131 | 2.78E-84 |
| PRDM16 | downregulated mRNA | 3.674836062 | 6.05E-84 |
| SLC7A8 | downregulated mRNA | 3.584618415 | 8.48E-84 |
| ABCA4 | downregulated mRNA | 4.615947615 | 2.13E-83 |
| AIF1L | downregulated mRNA | 3.2104658 | 5.10E-83 |
| AGBL4 | downregulated mRNA | 2.460889266 | 5.10E-83 |
| CHP2 | downregulated mRNA | 5.289635007 | 5.10E-83 |
| BTG2 | downregulated mRNA | 2.102665715 | 5.41E-83 |
| EYA4 | downregulated mRNA | 3.484369868 | 1.37E-82 |
| KNG1 | downregulated mRNA | 7.523966569 | 2.26E-82 |
| MECOM | downregulated mRNA | 2.536957598 | 2.77E-82 |
| PCK2 | downregulated mRNA | 2.297018246 | 2.87E-82 |
| TPPP2 | downregulated mRNA | 4.114271436 | 3.69E-82 |
| XPNPEP2 | downregulated mRNA | 5.398114113 | 6.90E-82 |
| HCRTR2 | downregulated mRNA | 5.854443628 | 1.50E-81 |
| TMEM178A | downregulated mRNA | 3.195527161 | 1.85E-81 |
| CASR | downregulated mRNA | 5.516685556 | 1.21E-80 |
| ASS1 | downregulated mRNA | 2.718956325 | 2.18E-80 |
| SLC28A2 | downregulated mRNA | 3.512220522 | 3.36E-79 |
| GP2 | downregulated mRNA | 6.852771734 | 5.30E-79 |
| MRO | downregulated mRNA | 2.985730554 | 1.61E-78 |
| KCNE1B | downregulated mRNA | 5.15759945 | 3.27E-78 |
| TMEM52B | downregulated mRNA | 5.020680851 | 4.71E-78 |
| SLC15A2 | downregulated mRNA | 3.055142113 | 5.20E-78 |
| PPP2R2B | downregulated mRNA | 2.280333058 | 6.51E-78 |
| RNF150 | downregulated mRNA | 3.091123307 | 9.54E-78 |
| IRX1 | downregulated mRNA | 4.567055591 | 9.54E-78 |
| ACY1 | downregulated mRNA | 2.180368199 | 1.21E-76 |
| FLRT1 | downregulated mRNA | 4.091448611 | 2.37E-76 |
| CCDC181 | downregulated mRNA | 3.042859837 | 2.53E-76 |
| SLC5A7 | downregulated mRNA | 4.337606331 | 7.24E-76 |
| CASZ1 | downregulated mRNA | 2.01669899 | 1.59E-75 |
| MYOZ2 | downregulated mRNA | 3.116803306 | 3.42E-75 |
| CYP27B1 | downregulated mRNA | 3.589384342 | 1.09E-74 |
| KLHL13 | downregulated mRNA | 2.164049549 | 1.25E-74 |
| ALB | downregulated mRNA | 5.063237051 | 1.28E-74 |
| RASL11B | downregulated mRNA | 4.258579517 | 1.68E-74 |
| SLC5A2 | downregulated mRNA | 4.717663386 | 1.82E-74 |
| ERICH4 | downregulated mRNA | 3.60802419 | 3.14E-74 |
| TYRO3 | downregulated mRNA | 2.23910561 | 1.51E-73 |
| FAM167A | downregulated mRNA | 3.500115192 | 1.56E-73 |
| KCNK10 | downregulated mRNA | 3.339284371 | 1.62E-73 |
| TRPV5 | downregulated mRNA | 5.123116464 | 5.36E-73 |
| EHF | downregulated mRNA | 5.209985124 | 1.14E-72 |
| TCF24 | downregulated mRNA | 5.055608269 | 1.73E-72 |
| AQP2 | downregulated mRNA | 8.902341454 | 5.55E-72 |
| C1orf226 | downregulated mRNA | 2.759813024 | 1.26E-71 |
| COL4A6 | downregulated mRNA | 4.365146919 | 2.75E-71 |
| TNNT2 | downregulated mRNA | 4.840146033 | 3.27E-71 |
| SORD | downregulated mRNA | 2.182975971 | 3.62E-71 |
| IYD | downregulated mRNA | 4.276635748 | 6.79E-71 |
| LMO3 | downregulated mRNA | 3.333767267 | 9.15E-71 |
| AVPR2 | downregulated mRNA | 3.405075572 | 4.36E-70 |
| SPTB | downregulated mRNA | 2.643433247 | 4.57E-70 |
| TRPM6 | downregulated mRNA | 2.98301443 | 5.25E-70 |
| PTH1R | downregulated mRNA | 3.243995058 | 5.56E-70 |
| NOS1AP | downregulated mRNA | 2.770831145 | 6.56E-70 |
| EDDM3A | downregulated mRNA | 4.919573223 | 7.81E-70 |
| EPCAM | downregulated mRNA | 2.842395419 | 2.14E-69 |
| UMOD | downregulated mRNA | 8.470634076 | 6.21E-69 |
| NIPAL1 | downregulated mRNA | 3.195223598 | 1.60E-68 |
| RP11-307N16.6 | downregulated mRNA | 3.021992281 | 1.24E-67 |
| MAP6 | downregulated mRNA | 2.386954242 | 1.97E-67 |
| SERPINA5 | downregulated mRNA | 4.873402002 | 3.09E-67 |
| REEP6 | downregulated mRNA | 2.957030306 | 3.26E-67 |
| RP5-1187M17.10 | downregulated mRNA | 2.293292786 | 3.46E-67 |
| SLC47A2 | downregulated mRNA | 3.917865856 | 3.71E-67 |
| FAM19A4 | downregulated mRNA | 4.443847359 | 6.07E-67 |
| PAPPA | downregulated mRNA | 3.122978162 | 6.85E-67 |
| CALML3 | downregulated mRNA | 5.651326633 | 1.14E-66 |
| EPGN | downregulated mRNA | 3.987273304 | 4.64E-66 |
| SLC22A8 | downregulated mRNA | 6.129832909 | 5.43E-66 |
| MPP7 | downregulated mRNA | 2.770680828 | 5.85E-66 |
| DTX1 | downregulated mRNA | 2.438173096 | 6.88E-66 |
| GADL1 | downregulated mRNA | 4.401498199 | 9.16E-66 |
| HPD | downregulated mRNA | 4.864576916 | 1.38E-65 |
| PEG3 | downregulated mRNA | 2.349650307 | 1.48E-65 |
| AGXT | downregulated mRNA | 3.613900182 | 2.03E-65 |
| SLC14A1 | downregulated mRNA | 2.983147079 | 4.17E-65 |
| HOMER1 | downregulated mRNA | 2.190943329 | 6.99E-65 |
| MUC15 | downregulated mRNA | 7.226322165 | 9.44E-65 |
| DIO1 | downregulated mRNA | 4.788764466 | 1.61E-64 |
| FAM222A | downregulated mRNA | 2.731366267 | 2.26E-64 |
| DEGS2 | downregulated mRNA | 2.966666433 | 2.66E-64 |
| ST6GAL1 | downregulated mRNA | 2.163078415 | 3.56E-64 |
| PCDH9 | downregulated mRNA | 3.242437008 | 4.48E-64 |
| RP11-766F14.2 | downregulated mRNA | 6.747881872 | 9.19E-64 |
| CACNA2D3 | downregulated mRNA | 2.086537178 | 9.75E-64 |
| AMPH | downregulated mRNA | 3.148557585 | 1.14E-63 |
| RNF43 | downregulated mRNA | 2.537314209 | 4.19E-63 |
| CLDN14 | downregulated mRNA | 2.940946336 | 5.80E-63 |
| NYAP1 | downregulated mRNA | 2.595430667 | 1.21E-62 |
| VSIG8 | downregulated mRNA | 2.844927989 | 2.14E-62 |
| TNNI1 | downregulated mRNA | 4.035729738 | 3.59E-62 |
| GPC3 | downregulated mRNA | 3.632612911 | 1.37E-61 |
| FMO5 | downregulated mRNA | 2.439348596 | 2.00E-61 |
| STRA6 | downregulated mRNA | 4.024391741 | 2.66E-61 |
| MARVELD2 | downregulated mRNA | 2.369941236 | 3.25E-61 |
| PDE1A | downregulated mRNA | 2.796969629 | 4.70E-61 |
| CEL | downregulated mRNA | 3.712140074 | 5.49E-61 |
| C2orf71 | downregulated mRNA | 5.800238343 | 1.63E-60 |
| NKD1 | downregulated mRNA | 2.900434499 | 1.80E-60 |
| HECW1 | downregulated mRNA | 3.71163578 | 1.83E-60 |
| SUSD4 | downregulated mRNA | 3.635886274 | 2.09E-60 |
| KLRG2 | downregulated mRNA | 5.050792243 | 2.49E-60 |
| TFAP2B | downregulated mRNA | 5.741680379 | 6.37E-60 |
| DPEP1 | downregulated mRNA | 4.372164197 | 7.00E-60 |
| SLC26A4 | downregulated mRNA | 3.247355681 | 9.15E-60 |
| NRK | downregulated mRNA | 4.796666267 | 1.97E-59 |
| CST9 | downregulated mRNA | 5.484153406 | 3.27E-59 |
| SHBG | downregulated mRNA | 2.310106561 | 3.36E-59 |
| NAT8L | downregulated mRNA | 4.563215292 | 3.44E-59 |
| CDH16 | downregulated mRNA | 2.355164217 | 4.08E-59 |
| COL26A1 | downregulated mRNA | 4.631457863 | 4.34E-59 |
| RIMBP2 | downregulated mRNA | 3.441529327 | 5.64E-59 |
| ZNF488 | downregulated mRNA | 3.520223742 | 1.39E-58 |
| NUAK2 | downregulated mRNA | 2.107475802 | 2.49E-58 |
| SEMG2 | downregulated mRNA | 7.580252346 | 2.76E-58 |
| CLDN10 | downregulated mRNA | 2.295953352 | 2.84E-58 |
| S100A2 | downregulated mRNA | 3.435346096 | 1.13E-57 |
| C10orf55 | downregulated mRNA | 2.266731927 | 4.88E-57 |
| RAG2 | downregulated mRNA | 3.854874945 | 1.49E-56 |
| DACH1 | downregulated mRNA | 2.367676761 | 6.12E-56 |
| CA8 | downregulated mRNA | 3.243016681 | 1.10E-55 |
| FAM151A | downregulated mRNA | 3.962337959 | 1.52E-55 |
| SPTBN2 | downregulated mRNA | 4.294098889 | 2.34E-55 |
| KCTD8 | downregulated mRNA | 4.062890433 | 2.80E-55 |
| PTGER1 | downregulated mRNA | 4.820555026 | 3.90E-55 |
| HELT | downregulated mRNA | 5.522605678 | 4.08E-55 |
| PRR35 | downregulated mRNA | 6.916343974 | 5.44E-55 |
| COL4A4 | downregulated mRNA | 2.112801359 | 6.46E-55 |
| SLC52A3 | downregulated mRNA | 2.949388926 | 6.72E-55 |
| CCSER1 | downregulated mRNA | 2.465957419 | 1.15E-54 |
| PDILT | downregulated mRNA | 3.068834931 | 2.22E-54 |
| KCNJ12 | downregulated mRNA | 2.321785199 | 7.92E-54 |
| AFM | downregulated mRNA | 4.677940985 | 8.57E-54 |
| BMP6 | downregulated mRNA | 2.117211791 | 2.36E-53 |
| ASB15 | downregulated mRNA | 5.205501877 | 2.77E-53 |
| ENTPD3 | downregulated mRNA | 3.217729211 | 3.37E-53 |
| RP11-190A12.7 | downregulated mRNA | 3.948108678 | 5.88E-53 |
| MRLN | downregulated mRNA | 4.478685955 | 7.44E-53 |
| MT1G | downregulated mRNA | 4.437701209 | 1.49E-52 |
| GGACT | downregulated mRNA | 2.376804646 | 1.88E-52 |
| ADGRV1 | downregulated mRNA | 2.711843579 | 2.10E-52 |
| GATA3 | downregulated mRNA | 3.060476275 | 2.22E-52 |
| AQP3 | downregulated mRNA | 2.198273878 | 5.11E-52 |
| F11 | downregulated mRNA | 4.230181092 | 7.00E-52 |
| PRRG2 | downregulated mRNA | 2.786381909 | 8.36E-52 |
| ESRRG | downregulated mRNA | 3.059813245 | 1.15E-51 |
| PROZ | downregulated mRNA | 4.1566622 | 2.51E-51 |
| TNNC1 | downregulated mRNA | 3.321103667 | 2.64E-51 |
| RASD1 | downregulated mRNA | 2.527769365 | 3.60E-51 |
| SSC4D | downregulated mRNA | 2.220179894 | 7.88E-51 |
| HOXB6 | downregulated mRNA | 2.066811626 | 9.99E-51 |
| RIPPLY1 | downregulated mRNA | 2.834308038 | 1.09E-50 |
| KSR2 | downregulated mRNA | 3.211318297 | 1.19E-50 |
| FBP1 | downregulated mRNA | 2.342580805 | 1.37E-50 |
| HPCAL4 | downregulated mRNA | 3.973565037 | 2.40E-50 |
| ITLN1 | downregulated mRNA | 3.642762048 | 2.81E-50 |
| MYBPH | downregulated mRNA | 3.195215735 | 1.13E-49 |
| DCXR | downregulated mRNA | 2.05946633 | 1.59E-49 |
| CCDC160 | downregulated mRNA | 2.378556513 | 1.78E-49 |
| PADI2 | downregulated mRNA | 2.468801575 | 1.81E-49 |
| C5orf67 | downregulated mRNA | 2.786164785 | 2.04E-49 |
| GRIK2 | downregulated mRNA | 2.793097774 | 2.06E-49 |
| SLC13A3 | downregulated mRNA | 4.686813691 | 2.63E-49 |
| SLC2A12 | downregulated mRNA | 3.299427468 | 4.55E-49 |
| CTSV | downregulated mRNA | 2.849061757 | 8.54E-49 |
| IGSF10 | downregulated mRNA | 2.729281368 | 1.32E-48 |
| TMEM207 | downregulated mRNA | 8.199426812 | 1.37E-48 |
| TMEM72 | downregulated mRNA | 3.0106605 | 1.42E-48 |
| LRRC2 | downregulated mRNA | 3.411750836 | 2.11E-48 |
| ADH1C | downregulated mRNA | 4.33468467 | 2.28E-48 |
| MRAP2 | downregulated mRNA | 3.337871187 | 2.95E-48 |
| ALDOB | downregulated mRNA | 4.721738743 | 3.27E-48 |
| CGN | downregulated mRNA | 2.424939077 | 3.55E-48 |
| NUDT10 | downregulated mRNA | 2.665778142 | 3.64E-48 |
| NR1I3 | downregulated mRNA | 2.446610601 | 4.35E-48 |
| PLAU | downregulated mRNA | 2.159095311 | 5.62E-48 |
| SLC9A3 | downregulated mRNA | 3.483428976 | 6.80E-48 |
| PIPOX | downregulated mRNA | 2.972940587 | 7.55E-48 |
| GALNT3 | downregulated mRNA | 2.541987511 | 9.39E-48 |
| CLCNKA | downregulated mRNA | 4.91513377 | 1.31E-47 |
| FAM217A | downregulated mRNA | 2.351575345 | 2.13E-47 |
| RAP1GAP | downregulated mRNA | 2.553240039 | 3.43E-47 |
| FAM3B | downregulated mRNA | 3.856621187 | 4.97E-47 |
| TYRP1 | downregulated mRNA | 5.238532383 | 5.78E-47 |
| SLC9A4 | downregulated mRNA | 6.102155439 | 6.50E-47 |
| SPTSSB | downregulated mRNA | 3.454553 | 6.74E-47 |
| OLFM3 | downregulated mRNA | 5.425972265 | 8.48E-47 |
| NCCRP1 | downregulated mRNA | 2.937513911 | 1.08E-46 |
| RASSF10 | downregulated mRNA | 3.088269142 | 2.04E-46 |
| ACKR2 | downregulated mRNA | 2.254740417 | 2.37E-46 |
| TTC36 | downregulated mRNA | 2.755002384 | 4.08E-46 |
| COL4A5 | downregulated mRNA | 2.29917019 | 4.54E-46 |
| ACOT12 | downregulated mRNA | 4.279310698 | 5.69E-46 |
| CGNL1 | downregulated mRNA | 2.106484159 | 6.46E-46 |
| SLC5A11 | downregulated mRNA | 3.069999544 | 9.17E-46 |
| OVCH2 | downregulated mRNA | 4.402106536 | 9.41E-46 |
| CKM | downregulated mRNA | 2.87044078 | 9.96E-46 |
| MYO3B | downregulated mRNA | 3.73747388 | 1.06E-45 |
| ARL4D | downregulated mRNA | 2.673916002 | 1.24E-45 |
| C8orf4 | downregulated mRNA | 2.162168062 | 1.87E-45 |
| GPR182 | downregulated mRNA | 2.421799794 | 1.88E-45 |
| NPHS2 | downregulated mRNA | 7.100151799 | 2.80E-45 |
| GGT6 | downregulated mRNA | 4.696073886 | 4.90E-45 |
| FAM46B | downregulated mRNA | 2.565849102 | 8.25E-45 |
| NDST3 | downregulated mRNA | 3.048250794 | 1.03E-44 |
| KLK7 | downregulated mRNA | 5.806927817 | 1.07E-44 |
| NTNG1 | downregulated mRNA | 4.457754236 | 1.80E-44 |
| MAL2 | downregulated mRNA | 2.712286545 | 2.26E-44 |
| CHL1 | downregulated mRNA | 3.314831225 | 3.51E-44 |
| GAL3ST3 | downregulated mRNA | 4.344311361 | 5.57E-44 |
| NELL1 | downregulated mRNA | 5.777762792 | 9.55E-44 |
| LIPH | downregulated mRNA | 3.560071759 | 1.13E-43 |
| HSPB7 | downregulated mRNA | 3.231657811 | 1.24E-43 |
| NPY5R | downregulated mRNA | 2.412019065 | 1.41E-43 |
| SLC34A3 | downregulated mRNA | 3.395535422 | 1.47E-43 |
| HOXB9 | downregulated mRNA | 3.257932451 | 1.87E-43 |
| ANKRD34B | downregulated mRNA | 3.908352757 | 3.15E-43 |
| OTOGL | downregulated mRNA | 2.495171398 | 9.67E-43 |
| MAGEE2 | downregulated mRNA | 2.720905602 | 1.13E-42 |
| RALYL | downregulated mRNA | 5.726977923 | 1.16E-42 |
| SLC6A17 | downregulated mRNA | 2.738735253 | 1.39E-42 |
| AP1M2 | downregulated mRNA | 2.44419503 | 3.05E-42 |
| TMC1 | downregulated mRNA | 2.105161779 | 3.21E-42 |
| GPM6B | downregulated mRNA | 2.080178746 | 3.39E-42 |
| CLDN11 | downregulated mRNA | 2.450984802 | 5.19E-42 |
| SCN7A | downregulated mRNA | 3.974529501 | 5.96E-42 |
| RPS6KA6 | downregulated mRNA | 2.962594456 | 7.09E-42 |
| CYP4F2 | downregulated mRNA | 4.909074597 | 7.61E-42 |
| P2RX2 | downregulated mRNA | 3.563587278 | 1.14E-41 |
| RP11-565P22.6 | downregulated mRNA | 2.859371277 | 1.26E-41 |
| ADAMTS16 | downregulated mRNA | 2.660235893 | 1.27E-41 |
| CCDC64 | downregulated mRNA | 2.011517894 | 1.53E-41 |
| GATM | downregulated mRNA | 2.190242757 | 2.04E-41 |
| TUBB2B | downregulated mRNA | 2.587300058 | 2.19E-41 |
| MPPED2 | downregulated mRNA | 2.43038743 | 4.07E-41 |
| TACSTD2 | downregulated mRNA | 3.462993314 | 5.42E-41 |
| GJA3 | downregulated mRNA | 2.985251303 | 5.44E-41 |
| RGS6 | downregulated mRNA | 2.290251547 | 5.55E-41 |
| TSPAN8 | downregulated mRNA | 3.901329088 | 7.16E-41 |
| SULT2B1 | downregulated mRNA | 3.12801562 | 8.36E-41 |
| GMPR | downregulated mRNA | 2.277852956 | 9.07E-41 |
| SYT7 | downregulated mRNA | 3.327477418 | 1.37E-40 |
| DACH2 | downregulated mRNA | 3.18047235 | 1.85E-40 |
| PM20D1 | downregulated mRNA | 2.847621897 | 2.07E-40 |
| NPNT | downregulated mRNA | 2.005053621 | 2.26E-40 |
| C7 | downregulated mRNA | 3.296522911 | 4.04E-40 |
| OLFM4 | downregulated mRNA | 4.845243737 | 4.16E-40 |
| AGMAT | downregulated mRNA | 2.459989636 | 5.55E-40 |
| FREM1 | downregulated mRNA | 3.302998912 | 5.99E-40 |
| CYS1 | downregulated mRNA | 2.084473229 | 6.97E-40 |
| SLC34A1 | downregulated mRNA | 4.4190392 | 7.18E-40 |
| ERBB4 | downregulated mRNA | 3.838049618 | 1.21E-39 |
| MAN1C1 | downregulated mRNA | 2.183992616 | 1.52E-39 |
| CNGA1 | downregulated mRNA | 2.478151597 | 1.87E-39 |
| CLSTN2 | downregulated mRNA | 2.702002741 | 2.61E-39 |
| SLC6A4 | downregulated mRNA | 2.121908751 | 3.04E-39 |
| GLDC | downregulated mRNA | 2.345202536 | 4.21E-39 |
| DNASE1L3 | downregulated mRNA | 2.169996144 | 4.89E-39 |
| VWA2 | downregulated mRNA | 2.890363613 | 5.30E-39 |
| FABP1 | downregulated mRNA | 4.406285685 | 7.12E-39 |
| MOGAT2 | downregulated mRNA | 4.241775711 | 7.34E-39 |
| CHGB | downregulated mRNA | 3.607785687 | 9.85E-39 |
| MYH7 | downregulated mRNA | 3.104032033 | 1.73E-38 |
| BPI | downregulated mRNA | 2.408546078 | 3.61E-38 |
| FAM169A | downregulated mRNA | 3.062894312 | 4.38E-38 |
| SCNN1B | downregulated mRNA | 3.952836736 | 8.83E-38 |
| FMN2 | downregulated mRNA | 3.738440216 | 1.17E-37 |
| C9orf135 | downregulated mRNA | 3.477852828 | 1.49E-37 |
| PLEKHB1 | downregulated mRNA | 2.297006011 | 1.65E-37 |
| KLK6 | downregulated mRNA | 5.414077914 | 2.08E-37 |
| RXFP4 | downregulated mRNA | 2.404919496 | 2.13E-37 |
| SLC13A2 | downregulated mRNA | 4.70123627 | 2.24E-37 |
| ADH6 | downregulated mRNA | 2.621078165 | 2.25E-37 |
| COL9A2 | downregulated mRNA | 2.177965918 | 2.26E-37 |
| GRM1 | downregulated mRNA | 3.344151123 | 2.43E-37 |
| FXYD4 | downregulated mRNA | 6.123581478 | 2.56E-37 |
| ITGB6 | downregulated mRNA | 2.573099887 | 3.10E-37 |
| ABCA13 | downregulated mRNA | 3.455399788 | 3.53E-37 |
| GDF3 | downregulated mRNA | 2.137586148 | 4.15E-37 |
| ASPDH | downregulated mRNA | 2.73023899 | 6.04E-37 |
| DAO | downregulated mRNA | 3.237147397 | 7.43E-37 |
| CNTN1 | downregulated mRNA | 3.663912281 | 1.86E-36 |
| GPAT3 | downregulated mRNA | 2.461808807 | 3.01E-36 |
| SLC36A2 | downregulated mRNA | 4.676063582 | 3.32E-36 |
| INSRR | downregulated mRNA | 3.590882679 | 3.87E-36 |
| CYP2B6 | downregulated mRNA | 4.487484988 | 3.95E-36 |
| VGLL1 | downregulated mRNA | 6.152071752 | 1.17E-35 |
| MT1H | downregulated mRNA | 4.341384611 | 1.31E-35 |
| MAL | downregulated mRNA | 3.25845931 | 1.43E-35 |
| SEMG1 | downregulated mRNA | 5.014532004 | 1.52E-35 |
| PTPRQ | downregulated mRNA | 3.953488841 | 6.18E-35 |
| ALDH4A1 | downregulated mRNA | 2.063023392 | 6.25E-35 |
| SOWAHA | downregulated mRNA | 3.063550677 | 9.61E-35 |
| HSD11B2 | downregulated mRNA | 2.759314573 | 1.03E-34 |
| PPP1R1B | downregulated mRNA | 3.735841664 | 1.08E-34 |
| MFSD6L | downregulated mRNA | 3.371834762 | 1.11E-34 |
| C12orf74 | downregulated mRNA | 2.090299923 | 1.15E-34 |
| SHISA2 | downregulated mRNA | 2.620907587 | 1.18E-34 |
| GAS1 | downregulated mRNA | 2.09244748 | 1.35E-34 |
| PNPLA1 | downregulated mRNA | 2.468708219 | 1.36E-34 |
| SMIM5 | downregulated mRNA | 3.107295927 | 1.80E-34 |
| EPN3 | downregulated mRNA | 3.723039874 | 2.13E-34 |
| NKX6-2 | downregulated mRNA | 3.025651099 | 2.19E-34 |
| SLC12A3 | downregulated mRNA | 4.744402369 | 2.83E-34 |
| LDHD | downregulated mRNA | 2.25123165 | 3.01E-34 |
| MAP3K15 | downregulated mRNA | 2.836999375 | 4.36E-34 |
| B4GALNT2 | downregulated mRNA | 3.59516188 | 5.12E-34 |
| CAMK2A | downregulated mRNA | 2.737238095 | 5.77E-34 |
| DLX3 | downregulated mRNA | 2.227538737 | 8.93E-34 |
| CR2 | downregulated mRNA | 3.874694514 | 9.46E-34 |
| C10orf82 | downregulated mRNA | 2.776210915 | 9.47E-34 |
| AXDND1 | downregulated mRNA | 2.082842092 | 1.07E-33 |
| FAM181B | downregulated mRNA | 2.215703085 | 1.23E-33 |
| SCN2A | downregulated mRNA | 3.007842563 | 1.24E-33 |
| GLOD5 | downregulated mRNA | 2.393116735 | 1.24E-33 |
| SLC25A47 | downregulated mRNA | 2.406504403 | 1.28E-33 |
| SUCNR1 | downregulated mRNA | 2.147045323 | 1.30E-33 |
| ALDH3B2 | downregulated mRNA | 3.849192257 | 1.60E-33 |
| CRB2 | downregulated mRNA | 2.889179273 | 2.01E-33 |
| KDF1 | downregulated mRNA | 2.021745972 | 2.37E-33 |
| SYN2 | downregulated mRNA | 2.302389254 | 2.62E-33 |
| EPB41L4B | downregulated mRNA | 3.334852948 | 2.88E-33 |
| PLPPR1 | downregulated mRNA | 4.194228205 | 2.97E-33 |
| SCNN1G | downregulated mRNA | 4.718595152 | 3.73E-33 |
| FRG2C | downregulated mRNA | 4.089749836 | 3.88E-33 |
| OVOL1 | downregulated mRNA | 2.320809053 | 4.71E-33 |
| PROM2 | downregulated mRNA | 3.487579111 | 5.97E-33 |
| DDX25 | downregulated mRNA | 2.459740374 | 7.06E-33 |
| IGSF11 | downregulated mRNA | 2.890177806 | 7.32E-33 |
| LRIT3 | downregulated mRNA | 2.070016892 | 8.72E-33 |
| ACSL6 | downregulated mRNA | 2.144366393 | 9.14E-33 |
| VWA3B | downregulated mRNA | 2.237454654 | 1.10E-32 |
| SSTR5 | downregulated mRNA | 3.752126013 | 1.32E-32 |
| PAK6 | downregulated mRNA | 3.329906503 | 1.36E-32 |
| ANK2 | downregulated mRNA | 2.136683121 | 2.04E-32 |
| TJP3 | downregulated mRNA | 2.736088156 | 2.05E-32 |
| DLGAP2 | downregulated mRNA | 2.631697324 | 2.49E-32 |
| CPA1 | downregulated mRNA | 2.887829175 | 2.58E-32 |
| C11orf16 | downregulated mRNA | 2.013972828 | 4.50E-32 |
| TMEM30B | downregulated mRNA | 2.805464137 | 4.52E-32 |
| SLC30A2 | downregulated mRNA | 3.374630191 | 6.06E-32 |
| FER1L6 | downregulated mRNA | 3.346220082 | 6.67E-32 |
| AKAP4 | downregulated mRNA | 2.326188821 | 7.20E-32 |
| GRIK5 | downregulated mRNA | 2.989812682 | 8.66E-32 |
| OCLN | downregulated mRNA | 2.058633589 | 8.76E-32 |
| FP325317.1 | downregulated mRNA | 3.201229776 | 9.80E-32 |
| HOXB8 | downregulated mRNA | 2.213075331 | 1.07E-31 |
| PPP1R36 | downregulated mRNA | 2.396625525 | 2.60E-31 |
| DUSP26 | downregulated mRNA | 2.667661718 | 2.77E-31 |
| LAD1 | downregulated mRNA | 2.658153962 | 3.04E-31 |
| TMPRSS4 | downregulated mRNA | 3.698776966 | 3.07E-31 |
| HRASLS2 | downregulated mRNA | 2.052621583 | 3.11E-31 |
| NAP1L2 | downregulated mRNA | 2.228623659 | 3.97E-31 |
| FBXO2 | downregulated mRNA | 2.457691074 | 4.13E-31 |
| FSTL4 | downregulated mRNA | 2.5366496 | 4.45E-31 |
| ACOT6 | downregulated mRNA | 2.199367515 | 4.99E-31 |
| G6PC | downregulated mRNA | 3.513267604 | 8.25E-31 |
| PHYHD1 | downregulated mRNA | 2.218369433 | 8.84E-31 |
| WNT7B | downregulated mRNA | 3.803550334 | 1.05E-30 |
| C2orf54 | downregulated mRNA | 3.815822106 | 1.24E-30 |
| MAPK4 | downregulated mRNA | 4.058041524 | 1.57E-30 |
| IL19 | downregulated mRNA | 2.933982019 | 1.82E-30 |
| CAPSL | downregulated mRNA | 2.833571141 | 2.08E-30 |
| ARC | downregulated mRNA | 2.092746977 | 2.10E-30 |
| MRGPRF | downregulated mRNA | 2.19624177 | 2.18E-30 |
| ANKRD2 | downregulated mRNA | 3.202037681 | 2.31E-30 |
| DPP6 | downregulated mRNA | 3.357327321 | 2.78E-30 |
| CBLN2 | downregulated mRNA | 3.076346206 | 4.35E-30 |
| SPATA16 | downregulated mRNA | 3.518444119 | 4.88E-30 |
| MUC6 | downregulated mRNA | 2.628901338 | 5.01E-30 |
| NMUR2 | downregulated mRNA | 3.4084378 | 5.07E-30 |
| LMX1B | downregulated mRNA | 4.075759057 | 8.63E-30 |
| TMEM8C | downregulated mRNA | 3.225414786 | 1.17E-29 |
| GPR12 | downregulated mRNA | 3.552206842 | 1.23E-29 |
| MT1F | downregulated mRNA | 2.144474017 | 1.30E-29 |
| TTPA | downregulated mRNA | 3.307015186 | 1.36E-29 |
| KIAA2022 | downregulated mRNA | 2.927952379 | 1.39E-29 |
| CNKSR1 | downregulated mRNA | 2.541511203 | 1.41E-29 |
| CA10 | downregulated mRNA | 4.828120152 | 2.00E-29 |
| PRSS35 | downregulated mRNA | 2.17285738 | 2.17E-29 |
| PTCHD3 | downregulated mRNA | 2.732863389 | 5.29E-29 |
| FOXJ1 | downregulated mRNA | 3.161924613 | 6.27E-29 |
| LYPD6 | downregulated mRNA | 2.636840938 | 7.35E-29 |
| DOK7 | downregulated mRNA | 2.347759409 | 8.72E-29 |
| IL11 | downregulated mRNA | 3.313825895 | 1.18E-28 |
| MCCD1 | downregulated mRNA | 4.247147332 | 1.41E-28 |
| ALX1 | downregulated mRNA | 3.071766083 | 1.69E-28 |
| GMNC | downregulated mRNA | 3.556720661 | 5.61E-28 |
| PAPPA2 | downregulated mRNA | 3.390964034 | 6.84E-28 |
| PKHD1L1 | downregulated mRNA | 2.44704811 | 1.16E-27 |
| TACR3 | downregulated mRNA | 3.952022021 | 1.54E-27 |
| CHST6 | downregulated mRNA | 2.653502138 | 1.67E-27 |
| PLXNA4 | downregulated mRNA | 2.416199429 | 1.94E-27 |
| SCNN1A | downregulated mRNA | 2.851888365 | 1.97E-27 |
| PCK1 | downregulated mRNA | 2.848402007 | 1.99E-27 |
| TCEAL6 | downregulated mRNA | 3.185899995 | 2.27E-27 |
| FAM46D | downregulated mRNA | 3.11611009 | 2.86E-27 |
| S100A14 | downregulated mRNA | 2.05764321 | 3.01E-27 |
| DNASE1 | downregulated mRNA | 2.145190559 | 4.37E-27 |
| NTRK2 | downregulated mRNA | 2.074388929 | 4.71E-27 |
| SLC22A13 | downregulated mRNA | 2.900208671 | 5.98E-27 |
| PCDHB1 | downregulated mRNA | 2.832831628 | 7.65E-27 |
| MOXD1 | downregulated mRNA | 2.272131295 | 8.83E-27 |
| ZCCHC16 | downregulated mRNA | 2.466334437 | 1.14E-26 |
| FGF10 | downregulated mRNA | 3.427313826 | 1.56E-26 |
| HPSE2 | downregulated mRNA | 2.362481098 | 1.74E-26 |
| ANGPTL1 | downregulated mRNA | 2.478409458 | 1.81E-26 |
| KIF5A | downregulated mRNA | 2.113456495 | 2.07E-26 |
| GJA8 | downregulated mRNA | 3.138734225 | 2.14E-26 |
| ADAMTS19 | downregulated mRNA | 4.298396562 | 2.91E-26 |
| SLC7A13 | downregulated mRNA | 4.985577949 | 3.34E-26 |
| GABRA2 | downregulated mRNA | 4.810980839 | 3.63E-26 |
| C1orf116 | downregulated mRNA | 2.859249531 | 3.65E-26 |
| KHDRBS2 | downregulated mRNA | 2.250123537 | 4.98E-26 |
| WNT7A | downregulated mRNA | 2.97786939 | 9.00E-26 |
| PSAT1 | downregulated mRNA | 2.492540632 | 1.19E-25 |
| SLC30A8 | downregulated mRNA | 3.329127841 | 1.31E-25 |
| ADGRF1 | downregulated mRNA | 3.973346407 | 1.51E-25 |
| PIK3C2G | downregulated mRNA | 4.344492974 | 1.76E-25 |
| FAM180A | downregulated mRNA | 2.311708593 | 1.86E-25 |
| TTC29 | downregulated mRNA | 2.386990262 | 2.17E-25 |
| VTCN1 | downregulated mRNA | 3.41825298 | 2.30E-25 |
| L1CAM | downregulated mRNA | 3.139474001 | 2.35E-25 |
| GRHL2 | downregulated mRNA | 3.670935434 | 2.64E-25 |
| C16orf89 | downregulated mRNA | 2.828315049 | 3.60E-25 |
| ZP2 | downregulated mRNA | 2.772624902 | 3.76E-25 |
| FOXN1 | downregulated mRNA | 2.524461022 | 4.04E-25 |
| KRT40 | downregulated mRNA | 3.265018754 | 4.18E-25 |
| B4GALNT3 | downregulated mRNA | 2.191317305 | 4.47E-25 |
| ZPBP | downregulated mRNA | 2.894827516 | 4.55E-25 |
| MT1HL1 | downregulated mRNA | 2.584982394 | 5.34E-25 |
| UTS2R | downregulated mRNA | 4.088783567 | 5.92E-25 |
| DCN | downregulated mRNA | 2.535004404 | 6.76E-25 |
| C21orf62 | downregulated mRNA | 2.225514166 | 7.44E-25 |
| DNMT3L | downregulated mRNA | 2.998303858 | 8.68E-25 |
| FOLR3 | downregulated mRNA | 3.17516398 | 9.26E-25 |
| SIM1 | downregulated mRNA | 2.982557536 | 9.51E-25 |
| OVOL2 | downregulated mRNA | 3.449868129 | 1.31E-24 |
| CRISP2 | downregulated mRNA | 4.66022869 | 1.57E-24 |
| FUT3 | downregulated mRNA | 2.302102317 | 1.70E-24 |
| HS3ST6 | downregulated mRNA | 3.94702676 | 2.16E-24 |
| LRRC19 | downregulated mRNA | 2.000371465 | 2.23E-24 |
| PLEKHD1 | downregulated mRNA | 2.454069822 | 3.40E-24 |
| PDGFRA | downregulated mRNA | 2.359412161 | 3.47E-24 |
| POU2F3 | downregulated mRNA | 2.077801155 | 3.50E-24 |
| SLC22A7 | downregulated mRNA | 3.135496533 | 3.69E-24 |
| MTNR1A | downregulated mRNA | 3.003863413 | 3.92E-24 |
| BSPRY | downregulated mRNA | 2.053708747 | 4.04E-24 |
| KIAA1210 | downregulated mRNA | 2.148892756 | 4.74E-24 |
| HAO2 | downregulated mRNA | 2.491578855 | 5.75E-24 |
| TFCP2L1 | downregulated mRNA | 3.079899566 | 6.48E-24 |
| TREH | downregulated mRNA | 2.318766873 | 7.72E-24 |
| TAGLN3 | downregulated mRNA | 3.160764056 | 8.58E-24 |
| MFI2 | downregulated mRNA | 2.483158158 | 1.22E-23 |
| C2orf40 | downregulated mRNA | 2.042340791 | 1.61E-23 |
| KIAA1549L | downregulated mRNA | 2.055195214 | 1.89E-23 |
| SLC38A3 | downregulated mRNA | 2.931107488 | 2.78E-23 |
| ATP4B | downregulated mRNA | 2.742279042 | 2.92E-23 |
| TMC4 | downregulated mRNA | 2.051048701 | 3.09E-23 |
| SOSTDC1 | downregulated mRNA | 2.906589092 | 3.30E-23 |
| ITLN2 | downregulated mRNA | 2.902353189 | 3.75E-23 |
| DBX2 | downregulated mRNA | 2.53785835 | 4.16E-23 |
| TBL1Y | downregulated mRNA | 3.347734975 | 4.21E-23 |
| SRD5A2 | downregulated mRNA | 2.22645664 | 4.21E-23 |
| RBM11 | downregulated mRNA | 2.492970718 | 5.59E-23 |
| CYP3A4 | downregulated mRNA | 2.07019154 | 6.53E-23 |
| TMPRSS2 | downregulated mRNA | 3.470653105 | 7.84E-23 |
| WDR49 | downregulated mRNA | 2.031721592 | 1.02E-22 |
| LRRN2 | downregulated mRNA | 2.292125674 | 1.08E-22 |
| SGCZ | downregulated mRNA | 4.452723564 | 1.17E-22 |
| HS3ST5 | downregulated mRNA | 3.188933325 | 1.55E-22 |
| PRODH2 | downregulated mRNA | 2.256520595 | 1.59E-22 |
| PLCZ1 | downregulated mRNA | 2.352979672 | 1.64E-22 |
| IGSF5 | downregulated mRNA | 2.23418146 | 1.91E-22 |
| ASB10 | downregulated mRNA | 3.352563878 | 2.00E-22 |
| ADH1B | downregulated mRNA | 2.591872803 | 2.39E-22 |
| NPY2R | downregulated mRNA | 3.699443375 | 3.47E-22 |
| SYT1 | downregulated mRNA | 2.221245169 | 3.52E-22 |
| SYNE4 | downregulated mRNA | 2.165507062 | 3.59E-22 |
| PTGER3 | downregulated mRNA | 2.098403153 | 4.47E-22 |
| RAB25 | downregulated mRNA | 3.410866535 | 5.13E-22 |
| TDRD5 | downregulated mRNA | 2.801096956 | 5.45E-22 |
| FOXE3 | downregulated mRNA | 2.078307972 | 6.91E-22 |
| TNS4 | downregulated mRNA | 2.244889622 | 7.17E-22 |
| C5orf38 | downregulated mRNA | 2.544096249 | 7.39E-22 |
| PLPP4 | downregulated mRNA | 2.465220904 | 9.70E-22 |
| LRP1B | downregulated mRNA | 2.544384765 | 9.95E-22 |
| AADACL4 | downregulated mRNA | 2.687747742 | 1.03E-21 |
| CACNA1S | downregulated mRNA | 2.064743417 | 1.05E-21 |
| PCDH15 | downregulated mRNA | 3.930733587 | 1.14E-21 |
| MIOX | downregulated mRNA | 2.313176557 | 1.65E-21 |
| GATA5 | downregulated mRNA | 2.150187177 | 1.79E-21 |
| CHGA | downregulated mRNA | 2.672405258 | 2.00E-21 |
| SLC7A14 | downregulated mRNA | 2.941333084 | 2.33E-21 |
| GABRG3 | downregulated mRNA | 2.645757837 | 1.28E-20 |
| TUBAL3 | downregulated mRNA | 3.806047889 | 1.31E-20 |
| FOXA3 | downregulated mRNA | 2.807460189 | 1.42E-20 |
| LGSN | downregulated mRNA | 2.064687407 | 1.57E-20 |
| CRYAA | downregulated mRNA | 4.575280329 | 1.68E-20 |
| CPNE4 | downregulated mRNA | 2.597232303 | 1.76E-20 |
| RPRM | downregulated mRNA | 3.285171378 | 1.79E-20 |
| TCEAL2 | downregulated mRNA | 3.121034121 | 2.06E-20 |
| UGT2A1 | downregulated mRNA | 4.666888158 | 3.41E-20 |
| ATP6V1C2 | downregulated mRNA | 2.13769597 | 3.83E-20 |
| BRINP3 | downregulated mRNA | 4.29942061 | 6.02E-20 |
| PLEKHG4B | downregulated mRNA | 2.107058316 | 9.58E-20 |
| TRPM5 | downregulated mRNA | 2.087498237 | 1.32E-19 |
| CCK | downregulated mRNA | 2.70771892 | 1.42E-19 |
| PRSS22 | downregulated mRNA | 3.180049016 | 1.79E-19 |
| DPT | downregulated mRNA | 2.165401827 | 1.95E-19 |
| HMX2 | downregulated mRNA | 5.428845033 | 2.36E-19 |
| NPAP1 | downregulated mRNA | 2.586565918 | 2.63E-19 |
| KIRREL2 | downregulated mRNA | 2.423407241 | 3.12E-19 |
| RDH12 | downregulated mRNA | 2.071832331 | 3.46E-19 |
| AFP | downregulated mRNA | 2.317429934 | 9.11E-19 |
| HMGCS2 | downregulated mRNA | 2.633055176 | 1.26E-18 |
| WT1 | downregulated mRNA | 2.497031402 | 1.67E-18 |
| PAH | downregulated mRNA | 2.885115534 | 1.68E-18 |
| PNMT | downregulated mRNA | 2.443896888 | 2.12E-18 |
| RSPO1 | downregulated mRNA | 2.977518796 | 2.33E-18 |
| CER1 | downregulated mRNA | 2.749302124 | 2.51E-18 |
| ATP10B | downregulated mRNA | 2.045482494 | 2.83E-18 |
| SLC22A6 | downregulated mRNA | 2.899279966 | 2.97E-18 |
| CDH3 | downregulated mRNA | 2.221846772 | 3.28E-18 |
| LHX1 | downregulated mRNA | 3.387686495 | 3.29E-18 |
| GABRP | downregulated mRNA | 2.193867978 | 3.42E-18 |
| TDGF1 | downregulated mRNA | 3.22223645 | 3.49E-18 |
| MUC13 | downregulated mRNA | 2.529937163 | 3.49E-18 |
| ESRP1 | downregulated mRNA | 3.058860293 | 5.74E-18 |
| SLC9A2 | downregulated mRNA | 2.972513731 | 5.82E-18 |
| CNTN5 | downregulated mRNA | 2.516249954 | 6.27E-18 |
| VAT1L | downregulated mRNA | 2.399724532 | 7.59E-18 |
| ARSH | downregulated mRNA | 2.967741109 | 8.88E-18 |
| TOX3 | downregulated mRNA | 2.174260697 | 1.18E-17 |
| BMP7 | downregulated mRNA | 3.104390609 | 1.27E-17 |
| PRDM7 | downregulated mRNA | 2.229823708 | 1.37E-17 |
| KCNMB2 | downregulated mRNA | 2.067358454 | 1.37E-17 |
| SHISA3 | downregulated mRNA | 2.099089056 | 1.43E-17 |
| RAET1L | downregulated mRNA | 2.273016752 | 1.93E-17 |
| CTXN3 | downregulated mRNA | 3.396229953 | 2.25E-17 |
| RNF223 | downregulated mRNA | 2.751487125 | 2.28E-17 |
| APELA | downregulated mRNA | 3.241601683 | 2.31E-17 |
| ZNF98 | downregulated mRNA | 2.100867651 | 2.51E-17 |
| IFNA14 | downregulated mRNA | 4.413251943 | 2.56E-17 |
| AMELY | downregulated mRNA | 3.753232345 | 2.87E-17 |
| IL1RL1 | downregulated mRNA | 2.079920587 | 3.44E-17 |
| WSCD2 | downregulated mRNA | 2.218207765 | 3.58E-17 |
| RDH8 | downregulated mRNA | 3.821339013 | 3.89E-17 |
| SLC4A9 | downregulated mRNA | 3.095544528 | 4.57E-17 |
| SERPINA4 | downregulated mRNA | 2.78788118 | 4.96E-17 |
| GABRA4 | downregulated mRNA | 2.907029957 | 4.99E-17 |
| KCNQ2 | downregulated mRNA | 2.571952164 | 5.38E-17 |
| FAM83B | downregulated mRNA | 3.413973416 | 6.73E-17 |
| PLG | downregulated mRNA | 3.211601358 | 1.09E-16 |
| LINGO2 | downregulated mRNA | 2.42266471 | 1.15E-16 |
| PSKH2 | downregulated mRNA | 4.090124226 | 1.21E-16 |
| CHRNA4 | downregulated mRNA | 3.20270728 | 1.51E-16 |
| RBP3 | downregulated mRNA | 2.642700623 | 1.63E-16 |
| ANKRD63 | downregulated mRNA | 2.335917252 | 1.88E-16 |
| KLK5 | downregulated mRNA | 3.509151212 | 1.89E-16 |
| CCL11 | downregulated mRNA | 2.408590057 | 2.29E-16 |
| PRL | downregulated mRNA | 2.125694895 | 2.31E-16 |
| PCDHA12 | downregulated mRNA | 2.036723111 | 2.46E-16 |
| GREM1 | downregulated mRNA | 2.40934376 | 2.78E-16 |
| C1orf87 | downregulated mRNA | 2.755482396 | 4.67E-16 |
| LGR5 | downregulated mRNA | 2.583996329 | 5.04E-16 |
| PPP1R1A | downregulated mRNA | 2.648286676 | 6.13E-16 |
| APOH | downregulated mRNA | 2.495706667 | 8.06E-16 |
| PSCA | downregulated mRNA | 2.538675523 | 1.03E-15 |
| TFAP2A | downregulated mRNA | 2.014386154 | 1.14E-15 |
| SIX4 | downregulated mRNA | 2.188968773 | 1.53E-15 |
| DNER | downregulated mRNA | 2.506930859 | 1.91E-15 |
| PLP1 | downregulated mRNA | 2.091828198 | 2.02E-15 |
| SH3GL2 | downregulated mRNA | 2.697031023 | 2.15E-15 |
| SLITRK3 | downregulated mRNA | 2.645049706 | 2.27E-15 |
| RORB | downregulated mRNA | 2.185192598 | 2.66E-15 |
| CLCNKB | downregulated mRNA | 3.140970534 | 3.04E-15 |
| PCSK2 | downregulated mRNA | 2.34288905 | 3.40E-15 |
| HTR3D | downregulated mRNA | 2.663421298 | 3.85E-15 |
| GCM1 | downregulated mRNA | 2.003123033 | 3.96E-15 |
| C1orf64 | downregulated mRNA | 2.928766496 | 4.08E-15 |
| SORCS1 | downregulated mRNA | 2.250906176 | 5.83E-15 |
| SLC4A1 | downregulated mRNA | 3.193424297 | 6.39E-15 |
| TMEM174 | downregulated mRNA | 2.258560441 | 6.56E-15 |
| PHF21B | downregulated mRNA | 2.23019562 | 6.62E-15 |
| PAGE4 | downregulated mRNA | 3.547073599 | 6.81E-15 |
| C1orf168 | downregulated mRNA | 2.058105291 | 9.16E-15 |
| RBFOX1 | downregulated mRNA | 2.34085171 | 1.34E-14 |
| SPDYC | downregulated mRNA | 2.087525181 | 1.40E-14 |
| ZNF728 | downregulated mRNA | 2.393747701 | 1.91E-14 |
| KCNB2 | downregulated mRNA | 2.524032647 | 2.18E-14 |
| LYPD6B | downregulated mRNA | 2.715775311 | 2.30E-14 |
| XAGE2 | downregulated mRNA | 3.14018739 | 2.31E-14 |
| IRS4 | downregulated mRNA | 2.919847068 | 2.68E-14 |
| FAM83E | downregulated mRNA | 2.341219642 | 3.72E-14 |
| SCN2B | downregulated mRNA | 2.262450668 | 3.90E-14 |
| DLK1 | downregulated mRNA | 2.988206196 | 3.96E-14 |
| DIRAS1 | downregulated mRNA | 2.194190616 | 4.51E-14 |
| GUCA1C | downregulated mRNA | 3.173159196 | 4.61E-14 |
| CLEC4M | downregulated mRNA | 2.10731561 | 4.84E-14 |
| CWH43 | downregulated mRNA | 2.71642855 | 5.66E-14 |
| CYP4F3 | downregulated mRNA | 2.154446421 | 6.26E-14 |
| NOTUM | downregulated mRNA | 2.187560338 | 8.62E-14 |
| PI16 | downregulated mRNA | 2.268732324 | 8.89E-14 |
| RAB3B | downregulated mRNA | 2.0981204 | 1.27E-13 |
| OXGR1 | downregulated mRNA | 2.572184226 | 1.33E-13 |
| AMER2 | downregulated mRNA | 2.450135118 | 1.37E-13 |
| SLC7A4 | downregulated mRNA | 2.011183595 | 1.63E-13 |
| LPA | downregulated mRNA | 2.158621694 | 1.91E-13 |
| SFTA2 | downregulated mRNA | 2.440384472 | 2.11E-13 |
| PRR15L | downregulated mRNA | 2.077804828 | 2.25E-13 |
| SCGB2A1 | downregulated mRNA | 2.161246707 | 2.39E-13 |
| FRMD7 | downregulated mRNA | 3.642780998 | 2.87E-13 |
| FSHB | downregulated mRNA | 3.630757481 | 3.02E-13 |
| FRG2 | downregulated mRNA | 3.994854791 | 3.09E-13 |
| ATP6V1B1 | downregulated mRNA | 2.659086899 | 3.80E-13 |
| FRG2B | downregulated mRNA | 4.142171342 | 3.89E-13 |
| KLHL1 | downregulated mRNA | 3.146176009 | 6.13E-13 |
| SMIM22 | downregulated mRNA | 2.140174227 | 8.75E-13 |
| SYNPR | downregulated mRNA | 3.180789446 | 1.17E-12 |
| ZG16 | downregulated mRNA | 2.39384784 | 1.27E-12 |
| TRIM40 | downregulated mRNA | 2.021108503 | 1.34E-12 |
| ETNPPL | downregulated mRNA | 2.440082458 | 1.46E-12 |
| AQP5 | downregulated mRNA | 2.493898262 | 1.56E-12 |
| NDNF | downregulated mRNA | 2.427102326 | 1.87E-12 |
| TMEM132C | downregulated mRNA | 2.015007977 | 1.97E-12 |
| FGFBP1 | downregulated mRNA | 2.984186384 | 2.21E-12 |
| TMEM61 | downregulated mRNA | 2.793389371 | 2.45E-12 |
| ATP2B3 | downregulated mRNA | 2.438876352 | 2.97E-12 |
| KRT24 | downregulated mRNA | 2.219615793 | 3.24E-12 |
| SLC7A3 | downregulated mRNA | 2.440761048 | 3.27E-12 |
| KCNA4 | downregulated mRNA | 2.4615182 | 3.69E-12 |
| ZNF804B | downregulated mRNA | 2.854270715 | 8.33E-12 |
| HAO1 | downregulated mRNA | 3.248250931 | 1.03E-11 |
| UMODL1 | downregulated mRNA | 2.181253193 | 1.08E-11 |
| KRTAP17-1 | downregulated mRNA | 2.174051915 | 1.44E-11 |
| KCNJ13 | downregulated mRNA | 2.279898348 | 1.58E-11 |
| TAC1 | downregulated mRNA | 2.883599259 | 1.78E-11 |
| GPR22 | downregulated mRNA | 2.229101928 | 1.96E-11 |
| CADM2 | downregulated mRNA | 2.033303122 | 2.40E-11 |
| FGF9 | downregulated mRNA | 2.86408729 | 2.96E-11 |
| SLC22A12 | downregulated mRNA | 2.035783526 | 3.26E-11 |
| CYP1A1 | downregulated mRNA | 2.893719889 | 4.81E-11 |
| CCDC185 | downregulated mRNA | 2.782112915 | 5.32E-11 |
| RP11-520P18.5 | downregulated mRNA | 4.139962098 | 6.01E-11 |
| ARL14EPL | downregulated mRNA | 2.488629828 | 7.44E-11 |
| TFAP2C | downregulated mRNA | 2.050539797 | 8.13E-11 |
| RBBP8NL | downregulated mRNA | 3.191658906 | 8.67E-11 |
| PDX1 | downregulated mRNA | 2.690997845 | 8.70E-11 |
| SLC6A19 | downregulated mRNA | 2.196710186 | 1.28E-10 |
| UNC13C | downregulated mRNA | 2.005095702 | 1.34E-10 |
| CDH9 | downregulated mRNA | 2.230243435 | 1.42E-10 |
| CLEC3A | downregulated mRNA | 2.456855709 | 2.13E-10 |
| ABCG8 | downregulated mRNA | 2.057816025 | 2.22E-10 |
| NUPR2 | downregulated mRNA | 2.915844598 | 2.85E-10 |
| HMX3 | downregulated mRNA | 2.513940906 | 4.11E-10 |
| MYF6 | downregulated mRNA | 2.163885687 | 4.49E-10 |
| AGR2 | downregulated mRNA | 2.630801266 | 4.71E-10 |
| SVOPL | downregulated mRNA | 2.070175732 | 4.96E-10 |
| SLCO1A2 | downregulated mRNA | 2.230122747 | 9.57E-10 |
| AQP6 | downregulated mRNA | 2.715253 | 1.04E-09 |
| CLDN8 | downregulated mRNA | 3.705822856 | 1.18E-09 |
| TMEM213 | downregulated mRNA | 2.8311971 | 1.41E-09 |
| LSAMP | downregulated mRNA | 2.087413568 | 1.58E-09 |
| PATE1 | downregulated mRNA | 2.908376813 | 1.85E-09 |
| CNTNAP4 | downregulated mRNA | 2.137838164 | 1.96E-09 |
| PLA2G3 | downregulated mRNA | 2.56904802 | 4.99E-09 |
| RP11-385J1.3 | downregulated mRNA | 2.384668034 | 5.07E-09 |
| CALCA | downregulated mRNA | 3.053773063 | 5.89E-09 |
| ZIC3 | downregulated mRNA | 2.213845786 | 6.24E-09 |
| VWA5B1 | downregulated mRNA | 2.07229065 | 1.08E-08 |
| ADGRA1 | downregulated mRNA | 2.424782978 | 1.27E-08 |
| TH | downregulated mRNA | 2.026252268 | 1.88E-08 |
| PCP4 | downregulated mRNA | 2.341928261 | 2.55E-08 |
| ISX | downregulated mRNA | 2.616441535 | 2.90E-08 |
| HOXB1 | downregulated mRNA | 2.202549294 | 3.20E-08 |
| DEFB132 | downregulated mRNA | 3.745780203 | 3.89E-08 |
| FOXD3 | downregulated mRNA | 2.211827654 | 4.17E-08 |
| BSND | downregulated mRNA | 3.266830725 | 4.43E-08 |
| GPR50 | downregulated mRNA | 2.430196978 | 5.19E-08 |
| LCE2D | downregulated mRNA | 3.532166931 | 6.09E-08 |
| NXPH2 | downregulated mRNA | 3.514768129 | 6.69E-08 |
| CH507-152C13.3 | downregulated mRNA | 3.874003885 | 8.42E-08 |
| CEACAM7 | downregulated mRNA | 2.456631352 | 1.16E-07 |
| PMP2 | downregulated mRNA | 2.424097555 | 1.19E-07 |
| ROS1 | downregulated mRNA | 2.414034457 | 1.61E-07 |
| AGTR2 | downregulated mRNA | 2.616944503 | 3.27E-07 |
| CDH19 | downregulated mRNA | 2.063233303 | 3.36E-07 |
| EPHA5 | downregulated mRNA | 2.075123046 | 3.91E-07 |
| NR0B2 | downregulated mRNA | 2.700085238 | 4.16E-07 |
| HTR3B | downregulated mRNA | 2.672496324 | 5.62E-07 |
| CAPZA3 | downregulated mRNA | 2.48499532 | 1.09E-06 |
| DEFB125 | downregulated mRNA | 3.05874018 | 1.11E-06 |
| MC2R | downregulated mRNA | 2.951855769 | 1.41E-06 |
| FAM216B | downregulated mRNA | 2.099292719 | 1.69E-06 |
| PFN3 | downregulated mRNA | 2.291982804 | 2.41E-06 |
| CDH18 | downregulated mRNA | 2.084206817 | 4.11E-06 |
| PLA2G4F | downregulated mRNA | 2.218199236 | 6.76E-06 |
| GCGR | downregulated mRNA | 2.229949737 | 8.47E-06 |
| CST11 | downregulated mRNA | 2.346957618 | 8.52E-06 |
| DEFB127 | downregulated mRNA | 2.663411709 | 1.02E-05 |
| SPRR2A | downregulated mRNA | 2.124654982 | 1.16E-05 |
| RHBG | downregulated mRNA | 2.006299365 | 1.39E-05 |
| ODAM | downregulated mRNA | 2.849340068 | 1.71E-05 |
| LRRC52 | downregulated mRNA | 2.657911852 | 1.96E-05 |
| SALL3 | downregulated mRNA | 2.317555996 | 2.32E-05 |
| SCGB1C2 | downregulated mRNA | 2.196616951 | 4.59E-05 |
| C20orf85 | downregulated mRNA | 2.621823676 | 5.35E-05 |
| CRABP1 | downregulated mRNA | 2.010095408 | 0.000115474 |
| MYF5 | downregulated mRNA | 2.280671878 | 0.00026903 |
| POU3F4 | downregulated mRNA | 2.235815735 | 0.001051638 |
| FGF4 | downregulated mRNA | 2.001704738 | 0.001385825 |

**Table S3. The subcellular distribution of lncRNAs in the ceRNA network**

| **Gene** | **Subcellular Distribution** |
| --- | --- |
| AC000095.11 | Nuclear |
| AC002059.10 | Nuclear |
| AC003090.1 | Nuclear |
| AC006145.4 | Nuclear |
| AC006262.4 | Nuclear |
| AC007255.8 | Nuclear |
| AC007278.2 | Nuclear |
| AC010729.1 | Nuclear |
| AC011286.1 | Nuclear |
| AC011893.3 | Nuclear |
| AC012123.1 | Nuclear |
| AC013460.1 | Nuclear |
| AC018742.1 | Nuclear |
| AC022431.3 | Nuclear |
| AC034110.1 | Nuclear |
| AC068138.1 | Nuclear |
| AC069363.1 | Nuclear |
| AC073130.1 | Nuclear |
| AC073257.2 | Nuclear |
| AC073321.4 | Nuclear |
| AC078852.2 | Nuclear |
| AC078883.3 | Nuclear |
| AC090505.6 | Nuclear |
| AC092620.3 | Nuclear |
| AC092675.3 | Nuclear |
| AC097382.5 | Nuclear |
| AC099552.4 | Nuclear |
| AC104820.2 | Nuclear |
| AC105402.4 | Nuclear |
| AC116614.1 | Nuclear |
| AC123023.1 | Nuclear |
| AC138430.4 | Nuclear |
| AC144831.1 | Nuclear |
| AC144831.3 | Nuclear |
| ADAM20P1 | Nuclear |
| AF011889.2 | Nuclear |
| AF064858.7 | Nuclear |
| AF064858.8 | Nuclear |
| AF127936.5 | Nuclear |
| AF131215.4 | Nuclear |
| AP000233.4 | Nuclear |
| AP000593.7 | Nuclear |
| AP000696.2 | Nuclear |
| AP001056.1 | Nuclear |
| AP001059.5 | Nuclear |
| AP003774.1 | Nuclear |
| ASMTL-AS1 | Nuclear |
| BRWD1-IT1 | Nuclear |
| BX255923.3 | Nuclear |
| C15orf56 | Nuclear |
| C17orf77 | Nuclear |
| C20orf197 | Nuclear |
| C2-AS1 | Nuclear |
| C9orf139 | Nuclear |
| CALML3-AS1 | Nuclear |
| CASC20 | Nuclear |
| CDKN2B-AS1 | Nuclear |
| CFLAR-AS1 | Nuclear |
| CH17-360D5.3 | Nuclear |
| CLDN10-AS1 | Nuclear |
| CTA-228A9.3 | Nuclear |
| CTB-113P19.1 | Nuclear |
| CTB-138E5.1 | Nuclear |
| CTB-186G2.1 | Nuclear |
| CTB-193M12.3 | Nuclear |
| CTB-1I21.1 | Nuclear |
| CTB-26E19.1 | Nuclear |
| CTC-232P5.3 | Nuclear |
| CTC-344H19.4 | Nuclear |
| CTD-2008P7.8 | Nuclear |
| CTD-2026K11.6 | Nuclear |
| CTD-2034I4.2 | Nuclear |
| CTD-2036P10.6 | Nuclear |
| CTD-2105E13.16 | Nuclear |
| CTD-2116N20.1 | Nuclear |
| CTD-2223O18.1 | Nuclear |
| CTD-2251F13.1 | Nuclear |
| CTD-2297D10.2 | Nuclear |
| CTD-2515H24.2 | Nuclear |
| CTD-2527I21.15 | Nuclear |
| CTD-2587H19.2 | Nuclear |
| CTD-2626G11.2 | Nuclear |
| CTD-3023L14.2 | Nuclear |
| CTD-3128G10.7 | Nuclear |
| DGCR5 | Nuclear |
| DGCR9 | Nuclear |
| DKFZp434J0226 | Nuclear |
| DKFZP434L187 | Nuclear |
| DLEU7-AS1 | Nuclear |
| F11-AS1 | Nuclear |
| FAM167A-AS1 | Nuclear |
| FAM230C | Nuclear |
| FLJ26245 | Nuclear |
| GAS1RR | Nuclear |
| GATA3-AS1 | Nuclear |
| GATM-AS1 | Nuclear |
| GPC5-IT1 | Nuclear |
| HOTTIP | Nuclear |
| IFNG-AS1 | Nuclear |
| IL21R-AS1 | Nuclear |
| INHBA-AS1 | Nuclear |
| JAKMIP2-AS1 | Nuclear |
| KB-1562D12.1 | Nuclear |
| KCNQ5-IT1 | Nuclear |
| LA16c-349E10.1 | Nuclear |
| LA16c-361A3.3 | Nuclear |
| LA16c-381G6.1 | Nuclear |
| LA16c-390H2.4 | Nuclear |
| LINC00160 | Nuclear |
| LINC00165 | Nuclear |
| LINC00173 | Nuclear |
| LINC00202-2 | Nuclear |
| LINC00284 | Nuclear |
| LINC00298 | Nuclear |
| LINC00299 | Nuclear |
| LINC00313 | Nuclear |
| LINC00342 | Nuclear |
| LINC00371 | Nuclear |
| LINC00473 | Nuclear |
| LINC00475 | Nuclear |
| LINC00487 | Nuclear |
| LINC00521 | Nuclear |
| LINC00547 | Nuclear |
| LINC00588 | Nuclear |
| LINC00602 | Nuclear |
| LINC00607 | Nuclear |
| LINC00675 | Nuclear |
| LINC00698 | Nuclear |
| LINC00824 | Nuclear |
| LINC00856 | Nuclear |
| LINC00871 | Nuclear |
| LINC00879 | Nuclear |
| LINC00881 | Nuclear |
| LINC00895 | Nuclear |
| LINC01033 | Nuclear |
| LINC01127 | Nuclear |
| LINC01151 | Nuclear |
| LINC01163 | Nuclear |
| LINC01206 | Nuclear |
| LINC01226 | Nuclear |
| LINC01268 | Nuclear |
| LINC01271 | Nuclear |
| LINC01272 | Nuclear |
| LINC01305 | Nuclear |
| LINC01397 | Nuclear |
| LINC01429 | Nuclear |
| LINC01433 | Nuclear |
| LINC01526 | Nuclear |
| LINC01529 | Nuclear |
| LINC01541 | Nuclear |
| LINC01544 | Nuclear |
| LINC01555 | Nuclear |
| LINC01561 | Nuclear |
| LINC01614 | Nuclear |
| LINGO1-AS1 | Nuclear |
| MAFA-AS1 | Nuclear |
| MTUS2-AS1 | Nuclear |
| MUC2 | Nuclear |
| NALCN-AS1 | Nuclear |
| NRIR | Nuclear |
| PHKA2-AS1 | Nuclear |
| PIK3CD-AS1 | Nuclear |
| PP12613 | Nuclear |
| PVT1 | Nuclear |
| PWRN1 | Nuclear |
| PWRN3 | Nuclear |
| RAPGEF4-AS1 | Nuclear |
| RORB-AS1 | Nuclear |
| RP11-1029J19.4 | Nuclear |
| RP11-1069G10.1 | Nuclear |
| RP11-107N7.1 | Nuclear |
| RP11-1094M14.5 | Nuclear |
| RP11-10L7.1 | Nuclear |
| RP11-10N16.2 | Nuclear |
| RP11-1151B14.1 | Nuclear |
| RP11-1151B14.4 | Nuclear |
| RP11-1151B14.5 | Nuclear |
| RP11-115D19.3 | Nuclear |
| RP11-116D2.1 | Nuclear |
| RP11-116O18.1 | Nuclear |
| RP11-118K6.3 | Nuclear |
| RP11-1191J2.2 | Nuclear |
| RP11-120K18.2 | Nuclear |
| RP11-123K3.9 | Nuclear |
| RP11-124N14.3 | Nuclear |
| RP11-129I19.2 | Nuclear |
| RP11-131N11.4 | Nuclear |
| RP11-132E11.2 | Nuclear |
| RP11-138J23.1 | Nuclear |
| RP11-1399P15.1 | Nuclear |
| RP11-143A12.3 | Nuclear |
| RP11-145H9.3 | Nuclear |
| RP11-149I2.4 | Nuclear |
| RP11-14C10.3 | Nuclear |
| RP11-14J7.6 | Nuclear |
| RP11-168K11.3 | Nuclear |
| RP11-16E12.2 | Nuclear |
| RP11-172F10.1 | Nuclear |
| RP11-177H13.2 | Nuclear |
| RP11-180C16.1 | Nuclear |
| RP11-181E10.3 | Nuclear |
| RP11-18F14.4 | Nuclear |
| RP11-196G11.2 | Nuclear |
| RP11-20J15.3 | Nuclear |
| RP11-212I21.2 | Nuclear |
| RP11-21A7A.3 | Nuclear |
| RP11-227G15.12 | Nuclear |
| RP11-22H5.2 | Nuclear |
| RP11-24F11.2 | Nuclear |
| RP11-258F1.1 | Nuclear |
| RP11-260O18.1 | Nuclear |
| RP11-261P9.4 | Nuclear |
| RP11-264E20.1 | Nuclear |
| RP11-264E20.2 | Nuclear |
| RP11-267L5.1 | Nuclear |
| RP11-274H2.3 | Nuclear |
| RP11-274H2.5 | Nuclear |
| RP11-274H24.1 | Nuclear |
| RP11-282I1.1 | Nuclear |
| RP11-290F5.1 | Nuclear |
| RP11-290L1.3 | Nuclear |
| RP11-290O12.2 | Nuclear |
| RP11-29P20.1 | Nuclear |
| RP11-2E11.5 | Nuclear |
| RP11-310H4.2 | Nuclear |
| RP11-310H4.3 | Nuclear |
| RP11-311F12.1 | Nuclear |
| RP11-313P18.2 | Nuclear |
| RP11-319G9.5 | Nuclear |
| RP11-322E11.2 | Nuclear |
| RP11-326C3.2 | Nuclear |
| RP11-327F22.1 | Nuclear |
| RP11-327F22.6 | Nuclear |
| RP11-327I22.5 | Nuclear |
| RP11-343J3.2 | Nuclear |
| RP11-353N4.5 | Nuclear |
| RP11-354K1.1 | Nuclear |
| RP11-35J10.6 | Nuclear |
| RP11-365O16.6 | Nuclear |
| RP11-366L20.2 | Nuclear |
| RP11-369C8.1 | Nuclear |
| RP11-377G16.2 | Nuclear |
| RP11-379F12.4 | Nuclear |
| RP11-380G5.2 | Nuclear |
| RP11-380J14.1 | Nuclear |
| RP11-384F7.2 | Nuclear |
| RP11-395B7.2 | Nuclear |
| RP11-395I14.2 | Nuclear |
| RP11-3P17.4 | Nuclear |
| RP11-403I13.5 | Nuclear |
| RP11-408O19.5 | Nuclear |
| RP11-412P11.1 | Nuclear |
| RP11-417L19.2 | Nuclear |
| RP11-41O4.2 | Nuclear |
| RP11-426C22.4 | Nuclear |
| RP11-429B14.4 | Nuclear |
| RP1-142L7.8 | Nuclear |
| RP1-142L7.9 | Nuclear |
| RP11-434E6.4 | Nuclear |
| RP11-438B23.2 | Nuclear |
| RP11-439C15.4 | Nuclear |
| RP11-445N18.5 | Nuclear |
| RP11-449J21.5 | Nuclear |
| RP11-451B8.1 | Nuclear |
| RP11-455F5.6 | Nuclear |
| RP11-459O16.8 | Nuclear |
| RP11-45A17.4 | Nuclear |
| RP11-45M22.5 | Nuclear |
| RP11-467P9.1 | Nuclear |
| RP11-46C24.3 | Nuclear |
| RP11-471M2.3 | Nuclear |
| RP11-474B16.1 | Nuclear |
| RP11-476M19.2 | Nuclear |
| RP11-478J18.2 | Nuclear |
| RP11-486M23.1 | Nuclear |
| RP11-488C13.6 | Nuclear |
| RP11-493L12.5 | Nuclear |
| RP11-494H4.3 | Nuclear |
| RP11-494M8.4 | Nuclear |
| RP11-511B23.2 | Nuclear |
| RP11-521I2.3 | Nuclear |
| RP11-524N5.1 | Nuclear |
| RP11-527L4.2 | Nuclear |
| RP11-531H8.1 | Nuclear |
| RP11-536G4.2 | Nuclear |
| RP11-540O11.8 | Nuclear |
| RP11-543P15.3 | Nuclear |
| RP11-551L14.4 | Nuclear |
| RP11-554A11.4 | Nuclear |
| RP11-560I19.1 | Nuclear |
| RP11-565A3.2 | Nuclear |
| RP11-568J23.8 | Nuclear |
| RP11-569G13.2 | Nuclear |
| RP11-573D15.8 | Nuclear |
| RP11-57A1.1 | Nuclear |
| RP11-586D19.2 | Nuclear |
| RP11-586K2.1 | Nuclear |
| RP11-598F7.3 | Nuclear |
| RP11-608O21.1 | Nuclear |
| RP11-60A8.1 | Nuclear |
| RP11-61F12.1 | Nuclear |
| RP11-61I13.3 | Nuclear |
| RP11-632K5.3 | Nuclear |
| RP11-663P9.1 | Nuclear |
| RP11-675F6.4 | Nuclear |
| RP1-167G20.1 | Nuclear |
| RP11-680F20.6 | Nuclear |
| RP11-6O2.4 | Nuclear |
| RP11-70D24.3 | Nuclear |
| RP1-170O19.24 | Nuclear |
| RP11-713N11.5 | Nuclear |
| RP11-725G5.2 | Nuclear |
| RP11-734K21.2 | Nuclear |
| RP11-734K21.5 | Nuclear |
| RP11-757O6.1 | Nuclear |
| RP11-77I22.2 | Nuclear |
| RP11-785G17.1 | Nuclear |
| RP11-789C17.1 | Nuclear |
| RP11-794G24.1 | Nuclear |
| RP11-817J15.3 | Nuclear |
| RP11-834C11.5 | Nuclear |
| RP11-849I19.1 | Nuclear |
| RP11-84C10.4 | Nuclear |
| RP11-861E21.2 | Nuclear |
| RP11-866E20.3 | Nuclear |
| RP11-893F2.5 | Nuclear |
| RP11-909N17.2 | Nuclear |
| RP11-93I21.3 | Nuclear |
| RP11-94C24.13 | Nuclear |
| RP11-964E11.2 | Nuclear |
| RP11-96A15.1 | Nuclear |
| RP11-982M15.7 | Nuclear |
| RP11-9N12.2 | Nuclear |
| RP1-278C19.8 | Nuclear |
| RP13-192B19.2 | Nuclear |
| RP13-297E16.4 | Nuclear |
| RP13-39P12.3 | Nuclear |
| RP13-516M14.10 | Nuclear |
| RP13-714J12.1 | Nuclear |
| RP13-895J2.3 | Nuclear |
| RP1-45C12.1 | Nuclear |
| RP1-79C4.4 | Nuclear |
| RP1-80N2.2 | Nuclear |
| RP3-340N1.2 | Nuclear |
| RP3-393E18.2 | Nuclear |
| RP3-462D8.2 | Nuclear |
| RP4-536B24.4 | Nuclear |
| RP4-539M6.22 | Nuclear |
| RP4-545L17.12 | Nuclear |
| RP4-566L20.1 | Nuclear |
| RP4-644L1.2 | Nuclear |
| RP4-646N3.1 | Nuclear |
| RP4-647J21.1 | Nuclear |
| RP4-655J12.4 | Nuclear |
| RP4-684O24.5 | Nuclear |
| RP4-753M9.1 | Nuclear |
| RP4-764O22.1 | Nuclear |
| RP4-806M20.4 | Nuclear |
| RP4-813D12.3 | Nuclear |
| RP5-1009E24.8 | Nuclear |
| RP5-1029K10.2 | Nuclear |
| RP5-1031D4.2 | Nuclear |
| RP5-1039K5.16 | Nuclear |
| RP5-1056H1.2 | Nuclear |
| RP5-1065P14.2 | Nuclear |
| RP5-1073O3.2 | Nuclear |
| RP5-1120P11.3 | Nuclear |
| RP5-837J1.4 | Nuclear |
| RP5-858L17.1 | Nuclear |
| RP5-881P19.7 | Nuclear |
| RP5-902P8.10 | Nuclear |
| RP5-921G16.1 | Nuclear |
| RP5-943J3.1 | Nuclear |
| RP5-998N21.4 | Nuclear |
| RP6-24A23.3 | Nuclear |
| RRS1-AS1 | Nuclear |
| SCHLAP1 | Nuclear |
| SSTR5-AS1 | Nuclear |
| TARID | Nuclear |
| TCL6 | Nuclear |
| TNK2-AS1 | Nuclear |
| U95743.1 | Nuclear |
| XX-C00717C00720L.1 | Nuclear |
| XXyac-YX65C7_A.3 | Nuclear |
| YEATS2-AS1 | Nuclear |
| ABHD11-AS1 | Nuclear and Cytoplasm |
| AC002480.2 | Nuclear and Cytoplasm |
| AC003003.5 | Nuclear and Cytoplasm |
| AC003088.1 | Nuclear and Cytoplasm |
| AC003092.1 | Nuclear and Cytoplasm |
| AC004988.1 | Nuclear and Cytoplasm |
| AC005082.12 | Nuclear and Cytoplasm |
| AC005264.2 | Nuclear and Cytoplasm |
| AC005306.3 | Nuclear and Cytoplasm |
| AC005785.2 | Nuclear and Cytoplasm |
| AC006262.5 | Nuclear and Cytoplasm |
| AC007278.3 | Nuclear and Cytoplasm |
| AC008088.4 | Nuclear and Cytoplasm |
| AC008592.5 | Nuclear and Cytoplasm |
| AC008746.12 | Nuclear and Cytoplasm |
| AC009133.17 | Nuclear and Cytoplasm |
| AC009264.1 | Nuclear and Cytoplasm |
| AC009501.4 | Nuclear and Cytoplasm |
| AC009784.3 | Nuclear and Cytoplasm |
| AC013463.2 | Nuclear and Cytoplasm |
| AC015849.16 | Nuclear and Cytoplasm |
| AC016735.2 | Nuclear and Cytoplasm |
| AC019181.2 | Nuclear and Cytoplasm |
| AC026471.6 | Nuclear and Cytoplasm |
| AC074286.1 | Nuclear and Cytoplasm |
| AC079466.1 | Nuclear and Cytoplasm |
| AC093484.4 | Nuclear and Cytoplasm |
| AC093642.3 | Nuclear and Cytoplasm |
| AC131056.3 | Nuclear and Cytoplasm |
| AC133644.2 | Nuclear and Cytoplasm |
| AC137932.6 | Nuclear and Cytoplasm |
| AC147651.4 | Nuclear and Cytoplasm |
| AC156455.1 | Nuclear and Cytoplasm |
| AC159540.1 | Nuclear and Cytoplasm |
| ADAMTS19-AS1 | Nuclear and Cytoplasm |
| AFAP1-AS1 | Nuclear and Cytoplasm |
| AL161668.5 | Nuclear and Cytoplasm |
| APCDD1L-AS1 | Nuclear and Cytoplasm |
| ARHGEF26-AS1 | Nuclear and Cytoplasm |
| ASAP1-IT2 | Nuclear and Cytoplasm |
| ASH1L-IT1 | Nuclear and Cytoplasm |
| BVES-AS1 | Nuclear and Cytoplasm |
| C20orf203 | Nuclear and Cytoplasm |
| C5orf58 | Nuclear and Cytoplasm |
| CASC11 | Nuclear and Cytoplasm |
| CH17-360D5.1 | Nuclear and Cytoplasm |
| CH507-513H4.3 | Nuclear and Cytoplasm |
| CITF22-62D4.1 | Nuclear and Cytoplasm |
| CTA-392C11.1 | Nuclear and Cytoplasm |
| CTA-414D7.1 | Nuclear and Cytoplasm |
| CTB-181H17.1 | Nuclear and Cytoplasm |
| CTC-251D13.1 | Nuclear and Cytoplasm |
| CTC-273B12.10 | Nuclear and Cytoplasm |
| CTC-523E23.5 | Nuclear and Cytoplasm |
| CTD-2008P7.9 | Nuclear and Cytoplasm |
| CTD-2015G9.2 | Nuclear and Cytoplasm |
| CTD-2020K17.1 | Nuclear and Cytoplasm |
| CTD-2035E11.5 | Nuclear and Cytoplasm |
| CTD-2118P12.1 | Nuclear and Cytoplasm |
| CTD-2196E14.5 | Nuclear and Cytoplasm |
| CTD-2201E18.5 | Nuclear and Cytoplasm |
| CTD-2228K2.7 | Nuclear and Cytoplasm |
| CTD-2231H16.1 | Nuclear and Cytoplasm |
| CTD-2265O21.3 | Nuclear and Cytoplasm |
| CTD-2287O16.4 | Nuclear and Cytoplasm |
| CTD-2354A18.1 | Nuclear and Cytoplasm |
| CTD-2357A8.3 | Nuclear and Cytoplasm |
| CTD-2377O17.1 | Nuclear and Cytoplasm |
| CTD-2521M24.5 | Nuclear and Cytoplasm |
| CTD-2530N21.5 | Nuclear and Cytoplasm |
| CTD-2537I9.13 | Nuclear and Cytoplasm |
| CTD-2540F13.2 | Nuclear and Cytoplasm |
| CTD-2542L18.1 | Nuclear and Cytoplasm |
| CTD-2545H1.2 | Nuclear and Cytoplasm |
| CTD-2576D5.4 | Nuclear and Cytoplasm |
| CTD-2587H24.5 | Nuclear and Cytoplasm |
| CTD-2589M5.5 | Nuclear and Cytoplasm |
| CTD-2616J11.2 | Nuclear and Cytoplasm |
| CTD-2616J11.3 | Nuclear and Cytoplasm |
| CTD-3064M3.7 | Nuclear and Cytoplasm |
| DARS-AS1 | Nuclear and Cytoplasm |
| DEPDC1-AS1 | Nuclear and Cytoplasm |
| DLG1-AS1 | Nuclear and Cytoplasm |
| DSCAM-AS1 | Nuclear and Cytoplasm |
| DUXAP8 | Nuclear and Cytoplasm |
| EML2-AS1 | Nuclear and Cytoplasm |
| ENO1-IT1 | Nuclear and Cytoplasm |
| EPHA5-AS1 | Nuclear and Cytoplasm |
| FAM13A-AS1 | Nuclear and Cytoplasm |
| FGF12-AS2 | Nuclear and Cytoplasm |
| FIRRE | Nuclear and Cytoplasm |
| FLJ35934 | Nuclear and Cytoplasm |
| FTO-IT1 | Nuclear and Cytoplasm |
| GACAT2 | Nuclear and Cytoplasm |
| GAPLINC | Nuclear and Cytoplasm |
| GAS6-AS1 | Nuclear and Cytoplasm |
| GS1-600G8.5 | Nuclear and Cytoplasm |
| HIF1A-AS2 | Nuclear and Cytoplasm |
| HP09025 | Nuclear and Cytoplasm |
| IL21-AS1 | Nuclear and Cytoplasm |
| ITGB2-AS1 | Nuclear and Cytoplasm |
| KB-1836B5.1 | Nuclear and Cytoplasm |
| KB-1980E6.3 | Nuclear and Cytoplasm |
| KCNK4-TEX40 | Nuclear and Cytoplasm |
| KCNMB2-AS1 | Nuclear and Cytoplasm |
| LA16c-358B7.4 | Nuclear and Cytoplasm |
| LINC00158 | Nuclear and Cytoplasm |
| LINC00200 | Nuclear and Cytoplasm |
| LINC00221 | Nuclear and Cytoplasm |
| LINC00355 | Nuclear and Cytoplasm |
| LINC00461 | Nuclear and Cytoplasm |
| LINC00472 | Nuclear and Cytoplasm |
| LINC00528 | Nuclear and Cytoplasm |
| LINC00589 | Nuclear and Cytoplasm |
| LINC00632 | Nuclear and Cytoplasm |
| LINC00652 | Nuclear and Cytoplasm |
| LINC00664 | Nuclear and Cytoplasm |
| LINC00704 | Nuclear and Cytoplasm |
| LINC00862 | Nuclear and Cytoplasm |
| LINC00864 | Nuclear and Cytoplasm |
| LINC00885 | Nuclear and Cytoplasm |
| LINC00887 | Nuclear and Cytoplasm |
| LINC00896 | Nuclear and Cytoplasm |
| LINC00922 | Nuclear and Cytoplasm |
| LINC00982 | Nuclear and Cytoplasm |
| LINC01012 | Nuclear and Cytoplasm |
| LINC01021 | Nuclear and Cytoplasm |
| LINC01030 | Nuclear and Cytoplasm |
| LINC01094 | Nuclear and Cytoplasm |
| LINC01146 | Nuclear and Cytoplasm |
| LINC01152 | Nuclear and Cytoplasm |
| LINC01192 | Nuclear and Cytoplasm |
| LINC01234 | Nuclear and Cytoplasm |
| LINC01260 | Nuclear and Cytoplasm |
| LINC01317 | Nuclear and Cytoplasm |
| LINC01411 | Nuclear and Cytoplasm |
| LINC01426 | Nuclear and Cytoplasm |
| LINC01447 | Nuclear and Cytoplasm |
| LINC01480 | Nuclear and Cytoplasm |
| LINC01531 | Nuclear and Cytoplasm |
| LINC01543 | Nuclear and Cytoplasm |
| LINC01583 | Nuclear and Cytoplasm |
| LINC01589 | Nuclear and Cytoplasm |
| LINC01606 | Nuclear and Cytoplasm |
| LL22NC03-63E9.3 | Nuclear and Cytoplasm |
| LL22NC03-N14H11.1 | Nuclear and Cytoplasm |
| LUCAT1 | Nuclear and Cytoplasm |
| LY86-AS1 | Nuclear and Cytoplasm |
| LYPLAL1-AS1 | Nuclear and Cytoplasm |
| MCF2L-AS1 | Nuclear and Cytoplasm |
| MESTIT1 | Nuclear and Cytoplasm |
| MGC39584 | Nuclear and Cytoplasm |
| MIAT | Nuclear and Cytoplasm |
| MIR155HG | Nuclear and Cytoplasm |
| MIR210HG | Nuclear and Cytoplasm |
| MMP25-AS1 | Nuclear and Cytoplasm |
| MNX1-AS1 | Nuclear and Cytoplasm |
| NPSR1-AS1 | Nuclear and Cytoplasm |
| PCAT14 | Nuclear and Cytoplasm |
| PCED1B-AS1 | Nuclear and Cytoplasm |
| PLS3-AS1 | Nuclear and Cytoplasm |
| PP7080 | Nuclear and Cytoplasm |
| PRR7-AS1 | Nuclear and Cytoplasm |
| RMRP | Nuclear and Cytoplasm |
| RNU12 | Nuclear and Cytoplasm |
| RP1-101G11.3 | Nuclear and Cytoplasm |
| RP11-102G14.1 | Nuclear and Cytoplasm |
| RP11-1086F11.1 | Nuclear and Cytoplasm |
| RP11-10J5.1 | Nuclear and Cytoplasm |
| RP11-112L6.3 | Nuclear and Cytoplasm |
| RP11-115D19.1 | Nuclear and Cytoplasm |
| RP11-121A8.1 | Nuclear and Cytoplasm |
| RP11-123K19.1 | Nuclear and Cytoplasm |
| RP11-138I1.2 | Nuclear and Cytoplasm |
| RP11-145A3.1 | Nuclear and Cytoplasm |
| RP11-147L13.2 | Nuclear and Cytoplasm |
| RP11-155G14.6 | Nuclear and Cytoplasm |
| RP11-167N4.2 | Nuclear and Cytoplasm |
| RP11-16C1.2 | Nuclear and Cytoplasm |
| RP11-178L8.5 | Nuclear and Cytoplasm |
| RP1-118J21.25 | Nuclear and Cytoplasm |
| RP11-1C1.4 | Nuclear and Cytoplasm |
| RP11-206M11.7 | Nuclear and Cytoplasm |
| RP11-211G23.2 | Nuclear and Cytoplasm |
| RP11-21K12.3 | Nuclear and Cytoplasm |
| RP11-227G15.9 | Nuclear and Cytoplasm |
| RP11-22C11.2 | Nuclear and Cytoplasm |
| RP11-231E4.5 | Nuclear and Cytoplasm |
| RP11-244M2.1 | Nuclear and Cytoplasm |
| RP11-252K23.1 | Nuclear and Cytoplasm |
| RP11-259N19.1 | Nuclear and Cytoplasm |
| RP11-264B17.3 | Nuclear and Cytoplasm |
| RP11-267C16.1 | Nuclear and Cytoplasm |
| RP11-284F21.10 | Nuclear and Cytoplasm |
| RP11-284F21.7 | Nuclear and Cytoplasm |
| RP11-284F21.9 | Nuclear and Cytoplasm |
| RP11-290F5.2 | Nuclear and Cytoplasm |
| RP11-295M3.4 | Nuclear and Cytoplasm |
| RP11-299G20.2 | Nuclear and Cytoplasm |
| RP11-307O13.1 | Nuclear and Cytoplasm |
| RP11-314N14.1 | Nuclear and Cytoplasm |
| RP11-31F19.1 | Nuclear and Cytoplasm |
| RP11-321G12.1 | Nuclear and Cytoplasm |
| RP11-326C3.14 | Nuclear and Cytoplasm |
| RP11-327F22.2 | Nuclear and Cytoplasm |
| RP11-332J15.4 | Nuclear and Cytoplasm |
| RP11-341G23.4 | Nuclear and Cytoplasm |
| RP11-348J24.2 | Nuclear and Cytoplasm |
| RP11-352D3.2 | Nuclear and Cytoplasm |
| RP11-352G9.1 | Nuclear and Cytoplasm |
| RP11-356I2.4 | Nuclear and Cytoplasm |
| RP11-356J5.12 | Nuclear and Cytoplasm |
| RP11-359E10.1 | Nuclear and Cytoplasm |
| RP11-359G22.2 | Nuclear and Cytoplasm |
| RP11-360O19.4 | Nuclear and Cytoplasm |
| RP11-362F19.1 | Nuclear and Cytoplasm |
| RP11-367G18.1 | Nuclear and Cytoplasm |
| RP11-379F12.3 | Nuclear and Cytoplasm |
| RP11-401P9.4 | Nuclear and Cytoplasm |
| RP11-404F10.2 | Nuclear and Cytoplasm |
| RP1-140K8.5 | Nuclear and Cytoplasm |
| RP11-440G9.1 | Nuclear and Cytoplasm |
| RP11-445F12.1 | Nuclear and Cytoplasm |
| RP11-44F14.2 | Nuclear and Cytoplasm |
| RP11-44F14.8 | Nuclear and Cytoplasm |
| RP11-44N11.2 | Nuclear and Cytoplasm |
| RP11-456H18.2 | Nuclear and Cytoplasm |
| RP11-464D20.6 | Nuclear and Cytoplasm |
| RP11-465L10.10 | Nuclear and Cytoplasm |
| RP11-469H8.6 | Nuclear and Cytoplasm |
| RP11-476K15.1 | Nuclear and Cytoplasm |
| RP11-480A16.1 | Nuclear and Cytoplasm |
| RP11-482D24.3 | Nuclear and Cytoplasm |
| RP11-496D24.2 | Nuclear and Cytoplasm |
| RP11-496I9.1 | Nuclear and Cytoplasm |
| RP11-497H17.1 | Nuclear and Cytoplasm |
| RP11-513G11.4 | Nuclear and Cytoplasm |
| RP11-528A4.2 | Nuclear and Cytoplasm |
| RP11-531A24.3 | Nuclear and Cytoplasm |
| RP11-536K7.3 | Nuclear and Cytoplasm |
| RP11-552M14.1 | Nuclear and Cytoplasm |
| RP11-554A11.9 | Nuclear and Cytoplasm |
| RP11-567G11.1 | Nuclear and Cytoplasm |
| RP11-572O17.1 | Nuclear and Cytoplasm |
| RP11-579D7.4 | Nuclear and Cytoplasm |
| RP11-587P21.2 | Nuclear and Cytoplasm |
| RP11-617F23.2 | Nuclear and Cytoplasm |
| RP11-61L19.2 | Nuclear and Cytoplasm |
| RP11-674N23.4 | Nuclear and Cytoplasm |
| RP1-167A14.2 | Nuclear and Cytoplasm |
| RP11-689K5.3 | Nuclear and Cytoplasm |
| RP1-168L15.5 | Nuclear and Cytoplasm |
| RP11-697N18.4 | Nuclear and Cytoplasm |
| RP11-713C5.1 | Nuclear and Cytoplasm |
| RP11-776H12.1 | Nuclear and Cytoplasm |
| RP11-785D18.3 | Nuclear and Cytoplasm |
| RP11-798M19.6 | Nuclear and Cytoplasm |
| RP11-81H14.1 | Nuclear and Cytoplasm |
| RP11-81H14.2 | Nuclear and Cytoplasm |
| RP11-834C11.4 | Nuclear and Cytoplasm |
| RP11-883A18.3 | Nuclear and Cytoplasm |
| RP11-893F2.6 | Nuclear and Cytoplasm |
| RP11-89K21.1 | Nuclear and Cytoplasm |
| RP11-8L2.1 | Nuclear and Cytoplasm |
| RP11-91J3.3 | Nuclear and Cytoplasm |
| RP11-91P24.6 | Nuclear and Cytoplasm |
| RP11-92A5.2 | Nuclear and Cytoplasm |
| RP11-932O9.10 | Nuclear and Cytoplasm |
| RP11-94H18.1 | Nuclear and Cytoplasm |
| RP11-982M15.8 | Nuclear and Cytoplasm |
| RP1-206D15.6 | Nuclear and Cytoplasm |
| RP1-224A6.9 | Nuclear and Cytoplasm |
| RP1-244F24.1 | Nuclear and Cytoplasm |
| RP1-29C18.8 | Nuclear and Cytoplasm |
| RP13-463N16.6 | Nuclear and Cytoplasm |
| RP13-616I3.1 | Nuclear and Cytoplasm |
| RP13-650J16.1 | Nuclear and Cytoplasm |
| RP1-78O14.1 | Nuclear and Cytoplasm |
| RP3-333A15.2 | Nuclear and Cytoplasm |
| RP3-455J7.4 | Nuclear and Cytoplasm |
| RP3-460G2.2 | Nuclear and Cytoplasm |
| RP3-466P17.1 | Nuclear and Cytoplasm |
| RP4-568C11.4 | Nuclear and Cytoplasm |
| RP4-665J23.1 | Nuclear and Cytoplasm |
| RP4-737E23.2 | Nuclear and Cytoplasm |
| RP4-794I6.4 | Nuclear and Cytoplasm |
| RP5-1028K7.2 | Nuclear and Cytoplasm |
| RP5-1070A16.1 | Nuclear and Cytoplasm |
| RP5-1091N2.9 | Nuclear and Cytoplasm |
| RP5-1112D6.4 | Nuclear and Cytoplasm |
| RP5-1120P11.1 | Nuclear and Cytoplasm |
| RP5-1171I10.5 | Nuclear and Cytoplasm |
| RP5-1172A22.1 | Nuclear and Cytoplasm |
| RP5-1185I7.1 | Nuclear and Cytoplasm |
| RP5-875O13.1 | Nuclear and Cytoplasm |
| RP5-881L22.5 | Nuclear and Cytoplasm |
| RP5-884M6.1 | Nuclear and Cytoplasm |
| RP5-902P8.12 | Nuclear and Cytoplasm |
| RP5-940J5.3 | Nuclear and Cytoplasm |
| RP5-984P4.6 | Nuclear and Cytoplasm |
| RP6-91H8.3 | Nuclear and Cytoplasm |
| SEMA3B-AS1 | Nuclear and Cytoplasm |
| SEMA6A-AS1 | Nuclear and Cytoplasm |
| SFTA1P | Nuclear and Cytoplasm |
| SH3PXD2A-AS1 | Nuclear and Cytoplasm |
| SLC16A1-AS1 | Nuclear and Cytoplasm |
| SLC25A5-AS1 | Nuclear and Cytoplasm |
| SLC7A11-AS1 | Nuclear and Cytoplasm |
| SLX1A-SULT1A3 | Nuclear and Cytoplasm |
| SNAP25-AS1 | Nuclear and Cytoplasm |
| SNHG12 | Nuclear and Cytoplasm |
| SOX21-AS1 | Nuclear and Cytoplasm |
| SPATA13 | Nuclear and Cytoplasm |
| SPRY4-IT1 | Nuclear and Cytoplasm |
| STEAP3-AS1 | Nuclear and Cytoplasm |
| TINCR | Nuclear and Cytoplasm |
| TPTEP1 | Nuclear and Cytoplasm |
| TRG-AS1 | Nuclear and Cytoplasm |
| TRPM2-AS | Nuclear and Cytoplasm |
| TSSC1-IT1 | Nuclear and Cytoplasm |
| TTLL7-IT1 | Nuclear and Cytoplasm |
| U52111.14 | Nuclear and Cytoplasm |
| UCA1 | Nuclear and Cytoplasm |
| UG0898H09 | Nuclear and Cytoplasm |
| UNQ6494 | Nuclear and Cytoplasm |
| VWA8-AS1 | Nuclear and Cytoplasm |
| WI2-87327B8.2 | Nuclear and Cytoplasm |
| WT1-AS | Nuclear and Cytoplasm |
| AC003009.1 | Cytoplasm |
| AC010091.1 | Cytoplasm |
| AC011239.1 | Cytoplasm |
| AC017002.1 | Cytoplasm |
| AC019117.1 | Cytoplasm |
| AC027119.1 | Cytoplasm |
| AC067959.1 | Cytoplasm |
| AC092580.4 | Cytoplasm |
| AC092667.2 | Cytoplasm |
| AC098828.2 | Cytoplasm |
| AC099684.1 | Cytoplasm |
| AC104699.1 | Cytoplasm |
| AC108676.1 | Cytoplasm |
| AC114877.3 | Cytoplasm |
| AC133785.1 | Cytoplasm |
| AC142119.1 | Cytoplasm |
| AF121898.3 | Cytoplasm |
| AP000345.1 | Cytoplasm |
| AP000439.3 | Cytoplasm |
| AP004372.1 | Cytoplasm |
| C12orf77 | Cytoplasm |
| C15orf54 | Cytoplasm |
| CH17-360D5.2 | Cytoplasm |
| CTA-384D8.34 | Cytoplasm |
| CTA-384D8.35 | Cytoplasm |
| CTA-398F10.2 | Cytoplasm |
| CTB-127C13.1 | Cytoplasm |
| CTB-33O18.1 | Cytoplasm |
| CTC-241F20.4 | Cytoplasm |
| CTD-2207A17.1 | Cytoplasm |
| CTD-2263F21.1 | Cytoplasm |
| CTD-2309O5.3 | Cytoplasm |
| CTD-2501M5.1 | Cytoplasm |
| CTD-2562J17.4 | Cytoplasm |
| CTD-2587H19.3 | Cytoplasm |
| CTD-2621I17.6 | Cytoplasm |
| CTD-3187F8.14 | Cytoplasm |
| DAPK1-IT1 | Cytoplasm |
| EGFR-AS1 | Cytoplasm |
| ELOVL2-AS1 | Cytoplasm |
| FGF10-AS1 | Cytoplasm |
| HNF4A-AS1 | Cytoplasm |
| HOXB-AS3 | Cytoplasm |
| IFT74-AS1 | Cytoplasm |
| KCCAT211 | Cytoplasm |
| KCNH1-IT1 | Cytoplasm |
| LAMA5-AS1 | Cytoplasm |
| LINC00162 | Cytoplasm |
| LINC00237 | Cytoplasm |
| LINC00426 | Cytoplasm |
| LINC00443 | Cytoplasm |
| LINC00460 | Cytoplasm |
| LINC00462 | Cytoplasm |
| LINC00645 | Cytoplasm |
| LINC00678 | Cytoplasm |
| LINC00707 | Cytoplasm |
| LINC00838 | Cytoplasm |
| LINC01018 | Cytoplasm |
| LINC01055 | Cytoplasm |
| LINC01224 | Cytoplasm |
| LINC01235 | Cytoplasm |
| LINC01265 | Cytoplasm |
| LINC01281 | Cytoplasm |
| LINC01314 | Cytoplasm |
| LINC01351 | Cytoplasm |
| LINC01358 | Cytoplasm |
| LINC01405 | Cytoplasm |
| LINC01468 | Cytoplasm |
| LINC01546 | Cytoplasm |
| LINC01551 | Cytoplasm |
| LINC01559 | Cytoplasm |
| LINC01587 | Cytoplasm |
| LINC01615 | Cytoplasm |
| LMO7-AS1 | Cytoplasm |
| MNX1-AS2 | Cytoplasm |
| MYCNOS | Cytoplasm |
| PAQR9-AS1 | Cytoplasm |
| PCSK6-AS1 | Cytoplasm |
| PHEX-AS1 | Cytoplasm |
| PROX1-AS1 | Cytoplasm |
| PSORS1C3 | Cytoplasm |
| RC3H1-IT1 | Cytoplasm |
| RP1-102K2.6 | Cytoplasm |
| RP11-100E13.1 | Cytoplasm |
| RP11-1018N14.5 | Cytoplasm |
| RP11-10O22.1 | Cytoplasm |
| RP11-114M1.2 | Cytoplasm |
| RP11-115D19.2 | Cytoplasm |
| RP11-115H13.1 | Cytoplasm |
| RP11-115H15.2 | Cytoplasm |
| RP11-120K24.3 | Cytoplasm |
| RP11-128L5.1 | Cytoplasm |
| RP11-138H10.2 | Cytoplasm |
| RP11-141O11.2 | Cytoplasm |
| RP11-142A23.1 | Cytoplasm |
| RP11-145M4.2 | Cytoplasm |
| RP11-161D15.1 | Cytoplasm |
| RP11-161D15.3 | Cytoplasm |
| RP11-16E23.3 | Cytoplasm |
| RP11-1C1.6 | Cytoplasm |
| RP11-222K16.2 | Cytoplasm |
| RP11-239L20.6 | Cytoplasm |
| RP11-247A12.7 | Cytoplasm |
| RP11-255G12.3 | Cytoplasm |
| RP11-255M2.1 | Cytoplasm |
| RP11-276E17.2 | Cytoplasm |
| RP11-283G6.4 | Cytoplasm |
| RP11-283G6.5 | Cytoplasm |
| RP11-291B21.2 | Cytoplasm |
| RP11-297C4.2 | Cytoplasm |
| RP11-297L17.2 | Cytoplasm |
| RP11-2E17.2 | Cytoplasm |
| RP11-317M11.1 | Cytoplasm |
| RP11-339D23.1 | Cytoplasm |
| RP11-354K4.2 | Cytoplasm |
| RP11-356I2.1 | Cytoplasm |
| RP11-363J20.1 | Cytoplasm |
| RP11-384C4.6 | Cytoplasm |
| RP11-392O17.1 | Cytoplasm |
| RP11-395B7.4 | Cytoplasm |
| RP11-404P21.3 | Cytoplasm |
| RP11-405M12.3 | Cytoplasm |
| RP11-429E11.2 | Cytoplasm |
| RP11-429E11.3 | Cytoplasm |
| RP11-44M6.1 | Cytoplasm |
| RP11-451G4.2 | Cytoplasm |
| RP11-470M17.2 | Cytoplasm |
| RP11-472G21.2 | Cytoplasm |
| RP11-480D4.6 | Cytoplasm |
| RP11-482D24.2 | Cytoplasm |
| RP11-497G19.7 | Cytoplasm |
| RP11-498B4.5 | Cytoplasm |
| RP11-524H19.2 | Cytoplasm |
| RP11-525K10.3 | Cytoplasm |
| RP11-536I6.2 | Cytoplasm |
| RP11-538D16.3 | Cytoplasm |
| RP11-538I12.3 | Cytoplasm |
| RP11-547D24.1 | Cytoplasm |
| RP11-553L6.2 | Cytoplasm |
| RP11-554I8.2 | Cytoplasm |
| RP11-629G13.1 | Cytoplasm |
| RP11-636O21.1 | Cytoplasm |
| RP11-643M14.1 | Cytoplasm |
| RP11-662G23.1 | Cytoplasm |
| RP11-663N22.1 | Cytoplasm |
| RP11-680A11.5 | Cytoplasm |
| RP11-706C16.7 | Cytoplasm |
| RP11-728F11.4 | Cytoplasm |
| RP11-73G16.1 | Cytoplasm |
| RP11-758N13.1 | Cytoplasm |
| RP11-818F20.5 | Cytoplasm |
| RP11-861L17.4 | Cytoplasm |
| RP11-865I6.2 | Cytoplasm |
| RP11-89M20.2 | Cytoplasm |
| RP11-903H12.3 | Cytoplasm |
| RP11-90P13.1 | Cytoplasm |
| RP11-999E24.3 | Cytoplasm |
| RP1-232P20.1 | Cytoplasm |
| RP13-631K18.3 | Cytoplasm |
| RP13-895J2.6 | Cytoplasm |
| RP3-323N1.2 | Cytoplasm |
| RP3-380B4.1 | Cytoplasm |
| RP3-477O4.14 | Cytoplasm |
| RP3-522D1.1 | Cytoplasm |
| RP3-523K23.2 | Cytoplasm |
| RP4-541C22.5 | Cytoplasm |
| RP4-555D20.4 | Cytoplasm |
| RP4-802A10.1 | Cytoplasm |
| RP5-1092A11.5 | Cytoplasm |
| RP5-867C24.4 | Cytoplasm |
| RP5-952N6.1 | Cytoplasm |
| RP6-191P20.4 | Cytoplasm |
| RP6-24A23.7 | Cytoplasm |
| RP6-91H8.5 | Cytoplasm |
| SERPINB9P1 | Cytoplasm |
| TMEM108-AS1 | Cytoplasm |
| USP30-AS1 | Cytoplasm |
| XXbac-BPG27H4.8 | Cytoplasm |
| XXbac-B461K10.4 | Cytoplasm |
| AC000036.4 | No data |
| AC000067.1 | No data |
| AC002044.4 | No data |
| AC002306.1 | No data |
| AC002331.1 | No data |
| AC002401.1 | No data |
| AC003092.2 | No data |
| AC003984.1 | No data |
| AC004066.3 | No data |
| AC004448.5 | No data |
| AC004603.4 | No data |
| AC004840.8 | No data |
| AC004862.6 | No data |
| AC004901.1 | No data |
| AC004906.3 | No data |
| AC005152.3 | No data |
| AC005281.2 | No data |
| AC005301.8 | No data |
| AC005387.2 | No data |
| AC005387.3 | No data |
| AC005522.7 | No data |
| AC005550.3 | No data |
| AC005616.2 | No data |
| AC005757.6 | No data |
| AC005954.3 | No data |
| AC006227.1 | No data |
| AC006272.2 | No data |
| AC006369.2 | No data |
| AC006458.3 | No data |
| AC006960.7 | No data |
| AC007040.8 | No data |
| AC007050.17 | No data |
| AC007078.4 | No data |
| AC007193.6 | No data |
| AC007326.10 | No data |
| AC007362.3 | No data |
| AC007461.2 | No data |
| AC007750.5 | No data |
| AC007879.3 | No data |
| AC008060.7 | No data |
| AC008067.2 | No data |
| AC008268.1 | No data |
| AC008271.1 | No data |
| AC008991.1 | No data |
| AC009305.1 | No data |
| AC009478.1 | No data |
| AC009495.3 | No data |
| AC009518.4 | No data |
| AC010884.1 | No data |
| AC010894.5 | No data |
| AC011516.2 | No data |
| AC011524.1 | No data |
| AC011524.2 | No data |
| AC011524.3 | No data |
| AC011625.1 | No data |
| AC011752.1 | No data |
| AC011899.9 | No data |
| AC012307.2 | No data |
| AC012462.2 | No data |
| AC012593.1 | No data |
| AC012613.2 | No data |
| AC012668.2 | No data |
| AC015849.2 | No data |
| AC015933.2 | No data |
| AC015977.6 | No data |
| AC016700.2 | No data |
| AC017060.1 | No data |
| AC017104.2 | No data |
| AC018685.1 | No data |
| AC019055.1 | No data |
| AC019064.1 | No data |
| AC019117.2 | No data |
| AC020956.3 | No data |
| AC023669.1 | No data |
| AC024084.1 | No data |
| AC024592.9 | No data |
| AC025811.3 | No data |
| AC026167.1 | No data |
| AC027601.1 | No data |
| AC055764.1 | No data |
| AC064834.3 | No data |
| AC067945.4 | No data |
| AC068196.1 | No data |
| AC068492.1 | No data |
| AC068535.3 | No data |
| AC068858.1 | No data |
| AC069155.1 | No data |
| AC069394.1 | No data |
| AC073115.6 | No data |
| AC073115.7 | No data |
| AC073218.3 | No data |
| AC073257.1 | No data |
| AC074366.3 | No data |
| AC078842.3 | No data |
| AC079154.1 | No data |
| AC079612.1 | No data |
| AC079767.4 | No data |
| AC083867.4 | No data |
| AC084117.3 | No data |
| AC090505.1 | No data |
| AC091153.4 | No data |
| AC091177.1 | No data |
| AC091814.3 | No data |
| AC092295.4 | No data |
| AC092484.1 | No data |
| AC092635.1 | No data |
| AC092657.2 | No data |
| AC093063.3 | No data |
| AC093326.1 | No data |
| AC093627.11 | No data |
| AC093642.4 | No data |
| AC093642.6 | No data |
| AC093802.1 | No data |
| AC096558.1 | No data |
| AC096574.5 | No data |
| AC096669.1 | No data |
| AC096669.3 | No data |
| AC097495.2 | No data |
| AC097499.1 | No data |
| AC097713.3 | No data |
| AC097713.4 | No data |
| AC099552.3 | No data |
| AC103563.8 | No data |
| AC103563.9 | No data |
| AC104532.3 | No data |
| AC104777.2 | No data |
| AC105398.3 | No data |
| AC106873.4 | No data |
| AC107057.1 | No data |
| AC107218.3 | No data |
| AC108463.1 | No data |
| AC109826.1 | No data |
| AC112715.2 | No data |
| AC114730.7 | No data |
| AC114803.3 | No data |
| AC114812.8 | No data |
| AC116366.5 | No data |
| AC124861.1 | No data |
| AC124944.3 | No data |
| AC124944.5 | No data |
| AC124997.1 | No data |
| AC128709.3 | No data |
| AC130469.2 | No data |
| AC131056.5 | No data |
| AC141930.2 | No data |
| AC145110.1 | No data |
| AE000662.93 | No data |
| AF064858.10 | No data |
| AF064858.11 | No data |
| AF067845.1 | No data |
| AKT3-IT1 | No data |
| AL592528.1 | No data |
| ANKRD33B-AS1 | No data |
| ANKRD62P1-PARP4P3 | No data |
| AOAH-IT1 | No data |
| AP000146.2 | No data |
| AP000289.6 | No data |
| AP000344.3 | No data |
| AP000439.1 | No data |
| AP000459.7 | No data |
| AP000472.3 | No data |
| AP000476.1 | No data |
| AP000697.6 | No data |
| AP000797.3 | No data |
| AP000797.4 | No data |
| AP000997.1 | No data |
| AP000997.2 | No data |
| AP001055.6 | No data |
| AP001063.1 | No data |
| AP001257.1 | No data |
| AP001604.3 | No data |
| AP002954.4 | No data |
| AP003025.2 | No data |
| ARAP1-AS2 | No data |
| ARHGAP26-AS1 | No data |
| ARHGAP26-IT1 | No data |
| ATP11A-AS1 | No data |
| BARX1-AS1 | No data |
| BMPR1B-AS1 | No data |
| BPESC1 | No data |
| C1orf147 | No data |
| C3orf67-AS1 | No data |
| CARS-AS1 | No data |
| CASC6 | No data |
| CCDC39-AS1 | No data |
| CDKN2A-AS1 | No data |
| CDRT7 | No data |
| CELF2-AS1 | No data |
| CERS3-AS1 | No data |
| CHL1-AS2 | No data |
| COL18A1-AS1 | No data |
| CSNK1G2-AS1 | No data |
| CTA-125H2.2 | No data |
| CTA-221G9.11 | No data |
| CTA-243E7.3 | No data |
| CTA-243E7.4 | No data |
| CTA-276F8.2 | No data |
| CTA-305I2.1 | No data |
| CTA-339C12.1 | No data |
| CTA-363E6.1 | No data |
| CTA-722E9.1 | No data |
| CTA-797E19.1 | No data |
| CTA-833B7.2 | No data |
| CTB-102L5.8 | No data |
| CTB-107G13.1 | No data |
| CTB-118P15.2 | No data |
| CTB-140J7.2 | No data |
| CTB-174D11.2 | No data |
| CTB-178M22.1 | No data |
| CTB-31N19.5 | No data |
| CTB-31O20.8 | No data |
| CTB-33O18.2 | No data |
| CTB-35F21.1 | No data |
| CTB-35F21.2 | No data |
| CTB-35F21.3 | No data |
| CTB-37A13.1 | No data |
| CTB-39G8.3 | No data |
| CTB-43E15.1 | No data |
| CTB-49A3.4 | No data |
| CTB-50L17.5 | No data |
| CTB-61M7.1 | No data |
| CTB-70G10.1 | No data |
| CTB-73N10.1 | No data |
| CTB-78F1.1 | No data |
| CTB-79E8.2 | No data |
| CTB-91J4.1 | No data |
| CTB-92J24.3 | No data |
| CTC-268N12.3 | No data |
| CTC-327F10.4 | No data |
| CTC-327F10.5 | No data |
| CTC-329D1.3 | No data |
| CTC-340D7.1 | No data |
| CTC-340I23.2 | No data |
| CTC-360G5.6 | No data |
| CTC-379B2.4 | No data |
| CTC-391G2.1 | No data |
| CTC-435M10.10 | No data |
| CTC-453G23.5 | No data |
| CTC-458G6.2 | No data |
| CTC-459M5.1 | No data |
| CTC-472C24.1 | No data |
| CTC-484P3.3 | No data |
| CTC-498J12.1 | No data |
| CTC-503J8.4 | No data |
| CTC-529L17.2 | No data |
| CTC-535M15.2 | No data |
| CTC-537E7.2 | No data |
| CTC-542B22.2 | No data |
| CTC-575I10.1 | No data |
| CTC-806A22.1 | No data |
| CTD-2004A9.1 | No data |
| CTD-2007H18.1 | No data |
| CTD-2010I22.2 | No data |
| CTD-2013N17.4 | No data |
| CTD-2015A6.2 | No data |
| CTD-2016O11.1 | No data |
| CTD-2023M8.1 | No data |
| CTD-2024I7.13 | No data |
| CTD-2083E4.7 | No data |
| CTD-2085J24.3 | No data |
| CTD-2130F23.2 | No data |
| CTD-2130O13.1 | No data |
| CTD-2168K21.1 | No data |
| CTD-2171N6.1 | No data |
| CTD-2193G5.1 | No data |
| CTD-2195B23.3 | No data |
| CTD-2227I18.1 | No data |
| CTD-2236F14.1 | No data |
| CTD-2240J17.4 | No data |
| CTD-2247C11.5 | No data |
| CTD-2252P21.1 | No data |
| CTD-2278I10.1 | No data |
| CTD-2282P23.2 | No data |
| CTD-2291D10.3 | No data |
| CTD-2297D10.1 | No data |
| CTD-2313F11.1 | No data |
| CTD-2315E11.1 | No data |
| CTD-2330K9.2 | No data |
| CTD-2363C16.1 | No data |
| CTD-2373N4.5 | No data |
| CTD-2376I20.1 | No data |
| CTD-2527I21.14 | No data |
| CTD-2527I21.9 | No data |
| CTD-2528A14.5 | No data |
| CTD-2529P6.3 | No data |
| CTD-2532K18.2 | No data |
| CTD-2534I21.9 | No data |
| CTD-2537I9.12 | No data |
| CTD-2553C6.1 | No data |
| CTD-2553L13.4 | No data |
| CTD-2560E9.5 | No data |
| CTD-2562J17.2 | No data |
| CTD-2568A17.1 | No data |
| CTD-2571L23.8 | No data |
| CTD-2583P5.1 | No data |
| CTD-2583P5.3 | No data |
| CTD-3032J10.2 | No data |
| CTD-3032J10.3 | No data |
| CTD-3032J10.4 | No data |
| CTD-3118D11.3 | No data |
| CTD-3162L10.3 | No data |
| CTD-3195I5.3 | No data |
| CTD-3195I5.4 | No data |
| DLGAP2-AS1 | No data |
| DOCK4-AS1 | No data |
| DPP9-AS1 | No data |
| EGLN3-AS1 | No data |
| ELDR | No data |
| ENOX1-AS2 | No data |
| FAM215A | No data |
| FAM3D-AS1 | No data |
| FLJ22763 | No data |
| FOXC2-AS1 | No data |
| FOXCUT | No data |
| FOXN3-AS2 | No data |
| GK-IT1 | No data |
| GM140 | No data |
| GPC6-AS1 | No data |
| GPC6-AS2 | No data |
| GRID1-AS1 | No data |
| GRPEL2-AS1 | No data |
| GS1-174L6.4 | No data |
| GS1-278J22.1 | No data |
| GS1-278J22.2 | No data |
| GS1-279B7.1 | No data |
| HLA-DQB1-AS1 | No data |
| HM13-IT1 | No data |
| HMBOX1-IT1 | No data |
| IL20RB-AS1 | No data |
| ITCH-IT1 | No data |
| KB-1043D8.8 | No data |
| KB-1460A1.1 | No data |
| KB-1460A1.2 | No data |
| KB-173C10.1 | No data |
| KCNIP4-IT1 | No data |
| KCNMA1-AS1 | No data |
| KCNMA1-AS3 | No data |
| KIRREL3-AS1 | No data |
| L3MBTL4-AS1 | No data |
| LA16c-321D4.2 | No data |
| LA16c-325D7.1 | No data |
| LA16c-329F2.1 | No data |
| LA16c-444G7.1 | No data |
| LATS2-AS1 | No data |
| LIMS1-AS1 | No data |
| LINC00051 | No data |
| LINC00102 | No data |
| LINC00111 | No data |
| LINC00161 | No data |
| LINC00264 | No data |
| LINC00272 | No data |
| LINC00303 | No data |
| LINC00307 | No data |
| LINC00314 | No data |
| LINC00343 | No data |
| LINC00379 | No data |
| LINC00380 | No data |
| LINC00384 | No data |
| LINC00397 | No data |
| LINC00410 | No data |
| LINC00427 | No data |
| LINC00437 | No data |
| LINC00454 | No data |
| LINC00477 | No data |
| LINC00492 | No data |
| LINC00507 | No data |
| LINC00524 | No data |
| LINC00544 | No data |
| LINC00551 | No data |
| LINC00593 | No data |
| LINC00603 | No data |
| LINC00626 | No data |
| LINC00676 | No data |
| LINC00705 | No data |
| LINC00710 | No data |
| LINC00836 | No data |
| LINC00845 | No data |
| LINC00861 | No data |
| LINC00868 | No data |
| LINC00906 | No data |
| LINC00919 | No data |
| LINC00928 | No data |
| LINC00942 | No data |
| LINC00943 | No data |
| LINC00944 | No data |
| LINC00955 | No data |
| LINC00971 | No data |
| LINC01020 | No data |
| LINC01031 | No data |
| LINC01048 | No data |
| LINC01067 | No data |
| LINC01077 | No data |
| LINC01141 | No data |
| LINC01150 | No data |
| LINC01159 | No data |
| LINC01207 | No data |
| LINC01227 | No data |
| LINC01255 | No data |
| LINC01280 | No data |
| LINC01304 | No data |
| LINC01312 | No data |
| LINC01330 | No data |
| LINC01378 | No data |
| LINC01428 | No data |
| LINC01440 | No data |
| LINC01479 | No data |
| LINC01485 | No data |
| LINC01492 | No data |
| LINC01501 | No data |
| LINC01510 | No data |
| LINC01513 | No data |
| LINC01517 | No data |
| LINC01522 | No data |
| LINC01523 | No data |
| LINC01538 | No data |
| LINC01571 | No data |
| LINC01611 | No data |
| LINC01612 | No data |
| LINC01616 | No data |
| LINC01621 | No data |
| LINCMD1 | No data |
| LRP1-AS | No data |
| LZTS1-AS1 | No data |
| MLIP-AS1 | No data |
| MYCNUT | No data |
| MYO16-AS1 | No data |
| NAV2-AS3 | No data |
| NLGN1-AS1 | No data |
| NRG3-AS1 | No data |
| NUCB1-AS1 | No data |
| OR7E47P | No data |
| OSTM1-AS1 | No data |
| PCAT2 | No data |
| PCAT29 | No data |
| PCGEM1 | No data |
| PHACTR2-AS1 | No data |
| PLA2G4C-AS1 | No data |
| PTCSC3 | No data |
| PTPRJ-AS1 | No data |
| RERG-IT1 | No data |
| RNF216-IT1 | No data |
| RP1-101D8.1 | No data |
| RP1-105O18.1 | No data |
| RP1-10C16.1 | No data |
| RP11-1000B6.2 | No data |
| RP11-1007G5.2 | No data |
| RP11-100G15.10 | No data |
| RP11-1026M7.2 | No data |
| RP11-1029J19.2 | No data |
| RP11-1030E3.1 | No data |
| RP11-103C16.2 | No data |
| RP11-103H7.3 | No data |
| RP11-1070N10.6 | No data |
| RP11-107I14.2 | No data |
| RP11-107I14.4 | No data |
| RP11-1084E5.1 | No data |
| RP11-108E14.1 | No data |
| RP11-108P20.2 | No data |
| RP11-109P6.2 | No data |
| RP11-10A14.6 | No data |
| RP11-10C8.2 | No data |
| RP11-1105G2.4 | No data |
| RP11-1137G4.3 | No data |
| RP11-113I22.1 | No data |
| RP11-113O24.3 | No data |
| RP11-1140I5.1 | No data |
| RP11-1149M10.2 | No data |
| RP11-114H24.7 | No data |
| RP11-1151B14.2 | No data |
| RP11-1151B14.3 | No data |
| RP11-115C10.1 | No data |
| RP11-115N4.1 | No data |
| RP11-117L5.4 | No data |
| RP11-118M9.3 | No data |
| RP11-1191J2.4 | No data |
| RP1-111C20.3 | No data |
| RP11-120I21.2 | No data |
| RP11-121A14.2 | No data |
| RP11-121A14.3 | No data |
| RP11-122C21.1 | No data |
| RP11-124N19.3 | No data |
| RP11-1250I15.3 | No data |
| RP11-1259L22.1 | No data |
| RP11-1260E13.1 | No data |
| RP11-1263C18.1 | No data |
| RP11-126O1.2 | No data |
| RP11-126O1.4 | No data |
| RP11-127I20.7 | No data |
| RP11-128B16.3 | No data |
| RP11-128P17.1 | No data |
| RP11-128P17.2 | No data |
| RP11-128P17.4 | No data |
| RP11-12L8.1 | No data |
| RP11-12M5.3 | No data |
| RP11-132N15.1 | No data |
| RP11-132N15.2 | No data |
| RP11-132N15.3 | No data |
| RP11-1334A24.6 | No data |
| RP11-133F8.2 | No data |
| RP11-133L19.3 | No data |
| RP11-138H8.2 | No data |
| RP11-138I17.1 | No data |
| RP11-13J8.1 | No data |
| RP11-13N12.1 | No data |
| RP11-141O11.1 | No data |
| RP11-142A12.1 | No data |
| RP11-142G1.3 | No data |
| RP11-142M10.2 | No data |
| RP11-143J12.3 | No data |
| RP11-145M4.1 | No data |
| RP11-146E13.4 | No data |
| RP11-146N18.1 | No data |
| RP11-146N23.4 | No data |
| RP11-148B3.2 | No data |
| RP11-14C10.5 | No data |
| RP11-14K3.7 | No data |
| RP11-152O14.1 | No data |
| RP11-15I20.1 | No data |
| RP11-15M15.2 | No data |
| RP11-161D15.2 | No data |
| RP11-162J8.3 | No data |
| RP11-165M1.3 | No data |
| RP11-166B2.7 | No data |
| RP11-167B3.2 | No data |
| RP11-167B3.3 | No data |
| RP11-167N24.6 | No data |
| RP11-168L22.2 | No data |
| RP11-168O22.1 | No data |
| RP11-169E6.4 | No data |
| RP11-16D22.2 | No data |
| RP11-172E10.1 | No data |
| RP11-173L6.1 | No data |
| RP11-177B4.2 | No data |
| RP11-178L8.9 | No data |
| RP11-181G12.4 | No data |
| RP11-183I6.2 | No data |
| RP11-184A2.2 | No data |
| RP11-184A2.3 | No data |
| RP11-184I16.4 | No data |
| RP11-18H21.2 | No data |
| RP11-18H21.3 | No data |
| RP11-191N8.2 | No data |
| RP11-192P3.4 | No data |
| RP11-195B3.1 | No data |
| RP11-196H14.4 | No data |
| RP11-19D2.2 | No data |
| RP11-19E11.1 | No data |
| RP11-19J5.2 | No data |
| RP11-1H15.2 | No data |
| RP11-201E8.1 | No data |
| RP11-202D18.2 | No data |
| RP11-202G18.1 | No data |
| RP11-203E8.1 | No data |
| RP11-204N11.1 | No data |
| RP11-208K4.1 | No data |
| RP11-20D14.3 | No data |
| RP11-20G13.2 | No data |
| RP11-20G13.3 | No data |
| RP11-20I20.4 | No data |
| RP11-211N11.5 | No data |
| RP11-212I21.5 | No data |
| RP11-217E22.5 | No data |
| RP11-218E20.5 | No data |
| RP11-219E7.2 | No data |
| RP11-219E7.3 | No data |
| RP11-21A7A.2 | No data |
| RP11-21A7A.4 | No data |
| RP11-227G15.8 | No data |
| RP11-229P13.15 | No data |
| RP11-229P13.19 | No data |
| RP11-238I10.1 | No data |
| RP11-238K6.1 | No data |
| RP11-240L7.4 | No data |
| RP11-242P2.2 | No data |
| RP11-245J24.1 | No data |
| RP11-247A12.1 | No data |
| RP11-254F7.3 | No data |
| RP11-255M2.2 | No data |
| RP11-258F22.2 | No data |
| RP11-25E2.1 | No data |
| RP11-25G10.2 | No data |
| RP11-25K21.6 | No data |
| RP11-25O3.1 | No data |
| RP11-263E1.1 | No data |
| RP11-264A11.1 | No data |
| RP11-264K23.1 | No data |
| RP11-265D19.6 | No data |
| RP11-266E6.3 | No data |
| RP11-267A15.3 | No data |
| RP11-267N12.1 | No data |
| RP11-269G24.6 | No data |
| RP11-26E5.1 | No data |
| RP11-272D12.2 | No data |
| RP11-274H2.2 | No data |
| RP11-275I4.2 | No data |
| RP11-278A23.2 | No data |
| RP11-278H7.3 | No data |
| RP11-278H7.4 | No data |
| RP11-279O17.1 | No data |
| RP11-27G22.1 | No data |
| RP11-283C24.1 | No data |
| RP11-284H18.1 | No data |
| RP11-285E9.5 | No data |
| RP11-285G1.9 | No data |
| RP11-28G8.1 | No data |
| RP11-293M10.1 | No data |
| RP11-295I5.4 | No data |
| RP11-298O21.2 | No data |
| RP11-298O21.6 | No data |
| RP11-299P2.1 | No data |
| RP11-2I17.4 | No data |
| RP11-2L8.1 | No data |
| RP11-2L8.2 | No data |
| RP11-2N5.2 | No data |
| RP11-2O17.2 | No data |
| RP11-301G7.1 | No data |
| RP11-302F12.10 | No data |
| RP11-302L19.3 | No data |
| RP11-304L19.4 | No data |
| RP11-305P14.1 | No data |
| RP11-307L3.4 | No data |
| RP11-309M7.1 | No data |
| RP11-30L3.2 | No data |
| RP11-310P5.2 | No data |
| RP11-311F12.2 | No data |
| RP11-313P18.1 | No data |
| RP11-315F22.1 | No data |
| RP11-318A15.8 | No data |
| RP11-318G21.4 | No data |
| RP11-319E12.2 | No data |
| RP11-320H14.1 | No data |
| RP11-321P16.1 | No data |
| RP11-322D14.2 | No data |
| RP11-325E5.1 | No data |
| RP11-329N22.1 | No data |
| RP11-331K21.1 | No data |
| RP11-332J15.1 | No data |
| RP11-332J15.3 | No data |
| RP11-333B11.1 | No data |
| RP11-336A10.5 | No data |
| RP11-338E21.2 | No data |
| RP11-338L22.2 | No data |
| RP11-338O1.2 | No data |
| RP11-340F14.6 | No data |
| RP11-344E13.4 | No data |
| RP11-344N10.2 | No data |
| RP11-344P13.4 | No data |
| RP11-347P5.1 | No data |
| RP11-349K16.1 | No data |
| RP11-34F13.3 | No data |
| RP11-350D17.2 | No data |
| RP11-350G24.1 | No data |
| RP11-351A20.1 | No data |
| RP11-351M16.3 | No data |
| RP11-354P11.2 | No data |
| RP11-354P11.8 | No data |
| RP11-357D18.1 | No data |
| RP11-357N13.1 | No data |
| RP11-357N13.2 | No data |
| RP11-357N13.3 | No data |
| RP11-358M14.2 | No data |
| RP11-359N11.1 | No data |
| RP11-35J10.7 | No data |
| RP11-35J23.1 | No data |
| RP11-360I2.1 | No data |
| RP11-361I14.2 | No data |
| RP11-361L15.4 | No data |
| RP11-367J7.3 | No data |
| RP11-370B11.3 | No data |
| RP11-370F5.4 | No data |
| RP11-373E16.3 | No data |
| RP1-137K24.1 | No data |
| RP11-383C6.2 | No data |
| RP11-383J24.1 | No data |
| RP11-385G16.1 | No data |
| RP11-386B13.4 | No data |
| RP11-386M24.9 | No data |
| RP11-388C12.5 | No data |
| RP11-38J22.3 | No data |
| RP11-390E23.3 | No data |
| RP11-390N6.1 | No data |
| RP11-392B6.1 | No data |
| RP11-393N21.2 | No data |
| RP11-396O20.1 | No data |
| RP11-396O20.2 | No data |
| RP11-399F2.2 | No data |
| RP11-39M21.1 | No data |
| RP11-39M21.2 | No data |
| RP11-3G21.1 | No data |
| RP11-3J1.1 | No data |
| RP11-3P22.2 | No data |
| RP11-400N13.3 | No data |
| RP11-401F2.4 | No data |
| RP11-404O13.1 | No data |
| RP11-406H4.1 | No data |
| RP11-407A16.3 | No data |
| RP11-408N14.1 | No data |
| RP11-411B10.2 | No data |
| RP11-414H23.2 | No data |
| RP11-414H23.3 | No data |
| RP11-414J4.2 | No data |
| RP11-415C15.1 | No data |
| RP11-415D17.3 | No data |
| RP11-416A14.1 | No data |
| RP11-421P23.2 | No data |
| RP11-422J15.1 | No data |
| RP11-428C19.4 | No data |
| RP11-428C19.5 | No data |
| RP11-429J17.5 | No data |
| RP11-42A4.1 | No data |
| RP1-142L7.5 | No data |
| RP11-42O4.2 | No data |
| RP11-432J24.2 | No data |
| RP11-433J22.3 | No data |
| RP11-436F23.1 | No data |
| RP11-438D8.2 | No data |
| RP11-43D2.2 | No data |
| RP11-442J21.2 | No data |
| RP11-445F12.2 | No data |
| RP11-445K13.2 | No data |
| RP11-445N18.7 | No data |
| RP11-445P17.3 | No data |
| RP1-144F13.3 | No data |
| RP11-44K6.2 | No data |
| RP11-44K6.4 | No data |
| RP11-452C13.1 | No data |
| RP11-452L6.8 | No data |
| RP11-454P21.1 | No data |
| RP11-455F5.4 | No data |
| RP11-455F5.5 | No data |
| RP11-456I15.2 | No data |
| RP11-457K10.1 | No data |
| RP11-460I13.2 | No data |
| RP11-462B18.2 | No data |
| RP11-465K16.1 | No data |
| RP11-469N6.3 | No data |
| RP11-472N13.2 | No data |
| RP11-473C19.1 | No data |
| RP11-473L15.3 | No data |
| RP11-474B12.1 | No data |
| RP11-474I11.7 | No data |
| RP11-474I11.8 | No data |
| RP11-475O23.2 | No data |
| RP11-476D10.1 | No data |
| RP11-476H24.1 | No data |
| RP11-478P10.1 | No data |
| RP11-479J7.2 | No data |
| RP11-47J17.3 | No data |
| RP11-480D4.1 | No data |
| RP11-480D4.2 | No data |
| RP11-481C4.1 | No data |
| RP11-482E14.2 | No data |
| RP11-483L5.1 | No data |
| RP11-483P21.6 | No data |
| RP11-486L19.2 | No data |
| RP11-486M23.2 | No data |
| RP11-489G11.3 | No data |
| RP11-489O18.1 | No data |
| RP11-493P1.2 | No data |
| RP11-495K9.5 | No data |
| RP11-495O11.1 | No data |
| RP11-497D6.5 | No data |
| RP1-149A16.12 | No data |
| RP11-4O3.1 | No data |
| RP11-506B6.6 | No data |
| RP11-506E9.3 | No data |
| RP11-508N22.9 | No data |
| RP11-509A17.3 | No data |
| RP11-50B3.2 | No data |
| RP11-510C10.2 | No data |
| RP11-510C10.3 | No data |
| RP11-513G11.2 | No data |
| RP11-514D23.1 | No data |
| RP11-516J2.1 | No data |
| RP11-51G5.1 | No data |
| RP11-521M14.2 | No data |
| RP11-523O18.5 | No data |
| RP11-526F3.1 | No data |
| RP11-526N18.1 | No data |
| RP11-527L4.6 | No data |
| RP11-528G1.2 | No data |
| RP11-529E15.1 | No data |
| RP11-531H8.2 | No data |
| RP11-533O20.2 | No data |
| RP11-536C5.2 | No data |
| RP11-536G4.1 | No data |
| RP11-53B2.1 | No data |
| RP11-53B5.1 | No data |
| RP11-540K16.1 | No data |
| RP11-543D5.1 | No data |
| RP11-543G18.1 | No data |
| RP11-543H12.1 | No data |
| RP11-543N12.1 | No data |
| RP11-545A16.1 | No data |
| RP11-549L6.3 | No data |
| RP11-54A4.2 | No data |
| RP1-154K9.2 | No data |
| RP11-550H2.1 | No data |
| RP11-550H2.2 | No data |
| RP11-552D4.1 | No data |
| RP11-552E20.1 | No data |
| RP11-553K8.5 | No data |
| RP11-554A11.5 | No data |
| RP11-554D15.1 | No data |
| RP11-554D15.3 | No data |
| RP11-554D15.4 | No data |
| RP11-554D20.1 | No data |
| RP11-554E23.2 | No data |
| RP11-554L12.1 | No data |
| RP11-556E13.1 | No data |
| RP11-556O9.2 | No data |
| RP11-557C18.4 | No data |
| RP11-55L3.1 | No data |
| RP11-563M4.1 | No data |
| RP11-563N6.6 | No data |
| RP11-565P22.2 | No data |
| RP11-566H8.3 | No data |
| RP11-566K11.7 | No data |
| RP11-567E21.3 | No data |
| RP11-567M16.2 | No data |
| RP11-568J23.4 | No data |
| RP11-569G13.3 | No data |
| RP11-570L15.1 | No data |
| RP11-575A19.2 | No data |
| RP11-575B7.3 | No data |
| RP11-578F21.6 | No data |
| RP11-586L23.1 | No data |
| RP11-58A18.1 | No data |
| RP11-58G13.1 | No data |
| RP1-158P9.1 | No data |
| RP11-595B24.2 | No data |
| RP11-598F7.5 | No data |
| RP11-598F7.6 | No data |
| RP11-59O6.3 | No data |
| RP11-5L12.1 | No data |
| RP11-5P22.3 | No data |
| RP11-5P4.1 | No data |
| RP11-5P4.3 | No data |
| RP11-605F22.1 | No data |
| RP11-609N14.1 | No data |
| RP11-616M22.1 | No data |
| RP11-616M22.2 | No data |
| RP11-616M22.3 | No data |
| RP11-616M22.5 | No data |
| RP11-617B3.2 | No data |
| RP11-61O1.1 | No data |
| RP11-622A1.2 | No data |
| RP11-626H12.2 | No data |
| RP11-626P14.1 | No data |
| RP11-62F24.1 | No data |
| RP11-630D6.5 | No data |
| RP11-631F7.1 | No data |
| RP11-638I2.9 | No data |
| RP11-63A11.1 | No data |
| RP11-63G10.2 | No data |
| RP1-163G9.2 | No data |
| RP11-640N11.2 | No data |
| RP11-641D5.2 | No data |
| RP11-642C5.1 | No data |
| RP11-642D21.2 | No data |
| RP11-643C9.2 | No data |
| RP11-64B16.4 | No data |
| RP11-64D24.2 | No data |
| RP11-64D24.4 | No data |
| RP11-64P14.7 | No data |
| RP11-650L12.1 | No data |
| RP11-654A16.1 | No data |
| RP11-654G14.1 | No data |
| RP11-659E9.2 | No data |
| RP11-659P15.1 | No data |
| RP11-65J3.2 | No data |
| RP11-661C8.2 | No data |
| RP11-662I13.2 | No data |
| RP11-662M24.2 | No data |
| RP11-66A2.2 | No data |
| RP11-66B24.5 | No data |
| RP11-66H6.3 | No data |
| RP11-66N11.7 | No data |
| RP11-670N15.1 | No data |
| RP11-671P2.1 | No data |
| RP11-673E11.2 | No data |
| RP11-674P19.2 | No data |
| RP11-675F6.3 | No data |
| RP11-678G15.1 | No data |
| RP11-678G15.2 | No data |
| RP11-67C2.2 | No data |
| RP11-67M9.1 | No data |
| RP1-167O22.1 | No data |
| RP11-680B3.2 | No data |
| RP11-685F15.1 | No data |
| RP11-686G23.2 | No data |
| RP11-687D19.1 | No data |
| RP1-168P16.2 | No data |
| RP11-691H4.3 | No data |
| RP11-691H4.4 | No data |
| RP11-696F12.1 | No data |
| RP1-16A9.1 | No data |
| RP11-6L6.2 | No data |
| RP11-700H6.2 | No data |
| RP11-701H16.4 | No data |
| RP11-701I24.3 | No data |
| RP11-702F3.1 | No data |
| RP11-702L15.4 | No data |
| RP11-703M24.5 | No data |
| RP11-70D24.4 | No data |
| RP11-70J12.1 | No data |
| RP1-170O19.17 | No data |
| RP1-170O19.23 | No data |
| RP11-70O5.2 | No data |
| RP11-711K1.7 | No data |
| RP11-713P17.5 | No data |
| RP11-714L20.1 | No data |
| RP11-716O23.1 | No data |
| RP11-719N22.2 | No data |
| RP11-737F9.1 | No data |
| RP11-737O24.1 | No data |
| RP11-739N10.1 | No data |
| RP11-73M11.3 | No data |
| RP11-73M14.1 | No data |
| RP11-744D14.2 | No data |
| RP11-74M11.2 | No data |
| RP11-750H9.7 | No data |
| RP11-751H17.1 | No data |
| RP11-752D24.2 | No data |
| RP11-753D20.3 | No data |
| RP11-753H16.5 | No data |
| RP11-754N21.1 | No data |
| RP11-75C10.9 | No data |
| RP11-75L1.1 | No data |
| RP11-761I4.4 | No data |
| RP11-763F8.1 | No data |
| RP11-766F14.1 | No data |
| RP11-767N15.1 | No data |
| RP11-76C10.2 | No data |
| RP11-772C9.1 | No data |
| RP11-774D14.1 | No data |
| RP11-789C2.1 | No data |
| RP11-78C3.1 | No data |
| RP11-799B12.2 | No data |
| RP11-804N13.1 | No data |
| RP11-805I24.1 | No data |
| RP11-807H17.1 | No data |
| RP11-809C18.3 | No data |
| RP11-809N8.4 | No data |
| RP11-815J21.1 | No data |
| RP11-815M8.1 | No data |
| RP11-816J8.1 | No data |
| RP11-826N14.2 | No data |
| RP11-831A10.2 | No data |
| RP11-831H9.3 | No data |
| RP11-837J7.4 | No data |
| RP11-83C7.1 | No data |
| RP11-843A23.1 | No data |
| RP11-848P1.3 | No data |
| RP11-84D1.1 | No data |
| RP11-84D1.2 | No data |
| RP11-850F7.7 | No data |
| RP11-856M7.1 | No data |
| RP11-856M7.2 | No data |
| RP11-85O21.5 | No data |
| RP11-861E21.1 | No data |
| RP11-864I4.3 | No data |
| RP11-867G23.1 | No data |
| RP11-867G23.12 | No data |
| RP11-867G23.13 | No data |
| RP11-879F14.1 | No data |
| RP11-881L2.1 | No data |
| RP11-881M11.1 | No data |
| RP11-883G14.3 | No data |
| RP11-883G14.4 | No data |
| RP11-886P16.6 | No data |
| RP11-88H10.2 | No data |
| RP11-89B16.1 | No data |
| RP11-91I20.4 | No data |
| RP11-91K8.5 | No data |
| RP11-941H19.3 | No data |
| RP11-946L16.2 | No data |
| RP11-94A24.1 | No data |
| RP11-955H22.1 | No data |
| RP11-95P13.1 | No data |
| RP11-95P13.2 | No data |
| RP11-96B2.1 | No data |
| RP11-989F5.3 | No data |
| RP1-205F14P.1 | No data |
| RP1-207H1.3 | No data |
| RP1-209A6.1 | No data |
| RP1-225E12.3 | No data |
| RP1-228P16.4 | No data |
| RP1-251I12.1 | No data |
| RP1-283K11.2 | No data |
| RP1-288H2.2 | No data |
| RP1-29C18.10 | No data |
| RP13-1016M1.2 | No data |
| RP13-210D15.4 | No data |
| RP13-216E22.5 | No data |
| RP13-259N13.2 | No data |
| RP13-297E16.5 | No data |
| RP13-30A9.1 | No data |
| RP13-30A9.2 | No data |
| RP13-379O24.2 | No data |
| RP13-436F16.1 | No data |
| RP13-494C23.1 | No data |
| RP13-60M5.2 | No data |
| RP13-884E18.4 | No data |
| RP13-895J2.7 | No data |
| RP1-38C16.2 | No data |
| RP1-40E16.2 | No data |
| RP1-55C23.7 | No data |
| RP1-56J10.8 | No data |
| RP1-56K13.5 | No data |
| RP1-58B11.1 | No data |
| RP1-60O19.2 | No data |
| RP1-63G5.7 | No data |
| RP1-67A8.3 | No data |
| RP1-69D17.3 | No data |
| RP1-71H24.1 | No data |
| RP1-7G5.6 | No data |
| RP1-81D8.3 | No data |
| RP1-90G24.11 | No data |
| RP3-359N14.2 | No data |
| RP3-368B9.2 | No data |
| RP3-369A17.4 | No data |
| RP3-388N13.3 | No data |
| RP3-395M20.3 | No data |
| RP3-399L15.1 | No data |
| RP3-404F18.5 | No data |
| RP3-413H6.2 | No data |
| RP3-417L20.4 | No data |
| RP3-429O6.1 | No data |
| RP3-434P1.6 | No data |
| RP3-438O4.4 | No data |
| RP3-462C17.1 | No data |
| RP3-470L22.1 | No data |
| RP3-492J12.2 | No data |
| RP3-495K2.2 | No data |
| RP3-495K2.3 | No data |
| RP3-527G5.1 | No data |
| RP4-535B20.4 | No data |
| RP4-536B24.3 | No data |
| RP4-539M6.14 | No data |
| RP4-543J13.1 | No data |
| RP4-547N15.3 | No data |
| RP4-564F22.6 | No data |
| RP4-569D19.8 | No data |
| RP4-594I10.3 | No data |
| RP4-604K5.3 | No data |
| RP4-640H8.2 | No data |
| RP4-655J12.5 | No data |
| RP4-668E10.4 | No data |
| RP4-671O14.7 | No data |
| RP4-694A7.2 | No data |
| RP4-710M16.2 | No data |
| RP4-712E4.1 | No data |
| RP4-719C8.1 | No data |
| RP4-735C1.4 | No data |
| RP4-753P9.3 | No data |
| RP5-1018K9.1 | No data |
| RP5-1024C24.1 | No data |
| RP5-1028L10.2 | No data |
| RP5-1031J8.1 | No data |
| RP5-1057B20.3 | No data |
| RP5-1069C8.2 | No data |
| RP5-1077H22.1 | No data |
| RP5-1096D14.3 | No data |
| RP5-1100I6.1 | No data |
| RP5-1107A17.3 | No data |
| RP5-1121A15.3 | No data |
| RP5-1173A5.1 | No data |
| RP5-1195D24.1 | No data |
| RP5-859D4.3 | No data |
| RP5-867C24.5 | No data |
| RP5-881L22.6 | No data |
| RP5-896L10.1 | No data |
| RP5-899E9.1 | No data |
| RP5-912I13.1 | No data |
| RP5-916L7.2 | No data |
| RP5-921G16.2 | No data |
| RP5-940J5.8 | No data |
| RP5-951N9.1 | No data |
| RP5-968J1.1 | No data |
| RP5-983L19.2 | No data |
| RP5-988G17.1 | No data |
| RP6-91H8.1 | No data |
| RPS6KA2-AS1 | No data |
| SALRNA1 | No data |
| SALRNA2 | No data |
| SIRPG-AS1 | No data |
| SLC14A2-AS1 | No data |
| SLC6A1-AS1 | No data |
| SPATA13-AS1 | No data |
| SRGAP3-AS4 | No data |
| TBL1XR1-AS1 | No data |
| TBX18-AS1 | No data |
| TBX5-AS1 | No data |
| TCF4-AS2 | No data |
| TGFA-IT1 | No data |
| TMEM246-AS1 | No data |
| TMEM92-AS1 | No data |
| TPRG1-AS2 | No data |
| TRBV11-2 | No data |
| TRIM36-IT1 | No data |
| TRPC7-AS1 | No data |
| TRPC7-AS2 | No data |
| TTC21B-AS1 | No data |
| U47924.27 | No data |
| VCAN-AS1 | No data |
| WASF3-AS1 | No data |
| WI2-80269A6.1 | No data |
| WI2-8325B5.1 | No data |
| WSPAR | No data |
| WWC3-AS1 | No data |
| XIAP-AS1 | No data |
| XXbac-B33L19.12 | No data |
| XXbac-B476C20.13 | No data |
| XXbac-B476C20.14 | No data |
| XXbac-BPG170G13.32 | No data |
| XXbac-BPG248L24.13 | No data |
| XXbac-BPG254F23.7 | No data |
| XXbac-BPG299F13.14 | No data |
| XXbac-BPG300A18.13 | No data |
| Z69666.2 | No data |
| Z69720.2 | No data |
| ZBTB46-AS1 | No data |

**Table S4. The interactions of the ceRNA network in KIRC**

| **lncRNA** | **miRNA** | **mRNA** |
| --- | --- | --- |
| TPTEP1 | hsa-mir-122 | GALNT3 |
| C15orf56 | hsa-mir-122 | GALNT3 |
| LINC00303 | hsa-mir-122 | GALNT3 |
| LINC00314 | hsa-mir-122 | GALNT3 |
| SNHG12 | hsa-mir-122 | GALNT3 |
| RMRP | hsa-mir-122 | GALNT3 |
| PHEX-AS1 | hsa-mir-122 | GALNT3 |
| SLC25A5-AS1 | hsa-mir-122 | GALNT3 |
| SFTA1P | hsa-mir-122 | GALNT3 |
| ARHGAP26-AS1 | hsa-mir-122 | GALNT3 |
| LINC00343 | hsa-mir-122 | GALNT3 |
| LINC00355 | hsa-mir-122 | GALNT3 |
| LINC00111 | hsa-mir-122 | GALNT3 |
| NLGN1-AS1 | hsa-mir-122 | GALNT3 |
| LMO7-AS1 | hsa-mir-122 | GALNT3 |
| STEAP3-AS1 | hsa-mir-122 | GALNT3 |
| AOAH-IT1 | hsa-mir-122 | GALNT3 |
| LINC00410 | hsa-mir-122 | GALNT3 |
| ATP11A-AS1 | hsa-mir-122 | GALNT3 |
| AC108463.1 | hsa-mir-122 | GALNT3 |
| MYCNOS | hsa-mir-122 | GALNT3 |
| C5orf58 | hsa-mir-122 | GALNT3 |
| DSCAM-AS1 | hsa-mir-122 | GALNT3 |
| HM13-IT1 | hsa-mir-122 | GALNT3 |
| LINC00426 | hsa-mir-122 | GALNT3 |
| ARAP1-AS2 | hsa-mir-122 | GALNT3 |
| LINC00461 | hsa-mir-122 | GALNT3 |
| MIR210HG | hsa-mir-122 | GALNT3 |
| FAM13A-AS1 | hsa-mir-122 | GALNT3 |
| C1orf147 | hsa-mir-141 | PRELID2 |
| LY86-AS1 | hsa-mir-141 | PRELID2 |
| TSSC1-IT1 | hsa-mir-141 | PRELID2 |
| MIAT | hsa-mir-141 | PRELID2 |
| C12orf77 | hsa-mir-141 | PRELID2 |
| LINC00355 | hsa-mir-141 | PRELID2 |
| NLGN1-AS1 | hsa-mir-141 | PRELID2 |
| STEAP3-AS1 | hsa-mir-141 | PRELID2 |
| LINC00443 | hsa-mir-141 | PRELID2 |
| BPESC1 | hsa-mir-141 | PRELID2 |
| LINC00472 | hsa-mir-141 | PRELID2 |
| LATS2-AS1 | hsa-mir-141 | PRELID2 |
| DSCAM-AS1 | hsa-mir-141 | PRELID2 |
| LINC00426 | hsa-mir-141 | PRELID2 |
| ARHGEF26-AS1 | hsa-mir-141 | PRELID2 |
| LINC00461 | hsa-mir-141 | PRELID2 |
| FAM13A-AS1 | hsa-mir-141 | PRELID2 |
| VCAN-AS1 | hsa-mir-141 | PRELID2 |
| LINC00492 | hsa-mir-141 | PRELID2 |
| C15orf56 | hsa-mir-155 | GPM6B |
| LY86-AS1 | hsa-mir-155 | GPM6B |
| MIAT | hsa-mir-155 | GPM6B |
| PCGEM1 | hsa-mir-155 | GPM6B |
| NLGN1-AS1 | hsa-mir-155 | GPM6B |
| STEAP3-AS1 | hsa-mir-155 | GPM6B |
| LINC00472 | hsa-mir-155 | GPM6B |
| MIR155HG | hsa-mir-155 | GPM6B |
| ARHGEF26-AS1 | hsa-mir-155 | GPM6B |
| TRIM36-IT1 | hsa-mir-155 | GPM6B |
| C15orf56 | hsa-mir-155 | CD36 |
| LY86-AS1 | hsa-mir-155 | CD36 |
| MIAT | hsa-mir-155 | CD36 |
| PCGEM1 | hsa-mir-155 | CD36 |
| NLGN1-AS1 | hsa-mir-155 | CD36 |
| STEAP3-AS1 | hsa-mir-155 | CD36 |
| LINC00472 | hsa-mir-155 | CD36 |
| MIR155HG | hsa-mir-155 | CD36 |
| ARHGEF26-AS1 | hsa-mir-155 | CD36 |
| TRIM36-IT1 | hsa-mir-155 | CD36 |
| C15orf56 | hsa-mir-155 | PCDH9 |
| LY86-AS1 | hsa-mir-155 | PCDH9 |
| MIAT | hsa-mir-155 | PCDH9 |
| PCGEM1 | hsa-mir-155 | PCDH9 |
| NLGN1-AS1 | hsa-mir-155 | PCDH9 |
| STEAP3-AS1 | hsa-mir-155 | PCDH9 |
| LINC00472 | hsa-mir-155 | PCDH9 |
| MIR155HG | hsa-mir-155 | PCDH9 |
| ARHGEF26-AS1 | hsa-mir-155 | PCDH9 |
| TRIM36-IT1 | hsa-mir-155 | PCDH9 |
| C15orf56 | hsa-mir-155 | ITK |
| LY86-AS1 | hsa-mir-155 | ITK |
| MIAT | hsa-mir-155 | ITK |
| PCGEM1 | hsa-mir-155 | ITK |
| NLGN1-AS1 | hsa-mir-155 | ITK |
| STEAP3-AS1 | hsa-mir-155 | ITK |
| LINC00472 | hsa-mir-155 | ITK |
| MIR155HG | hsa-mir-155 | ITK |
| ARHGEF26-AS1 | hsa-mir-155 | ITK |
| TRIM36-IT1 | hsa-mir-155 | ITK |
| C15orf56 | hsa-mir-155 | ERMP1 |
| LY86-AS1 | hsa-mir-155 | ERMP1 |
| MIAT | hsa-mir-155 | ERMP1 |
| PCGEM1 | hsa-mir-155 | ERMP1 |
| NLGN1-AS1 | hsa-mir-155 | ERMP1 |
| STEAP3-AS1 | hsa-mir-155 | ERMP1 |
| LINC00472 | hsa-mir-155 | ERMP1 |
| MIR155HG | hsa-mir-155 | ERMP1 |
| ARHGEF26-AS1 | hsa-mir-155 | ERMP1 |
| TRIM36-IT1 | hsa-mir-155 | ERMP1 |
| C15orf56 | hsa-mir-155 | ZNF98 |
| LY86-AS1 | hsa-mir-155 | ZNF98 |
| MIAT | hsa-mir-155 | ZNF98 |
| PCGEM1 | hsa-mir-155 | ZNF98 |
| NLGN1-AS1 | hsa-mir-155 | ZNF98 |
| STEAP3-AS1 | hsa-mir-155 | ZNF98 |
| LINC00472 | hsa-mir-155 | ZNF98 |
| MIR155HG | hsa-mir-155 | ZNF98 |
| ARHGEF26-AS1 | hsa-mir-155 | ZNF98 |
| TRIM36-IT1 | hsa-mir-155 | ZNF98 |
| C15orf56 | hsa-mir-155 | ZIC3 |
| LY86-AS1 | hsa-mir-155 | ZIC3 |
| MIAT | hsa-mir-155 | ZIC3 |
| PCGEM1 | hsa-mir-155 | ZIC3 |
| NLGN1-AS1 | hsa-mir-155 | ZIC3 |
| STEAP3-AS1 | hsa-mir-155 | ZIC3 |
| LINC00472 | hsa-mir-155 | ZIC3 |
| MIR155HG | hsa-mir-155 | ZIC3 |
| ARHGEF26-AS1 | hsa-mir-155 | ZIC3 |
| TRIM36-IT1 | hsa-mir-155 | ZIC3 |
| C15orf56 | hsa-mir-155 | TYRP1 |
| LY86-AS1 | hsa-mir-155 | TYRP1 |
| MIAT | hsa-mir-155 | TYRP1 |
| PCGEM1 | hsa-mir-155 | TYRP1 |
| NLGN1-AS1 | hsa-mir-155 | TYRP1 |
| STEAP3-AS1 | hsa-mir-155 | TYRP1 |
| LINC00472 | hsa-mir-155 | TYRP1 |
| MIR155HG | hsa-mir-155 | TYRP1 |
| ARHGEF26-AS1 | hsa-mir-155 | TYRP1 |
| TRIM36-IT1 | hsa-mir-155 | TYRP1 |
| C15orf56 | hsa-mir-155 | SPI1 |
| LY86-AS1 | hsa-mir-155 | SPI1 |
| MIAT | hsa-mir-155 | SPI1 |
| PCGEM1 | hsa-mir-155 | SPI1 |
| NLGN1-AS1 | hsa-mir-155 | SPI1 |
| STEAP3-AS1 | hsa-mir-155 | SPI1 |
| LINC00472 | hsa-mir-155 | SPI1 |
| MIR155HG | hsa-mir-155 | SPI1 |
| ARHGEF26-AS1 | hsa-mir-155 | SPI1 |
| TRIM36-IT1 | hsa-mir-155 | SPI1 |
| TPTEP1 | hsa-mir-206 | BDNF |
| C15orf54 | hsa-mir-206 | BDNF |
| C15orf56 | hsa-mir-206 | BDNF |
| COL18A1-AS1 | hsa-mir-206 | BDNF |
| LINC00158 | hsa-mir-206 | BDNF |
| SNHG12 | hsa-mir-206 | BDNF |
| RMRP | hsa-mir-206 | BDNF |
| SFTA1P | hsa-mir-206 | BDNF |
| MIAT | hsa-mir-206 | BDNF |
| C12orf77 | hsa-mir-206 | BDNF |
| STEAP3-AS1 | hsa-mir-206 | BDNF |
| TTLL7-IT1 | hsa-mir-206 | BDNF |
| LINC00460 | hsa-mir-206 | BDNF |
| C5orf58 | hsa-mir-206 | BDNF |
| MCF2L-AS1 | hsa-mir-206 | BDNF |
| LINC00427 | hsa-mir-206 | BDNF |
| DAPK1-IT1 | hsa-mir-206 | BDNF |
| LINC00426 | hsa-mir-206 | BDNF |
| CCDC39-AS1 | hsa-mir-206 | BDNF |
| ARAP1-AS2 | hsa-mir-206 | BDNF |
| MIR210HG | hsa-mir-206 | BDNF |
| TPTEP1 | hsa-mir-206 | SFRP1 |
| C15orf54 | hsa-mir-206 | SFRP1 |
| C15orf56 | hsa-mir-206 | SFRP1 |
| COL18A1-AS1 | hsa-mir-206 | SFRP1 |
| LINC00158 | hsa-mir-206 | SFRP1 |
| SNHG12 | hsa-mir-206 | SFRP1 |
| RMRP | hsa-mir-206 | SFRP1 |
| SFTA1P | hsa-mir-206 | SFRP1 |
| MIAT | hsa-mir-206 | SFRP1 |
| C12orf77 | hsa-mir-206 | SFRP1 |
| STEAP3-AS1 | hsa-mir-206 | SFRP1 |
| TTLL7-IT1 | hsa-mir-206 | SFRP1 |
| LINC00460 | hsa-mir-206 | SFRP1 |
| C5orf58 | hsa-mir-206 | SFRP1 |
| MCF2L-AS1 | hsa-mir-206 | SFRP1 |
| LINC00427 | hsa-mir-206 | SFRP1 |
| DAPK1-IT1 | hsa-mir-206 | SFRP1 |
| LINC00426 | hsa-mir-206 | SFRP1 |
| CCDC39-AS1 | hsa-mir-206 | SFRP1 |
| ARAP1-AS2 | hsa-mir-206 | SFRP1 |
| MIR210HG | hsa-mir-206 | SFRP1 |
| TPTEP1 | hsa-mir-206 | STC2 |
| C15orf54 | hsa-mir-206 | STC2 |
| C15orf56 | hsa-mir-206 | STC2 |
| COL18A1-AS1 | hsa-mir-206 | STC2 |
| LINC00158 | hsa-mir-206 | STC2 |
| SNHG12 | hsa-mir-206 | STC2 |
| RMRP | hsa-mir-206 | STC2 |
| SFTA1P | hsa-mir-206 | STC2 |
| MIAT | hsa-mir-206 | STC2 |
| C12orf77 | hsa-mir-206 | STC2 |
| STEAP3-AS1 | hsa-mir-206 | STC2 |
| TTLL7-IT1 | hsa-mir-206 | STC2 |
| LINC00460 | hsa-mir-206 | STC2 |
| C5orf58 | hsa-mir-206 | STC2 |
| MCF2L-AS1 | hsa-mir-206 | STC2 |
| LINC00427 | hsa-mir-206 | STC2 |
| DAPK1-IT1 | hsa-mir-206 | STC2 |
| LINC00426 | hsa-mir-206 | STC2 |
| CCDC39-AS1 | hsa-mir-206 | STC2 |
| ARAP1-AS2 | hsa-mir-206 | STC2 |
| MIR210HG | hsa-mir-206 | STC2 |
| C1orf147 | hsa-mir-21 | FASLG |
| LINC00221 | hsa-mir-21 | FASLG |
| ASH1L-IT1 | hsa-mir-21 | FASLG |
| ITCH-IT1 | hsa-mir-21 | FASLG |
| C5orf58 | hsa-mir-21 | FASLG |
| SRGAP3-AS4 | hsa-mir-21 | FASLG |
| GPC6-AS1 | hsa-mir-21 | FASLG |
| ARHGEF26-AS1 | hsa-mir-21 | FASLG |
| RERG-IT1 | hsa-mir-21 | FASLG |
| C1orf147 | hsa-mir-21 | TGFBI |
| LINC00221 | hsa-mir-21 | TGFBI |
| ASH1L-IT1 | hsa-mir-21 | TGFBI |
| ITCH-IT1 | hsa-mir-21 | TGFBI |
| C5orf58 | hsa-mir-21 | TGFBI |
| SRGAP3-AS4 | hsa-mir-21 | TGFBI |
| GPC6-AS1 | hsa-mir-21 | TGFBI |
| ARHGEF26-AS1 | hsa-mir-21 | TGFBI |
| RERG-IT1 | hsa-mir-21 | TGFBI |
| C1orf147 | hsa-mir-21 | CCL20 |
| LINC00221 | hsa-mir-21 | CCL20 |
| ASH1L-IT1 | hsa-mir-21 | CCL20 |
| ITCH-IT1 | hsa-mir-21 | CCL20 |
| C5orf58 | hsa-mir-21 | CCL20 |
| SRGAP3-AS4 | hsa-mir-21 | CCL20 |
| GPC6-AS1 | hsa-mir-21 | CCL20 |
| ARHGEF26-AS1 | hsa-mir-21 | CCL20 |
| RERG-IT1 | hsa-mir-21 | CCL20 |
| TPTEP1 | hsa-mir-216b | COL4A4 |
| C15orf56 | hsa-mir-216b | COL4A4 |
| LY86-AS1 | hsa-mir-216b | COL4A4 |
| SFTA1P | hsa-mir-216b | COL4A4 |
| MIAT | hsa-mir-216b | COL4A4 |
| C12orf77 | hsa-mir-216b | COL4A4 |
| LINC00200 | hsa-mir-216b | COL4A4 |
| LINC00410 | hsa-mir-216b | COL4A4 |
| BPESC1 | hsa-mir-216b | COL4A4 |
| LINC00472 | hsa-mir-216b | COL4A4 |
| LINC00426 | hsa-mir-216b | COL4A4 |
| LINC00461 | hsa-mir-216b | COL4A4 |
| MIR210HG | hsa-mir-216b | COL4A4 |
| TRIM36-IT1 | hsa-mir-216b | COL4A4 |
| LINC00507 | hsa-mir-216b | COL4A4 |
| TPTEP1 | hsa-mir-372 | GALNT3 |
| C15orf54 | hsa-mir-372 | GALNT3 |
| LINC00314 | hsa-mir-372 | GALNT3 |
| LINC00221 | hsa-mir-372 | GALNT3 |
| WWC3-AS1 | hsa-mir-372 | GALNT3 |
| MIAT | hsa-mir-372 | GALNT3 |
| C12orf77 | hsa-mir-372 | GALNT3 |
| NLGN1-AS1 | hsa-mir-372 | GALNT3 |
| LMO7-AS1 | hsa-mir-372 | GALNT3 |
| AKT3-IT1 | hsa-mir-372 | GALNT3 |
| LINC00379 | hsa-mir-372 | GALNT3 |
| STEAP3-AS1 | hsa-mir-372 | GALNT3 |
| ATP11A-AS1 | hsa-mir-372 | GALNT3 |
| TTLL7-IT1 | hsa-mir-372 | GALNT3 |
| LINC00472 | hsa-mir-372 | GALNT3 |
| LINC00462 | hsa-mir-372 | GALNT3 |
| KCNH1-IT1 | hsa-mir-372 | GALNT3 |
| C5orf58 | hsa-mir-372 | GALNT3 |
| MLIP-AS1 | hsa-mir-372 | GALNT3 |
| LINC00426 | hsa-mir-372 | GALNT3 |
| ARHGEF26-AS1 | hsa-mir-372 | GALNT3 |
| ARAP1-AS2 | hsa-mir-372 | GALNT3 |
| LINC00461 | hsa-mir-372 | GALNT3 |
| MIR210HG | hsa-mir-372 | GALNT3 |
| VCAN-AS1 | hsa-mir-372 | GALNT3 |
| LINC00507 | hsa-mir-372 | GALNT3 |
| TPTEP1 | hsa-mir-372 | CADM2 |
| C15orf54 | hsa-mir-372 | CADM2 |
| LINC00314 | hsa-mir-372 | CADM2 |
| LINC00221 | hsa-mir-372 | CADM2 |
| WWC3-AS1 | hsa-mir-372 | CADM2 |
| MIAT | hsa-mir-372 | CADM2 |
| C12orf77 | hsa-mir-372 | CADM2 |
| NLGN1-AS1 | hsa-mir-372 | CADM2 |
| LMO7-AS1 | hsa-mir-372 | CADM2 |
| AKT3-IT1 | hsa-mir-372 | CADM2 |
| LINC00379 | hsa-mir-372 | CADM2 |
| STEAP3-AS1 | hsa-mir-372 | CADM2 |
| ATP11A-AS1 | hsa-mir-372 | CADM2 |
| TTLL7-IT1 | hsa-mir-372 | CADM2 |
| LINC00472 | hsa-mir-372 | CADM2 |
| LINC00462 | hsa-mir-372 | CADM2 |
| KCNH1-IT1 | hsa-mir-372 | CADM2 |
| C5orf58 | hsa-mir-372 | CADM2 |
| MLIP-AS1 | hsa-mir-372 | CADM2 |
| LINC00426 | hsa-mir-372 | CADM2 |
| ARHGEF26-AS1 | hsa-mir-372 | CADM2 |
| ARAP1-AS2 | hsa-mir-372 | CADM2 |
| LINC00461 | hsa-mir-372 | CADM2 |
| MIR210HG | hsa-mir-372 | CADM2 |
| VCAN-AS1 | hsa-mir-372 | CADM2 |
| LINC00507 | hsa-mir-372 | CADM2 |
| TPTEP1 | hsa-mir-506 | VIM |
| C15orf56 | hsa-mir-506 | VIM |
| CSNK1G2-AS1 | hsa-mir-506 | VIM |
| LINC00221 | hsa-mir-506 | VIM |
| C20orf203 | hsa-mir-506 | VIM |
| RNU12 | hsa-mir-506 | VIM |
| LY86-AS1 | hsa-mir-506 | VIM |
| LINC00343 | hsa-mir-506 | VIM |
| PCGEM1 | hsa-mir-506 | VIM |
| LINC00355 | hsa-mir-506 | VIM |
| LINC00200 | hsa-mir-506 | VIM |
| STEAP3-AS1 | hsa-mir-506 | VIM |
| TPRG1-AS2 | hsa-mir-506 | VIM |
| FGF12-AS2 | hsa-mir-506 | VIM |
| BPESC1 | hsa-mir-506 | VIM |
| LINC00472 | hsa-mir-506 | VIM |
| GAS6-AS1 | hsa-mir-506 | VIM |
| LINC00426 | hsa-mir-506 | VIM |
| TPTEP1 | hsa-mir-506 | SLC16A1 |
| C15orf56 | hsa-mir-506 | SLC16A1 |
| CSNK1G2-AS1 | hsa-mir-506 | SLC16A1 |
| LINC00221 | hsa-mir-506 | SLC16A1 |
| C20orf203 | hsa-mir-506 | SLC16A1 |
| RNU12 | hsa-mir-506 | SLC16A1 |
| LY86-AS1 | hsa-mir-506 | SLC16A1 |
| LINC00343 | hsa-mir-506 | SLC16A1 |
| PCGEM1 | hsa-mir-506 | SLC16A1 |
| LINC00355 | hsa-mir-506 | SLC16A1 |
| LINC00200 | hsa-mir-506 | SLC16A1 |
| STEAP3-AS1 | hsa-mir-506 | SLC16A1 |
| TPRG1-AS2 | hsa-mir-506 | SLC16A1 |
| FGF12-AS2 | hsa-mir-506 | SLC16A1 |
| BPESC1 | hsa-mir-506 | SLC16A1 |
| LINC00472 | hsa-mir-506 | SLC16A1 |
| GAS6-AS1 | hsa-mir-506 | SLC16A1 |
| LINC00426 | hsa-mir-506 | SLC16A1 |

**Table S5. Seventeen lncRNAs associated with overall survival in KIRC**

| **Gene** | **HR** | **z** | **P value** |
| --- | --- | --- | --- |
| LINC00460 | 1.166721593 | 5.986566411 | 2.14E-09 |
| STEAP3-AS1 | 1.302736021 | 5.779170053 | 7.51E-09 |
| MIAT | 1.26417884 | 5.693565276 | 1.24E-08 |
| COL18A1-AS1 | 0.757611155 | -5.464946124 | 4.63E-08 |
| HM13-IT1 | 1.467834049 | 4.83495778 | 1.33E-06 |
| C20orf203 | 1.257192189 | 4.828996779 | 1.37E-06 |
| RNU12 | 1.234525047 | 4.22684623 | 2.37E-05 |
| SNHG12 | 1.339172155 | 4.104698378 | 4.05E-05 |
| PHEX-AS1 | 1.244332957 | 3.964298373 | 7.36E-05 |
| C1orf147 | 1.264500655 | 3.863993821 | 0.000111548 |
| MIR155HG | 1.236035732 | 3.836108272 | 0.000124999 |
| KCNH1-IT1 | 1.317347146 | 3.805923367 | 0.000141276 |
| LINC00443 | 0.740636768 | -3.707395426 | 0.000209402 |
| FAM13A-AS1 | 1.335926554 | 3.702917098 | 0.000213135 |
| TPRG1-AS2 | 1.373983686 | 3.623341612 | 0.000290821 |
| C5orf58 | 1.273872276 | 3.505375231 | 0.000455964 |
| LINC00472 | 0.78440945 | -3.380721105 | 0.000722959 |

**Table S6. The results of GSEA**

| ID | Set Size | NES | P value |
| --- | --- | --- | --- |
| GO HUMORAL IMMUNE RESPONSE MEDIATED BY CIRCULATING IMMUNOGLOBULIN | 136 | 3.512138 | 0.001656 |
| GO B CELL MEDIATED IMMUNITY | 199 | 3.363956 | 0.001645 |
| GO COMPLEMENT ACTIVATION | 156 | 3.214374 | 0.001616 |
| GO REGULATION OF HUMORAL IMMUNE RESPONSE | 124 | 3.172078 | 0.001706 |
| GO LYMPHOCYTE MEDIATED IMMUNITY | 327 | 3.106806 | 0.001618 |
| GO ADAPTIVE IMMUNE RESPONSE BASED ON SOMATIC RECOMBINATION OF IMMUNE RECEPTORS BUILT FROM IMMUNOGLOBULIN SUPERFAMILY DOMAINS | 335 | 3.088828 | 0.001605 |
| GO POSITIVE REGULATION OF B CELL ACTIVATION | 124 | 3.07926 | 0.001706 |
| GO IMMUNOGLOBULIN PRODUCTION | 171 | 3.072804 | 0.001637 |
| GO REGULATION OF B CELL ACTIVATION | 167 | 3.054455 | 0.001637 |
| GO B CELL RECEPTOR SIGNALING PATHWAY | 111 | 3.032187 | 0.001669 |
| GO PHAGOCYTOSIS RECOGNITION | 70 | 3.018144 | 0.001838 |
| GO HUMORAL IMMUNE RESPONSE | 305 | 2.976299 | 0.001634 |
| GO POSITIVE REGULATION OF LYMPHOCYTE ACTIVATION | 310 | 2.88619 | 0.001634 |
| GO POSITIVE REGULATION OF CELL ACTIVATION | 364 | 2.83204 | 0.00155 |
| GO B CELL ACTIVATION | 272 | 2.776456 | 0.00161 |
| GO PHAGOCYTOSIS | 338 | 2.771825 | 0.00161 |
| GO MEMBRANE INVAGINATION | 121 | 2.768312 | 0.001664 |
| GO PRODUCTION OF MOLECULAR MEDIATOR OF IMMUNE RESPONSE | 259 | 2.756165 | 0.001639 |
| GO DEFENSE RESPONSE TO BACTERIUM | 250 | 2.744581 | 0.001664 |
| GO FC RECEPTOR MEDIATED STIMULATORY SIGNALING PATHWAY | 141 | 2.741822 | 0.00165 |
| GO REGULATION OF LYMPHOCYTE ACTIVATION | 450 | 2.720867 | 0.001534 |
| GO DNA DEPENDENT DNA REPLICATION | 140 | 2.677057 | 0.001631 |
| GO ANTIGEN RECEPTOR MEDIATED SIGNALING PATHWAY | 292 | 2.640419 | 0.001634 |
| GO IMMUNE RESPONSE REGULATING CELL SURFACE RECEPTOR SIGNALING PATHWAY | 475 | 2.598128 | 0.00152 |
| GO MITOTIC SISTER CHROMATID SEGREGATION | 146 | 2.579567 | 0.001637 |
| GO REGULATORY T CELL DIFFERENTIATION | 29 | 2.561322 | 0.001828 |
| GO REGULATION OF IMMUNE EFFECTOR PROCESS | 432 | 2.560599 | 0.001555 |
| GO POSITIVE REGULATION OF LEUKOCYTE CELL CELL ADHESION | 208 | 2.551824 | 0.001664 |
| GO CHROMOSOME SEPARATION | 86 | 2.544666 | 0.001745 |
| GO DEFENSE RESPONSE TO OTHER ORGANISM | 465 | 2.502197 | 0.001536 |
| GO REGULATION OF CHROMOSOME SEPARATION | 60 | 2.49298 | 0.001883 |
| GO FC EPSILON RECEPTOR SIGNALING PATHWAY | 165 | 2.484659 | 0.001626 |
| GO REGULATION OF CHROMOSOME SEGREGATION | 98 | 2.466944 | 0.001701 |
| GO SISTER CHROMATID SEGREGATION | 180 | 2.465213 | 0.001692 |
| GO CHROMOSOME SEGREGATION | 293 | 2.456452 | 0.001616 |
| GO MITOTIC NUCLEAR DIVISION | 272 | 2.447341 | 0.00161 |
| GO MEIOTIC CELL CYCLE PROCESS | 171 | 2.446335 | 0.001637 |
| GO INTERFERON GAMMA PRODUCTION | 106 | 2.443153 | 0.001664 |
| GO METAPHASE ANAPHASE TRANSITION OF CELL CYCLE | 55 | 2.441475 | 0.001866 |
| GO POSITIVE REGULATION OF INTERFERON GAMMA PRODUCTION | 63 | 2.436134 | 0.001894 |
| GO NUCLEAR CHROMOSOME SEGREGATION | 243 | 2.434693 | 0.001672 |
| GO CELL RECOGNITION | 195 | 2.426229 | 0.001675 |
| GO LEUKOCYTE MIGRATION | 470 | 2.418368 | 0.001536 |
| GO POSITIVE REGULATION OF CELL CELL ADHESION | 244 | 2.408196 | 0.001667 |
| GO FC RECEPTOR SIGNALING PATHWAY | 237 | 2.408087 | 0.001695 |
| GO DNA REPLICATION | 260 | 2.406958 | 0.001629 |
| GO MITOTIC SPINDLE ORGANIZATION | 101 | 2.39507 | 0.001686 |
| GO POSITIVE REGULATION OF LYMPHOCYTE DIFFERENTIATION | 90 | 2.388972 | 0.001761 |
| GO KINETOCHORE ORGANIZATION | 19 | 2.38743 | 0.001887 |
| GO COLLAGEN FIBRIL ORGANIZATION | 51 | 2.38692 | 0.001845 |
| GO T CELL ACTIVATION | 429 | 2.37961 | 0.001553 |
| GO REGULATION OF T CELL ACTIVATION | 297 | 2.36665 | 0.00161 |
| GO MICROTUBULE CYTOSKELETON ORGANIZATION INVOLVED IN MITOSIS | 122 | 2.362653 | 0.001678 |
| GO LEUKOCYTE CELL CELL ADHESION | 316 | 2.352301 | 0.00161 |
| GO REGULATION OF SISTER CHROMATID SEGREGATION | 78 | 2.345954 | 0.001828 |
| GO LYMPHOCYTE COSTIMULATION | 56 | 2.331319 | 0.001862 |
| GO REGULATION OF ADAPTIVE IMMUNE RESPONSE | 153 | 2.328063 | 0.001629 |
| GO POSITIVE REGULATION OF ALPHA BETA T CELL ACTIVATION | 61 | 2.324568 | 0.001908 |
| GO B CELL PROLIFERATION | 78 | 2.319446 | 0.001828 |
| GO CELL CYCLE DNA REPLICATION | 63 | 2.31718 | 0.001894 |
| GO MITOTIC CELL CYCLE CHECKPOINT | 157 | 2.316969 | 0.001626 |
| GO REGULATION OF LEUKOCYTE PROLIFERATION | 210 | 2.313157 | 0.001692 |
| GO NEGATIVE REGULATION OF CHROMOSOME SEGREGATION | 43 | 2.312462 | 0.001845 |
| GO INTERLEUKIN 1 SECRETION | 53 | 2.311015 | 0.001855 |
| GO POSITIVE REGULATION OF LEUKOCYTE PROLIFERATION | 131 | 2.309215 | 0.001669 |
| GO ALPHA BETA T CELL ACTIVATION | 130 | 2.30555 | 0.001681 |
| GO MEIOSIS I CELL CYCLE PROCESS | 110 | 2.304448 | 0.001664 |
| GO POSITIVE REGULATION OF T CELL PROLIFERATION | 90 | 2.300662 | 0.001761 |
| GO T CELL DIFFERENTIATION | 226 | 2.299979 | 0.001698 |
| GO REGULATION OF CD4 POSITIVE ALPHA BETA T CELL DIFFERENTIATION | 46 | 2.296062 | 0.001808 |
| GO SPINDLE ORGANIZATION | 162 | 2.286245 | 0.001653 |
| GO MEIOTIC CHROMOSOME SEGREGATION | 77 | 2.28486 | 0.001812 |
| GO LYMPHOCYTE DIFFERENTIATION | 324 | 2.283255 | 0.001626 |
| GO REGULATION OF LYMPHOCYTE DIFFERENTIATION | 159 | 2.269896 | 0.001658 |
| GO ALPHA BETA T CELL DIFFERENTIATION | 99 | 2.267618 | 0.001695 |
| GO NUCLEAR DNA REPLICATION | 54 | 2.267138 | 0.001848 |
| GO REGULATION OF NUCLEAR DIVISION | 201 | 2.266799 | 0.001656 |
| GO REGULATION OF ALPHA BETA T CELL ACTIVATION | 89 | 2.263864 | 0.001757 |
| GO COLLAGEN CATABOLIC PROCESS | 46 | 2.259207 | 0.001808 |
| GO CELL CYCLE CHECKPOINT | 205 | 2.256675 | 0.001689 |
| GO CENTROMERE COMPLEX ASSEMBLY | 50 | 2.255486 | 0.001825 |
| GO POSITIVE REGULATION OF ANTIGEN RECEPTOR MEDIATED SIGNALING PATHWAY | 21 | 2.253723 | 0.001821 |
| GO PROTEIN LOCALIZATION TO CHROMOSOME CENTROMERIC REGION | 20 | 2.252163 | 0.001883 |
| GO LYMPHOCYTE CHEMOTAXIS | 58 | 2.251626 | 0.001876 |
| GO MEIOTIC CELL CYCLE | 232 | 2.246345 | 0.001704 |
| GO ORGANELLE FISSION | 435 | 2.244191 | 0.001541 |
| GO LEUKOCYTE PROLIFERATION | 267 | 2.242411 | 0.001631 |
| GO NEGATIVE REGULATION OF METAPHASE ANAPHASE TRANSITION OF CELL CYCLE | 36 | 2.242239 | 0.001821 |
| GO INTERLEUKIN 1 BETA SECRETION | 46 | 2.238934 | 0.001808 |
| GO POSITIVE REGULATION OF LEUKOCYTE DIFFERENTIATION | 138 | 2.235929 | 0.001656 |
| GO DNA REPLICATION INDEPENDENT NUCLEOSOME ORGANIZATION | 51 | 2.231974 | 0.001845 |
| GO LYMPHOCYTE ACTIVATION INVOLVED IN IMMUNE RESPONSE | 155 | 2.231332 | 0.001623 |
| GO REGULATION OF LYMPHOCYTE MEDIATED IMMUNITY | 143 | 2.229143 | 0.001647 |
| GO POSITIVE REGULATION OF INTERLEUKIN 1 SECRETION | 34 | 2.228295 | 0.001842 |
| GO T CELL SELECTION | 48 | 2.227212 | 0.001838 |
| GO ACUTE INFLAMMATORY RESPONSE | 101 | 2.223202 | 0.001686 |
| GO REGULATION OF CD4 POSITIVE ALPHA BETA T CELL ACTIVATION | 55 | 2.220326 | 0.001866 |
| GO CHRONIC INFLAMMATORY RESPONSE | 18 | 2.220168 | 0.001898 |
| GO NEGATIVE REGULATION OF LYMPHOCYTE ACTIVATION | 133 | 2.215075 | 0.001672 |
| GO REGULATION OF T CELL DIFFERENTIATION | 130 | 2.214549 | 0.001681 |
| GO PROTEIN HETEROTRIMERIZATION | 13 | 2.210605 | 0.001931 |
| GO REGULATION OF ALPHA BETA T CELL DIFFERENTIATION | 62 | 2.208237 | 0.001908 |
| GO DNA DEPENDENT DNA REPLICATION MAINTENANCE OF FIDELITY | 38 | 2.207748 | 0.001825 |
| GO REGULATION OF LYMPHOCYTE CHEMOTAXIS | 26 | 2.203655 | 0.001815 |
| GO T CELL ACTIVATION INVOLVED IN IMMUNE RESPONSE | 85 | 2.200628 | 0.001792 |
| GO CHROMATIN REMODELING AT CENTROMERE | 42 | 2.198311 | 0.001859 |
| GO PROTEIN LOCALIZATION TO KINETOCHORE | 15 | 2.197904 | 0.00188 |
| GO REGULATION OF DNA DEPENDENT DNA REPLICATION | 49 | 2.196476 | 0.001821 |
| GO CD4 POSITIVE ALPHA BETA T CELL LINEAGE COMMITMENT | 17 | 2.193727 | 0.001898 |
| GO LEUKOCYTE APOPTOTIC PROCESS | 101 | 2.192853 | 0.001686 |
| GO CD4 POSITIVE ALPHA BETA T CELL ACTIVATION | 84 | 2.189352 | 0.001773 |
| GO POSITIVE REGULATION OF ALPHA BETA T CELL PROLIFERATION | 19 | 2.187891 | 0.001887 |
| GO NEGATIVE REGULATION OF NUCLEAR DIVISION | 58 | 2.18477 | 0.001876 |
| GO LEUKOCYTE CHEMOTAXIS | 206 | 2.181826 | 0.001678 |
| GO T CELL CHEMOTAXIS | 25 | 2.180624 | 0.001845 |
| GO RESPONSE TO CHEMOKINE | 92 | 2.174697 | 0.00173 |
| GO LEUKOCYTE DIFFERENTIATION | 476 | 2.174586 | 0.001529 |
| GO NEGATIVE T CELL SELECTION | 12 | 2.173441 | 0.001908 |
| GO T HELPER 17 CELL LINEAGE COMMITMENT | 12 | 2.172301 | 0.001908 |
| GO POSITIVE REGULATION OF CD4 POSITIVE ALPHA BETA T CELL ACTIVATION | 34 | 2.1705 | 0.001842 |
| GO T CELL PROLIFERATION | 172 | 2.170007 | 0.001653 |
| GO POSITIVE REGULATION OF CYTOKINE PRODUCTION | 424 | 2.163174 | 0.001548 |
| GO POSITIVE REGULATION OF ALPHA BETA T CELL DIFFERENTIATION | 46 | 2.159663 | 0.001808 |
| GO DNA REPLICATION INITIATION | 37 | 2.159422 | 0.001805 |
| GO T CELL DIFFERENTIATION INVOLVED IN IMMUNE RESPONSE | 65 | 2.15907 | 0.001876 |
| GO RECOMBINATIONAL REPAIR | 118 | 2.158663 | 0.001672 |
| GO POSITIVE REGULATION OF ADAPTIVE IMMUNE RESPONSE | 99 | 2.15757 | 0.001695 |
| GO REGULATION OF B CELL PROLIFERATION | 59 | 2.144091 | 0.00188 |
| GO CELLULAR DEFENSE RESPONSE | 52 | 2.142285 | 0.001852 |
| GO INTERFERON GAMMA MEDIATED SIGNALING PATHWAY | 87 | 2.138413 | 0.001754 |
| GO SPINDLE MIDZONE ASSEMBLY | 10 | 2.135064 | 0.002004 |
| GO POSITIVE REGULATION OF INTERLEUKIN 1 PRODUCTION | 47 | 2.130823 | 0.001835 |
| GO REGULATION OF LEUKOCYTE APOPTOTIC PROCESS | 81 | 2.129325 | 0.001808 |
| GO CYTOKINE METABOLIC PROCESS | 108 | 2.126632 | 0.001642 |
| GO MEIOTIC CHROMOSOME SEPARATION | 22 | 2.126515 | 0.001828 |
| GO POSITIVE T CELL SELECTION | 35 | 2.125429 | 0.001812 |
| GO HISTONE EXCHANGE | 52 | 2.125287 | 0.001852 |
| GO NEUTROPHIL MIGRATION | 106 | 2.124738 | 0.001664 |
| GO REPLICATION FORK PROCESSING | 28 | 2.122141 | 0.001825 |
| GO NEGATIVE REGULATION OF CHROMOSOME ORGANIZATION | 137 | 2.119081 | 0.001661 |
| GO REGULATION OF B CELL RECEPTOR SIGNALING PATHWAY | 28 | 2.115887 | 0.001825 |
| GO REGULATION OF LEUKOCYTE MEDIATED IMMUNITY | 193 | 2.115778 | 0.001656 |
| GO INTERLEUKIN 17 PRODUCTION | 27 | 2.115264 | 0.001818 |
| GO POSITIVE REGULATION OF RESPONSE TO CYTOKINE STIMULUS | 54 | 2.11389 | 0.001848 |
| GO REGULATION OF DNA REPLICATION | 102 | 2.113469 | 0.001658 |
| GO REGULATION OF ANTIGEN RECEPTOR MEDIATED SIGNALING PATHWAY | 61 | 2.110065 | 0.001908 |
| GO REGULATION OF RESPONSE TO CYTOKINE STIMULUS | 162 | 2.107814 | 0.001653 |
| GO REGULATION OF B CELL MEDIATED IMMUNITY | 49 | 2.106799 | 0.001821 |
| GO REGULATION OF T HELPER CELL DIFFERENTIATION | 34 | 2.105396 | 0.001842 |
| GO POSITIVE REGULATION OF CELL CYCLE PHASE TRANSITION | 94 | 2.102683 | 0.001701 |
| GO NEGATIVE REGULATION OF IMMUNE RESPONSE | 132 | 2.101875 | 0.001661 |
| GO MITOTIC SPINDLE ASSEMBLY | 53 | 2.101709 | 0.001855 |
| GO REGULATION OF CELL CELL ADHESION | 374 | 2.099338 | 0.001527 |
| GO REGULATION OF ANTIGEN PROCESSING AND PRESENTATION | 18 | 2.098611 | 0.001898 |
| GO FEMALE MEIOTIC NUCLEAR DIVISION | 27 | 2.096383 | 0.001818 |
| GO REGULATION OF LYMPHOCYTE APOPTOTIC PROCESS | 53 | 2.094339 | 0.001855 |
| GO PROTEIN LOCALIZATION TO CHROMOSOME | 80 | 2.093556 | 0.001805 |
| GO KINETOCHORE ASSEMBLY | 14 | 2.092232 | 0.001969 |
| GO REGULATION OF LEUKOCYTE DIFFERENTIATION | 254 | 2.086885 | 0.001653 |
| GO REGULATION OF LEUKOCYTE CHEMOTAXIS | 107 | 2.083726 | 0.00165 |
| GO DNA INTEGRITY CHECKPOINT | 148 | 2.082741 | 0.0016 |
| GO DNA RECOMBINATION | 262 | 2.081303 | 0.001618 |
| GO ANTIMICROBIAL HUMORAL RESPONSE | 104 | 2.080467 | 0.001661 |
| GO NATURAL KILLER CELL MEDIATED IMMUNITY | 62 | 2.080148 | 0.001908 |
| GO POSITIVE REGULATION OF INTERLEUKIN 4 PRODUCTION | 23 | 2.078971 | 0.001845 |
| GO REGULATION OF INNATE IMMUNE RESPONSE | 413 | 2.078387 | 0.001558 |
| GO REGULATION OF B CELL APOPTOTIC PROCESS | 18 | 2.077267 | 0.001898 |
| GO DETECTION OF BIOTIC STIMULUS | 20 | 2.075427 | 0.001883 |
| GO GRANULOCYTE MIGRATION | 128 | 2.074834 | 0.001686 |
| GO NEGATIVE REGULATION OF CELL ACTIVATION | 179 | 2.073048 | 0.001672 |
| GO REGULATION OF T CELL CHEMOTAXIS | 16 | 2.071874 | 0.00189 |
| GO LYMPHOCYTE MIGRATION | 100 | 2.069506 | 0.001698 |
| GO NEGATIVE REGULATION OF LEUKOCYTE APOPTOTIC PROCESS | 48 | 2.068974 | 0.001838 |
| GO LYMPHOCYTE APOPTOTIC PROCESS | 68 | 2.066004 | 0.001835 |
| GO POSITIVE REGULATION OF LEUKOCYTE MIGRATION | 119 | 2.063899 | 0.001672 |
| GO T HELPER 1 TYPE IMMUNE RESPONSE | 41 | 2.063001 | 0.001855 |
| GO REGULATION OF TRANSCRIPTION INVOLVED IN G1 S TRANSITION OF MITOTIC CELL CYCLE | 29 | 2.062505 | 0.001828 |
| GO ATTACHMENT OF SPINDLE MICROTUBULES TO KINETOCHORE | 29 | 2.06227 | 0.001828 |
| GO NEGATIVE REGULATION OF EXTRINSIC APOPTOTIC SIGNALING PATHWAY VIA DEATH DOMAIN RECEPTORS | 36 | 2.062237 | 0.001821 |
| GO CHONDROCYTE DEVELOPMENT | 45 | 2.06095 | 0.001821 |
| GO LYMPHOCYTE HOMEOSTASIS | 63 | 2.055671 | 0.001894 |
| GO NEGATIVE REGULATION OF B CELL APOPTOTIC PROCESS | 12 | 2.051869 | 0.001908 |
| GO MYELOID DENDRITIC CELL ACTIVATION | 30 | 2.050275 | 0.001815 |
| GO DETECTION OF EXTERNAL BIOTIC STIMULUS | 17 | 2.046969 | 0.001898 |
| GO SPINDLE ASSEMBLY | 103 | 2.044136 | 0.001667 |
| GO REGULATION OF DOUBLE STRAND BREAK REPAIR VIA HOMOLOGOUS RECOMBINATION | 38 | 2.043297 | 0.001825 |
| GO SPINDLE ELONGATION | 10 | 2.043242 | 0.002004 |
| GO NEGATIVE REGULATION OF LEUKOCYTE PROLIFERATION | 75 | 2.042204 | 0.001818 |
| GO RESPONSE TO INTERLEUKIN 12 | 49 | 2.042142 | 0.001821 |
| GO POSITIVE REGULATION OF INTERLEUKIN 10 PRODUCTION | 30 | 2.042076 | 0.001815 |
| GO RESPONSE TO INTERFERON GAMMA | 191 | 2.041399 | 0.001675 |
| GO RECEPTOR MEDIATED ENDOCYTOSIS | 345 | 2.036577 | 0.001595 |
| GO CELL KILLING | 141 | 2.035196 | 0.00165 |
| GO INTERLEUKIN 10 PRODUCTION | 47 | 2.033494 | 0.001835 |
| GO LEUKOCYTE MEDIATED CYTOTOXICITY | 105 | 2.033454 | 0.001656 |
| GO CELL CHEMOTAXIS | 276 | 2.031864 | 0.001595 |
| GO POSITIVE REGULATION OF DNA REPLICATION | 36 | 2.03119 | 0.001821 |
| GO REGULATION OF LEUKOCYTE MIGRATION | 181 | 2.03055 | 0.001669 |
| GO MITOTIC CHROMOSOME CONDENSATION | 16 | 2.029587 | 0.00189 |
| GO DOUBLE STRAND BREAK REPAIR | 220 | 2.029513 | 0.001658 |
| GO REGULATION OF INTERLEUKIN 4 PRODUCTION | 28 | 2.029416 | 0.001825 |
| GO INTERLEUKIN 1 PRODUCTION | 87 | 2.02912 | 0.001754 |
| GO DNA REPLICATION CHECKPOINT | 16 | 2.027102 | 0.00189 |
| GO DETECTION OF MOLECULE OF BACTERIAL ORIGIN | 11 | 2.027089 | 0.001969 |
| GO POSITIVE REGULATION OF LEUKOCYTE CHEMOTAXIS | 81 | 2.026714 | 0.001808 |
| GO POSITIVE REGULATION OF REGULATORY T CELL DIFFERENTIATION | 13 | 2.026141 | 0.001931 |
| GO POSITIVE REGULATION OF LYMPHOCYTE CHEMOTAXIS | 20 | 2.026016 | 0.001883 |
| GO NATURAL KILLER CELL ACTIVATION | 71 | 2.025724 | 0.001845 |
| GO REGULATION OF T HELPER 1 TYPE IMMUNE RESPONSE | 27 | 2.025135 | 0.001818 |
| GO POSITIVE REGULATION OF CELL ADHESION | 389 | 2.02405 | 0.001546 |
| GO REGULATION OF CELL CYCLE G2 M PHASE TRANSITION | 203 | 2.019476 | 0.001678 |
| GO POSITIVE REGULATION OF RECEPTOR SIGNALING PATHWAY VIA STAT | 82 | 2.017983 | 0.001773 |
| GO NEGATIVE REGULATION OF B CELL ACTIVATION | 31 | 2.01597 | 0.001859 |
| GO INTERLEUKIN 1 BETA PRODUCTION | 74 | 2.015108 | 0.001821 |
| GO TUMOR NECROSIS FACTOR MEDIATED SIGNALING PATHWAY | 159 | 2.014727 | 0.001658 |
| GO PEPTIDE CROSS LINKING | 36 | 2.013875 | 0.001821 |
| GO RESPONSE TO TYPE I INTERFERON | 81 | 2.013016 | 0.001808 |
| GO POSITIVE REGULATION OF T CELL APOPTOTIC PROCESS | 11 | 2.012548 | 0.001969 |
| GO POSITIVE REGULATION OF CYTOKINE BIOSYNTHETIC PROCESS | 60 | 2.011942 | 0.001883 |
| GO INFLAMMATORY RESPONSE TO ANTIGENIC STIMULUS | 41 | 2.009254 | 0.001855 |
| GO THYMIC T CELL SELECTION | 21 | 2.007996 | 0.001821 |
| GO ACUTE PHASE RESPONSE | 44 | 2.005859 | 0.001842 |
| GO POSITIVE REGULATION OF CELL CYCLE PROCESS | 272 | 2.00038 | 0.00161 |
| GO COENZYME BIOSYNTHETIC PROCESS | 138 | -2.00744 | 0.002513 |
| GO THIOESTER METABOLIC PROCESS | 49 | -2.01403 | 0.002208 |
| GO MITOCHONDRIAL PROTEIN PROCESSING | 12 | -2.01405 | 0.004184 |
| GO MITOCHONDRIAL TRANSMEMBRANE TRANSPORT | 73 | -2.01793 | 0.002212 |
| GO RENAL SYSTEM PROCESS | 114 | -2.02902 | 0.002532 |
| GO TUBULIN DEACETYLATION | 11 | -2.03087 | 0.004049 |
| GO ACETYL COA BIOSYNTHETIC PROCESS FROM PYRUVATE | 15 | -2.0358 | 0.002128 |
| GO FATTY ACID BETA OXIDATION USING ACYL COA DEHYDROGENASE | 10 | -2.03949 | 0.001988 |
| GO GLUTAMINE FAMILY AMINO ACID CATABOLIC PROCESS | 27 | -2.03967 | 0.002212 |
| GO COENZYME METABOLIC PROCESS | 247 | -2.05633 | 0.002451 |
| GO REGULATION OF SODIUM ION TRANSMEMBRANE TRANSPORT | 59 | -2.05655 | 0.002128 |
| GO INTRACILIARY TRANSPORT INVOLVED IN CILIUM ASSEMBLY | 40 | -2.05748 | 0.00216 |
| GO ENERGY DERIVATION BY OXIDATION OF ORGANIC COMPOUNDS | 258 | -2.0602 | 0.002564 |
| GO INTRACILIARY TRANSPORT | 52 | -2.06061 | 0.002165 |
| GO XENOBIOTIC CATABOLIC PROCESS | 11 | -2.06564 | 0.002024 |
| GO COFACTOR BIOSYNTHETIC PROCESS | 201 | -2.07262 | 0.002513 |
| GO MITOCHONDRIAL CALCIUM ION TRANSMEMBRANE TRANSPORT | 23 | -2.0929 | 0.002174 |
| GO TRANSLATIONAL TERMINATION | 103 | -2.09511 | 0.002488 |
| GO CYTOSKELETAL ANCHORING AT PLASMA MEMBRANE | 10 | -2.09988 | 0.001988 |
| GO DICARBOXYLIC ACID METABOLIC PROCESS | 60 | -2.1354 | 0.002123 |
| GO COFACTOR METABOLIC PROCESS | 431 | -2.14201 | 0.002833 |
| GO NUCLEOSIDE BISPHOSPHATE BIOSYNTHETIC PROCESS | 66 | -2.14796 | 0.002179 |
| GO THIOESTER BIOSYNTHETIC PROCESS | 51 | -2.14797 | 0.002174 |
| GO ORGANIC CATION TRANSPORT | 35 | -2.16316 | 0.002222 |
| GO MONOCARBOXYLIC ACID METABOLIC PROCESS | 457 | -2.17761 | 0.00277 |
| GO ENDOTHELIUM DEVELOPMENT | 113 | -2.19646 | 0.002519 |
| GO ATP SYNTHESIS COUPLED ELECTRON TRANSPORT | 83 | -2.20823 | 0.002283 |
| GO RESPIRATORY ELECTRON TRANSPORT CHAIN | 101 | -2.22439 | 0.002445 |
| GO MITOCHONDRIAL TRANSLATIONAL TERMINATION | 88 | -2.24019 | 0.002288 |
| GO FATTY ACID METABOLIC PROCESS | 249 | -2.24207 | 0.002475 |
| GO CELLULAR LIPID CATABOLIC PROCESS | 203 | -2.26203 | 0.002463 |
| GO MITOCHONDRIAL TRANSLATION | 133 | -2.26447 | 0.002475 |
| GO BRANCHED CHAIN AMINO ACID CATABOLIC PROCESS | 19 | -2.27725 | 0.002119 |
| GO MITOCHONDRIAL GENE EXPRESSION | 157 | -2.28805 | 0.002584 |
| GO OXIDATIVE PHOSPHORYLATION | 106 | -2.29556 | 0.002494 |
| GO ALPHA AMINO ACID CATABOLIC PROCESS | 77 | -2.29987 | 0.002222 |
| GO ACETYL COA BIOSYNTHETIC PROCESS | 20 | -2.31938 | 0.002123 |
| GO LIPID MODIFICATION | 231 | -2.36291 | 0.002387 |
| GO NADH DEHYDROGENASE COMPLEX ASSEMBLY | 55 | -2.3941 | 0.002146 |
| GO AEROBIC RESPIRATION | 80 | -2.41374 | 0.002232 |
| GO CELLULAR RESPIRATION | 174 | -2.43786 | 0.002551 |
| GO MITOCHONDRIAL RESPIRATORY CHAIN COMPLEX ASSEMBLY | 87 | -2.4696 | 0.002315 |
| GO TRICARBOXYLIC ACID CYCLE | 33 | -2.47107 | 0.002155 |
| GO MITOCHONDRIAL ELECTRON TRANSPORT NADH TO UBIQUINONE | 45 | -2.51589 | 0.002208 |
| GO PEROXISOMAL TRANSPORT | 70 | -2.55137 | 0.002183 |
| GO SMALL MOLECULE CATABOLIC PROCESS | 387 | -2.62493 | 0.002865 |
| GO FATTY ACID BETA OXIDATION | 71 | -2.66952 | 0.002174 |
| GO PEROXISOME ORGANIZATION | 80 | -2.68081 | 0.002232 |
| GO CELLULAR AMINO ACID CATABOLIC PROCESS | 83 | -2.72926 | 0.002283 |
| GO LIPID OXIDATION | 100 | -2.78545 | 0.002421 |
| GO FATTY ACID CATABOLIC PROCESS | 102 | -2.81366 | 0.002506 |
| GO MONOCARBOXYLIC ACID CATABOLIC PROCESS | 121 | -2.84283 | 0.002494 |
| GO ORGANIC ACID CATABOLIC PROCESS | 246 | -2.90242 | 0.002481 |

**Table S7. The potential binding sites of three lncRNAs (LINC00443, LINC00460 and MIAT) on the targeted DEmiRNAs in the ceRNA network.**

| **lncRNA** | **miRNA** | **Seed position (Seed type)** |
| --- | --- | --- |
| LINC00443 | hsa-mir-141 | chr13:107321013 (7-mer-m8) |
| LINC00460 | hsa-mir-206 | chr13:107030346 (7-mer-A1) |
| MIAT | hsa-mir-141 | chr22:27042712 (7-mer-A1),  chr22:27067365 (7-mer-m8) |
| MIAT | hsa-mir-155 | chr22:27053426 (7-mer-A1) |
| MIAT | hsa-mir-206 | chr22:27069556 (7-mer-m8),  chr22:27072187 (7-mer-A1) |
| MIAT | hsa-mir-216b | chr22:27042620 (7-mer-m8),  chr22:27071885 (7-mer-m8) |
| MIAT | hsa-mir-372 | chr22:27061945 (7-mer-m8) |
